# Diretriz de Miocardites da Sociedade Brasileira de Cardiologia – 2022

**DOI:** 10.36660/abc.20220412

**Published:** 2022-07-07

**Authors:** Marcelo Westerlund Montera, Fabiana G. Marcondes-Braga, Marcus Vinícius Simões, Lídia Ana Zytynski Moura, Fabio Fernandes, Sandrigo Mangine, Amarino Carvalho de Oliveira, Aurea Lucia Alves de Azevedo Grippa de Souza, Bárbara Maria Ianni, Carlos Eduardo Rochitte, Claudio Tinoco Mesquita, Clerio F. de Azevedo, Dhayn Cassi de Almeida Freitas, Dirceu Thiago Pessoa de Melo, Edimar Alcides Bocchi, Estela Suzana Kleiman Horowitz, Evandro Tinoco Mesquita, Guilherme H. Oliveira, Humberto Villacorta, João Manoel Rossi, João Marcos Bemfica Barbosa, José Albuquerque de Figueiredo, Louise Freire Luiz, Ludhmila Abrahão Hajjar, Luis Beck-da-Silva, Luiz Antonio de Almeida Campos, Luiz Cláudio Danzmann, Marcelo Imbroise Bittencourt, Marcelo Iorio Garcia, Monica Samuel Avila, Nadine Oliveira Clausell, Nilson Araujo de Oliveira, Odilson Marcos Silvestre, Olga Ferreira de Souza, Ricardo Mourilhe-Rocha, Roberto Kalil, Sadeer G. Al-Kindi, Salvador Rassi, Silvia Marinho Martins Alves, Silvia Moreira Ayub Ferreira, Stéphanie Itala Rizk, Tiago Azevedo Costa Mattos, Vitor Barzilai, Wolney de Andrade Martins, Heinz-Peter Schultheiss

**Affiliations:** 1 Hospital Pró-Cardíaco Rio de Janeiro RJ Brasil Hospital Pró-Cardíaco, Rio de Janeiro, RJ – Brasil; 2 Hospital das Clínicas Faculdade de Medicina Universidade de São Paulo São Paulo SP Brasil Instituto do Coração (InCor) do Hospital das Clínicas da Faculdade de Medicina da Universidade de São Paulo (HCFMUSP), São Paulo, SP – Brasil; 3 Faculdade de Medicina de Ribeirão Preto Universidade de São Paulo São Paulo SP Brasil Faculdade de Medicina de Ribeirão Preto da Universidade de São Paulo, São Paulo, SP – Brasil; 4 Pontifícia Universidade Católica de Curitiba Curitiba PR Brasil Pontifícia Universidade Católica de Curitiba, Curitiba, PR – Brasil; 5 Universidade Federal Fluminense Rio de Janeiro RJ Brasil Universidade Federal Fluminense,Rio de Janeiro, RJ – Brasil; 6 Instituto do Coração Faculdade de Medicina Universidade de São Paulo São Paulo SP Brasil Instituto do Coração (InCor) – Faculdade de Medicina da Universidade de São Paulo, São Paulo, SP – Brasil; 7 Hospital do Coração São Paulo SP Brasil Hospital do Coração (HCOR), São Paulo, SP – Brasil; 8 Hospital Vitória Rio de Janeiro RJ Brasil Hospital Vitória, Rio de Janeiro, RJ – Brasil; 9 Duke University Durham EUA Duke University, Durham – EUA; 10 Universidade Federal do Acre Rio Branco AC Brasil Universidade Federal do Acre, Rio Branco, AC – Brasil; 11 Hospital do Coração de Campinas Campinas SP Brasil Hospital do Coração de Campinas, Campinas, SP – Brasil; 12 Instituto de Cardiologia do Rio Grande do Sul Fundação Universitária de Cardiologia Porto Alegre RS Brasil Instituto de Cardiologia do Rio Grande do Sul/Fundação Universitária de Cardiologia,Porto Alegre, RS – Brasil; 13 Centro de Ensino e Treinamento Edson de Godoy Bueno UHG Rio de Janeiro RJ Brasil Centro de Ensino e Treinamento Edson de Godoy Bueno / UHG, Rio de Janeiro, RJ – Brasil; 14 University Hospitals Cleveland Medical Center Cleveland Ohio EUA University Hospitals Cleveland Medical Center, Cleveland, Ohio – EUA; 15 Instituto Dante Pazzanese de Cardiologia São Paulo SP Brasil Instituto Dante Pazzanese de Cardiologia, São Paulo, SP – Brasil; 16 Universidade do Estado do Amazonas Boca do Acre AM Brasil Universidade do Estado do Amazonas, Boca do Acre, AM – Brasil; 17 Universidade Federal do Maranhão São Luís MA Brasil Universidade Federal do Maranhão, São Luís, MA – Brasil; 18 Instituto do Câncer do Estado de São Paulo Faculdade de Medicina Universidade de São Paulo São Paulo SP Brasil Instituto do Câncer do Estado de São Paulo da Faculdade de Medicina da Universidade de São Paulo, São Paulo, SP – Brasil; 19 Hospital de Clínicas de Porto Alegre Porto Alegre RS Brasil Hospital de Clínicas de Porto Alegre, Porto Alegre, RS – Brasil; 20 Universidade Federal do Rio Grande do Sul Porto Alegre RS Brasil Universidade Federal do Rio Grande do Sul (UFRGS), Porto Alegre, RS – Brasil; 21 Universidade Luterana do Brasil Canoas RS Brasil Universidade Luterana do Brasil, Canoas, RS – Brasil; 22 Universidade do Estado do Rio de Janeiro Rio de Janeiro RJ Brasil Universidade do Estado do Rio de Janeiro, Rio de Janeiro, RJ – Brasil; 23 Hospital Universitário Pedro Ernesto Rio de Janeiro RJ Brasil Hospital Universitário Pedro Ernesto, Rio de Janeiro, RJ – Brasil; 24 Hospital Universitário Clementino Fraga Filho Universidade Federal do Rio de Janeiro Rio de Janeiro RJ Brasil Hospital Universitário Clementino Fraga Filho (HUCFF) da Universidade Federal do Rio de Janeiro (UFRJ), Rio de Janeiro, RJ – Brasil; 25 Rede Dor São Luiz de Hospitais Rio de Janeiro RJ Brasil Rede Dor São Luiz de Hospitais, Rio de Janeiro, RJ – Brasil; 26 Hospital Sírio Libanês São Paulo SP Brasil Hospital Sírio Libanês, São Paulo, SP – Brasil; 27 Harrington Heart and Vascular Institute, University Hospitals Case Western Reserve University Cleveland Ohio EUA Harrington Heart and Vascular Institute, University Hospitals and Case Western Reserve University,Cleveland, Ohio – EUA; 28 Universidade Federal de Goiás Goiânia GO Brasil Universidade Federal de Goiás, Goiânia, GO – Brasil; 29 Pronto Socorro Cardiológico de Pernambuco Recife PE Brasil Pronto Socorro Cardiológico de Pernambuco (PROCAPE), Recife, PE – Brasil; 30 Universidade de Pernambuco Recife PE Brasil Universidade de Pernambuco (UPE), Recife, PE – Brasil; 31 Instituto de Cardiologia do Distrito Federal Brasília DF Brasil Instituto de Cardiologia do Distrito Federal, Brasília, DF – Brasil; 32 DASA Complexo Hospitalar de Niterói Niterói RJ Brasil DASA Complexo Hospitalar de Niterói, Niterói, RJ – Brasil; 33 Institute of Cardiac Diagnostics and Therapy Berlim Alemanha Institute of Cardiac Diagnostics and Therapy (IKDT), Berlim – Alemanha

**Table:** 

Diretriz de Miocardites da Sociedade Brasileira de Cardiologia – 2022
O relatório abaixo lista as declarações de interesse conforme relatadas à SBC pelos especialistas durante o período de desenvolvimento deste posicionamento, 2021.
Especialista	Tipo de relacionamento com a indústria
Amarino Carvalho de Oliveira Júnior	Nada a ser declarado
Aurea Lucia Alves de Azevedo Grippa de Souza	Outros relacionamentos Participação societária de qualquer natureza e qualquer valor economicamente apreciável de empresas na área de saúde, de ensino ou em empresas concorrentes ou fornecedoras da SBC: – Cardiologia: Curso PROKIDS Assistência Multidisciplinar LTDA
Bárbara Maria Ianni	Nada a ser declarado
Carlos Eduardo Rochitte	Nada a ser declarado
Claudio Tinoco Mesquita	Declaração financeira A - Pagamento de qualquer espécie e desde que economicamente apreciáveis, feitos a (i) você, (ii) ao seu cônjuge/ companheiro ou a qualquer outro membro que resida com você, (iii) a qualquer pessoa jurídica em que qualquer destes seja controlador, sócio, acionista ou participante, de forma direta ou indireta, recebimento por palestras, aulas, atuação como proctor de treinamentos, remunerações, honorários pagos por participações em conselhos consultivos, de investigadores,ou outros comitês, etc. Provenientes da indústria farmacêutica, de órteses, próteses, equipamentos e implantes,brasileiras ou estrangeiras: - Servier: Vastarel. B - Financiamento de pesquisas sob sua responsabilidade direta/pessoal (direcionado ao departamento ou instituição) provenientes da indústria farmacêutica, de órteses, próteses, equipamentos e implantes, brasileiras ou estrangeiras: - Alnylan: Onpatro.
Clerio F. Azevedo	Nada a ser declarado
Dhayn Cassi de Almeida Freitas	Nada a ser declarado
Dirceu Thiago Pessoa de Melo	Declaração financeira A - Pagamento de qualquer espécie e desde que economicamente apreciáveis, feitos a (i) você, (ii) ao seu cônjuge/ companheiro ou a qualquer outro membro que resida com você, (iii) a qualquer pessoa jurídica em que qualquer destes seja controlador, sócio, acionista ou participante, de forma direta ou indireta, recebimento por palestras, aulas, atuação como proctor de treinamentos, remunerações, honorários pagos por participações em conselhos consultivos, de investigadores,ou outros comitês, etc. Provenientes da indústria farmacêutica, de órteses, próteses, equipamentos e implantes,brasileiras ou estrangeiras: - Daiichi Sankyo. Outros relacionamentos Financiamento de atividades de educação médica continuada, incluindo viagens, hospedagens e inscrições para congressos e cursos, provenientes da indústria farmacêutica, de órteses, próteses, equipamentos e implantes, brasileiras ou estrangeiras: - Pfizer.
Edimar Alcides Bocchi	Declaração financeira A - Pagamento de qualquer espécie e desde que economicamente apreciáveis, feitos a (i) você, (ii) ao seu cônjuge/ companheiro ou a qualquer outro membro que resida com você, (iii) a qualquer pessoa jurídica em que qualquer destes seja controlador, sócio, acionista ou participante, de forma direta ou indireta, recebimento por palestras, aulas, atuação como proctor de treinamentos, remunerações, honorários pagos por participações em conselhos consultivos, de investigadores,ou outros comitês, etc. Provenientes da indústria farmacêutica, de órteses, próteses, equipamentos e implantes,brasileiras ou estrangeiras: - AstraZeneca: ISGLT2; Bayer: ISGLT2, Vericiguat; Boehringer: ISGLT2.
Estela Suzana Kleiman Horowitz	Declaração financeira B - financiamento de pesquisas sob sua responsabilidade direta/pessoal (direcionado ao departamento ou instituição) provenientes da indústria farmacêutica, de órteses, próteses, equipamentos e implantes, brasileiras ou estrangeiras: - Jansen: Rivaroxabana. C - Financiamento de pesquisa (pessoal), cujas receitas tenham sido provenientes da indústria farmacêutica, de órteses, próteses, equipamentos e implantes, brasileiras ou estrangeiras: - Jansen: Rivaroxabana.
Evandro Tinoco Mesquita	Outros relacionamentos Vínculo empregatício com a indústria farmacêutica, de órteses, próteses, Equipamentos e implantes, brasileiras ou estrangeiras, assim como se tem relação vínculo empregatício com operadoras de planos de saúde ou em auditorias médicas (incluindo meio período) durante o ano para o qual você está declarando: - UnitedHealth Group.
Fabiana G. Marcondes Braga	Declaração financeira A - Pagamento de qualquer espécie e desde que economicamente apreciáveis, feitos a (i) você, (ii) ao seu cônjuge/ companheiro ou a qualquer outro membro que resida com você, (iii) a qualquer pessoa jurídica em que qualquer destes seja controlador, sócio, acionista ou participante, de forma direta ou indireta, recebimento por palestras, aulas, atuação como proctor de treinamentos, remunerações, honorários pagos por participações em conselhos consultivos, de investigadores, ou outros comitês, etc. Provenientes da indústria farmacêutica, de órteses, próteses, equipamentos e implantes, brasileiras ou estrangeiras: - Novartis: Palestras; AstraZeneca: Palestras e Conselho Consultivo; Boehringer: Conselho Consultivo.
Fabio Fernandes	Declaração financeira A - Pagamento de qualquer espécie e desde que economicamente apreciáveis, feitos a (i) você, (ii) ao seu cônjuge/ companheiro ou a qualquer outro membro que resida com você, (iii) a qualquer pessoa jurídica em que qualquer destes seja controlador, sócio, acionista ou participante, de forma direta ou indireta, recebimento por palestras, aulas, atuação como proctor de treinamentos, remunerações, honorários pagos por participações em conselhos consultivos, de investigadores,ou outros comitês, etc. Provenientes da indústria farmacêutica, de órteses, próteses, equipamentos e implantes,brasileiras ou estrangeiras: - Pfizer: Tafamidis; Alnylan: Patisiran.
Guilherme H. Oliveira	Nada a ser declarado
Heinz-Peter Schultheiss	Nada a ser declarado
Humberto Villacorta	Declaração financeira A - Pagamento de qualquer espécie e desde que economicamente apreciáveis, feitos a (i) você, (ii) ao seu cônjuge/ companheiro ou a qualquer outro membro que resida com você, (iii) a qualquer pessoa jurídica em que qualquer destes seja controlador, sócio, acionista ou participante, de forma direta ou indireta, recebimento por palestras, aulas, atuação como proctor de treinamentos, remunerações, honorários pagos por participações em conselhos consultivos, de investigadores,ou outros comitês, etc. Provenientes da indústria farmacêutica, de órteses, próteses, equipamentos e implantes,brasileiras ou estrangeiras: - Novartis: Insuficiência Cardíaca; Roche: Biomarcadores; Servier: Insuficiência Cardíaca C - Financiamento de pesquisa (pessoal), cujas receitas tenham sido provenientes da indústria farmacêutica, de órteses, próteses, equipamentos e implantes, brasileiras ou estrangeiras: - Roche: GDF-15.
João Manoel Rossi Neto	Declaração financeira A - Pagamento de qualquer espécie e desde que economicamente apreciáveis, feitos a (i) você, (ii) ao seu cônjuge/ companheiro ou a qualquer outro membro que resida com você, (iii) a qualquer pessoa jurídica em que qualquer destes seja controlador, sócio, acionista ou participante, de forma direta ou indireta, recebimento por palestras, aulas, atuação como proctor de treinamentos, remunerações, honorários pagos por participações em conselhos consultivos, de investigadores,ou outros comitês, etc. Provenientes da indústria farmacêutica, de órteses, próteses, equipamentos e implantes,brasileiras ou estrangeiras: - Novartis: Aulas; AstraZeneca: Aulas.
João Marcos Bemfica Barbosa	Declaração financeira A - Pagamento de qualquer espécie e desde que economicamente apreciáveis, feitos a (i) você, (ii) ao seu cônjuge/ companheiro ou a qualquer outro membro que resida com você, (iii) a qualquer pessoa jurídica em que qualquer destes seja controlador, sócio, acionista ou participante, de forma direta ou indireta, recebimento por palestras, aulas, atuação como proctor de treinamentos, remunerações, honorários pagos por participações em conselhos consultivos, de investigadores,ou outros comitês, etc. Provenientes da indústria farmacêutica, de órteses, próteses, equipamentos e implantes,brasileiras ou estrangeiras: - Novartis: Entresto.
José Albuquerque de Figueiredo Neto	Declaração financeira A - Pagamento de qualquer espécie e desde que economicamente apreciáveis, feitos a (i) você, (ii) ao seu cônjuge/ companheiro ou a qualquer outro membro que resida com você, (iii) a qualquer pessoa jurídica em que qualquer destes seja controlador, sócio, acionista ou participante, de forma direta ou indireta, recebimento por palestras, aulas, atuação como proctor de treinamentos, remunerações, honorários pagos por participações em conselhos consultivos, de investigadores,ou outros comitês, etc. Provenientes da indústria farmacêutica, de órteses, próteses, equipamentos e implantes,brasileiras ou estrangeiras: - Novartis: Insuficiência Cardíaca.
Lídia Ana Zytynski Moura	Declaração financeira A - Pagamento de qualquer espécie e desde que economicamente apreciáveis, feitos a (i) você, (ii) ao seu cônjuge/ companheiro ou a qualquer outro membro que resida com você, (iii) a qualquer pessoa jurídica em que qualquer destes seja controlador, sócio, acionista ou participante, de forma direta ou indireta, recebimento por palestras, aulas, atuação como proctor de treinamentos, remunerações, honorários pagos por participações em conselhos consultivos, de investigadores,ou outros comitês, etc. Provenientes da indústria farmacêutica, de órteses, próteses, equipamentos e implantes,brasileiras ou estrangeiras: - Novartis: Entresto; AstraZeneca: Forxiga. B - financiamento de pesquisas sob sua responsabilidade direta/pessoal (direcionado ao departamento ou instituição) provenientes da indústria farmacêutica, de órteses, próteses, equipamentos e implantes, brasileiras ou estrangeiras: - AstraZeneca: Forxiga.
Louise Freire Luiz	Nada a ser declarado
Ludhmila Abrahão Hajjar	Nada a ser declarado
Luis Beck-da-Silva	Declaração financeira A - Pagamento de qualquer espécie e desde que economicamente apreciáveis, feitos a (i) você, (ii) ao seu cônjuge/ companheiro ou a qualquer outro membro que resida com você, (iii) a qualquer pessoa jurídica em que qualquer destes seja controlador, sócio, acionista ou participante, de forma direta ou indireta, recebimento por palestras, aulas, atuação como proctor de treinamentos, remunerações, honorários pagos por participações em conselhos consultivos, de investigadores,ou outros comitês, etc. Provenientes da indústria farmacêutica, de órteses, próteses, equipamentos e implantes,brasileiras ou estrangeiras: - Novartis: Insuficiência Cardíaca; AstraZeneca: Insuficiência Cardíaca. B - Financiamento de pesquisas sob sua responsabilidade direta/pessoal (direcionado ao departamento ou instituição) provenientes da indústria farmacêutica, de órteses, próteses, equipamentos e implantes, brasileiras ou estrangeiras: - Amgen: Insuficiência Cardíaca.
Luiz Antonio de Almeida Campos	Nada a ser declarado
Luiz Cláudio Danzmann	Declaração financeira A - Pagamento de qualquer espécie e desde que economicamente apreciáveis, feitos a (i) você, (ii) ao seu cônjuge/ companheiro ou a qualquer outro membro que resida com você, (iii) a qualquer pessoa jurídica em que qualquer destes seja controlador, sócio, acionista ou participante, de forma direta ou indireta, recebimento por palestras, aulas, atuação como proctor de treinamentos, remunerações, honorários pagos por participações em conselhos consultivos, de investigadores, ou outros comitês, etc. Provenientes da indústria farmacêutica, de órteses, próteses, equipamentos e implantes,brasileiras ou estrangeiras: - Novartis: Entresto; AstraZeneca: Forxiga; Servier: Procoralan.
Marcelo Imbroise Bittencourt	Declaração financeira A - Pagamento de qualquer espécie e desde que economicamente apreciáveis, feitos a (i) você, (ii) ao seu cônjuge/ companheiro ou a qualquer outro membro que resida com você, (iii) a qualquer pessoa jurídica em que qualquer destes seja controlador, sócio, acionista ou participante, de forma direta ou indireta, recebimento por palestras, aulas, atuação como proctor de treinamentos, remunerações, honorários pagos por participações em conselhos consultivos, de investigadores,ou outros comitês, etc. Provenientes da indústria farmacêutica, de órteses, próteses, equipamentos e implantes,brasileiras ou estrangeiras: - GENEONE - DASA: Testes genéticos; Sanofi: Terapia de reposição enzimática; AstraZeneca: Forxiga.
Marcelo Iorio Garcia	Nada a ser declarado
Marcelo Westerlund Montera	Nada a ser declarado
Marcus Vinícius Simões	Declaração financeira A - Pagamento de qualquer espécie e desde que economicamente apreciáveis, feitos a (i) você, (ii) ao seu cônjuge/ companheiro ou a qualquer outro membro que resida com você, (iii) a qualquer pessoa jurídica em que qualquer destes seja controlador, sócio, acionista ou participante, de forma direta ou indireta, recebimento por palestras, aulas, atuação como proctor de treinamentos, remunerações, honorários pagos por participações em conselhos consultivos, de investigadores,ou outros comitês, etc. Provenientes da indústria farmacêutica, de órteses, próteses, equipamentos e implantes,brasileiras ou estrangeiras: - Novartis: Entresto; AstraZeneca: Dapagliflozina. B - Financiamento de pesquisas sob sua responsabilidade direta/pessoal (direcionado ao departamento ou instituição) provenientes da indústria farmacêutica, de órteses, próteses, equipamentos e implantes, brasileiras ou estrangeiras: - Amgen: Omecamtiv/Mecarbil; Beringher Ingelheim: Empagliflozina.
Monica Samuel Avila	Nada a ser declarado
Nadine Oliveira Clausell	Nada a ser declarado
Nilson Araujo de Oliveira Jr.	Declaração financeira A - Pagamento de qualquer espécie e desde que economicamente apreciáveis, feitos a (i) você, (ii) ao seu cônjuge/ companheiro ou a qualquer outro membro que resida com você, (iii) a qualquer pessoa jurídica em que qualquer destes seja controlador, sócio, acionista ou participante, de forma direta ou indireta, recebimento por palestras, aulas, atuação como proctor de treinamentos, remunerações, honorários pagos por participações em conselhos consultivos, de investigadores, ou outros comitês, etc. Provenientes da indústria farmacêutica, de órteses, próteses, equipamentos e implantes, brasileiras ou estrangeiras: - Johnson e Johnson: Cateteres para eletrofisiologia invasiva. B - Financiamento de pesquisas sob sua responsabilidade direta/pessoal (direcionado ao departamento ou instituição) provenientes da indústria farmacêutica, de órteses, próteses, equipamentos e implantes, brasileiras ou estrangeiras: - Biotronik: Dispositivos de estimulação cardíaca. Outros lacionamentos Financiamento de atividades de educação médica continuada, incluindo viagens, hospedagens e inscrições para congressos e cursos, provenientes da indústria farmacêutica, de órteses, próteses, equipamentos e implantes, brasileiras ou estrangeiras: - Johnson e Johnson: Cateteres para eletrofisiologia.
Odilson Marcos Silvestre	Nada a ser declarado
Olga Ferreira de Souza	Nada a ser declarado
Ricardo Mourilhe-Rocha	Declaração financeira A - Pagamento de qualquer espécie e desde que economicamente apreciáveis, feitos a (i) você, (ii) ao seu cônjuge/ companheiro ou a qualquer outro membro que resida com você, (iii) a qualquer pessoa jurídica em que qualquer destes seja controlador, sócio, acionista ou participante, de forma direta ou indireta, recebimento por palestras, aulas, atuação como proctor de treinamentos, remunerações, honorários pagos por participações em conselhos consultivos, de investigadores, ou outros comitês, etc. Provenientes da indústria farmacêutica, de órteses, próteses, equipamentos e implantes, brasileiras ou estrangeiras: - AstraZeneca: Dapagliflozina; Boehringer: Empagliflozina; Novartis: Sacubitril/Valsartana. B - Financiamento de pesquisas sob sua responsabilidade direta/pessoal (direcionado ao departamento ou instituição) provenientes da indústria farmacêutica, de órteses, próteses, equipamentos e implantes, brasileiras ou estrangeiras: - PROADI/SUS: Telemedicina; Boehriner: Empagliflozina.
Roberto Kalil Filho	Nada a ser declarado
Sadeer G. Al-Kindi	Nada a ser declarado
Salvador Rassi	Declaração financeira A - Pagamento de qualquer espécie e desde que economicamente apreciáveis, feitos a (i) você, (ii) ao seu cônjuge/ companheiro ou a qualquer outro membro que resida com você, (iii) a qualquer pessoa jurídica em que qualquer destes seja controlador, sócio, acionista ou participante, de forma direta ou indireta, recebimento por palestras, aulas, atuação como proctor de treinamentos, remunerações, honorários pagos por participações em conselhos consultivos, de investigadores,ou outros comitês, etc. Provenientes da indústria farmacêutica, de órteses, próteses, equipamentos e implantes,brasileiras ou estrangeiras: - Novartis: Entresto; Servier: Procoralan B - financiamento de pesquisas sob sua responsabilidade direta/pessoal (direcionado ao departamento ou instituição) provenientes da indústria farmacêutica, de órteses, próteses, equipamentos e implantes, brasileiras ou estrangeiras: - Novartis: Entresto; Servier: Procoralan; Boehringer Ingelheim: Jardiance. Outros relacionamentos Financiamento de atividades de educação médica continuada, incluindo viagens, hospedagens e inscrições para congressos e cursos, provenientes da indústria farmacêutica, de órteses, próteses, equipamentos e implantes, brasileiras ou estrangeiras: - Novartis: Entresto; Servier: Procoralan.
Sandrigo Mangine	Declaração financeira A - Pagamento de qualquer espécie e desde que economicamente apreciáveis, feitos a (i) você, (ii) ao seu cônjuge/ companheiro ou a qualquer outro membro que resida com você, (iii) a qualquer pessoa jurídica em que qualquer destes seja controlador, sócio, acionista ou participante, de forma direta ou indireta, recebimento por palestras, aulas, atuação como proctor de treinamentos, remunerações, honorários pagos por participações em conselhos consultivos, de investigadores, ou outros comitês, etc. Provenientes da indústria farmacêutica, de órteses, próteses, equipamentos e implantes, brasileiras ou estrangeiras: - Novartis: Sacubitril/Valsartan; Pfizer: Doenças raras. Outros relacionamentos Financiamento de atividades de educação médica continuada, incluindo viagens, hospedagens e inscrições para congressos e cursos, provenientes da indústria farmacêutica, de órteses, próteses, equipamentos e implantes, brasileiras ou estrangeiras: - Pfizer: Doenças raras.
Silvia Marinho Martins Alves	Nada a ser declarado
Silvia Moreira Ayub Ferreira	Declaração financeira A - Pagamento de qualquer espécie e desde que economicamente apreciáveis, feitos a (i) você, (ii) ao seu cônjuge/ companheiro ou a qualquer outro membro que resida com você, (iii) a qualquer pessoa jurídica em que qualquer destes seja controlador, sócio, acionista ou participante, de forma direta ou indireta, recebimento por palestras, aulas, atuação como proctor de treinamentos, remunerações, honorários pagos por participações em conselhos consultivos, de investigadores, ou outros comitês, etc. Provenientes da indústria farmacêutica, de órteses, próteses, equipamentos e implantes, brasileiras ou estrangeiras: - Abbott: Mitraclip; Novartis: Entresto. Outros relacionamentos Financiamento de atividades de educação médica continuada, incluindo viagens, hospedagens e inscrições para congressos e cursos, provenientes da indústria farmacêutica, de órteses, próteses, equipamentos e implantes, brasileiras ou estrangeiras: - Abbott: Heartmate II e HeartMate 3.
Stéphanie Itala Rizk	Nada a ser declarado
Tiago Azevedo Costa Mattos	Nada a ser declarado
Vitor Barzilai	Nada a ser declarado
Wolney de Andrade Martins	Declaração financeira A - Pagamento de qualquer espécie e desde que economicamente apreciáveis, feitos a (i) você, (ii) ao seu cônjuge/ companheiro ou a qualquer outro membro que resida com você, (iii) a qualquer pessoa jurídica em que qualquer destes seja controlador, sócio, acionista ou participante, de forma direta ou indireta, recebimento por palestras, aulas, atuação como proctor de treinamentos, remunerações, honorários pagos por participações em conselhos consultivos, de investigadores,ou outros comitês, etc. Provenientes da indústria farmacêutica, de órteses, próteses, equipamentos e implantes,brasileiras ou estrangeiras: - Bayer: Cardio-oncologia.

## Sumário

1. Epidemiologia 149

2. Definição e Etiologia 150

2.1. Fator Genético na Etiopatogenia das Miocardites 151

3. Fisiopatogenia 151

4. Avaliação Diagnóstica 152

4.1. Critérios Diagnósticos de Suspeita de Miocardite 152

**4.1.1. Fluxograma de Avaliação Diagnóstica** 152

4.2. Avaliação Clínica: Situações Clínicas de Suspeição 152

4.3. Biomarcadores 154

**4.3.1. Marcadores Laboratoriais de Agressão Inflamatória** 154

**4.3.2. Marcadores Laboratoriais de Pesquisa Etiopatogênica** 155

4.4. Eletrocardiograma 155

**4.4.1. Critério de diagnóstico por eletrocardiograma/ Holter/testes de estresse** 156

**4.4.2. Prognóstico** 157

4.5. Eletrocardiograma 157

4.6. Ressonância Magnética Cardíaca 157

4.7. Medicina Nuclear 158

**4.7.1. Radiotraçadores para Cintilografia por Emissão de Fóton Único (SPECT)** 159

**4.7.2. Radiotraçadores para Tomografia por Emissão de Pósitrons (SPECT)** 160

**4.7.3. Perspectivas Adicionais** 161

4.8. Angiotomografia de Coronárias e Coronariografia 161

4.9. Biópsia Endomiocárdica: Indicações, Técnica e Complicações 162

**4.9.1. Ponderações para Indicação** 162

**4.9.2. Prognóstico** 163

**4.9.3. Técnica** 163

**4.9.4. Complicações** 164

4.10. Análise Histológica e Pesquisa Viral – Biologia Molecular e Genoma 164

**4.10.1. Análise Histológica** 164

**4.10.2. Análise Imuno-histoquímica** 164

**4.10.3. Análise do Perfil Genético** 165

**4.10.4. Virologia** 164

5. Tratamento 164

5.1. Fluxogramas Terapêuticos 164

5.2. Imunossupressão: Indicações e Tipos 164

5.3. Antivirais: Indicações e Tipos 168

5.4. IImunomodulação (Imunoglobulina – Imunoadsorção): Indicações e Tipos de Imunoglobulinas 169

**5.4.1. Imunoadsorção** 169

5.5. Terapêutica Cardioprotetora Convencional 169

**5.5.1. Sem Disfunção Ventricular** 169

**5.5.2. Com Disfunção Ventricular Hemodinâmica Estável** 170

**5.5.3. Paciente com Disfunção Ventricular e Hemodinâmica Instável: Abordagem Terapêutica** 170

5.6. Cuidados Gerais: Atividade Física e Vacinação 170

6. Situações Especiais 171

6.1. Miocardite Fulminante 171

**6.1.1. Avaliação Diagnóstica** 172

**6.1.2. Abordagem Terapêutica** 173

6.2. Sarcoidose 173

**6.2.1. Diagnóstico** 173

**6.2.2. Tratamento e Prognóstico** 175

**6.2.3. Prognóstico** 175

6.3. Células Igantes 176

**6.3.1. Tratamento** 176

**6.3.2. Manifestação Clínica e Diagnóstico** 178

6.4. Miocardite chagásica aguda e reagudização 179

**6.4.1. Manifestações Clínicas e meios de Infecção, Reagudização nos Pacientes Imunossuprimidos** 179

**6.4.2. Diagnóstico** 179

**6.4.3. Tratamento** 179

6.5. Miocardite por Doenças Tropicais 180

6.6. Miocardite por Covid-19 181

**6.6.1. Possível Fisiopatologia da Miocardite Relacionada ao SARS-CoV-2** 181

**6.6.2. Lesão Miocárdica Viral Direta** 181

**6.6.3. Diagnóstico de Miocardite Relacionada à Covid-19** 182

**6.6.4. Laboratório** 183

**6.6.5. Eletrocardiograma** 183

**6.6.6. Imagem** 184

**6.6.7. Biópsia Endomiocárdica** 184

6.7. Cardiotoxidade Aguda por Terapêutica Antineoplásica 185

**6.7.1. Agentes Antineoplásicos Indutores de Cardiotoxidade Aguda** 185

**6.7.2. Diagnóstico da Cardiotoxidade Aguda** 186

**6.7.3. Tratamento da Cardiotoxidade Aguda** 186

**6.7.4. Prognóstico** 188

**6.7.5. Prevenção** 188

6.8. Miocardite em Crianças e Adolescentes 189

**6.8.1. Fatores Causais** 189

**6.8.2. Prognóstico** 190

6.9. Miocardites com Envolvimento Pericárdico 192

**6.9.1. Diagnóstico e Tratamento** 192

6.10. Miocardite Simulando Infarto Agudo do Miocárdio 192

7. Cardite Reumática 193

8. Miocardites por Doenças Autoimunes 195

9. Manejo das Arritmias Cardíacas na Miocardite 196

9.1. Avaliação Não Invasiva e Invasiva das Arritmias na Fase Aguda e Crônica das Diversas Causa das Miocardites 196

9.2. Tratamento de Arritmias e Prevenção da Morte Súbita na Fase Aguda e Subaguda 197

10. Avaliação Prognóstica e Seguimento 198

10.1. Marcadores de Prognóstico e Evolução 198

10.2 Seguimento Ambulatorial nas Avaliações dos Métodos Complementares 198

Referências 199

## 1. Epidemiologia

A real incidência de miocardite é difícil de ser determinada, uma vez que as apresentações clínicas são muito heterogêneas e grande parcela dos casos cursa de forma subclínica, além de haver uma frequência muito baixa de emprego da biópsia endomiocárdica (BEM), o padrão-ouro para o diagnóstico.^1^

Levantamento de diferentes séries de estudos necroscópicos em indivíduos jovens vítimas de morte súbita inexplicada mostrou incidência muito variável de miocardite, podendo corresponder por até 42% dos casos.^2^ O Global Burden of Disease Study 2013 usou os códigos da Classificação Internacional de Doenças em análises estatísticas regionais e globais de 187 países, estimando a incidência anual de miocardite em torno de 22 casos para cada 100.000 pacientes atendidos.^3^ Em coortes de pacientes com apresentação clínica de miocardiopatia dilatada de etiologia não definida, miocardite comprovada por BEM pode ser detectada em até 16% dos pacientes adultos,^4^ e em até 46% de pacientes pediátricos.^5^

Muitos estudos indicam maior prevalência da miocardite aguda em homens do que em mulheres^6 , 7^ Alguns estudos sugerem que, em adultos, a manifestação clínica mais comum seja a miocardite linfocítica, com mediana de idade de 42 anos, enquanto pacientes com miocardite de células gigantes têm mediana de 43 anos de idade.^8^ Entretanto, recém-nascidos e crianças exibem mais tipicamente apresentação de miocardite fulminante e são mais suscetíveis à patogenicidade induzida por vírus do que adultos.^9 , 10^

A miocardite engloba um amplo espectro de prognósticos, dependendo da gravidade do quadro clínico inicial e da sua etiologia. Pacientes com sintomas leves e sem disfunção ventricular exibem com grande frequência resolução espontânea e excelente prognóstico.^11^ No entanto, estima-se que cerca de 30% dos casos de miocardite mais graves, documentados com BEM e cursando com disfunção ventricular evoluam para miocardiopatia dilatada e insuficiência cardíaca (IC) com prognóstico reservado. Em pacientes pediátricos, o prognóstico parece ser pior, com relatos de sobrevida em 10 anos livre de transplante cardíaco de apenas 60%.^5^

## 2. Definição e Etiologia

A miocardite pode ser definida como doença inflamatória do miocárdio, diagnosticada por critérios histológicos, imunológicos e imuno-histoquímicos. Os critérios histológicos incluem evidência de infiltrado inflamatório envolvendo o miocárdio associado com degeneração e necrose de cardiomiócitos e de origem não isquêmica. Os critérios imuno-histoquímicos quantitativos para identificar um infiltrado inflamatório anormal, indicativos de miocardite ativa, são: contagem de leucócitos ≥14 células/mm^2^, incluindo até 4 monócitos/mm^2^, com a presença de linfócitos-T CD3 positivos ≥7 células/mm^2^.^12^

Adicionalmente, conforme o tipo celular, o tipo de infiltrado inflamatório observado no diagnóstico histológico pode classificar a miocardite em linfocítica, eosinofílica, polimórfica, miocardite de células gigantes e sarcoidose cardíaca.^13^

Miocardite pode ser causada por uma grande variedade de agentes infecciosos, incluindo vírus, protozoários, bactérias, clamídias, rickéttsias, fungos e espiroquetas ( Tabela 1 ), bem como pode ser desencadeada por mecanismos não infecciosos como a miocardite tóxica (drogas, metais pesados, radiação), miocardite por mecanismos autoimunes e de hipersensibilidade (miocardite eosinofílica, colagenoses, induzida por vírus, rejeição do coração transplantado).^14 , 15^

**Tabela 1:** – Etiologia da miocardite aguda*

1 – Miocardite infecciosa
Viral
Vírus RNA	Vírus Coxsackie A e B, echo-vírus, poliovírus, vírus da influenza A e B, vírus sincicial respiratório, vírus da caxumba, vírus do sarampo, vírus da rubéola, vírus da hepatite C, vírus da dengue, vírus da febre amarela, vírus da Chikungunya, vírus Junin, vírus da febre de Lassa, *Rabies virus* , vírus da imunodeficiência humana-1
Vírus DNA	Adenovírus, parvovírus B19, citomegalovírus, herpes-vírus humano-6, vírus Epstein-Barr, vírus varicela-zóster, herpes-vírus simples, vírus da varíola, vírus vaccinia
Bactérias	*Staphylococcus, Streptococcus, Pneumococcus, Meningococcus, Gonococcus, Salmonella, Corynebacterium diphtheriae, Haemophilus influenzae, Mycobacterium * (tuberculose) *, Mycoplasma pneumoniae, Brucella*
Espiroquetas	*Borrelia* (doença de Lyme), *Leptospira* (doença de Weil)
Fungos	*Aspergillus, Actinomyces, Blastomyces, Candida, Coccidioides, Cryptococcus, Histoplasma, Mucormycoses, Nocardia, Sporothrix*
Protozoários	*Trypanosoma cruzi, Toxoplasma gondii, Entamoeba, Leishmania*
Parasitas	*Trichinella spiralis, Echinococcus granulosus, Taenia solium*
Rickéttsias	*Coxiella burnetii * (Febre Q), *R. Rickettsii * (febre maculosa das Montanhas Rochosas), *R. tsutsugamushi*
**2 – Miocardite imunomediada**
Alérgenos	Toxoide tetânico, vacinas, doença do soro Drogas: penicilina, cefaclor, conchicina, furosemida, isoniazida, lidocaína, tetraciclina, sulfonamidas, fenitoína, fenilbutazona, metildopa, diuréticos tiazídicos, amitriptlina
Aloantígenos	Rejeição do coração transplantado
Autoantígenos	Miocardite linfocítica infecção-negativa, miocardite de células gigantes infecção-negativa associadas a distúrbios autoimunes: lúpus eritematoso sistêmico, artrite reumatoide, síndrome de Churg-Strauss, doença de Kawasaki, doença inflamatória intestinal, esclerodermia, polimiosite, miastenia grave, diabetes melito dependente de insulina, sarcoidose, granulomatose de Wegener, febre reumática, imunoterapia oncológica (inibidores de check-point imunológico)
**3 – Miocardite tóxica**
Drogas	Anfetaminas, antraciclinas, cocaína, ciclofosfamida, etanol, fluouracil, lítio, catecolaminas, hemetina, trastuzumab, clozapina, interleucina-2, inibidores de check-point imunológico
Metais pesados	Cobre, ferro, chumbo
Miscelânea	Picada de escorpião, picada de cobra, picada de aranha, picada de abelha e vespa, monóxido de carbono, inalantes, fósforo, arsênico, azida de sódio
Hormônios	Feocromocitoma
Agentes físicos	Radiação, choque elétrico

Fonte: *Adaptada de Caforio et al.^5^

Dentre todos esses desencadeantes de miocardite, a infecção viral é a mais prevalente, particularmente em crianças. Os vírus cardiotrópicos mais prevalentes são: enterovírus, parvovírus B19 (PVB19), adenovírus, vírus influenza A, herpes-vírus humano (HHV), vírus Epstein-Barr, citomegalovírus, vírus da hepatite C e vírus do HIV. Algumas evidências sugerem que possa haver diferenças regionais em relação à prevalência dos diferentes agentes virais, com predomínio de adenovírus, parvovírus e herpes na população europeia^16^ e preponderância de enterovírus na população americana.^17^ Entretanto, parte dessas diferenças epidemiológicas pode ser decorrente de surtos de infecções virais específicas ocorrendo ao longo dos anos nas diversas regiões do mundo, bem como a diferenças nas técnicas de detecção viral, permanecendo o debate acerca da real influência da distribuição geográfica quanto às infecções virais cardiotrópicas.^18^

Na América do Sul e, especialmente, em algumas regiões do Brasil, a miocardite chagásica causada pelo protozoário *Trypanosoma cruzi* é uma das causas mais prevalentes de miocardite aguda, particularmente frente ao registro recente de surtos de casos associados à transmissão oral na Amazônia brasileira.^19^ Doenças sistêmicas autoimunes como a síndrome de Churg-Strauss e a síndrome hipereosinofílica estão associadas à miocardite eosinofílica. A miocardite de células gigantes e a sarcoidose, embora raras, revestem-se de especial importância clínica, uma vez que, se diagnosticadas precocemente, têm tratamento específico, o que pode garantir melhora do prognóstico.^20 , 21^

Miocardite autoimune pode ocorrer como acometimento orgânico isolado ou manifestar-se no contexto de doenças autoimunes com manifestações sistêmicas, sendo as mais frequentes: sarcoidose, síndrome hipereosinofílica, esclerodermia e lúpus eritematoso sistêmico.

Novos imunoterápicos para tratamento do câncer podem estar associados ao risco de miocardite, sendo mais recentemente reconhecidos os casos vinculados ao uso dos inibidores de *checkpoint* imunológico, como nivolumab e ipilimumab.^22 - 24^

### 2.1. Fator Genético na Etiopatogenia das Miocardites

Nas descrições clássicas da etiopatogenia da miocardite, as evidências de mecanismos envolvendo a atuação de vírus e reações autoimunes são bem documentadas. Pouco se fala a respeito da predisposição genética. Muitos autores acreditam que é provável que fenômenos genéticos possam contribuir para o desenvolvimento de miocardite viral e/ou autoimune.^12 , 25^

Dados laboratoriais consistentes com este argumento foram documentados em um estudo com 342 familiares de pacientes com cardiomiopatia dilatada (CMPD), em que se constatou a presença de anticorpos cardíacos com intensidade maior do que foi observado no grupo controle.^26^

Além disso, também é largamente reconhecida a probabilidade de uma interação complexa entre causas genéticas (predisposição individual) e não genéticas (ligadas ao agente agressor) na evolução final para cardiomiopatia dilatada. O problema é que as evidências científicas que suportam tal argumento são escassas.^27^

Há evidências de que, em cepas de camundongos suscetíveis, a infecção e a inflamação desencadeiam reações autoimunes no coração, geralmente como resultado da necrose dos miócitos e subsequente liberação de autoantígenos anteriormente ocultos no sistema imunológico. As mesmas linhagens de animais geneticamente predispostas desenvolvem miocardite linfocítica ou de célula gigante autoimune e depois cardiomiopatia dilatada após imunização com autoantígenos cardíacos (p. ex., miosina cardíaca).^28^

Além disso, há a evidência de que a miocardite pode estar presente em cardiomiopatias específicas (p. ex., cardiomiopatia arritmogênica), levando a alterações no fenótipo e progressão abrupta da doença. Nesse contexto, algumas mutações podem aumentar a suscetibilidade à miocardite.^29^

Entretanto, no geral, a miocardite segue classificada como um distúrbio adquirido não familiar, com evidências de estudos experimentais que indicam que alterações genéticas possam proporcionar suscetibilidade maior a esta doença.

## 3. Fisiopatogenia

De forma simplificada, podemos dividir a fisiopatologia das miocardites em infecciosas e não infecciosas. As infecciosas são as mais comuns e incluem uma enorme gama de vírus, bactérias, protozoários, fungos e outros patógenos mais raros (ver Tabela 1 ). Os agentes virais são os mais comumente envolvidos e estudados experimentalmente. Do ponto de vista não infeccioso, a autoimunidade está presente, mediante doenças específicas, drogas e autoanticorpos; a predisposição genética exerce papel importante em ambas (ver Tabela 1 ).

Modelos murinos de miocardite viral sugerem que seu desenvolvimento apresenta três fases: aguda (alguns dias), subaguda (algumas semanas a meses) e crônica (desenvolvimento da miocardiopatia dilatada);^30^ além disso, dois mecanismos patogênicos são descritos: lesão citopática direta induzida pelos microrganismos e resposta imune anticardíaca induzida pelos microrganismos.

A fase 1 corresponde à infecção inicial, que pode curar até mesmo sem sequela ou levar à IC ou morte, ou progredir para as fases 2/3.^31^ Na maioria dos pacientes com miocardite viral, o patógeno é eliminado e o sistema imune reduz sua atividade sem outras complicações adicionais. Entretanto, em uma minoria de pacientes, o vírus não é eliminado, resultando em lesão miocárdica persistente e inflamação secundária à produção de anticorpos.^17^ Assim, a miocardite viral poderia ser considerada um dos precursores para o desenvolvimento da miocardiopatia dilatada, sendo observada essa evolução em 21% dos casos de miocardite ao final de 3 anos.^32^

Os enterovírus, em especial o Coxsackie B3 (CVB3), iniciam a miocardite por meio do acoplamento ao receptor CAR (Coxsackie vírus e adenovírus receptor) e DAF (do inglês, *decay accelarating fator* ), culminado em morte celular através de apoptose^33^ ou necroptose.^34^ Cardiomiócitos infectados tornam-se lisados, o que resulta em liberação citosólica de proteínas e produtos virais. Após a fase aguda, o curso da doença depende da base genética, podendo evoluir para miocardiopatia dilatada ou haver resolução.^35 - 39^ Infecção por Coxsackie ativa respostas imunes inatas e adaptativas, incluindo, em primeiro momento, a produção de interferon e ativação de receptores *toll-like* (TLR).^40^ Na resposta adaptativa, a deficiência de células T e B leva à persistência viral e piora evolutiva.^41 , 42^

Outro aspecto importante é a produção de autoanticorpos específicos contra os cardiomiócitos, que ocorre por meio da liberação de peptídios cardíacos, havendo mimetismo molecular entre as proteínas cardíacas e os agentes virais. Na presença de citocinas coestimuladoras como TNF e IL1, esses anticorpos promovem resposta efetora de linfócitos T.^43^

Outros vírus como o parvovírus B19 e o herpes-vírus-6 têm sido cada vez mais descritos em biópsias cardíacas, havendo uma tendência de redução de identificação de enterovírus e adenovírus.^44^ No entanto, a presença desses microrganismos também tem sido observada em corações sem miocardite ou miocardiopatias de outras etiologias, tornando complexa a interpretação da associação entre a presença de agentes infecciosos no tecido cardíaco e o desenvolvimento de miocardite, bem como a influência da persistência desses agentes na evolução clínica.^45^

Em relação às miocardites não infecciosas, modelos animais de miocardite autoimune envolvem linhagens suscetíveis geneticamente que demonstram a presença de linfócitos T CD4+ reativos a autoantígenos, como a cadeia pesada da miosina, na ausência de agentes infecciosos.^46^ Além da resposta autoimune linfocitária, podemos observar respostas envolvendo macrófagos, como nas miocardites granulomatosas e eosinófilos nas situações de hipersensibilidade.

A miocardite de células gigantes é uma forma autoimune de agressão miocárdica e caracteriza-se histologicamente por um infiltrado de células gigantes multinucleadas, além de infiltrado inflamatório de células T, eosinófilos e histiócitos. A presença marcante de células CD8 (citotóxicas), liberação de citocinas inflamatórias e mediadores do estresse oxidativo leva a uma intensa agressão às células miocíticas e reposição por fibrose, culminando em rápida perda da função ventricular e evolução clínica desfavorável. Em 20% dos casos, existe associação com doenças autoimunes como tireoidite de Hashimoto, artrite reumatoide, miastenia grave, arterite de Takayasu, entre outras.^47^ A sarcoidose tem caráter multissistêmico, envolvendo o pulmão em 90% dos casos, associada a acúmulo de linfócitos T, fagócitos mononucleares e granulomas não caseosos nos tecidos envolvidos.^48 , 49^

Na miocardite induzida por drogas, a resposta de sensibilidade pode variar de horas a meses. Parte da justificativa da hipersensibilidade se dá em resposta a componentes quimicamente reativos que se ligam a proteínas promovendo modificações estruturais. Essas partículas são fagocitadas pelas células de defesa, por vezes macrófagos, os quais as apresentam na superfície dessas células aos linfócitos T. Como uma resposta de hipersensibilidade retardada, são liberadas citocinas como interleucina 5, estimulante de eosinófilos. Esse acúmulo de interleucina 5 promove um grande infiltrado eosinofílico com aumento da resposta de hipersensibilidade e maior lesão miocárdica. A predisposição genética parece favorecer esse padrão de resposta.^50^

A síndrome hipereosinofílica pode ocorrer em associação a diversas doenças com manifestação sistêmica, como síndrome de Churg-Strauss, câncer, infecções parasitárias e helmínticas, ou estar relacionada a vacinações. Estas podem promover uma resposta inflamatória intensa no miocárdio, levando à lesão celular com disfunção e IC.^51 , 52^ Do ponto de vista fisiopatológico, assim como em outros órgãos, ocorre um intenso infiltrado eosinofílico no miocárdio, infiltrado este que promove a liberação de mediadores altamente agressivos ao miócito, levando a necrose e perda da estrutura miocárdica. Entre os fatores agressores, estão a neurotoxina, derivada dos eosinófilos, a proteína catiônica do eosinófilo e a protease eosinofílica. Além desses fatores, a produção de citocinas inflamatórias como IL 1, TNF-alfa, IL 6, IL 8, IL3, IL5 e proteínas inflamatórias do macrófago promove a lesão e perda de miócitos, com evolução para disfunção miocárdica.^53^

Mais recentemente, o nivolumab, droga antitumoral que atua como inibidor de *checkpoint* , tem sido considerado como causa de miocardite linfocitária. Possível mecanismo fisiopatológico sugere que as células miocárdicas poderiam compartilhar antígenos com as células tumorais, sendo, consequentemente, alvos de células T ativadas, resultando em infiltrado inflamatório e desenvolvimento de IC e distúrbios de condução.^54^

## 4. Avaliação Diagnóstica

### 4.1. Critérios Diagnósticos de Suspeita de Miocardite

A suspeita clínica do diagnóstico de miocardite pelo consenso do grupo de doenças do miocárdio e pericárdio da sociedade europeia de cardiologia baseia-se na associação da apresentação clínica com exames complementares alterados sugestivos de lesão inflamatória miocárdica.^12 , 55^

**Figura 1:**
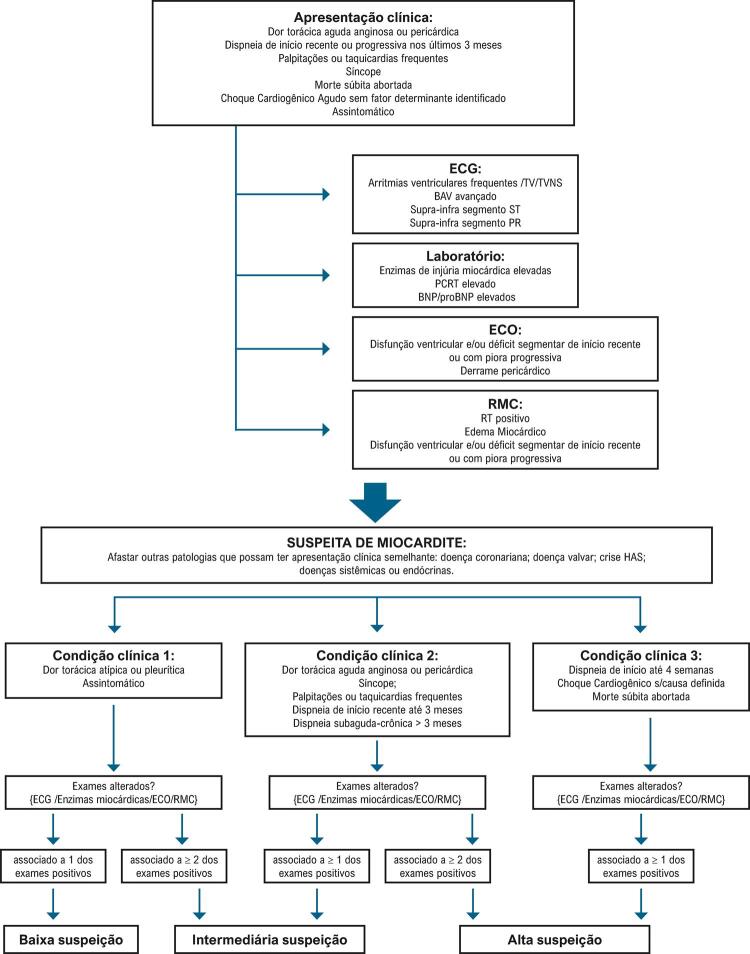
– Algoritmo de estratificação de suspeita clínica diagnóstica de miocardite.

BAV: bloqueio atrioventricular; BNP: peptídeo natriurético B; BNP: peptídeo natriurético tipo B; ECG: eletrocardiograma; ECO: ecocardiograma transtorácico; HAS: hipertensão arterial; PCRT: proteína C reativa titulada; PR: segmento PR; RMC: ressonância magnética cardíaca; RT: realce tardio; ST: segmento ST; TV: taquicardia ventricular; TVNS: taquicardia ventricular não sustentada.

Por meio de análise das apresentações clínicas mais frequentes da miocardite e na acurácia diagnóstica dos métodos de avaliação complementar em prognosticar a presença de agressão inflamatória miocárdica, propõe-se estratificar a suspeita clínica diagnóstica de miocardite em três níveis: baixa, intermediária e alta suspeição diagnóstica ( Figura 1 ).^32 , 56 - 63^ Esses critérios de suspeição foram estabelecidos por consenso de especialistas, e necessitam de validação futura por registros clínicos ou estudos multicêntricos.

#### 4.1.1. Fluxograma de Avaliação Diagnóstica

O fluxograma de avaliação diagnóstica da miocardite se baseia no grau de suspeita clínica e prognóstica do paciente (ver Figura 1 ). Os pacientes com baixa suspeita clínica apresentam um prognóstico favorável, sendo seguidos em acompanhamento clínico e avaliados quanto à necessidade de extratificação não invasiva de doença arterial coronariana (DAC). Os pacientes com suspeita intermediária com evolução clínica favorável têm a mesma linha de seguimento clínico e de investigação diagnóstica que os pacientes de baixo risco. Os pacientes que evoluem com manutenção ou piora clínica, função ventricular, arritmias ou bloqueio AV devem ser submetidos a coronariografia (CAT) e BEM. Os pacientes com alta suspeita diagnóstica, em geral, apresentam um pior prognóstico, devem ser submetidos a CAT e BEM, para definição etiológica, com objetivo de definir um tratamento específico para melhora do prognóstico.^32 , 56 , 64 , 65^

## 4.2. Avaliação Clínica: Situações Clínicas de Suspeição

A miocardite pode se manifestar de diferentes formas, variando desde quadro leve e oligossintomático até quadro grave, associado a arritmias ventriculares, instabilidade hemodinâmica e choque cardiogênico. Raramente, pode se apresentar como morte súbita (variando de 8,6% a 12%), principalmente na infância ou em adultos jovens.^66 , 67^

O quadro mais comum ocorre em pacientes jovens com dor torácica sugestiva de infarto agudo do miocárdio (IAM) com coronárias normais após infecção viral respiratória ou intestinal, embora os sintomas virais nem sempre precedam os quadros de miocardite (pode variar de 10% a 80% dos casos).^68 - 70^ Apesar de ocorrer predominantemente em pacientes jovens, a síndrome pode surgir em qualquer idade. Pode ocorrer também quadro de miocardite subclínica, elevação transitória de troponina ou alterações eletrocardiográficas após quadro viral agudo, o qual se manifesta com sintomas inespecíficos como febre, mialgia, sintomas respiratórios ou gastrointestinais.^68 , 71^

Há diferentes formas de apresentação dessa doença:^12 , 71 , 72^

- Quadro clínico semelhante à síndrome coronariana aguda (dor torácica, alterações eletrocardiográficas sugestiva de isquemia; elevação de marcadores de necrose miocárdica com coronárias normais).
- Sintomas novos agudos de IC (entre 3 dias e 3 meses) na ausência de doença coronariana ou causa conhecida para os sintomas.
- Sintomas de IC de início recente nos últimos meses (> 3 meses) na ausência de doença coronariana ou causa conhecida para os sintomas.
- Condições ameaçadoras da vida: arritmias ventriculares inexplicadas e/ou síncope e/ou morte súbita abortada; choque cardiogênico sem doença coronariana associada.

## A) Manifestação como dor torácica

Pacientes que se apresentam com dor torácica podem ter alterações eletrocardiográficas variáveis: supra ou infradesnivelamento do segmento ST; inversão de onda T ou ondas Q patológicas. Alterações segmentares ao ecodopplercardiograma e elevação de marcadores de necrose miocárdica, especialmente troponina, em pacientes com coronárias normais sugerem a hipótese de miocardite.^68 , 73^ Na maioria dos estudos, esses pacientes têm bom prognóstico em curto prazo, sendo o grau de comprometimento ventricular preditor de risco de morte.^71 , 74^ Uma minoria desenvolve miopericardite persistente e recorrente com função de ventrículo esquerdo (VE) normal que podem responder à colchicina.^75^

## B) Manifestação como insuficiência cardíaca aguda

A forma de apresentação pode ser aguda, associada ao aparecimento dos sintomas de IC em dias, mas também subagudo/crônico, cardiomiopatia de início recente em paciente sem causa aparente para a alteração de função miocárdica.

A apresentação da miocardite por sintomas de IC (dispneia, fadiga, intolerância ao exercício) pode ocorrer associada a leve comprometimento da função ventricular (fração de ejeção de ventrículo esquerdo [FEVE] entre 40% e 50%) que melhora em semanas a meses. Contudo, número menor de pacientes pode apresentar disfunção ventricular importante (FEVE <35%) e, destes, 50% desenvolvem disfunção de VE crônica; cerca de 25% necessitarão de transplante cardíaco ou dispositivo de assistência ventricular, enquanto os outros 25% terão melhora da função ventricular ao longo do seguimento; uma minoria dos casos pode evoluir com quadro de choque cardiogênico e necessidade de suporte circulatório mecânico.^68 , 76 - 79^ O risco de morte ou necessidade de transplante está fortemente associado ao grau de comprometimento hemodinâmico e da função ventricular esquerda e direita, que podem responder ao tratamento medicamentoso padrão para IC.^80^

A apresentação da doença na forma fulminante caracteriza-se pelo aparecimento abrupto (dias) dos sintomas de IC avançada. Esses pacientes, em geral, têm grave disfunção ventricular com pouca alteração dos diâmetros ventriculares. Trata-se de apresentação dramática que necessita de intervenção precoce.^68 , 81^Quando o quadro fulminante está associado à taquicardia ventricular persistente ou não há resposta à terapêutica padrão, o prognóstico é pior, e formas mais graves de miocardite, como miocardite de células gigantes, devem ser consideradas e investigadas.^8^

## C) Manifestação como insuficiência cardíaca crônica ou progressiva

Miocardite confirmada por critério imuno-hitopatológico está presente em até 40% dos pacientes com cardiomiopatia crônica que persistem sintomáticos a despeito do tratamento medicamentoso. A presença de inflamação acessada por histologia está associada a pior prognóstico.^71^

## D) Manifestação como condição ameaçadora da vida

- Arritmias ou distúrbios de condução

Pacientes com miocardite podem, ainda, apresentar distúrbios do sistema de condução, tais como bloqueio atrioventricular (BAV) de 2° ou 3° grau ou total, principalmente aqueles que apresentam sinais ecocardiográficos de hipertrofia por edema intersticial.^82^ A presença de bloqueio cardíaco ou arritmias ventriculares sintomáticas ou sustentadas em pacientes com cardiomiopatia deve levantar a suspeita de miocardite com causa definida (doença de Lyme; sarcoidose; displasia arritmogênica de ventrículo direito ou Chagas em áreas endêmicas).^71^

- Choque cardiogênico

Subgrupo pequeno de pacientes que se apresentam com quadro súbito de IC dentro de 2 semanas de quadro viral pode precisar de suporte inotrópico e/ou suporte circulatório mecânico. Em geral, ocorre recuperação da função ventricular quando sobrevivem ao quadro inicial, porém necessitam de instituição da terapêutica adequada o mais precoce possível.^71 , 81^

A Tabela 2 resume as principais síndromes clínicas de suspeição de miocardite e sugere possíveis agentes responsáveis por cada forma de apresentação da doença.^83^

**Tabela 2:** – Descrição do quadro clínico e possível causa das diferentes síndromes clínicas da miocardite

Síndrome clínica	Quadro clínico	Possível causa
Dor torácica aguda	Sintomas de angina; DAC afastada; alteração de segmento ST/T; arritmias; elevação de troponina I/T e NtproBNP intermitentes	Parvovírus B19 ou outros vírus com tropismo pelo coração associado ou não à pericardite
IC aguda	Dispneia; edema; disfunção sistólica e/ou diastólica de VE; alteração de ECG; elevação troponina I/T e NTproBNP intermitentes	Miocardite viral ou não viral ou cardiomiopatia inflamatória
IC crônica	Todos os sintomas de IC por algum tempo; DAC afastada; alterações do ECG como BRE, BRD, BAV; elevação troponina I/T e NtproBNP intermitentes	Miocardite focal viral ou não viral ou cardiomiopatia inflamatória
IC/arritmia ameaçadoras da vida	Choque cardiogênico; IC NYHA III/IV; troponina e NTproBNP elevados; arritmia grave; DAC afastada	Miocardite de células gigantes; miocardite eosinofílica; miocardite tóxica

*BAV: bloqueio atrioventricular; BRD: bloqueio de ramo direito; BRE: bloqueio de ramo esquerdo; DAC: doença arterial coronariana; ECG: eletrocardiograma; IC: insuficiência cardíaca; ST/T: segmento ST e onda T; VE: ventrículo esquerdo.*

## 4.3. Biomarcadores

### 4.3.1. Marcadores Laboratoriais de Agressão Inflamatória

Nenhum biomarcador, isoladamente, é suficiente para diagnosticar miocardite; contudo, alguns biomarcadores podem ser úteis como marcadores prognósticos. A seguir, comentaremos acerca dos principais biomarcadores usados nessa avaliação.

- **Marcadores inflamatórios.** Contagem de leucócitos, velocidade de hemossedimentação (VHS) e proteína C reativa (PC-R) podem estar elevadas em pacientes com miocardite. No entanto, não apresentam valor diagnóstico, pois são inespecíficos.
- **Troponinas.** As troponinas são mais específicas que CPK e CKMB para lesões miocárdicas e estão frequentemente elevadas em pacientes com miocardite. ^84^ No entanto, troponinas normais não excluem o diagnóstico. Embora não sejam suficientes para estabelecer o diagnóstico de miocardite, podem sugerir o diagnóstico, desde que excluídas causas óbvias como IAM e IC aguda. Em um estudo pequeno, em que vários biomarcadores foram avaliados, troponinas foram preditores do diagnóstico de miocardite confirmada por biópsia, com área sob a curva de 0,87, sensibilidade de 83% e especificidade de 80%. ^85^ Troponina é útil para o diagnóstico de miocardite em pacientes com miocardiopatia de instalação aguda.^12 , 72^
- **Peptídios natriuréticos.** BNP e NT-proBNP podem estar elevados na miocardite. ^86^ No entanto, não são úteis para confirmação diagnóstica, uma vez que se elevam frente a diferentes causas de IC. Contudo, podem ser marcadores prognósticos. Em um estudo com miocardite confirmada por biópsia, entre vários biomarcadores avaliados, somente o NT-proBNP acima do último quartil (>4.245pg/mL) foi preditor de morte ou transplante cardíaco. ^85^

### 4.3.2. Marcadores Laboratoriais de Pesquisa Etiopatogênica

**Sorologias virais.** São de valor limitado no diagnóstico de miocardite, uma vez que anticorpos IgG de vírus cardiotrópicos são muito prevalentes na população geral na ausência de doença cardíaca viral. Em um estudo, não se observou correlação entre sorologia viral e achados da biópsia.^87^ Em situações específicas, podem ser úteis a sorologia para hepatite C, pesquisa de vírus HIV em indivíduos de alto risco e doença de Lyme em áreas endêmicas. A pesquisa de marcadores sorológicas deve ser ditada pela elevada suspeição clínica para aquela doença ( Tabela 3 ).

**Tabela 3:** – Recomendações na avaliação laboratorial inicial da miocardite

Indicações	Classe	Nível de evidência
Uso de marcadores inflamatórios para o diagnóstico de miocardite	I	C
Biomarcadores de lesão miocárdica para auxiliar o diagnóstico de miocardite	I	B
BNP ou NT-ProBNP para auxiliar o diagnóstico e estratificação de prognóstico de miocardite	I	B
Investigação sorológica e/ou detecção de antígenos e/ou RT-PCR para diagnóstico de Covid-19 em casos suspeitos	I	B
Investigação sorológica e/ou detecção de antígenos e/ou RT-PCR para avaliação inicial de pacientes em situações especiais de suspeita de miocardite por etiologias específicas	IIa	C
Sorologias virais na investigação de rotina de todos os casos de miocardite	III	C

*BNP: peptídeo natriurético tipo B; RT-PCR: reação em cadeia de polimerase.*

**Marcadores imuno-histoquímicos e análise de genoma viral.** São superiores aos critérios de Dallas e, portanto, úteis no diagnóstico etiológico. A taxa de complicações com a BEM é baixa ( Tabela 3 ).^88 - 90^

## 4.4. Eletrocardiograma

**Figura 2:**
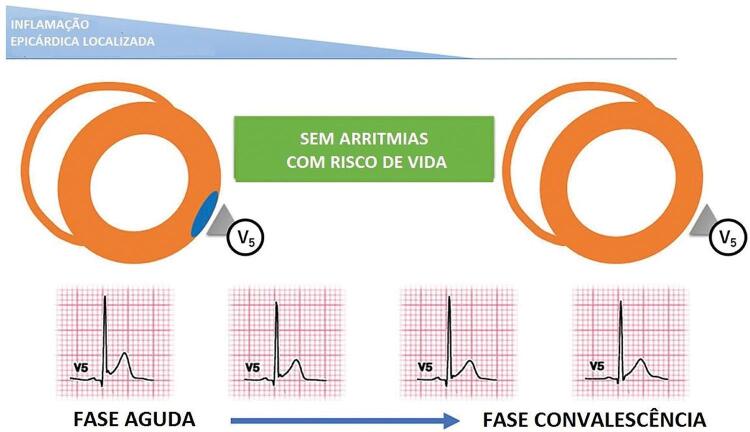
- Curso evolutivo do padrão de repolarização precoce na miocardite aguda.

O eletrocardiograma (ECG) é comumente solicitado para triagem de miocardite, mas com especificidade limitada, embora os pacientes frequentemente apresentem alguma alteração no ECG.^12^ Taquicardia sinusal pode ser a forma mais comum de apresentação do ECG.^14^ Algumas alterações no ECG são mais sugestivas de miocardite do que outras. Por exemplo, a elevação do segmento ST-T na miocardite é tipicamente côncava (em vez de convexa na isquemia miocárdica), difusa sem alterações recíprocas, transitória e reversível na evolução ( Figura 2 ).^91^

Adaptada de Oka et al.^91^

O padrão de repolarização precoce (RP) no ECG de alguns pacientes com miocardite aguda pode ser a evidência de inflamação/edema localizado no epicárdico do VE. Oka et al.^91^ mostraram que o padrão de RP no ECG da miocardite aguda foi transitório, reversível e não estava associado a um pior prognóstico.^91^

O BAV, na presença de dilatação leve do VE, pode ser devido a várias causas (incluindo laminopatia), mas também pode ser sugestivo de doença de Lyme, sarcoidose cardíaca ou miocardite de células gigantes. Ogunbayo identificou que, em 31.760 pacientes com diagnóstico primário de miocardite, o bloqueio cardíaco foi relatado em 540 (1,7%), sendo 21,6% com BAV de primeiro grau, 11,2% com BAV de segundo grau e 67,2% com BAV de alto grau. O BAV de alto grau apresentou associação independente com o aumento da morbimortalidade.^92^

Recente metanálise mostrou que o alargamento de QRS esteve presente como característica precoce da miocardite fulminante.^93^ Em um estudo em que pacientes internados agudamente com miocardite sem IC prévia foram submetidos à BEM, o alargamento do QRS foi preditor independente de morte cardíaca ou transplante cardíaco.^94^

Uma proporção significativa de pacientes com miocardite aguda apresenta morte súbita cardíaca, presumivelmente por arritmia cardíaca. Estudo recente de Adegbala mostrou um total de 32.107 internações por miocardite aguda entre 2007 e 2014, nos EUA, das quais 10.844 (33,71%) apresentaram arritmias, sendo as mais comuns taquicardia ventricular (22,3%) e fibrilação atrial (26,9%), e a presença dessas arritmias teve impacto na mortalidade.^95^

Resumidamente, o ECG fornece uma ferramenta conveniente para a estratificação de risco e a triagem inicial, mas com valor diagnóstico fraco.^14^

### 4.4.1. Critério de diagnóstico por eletrocardiograma/ *Holter* /testes de estresse ^12^

O ECG de 12 derivações é prática usual na investigação diagnóstica e na avaliação prognóstica da miocardite ( Tabela 4 ). As alterações mais frequentemente associadas com a miocardite no ECG de 12 derivações e/ou *Holter* e/ou testes de estresse, com qualquer um dos seguintes: bloqueio atrioventricular de I a III graus ou bloqueio de ramo, alteração de ST/T (elevação de ST ou sem elevação do segmento ST, inversão da onda T), parada sinusal, taquicardia ou fibrilação ventriculares e assistolia, fibrilação atrial, redução da altura da onda R, atraso da condução intraventricular (complexo QRS alargado), ondas Q anormais, baixa voltagem, batimentos prematuros frequentes, taquicardia supraventricular.

**Tabela 4:** – Recomendações de eletrocardiograma para avaliação de miocardite

Indicações	Classe	Nível de evidência
Eletrocardiograma na suspeita de miocardite	I	C
Eletrocardiograma para avaliar prognóstico na miocardite	I	C

### 4.4.2. Prognóstico

Alargamento do QRS, BAV de alto grau, taquicardia ventricular e fibrilação atrial aumentaram a mortalidade.

## 4.5. Ecocardiograma

O ecocardiograma tem um papel limitado no diagnóstico da miocardite propriamente dita. Trata-se de uma ferramenta muito importante na exclusão de outras patologias, devendo sempre ser realizada quando ocorre a suspeita clínica ( Tabela 5 ).^96 , 97^ Não existe um achado ecocardiográfico específico, e as alterações encontradas apenas vão espelhar um quadro inflamatório miocárdico. Portanto, podemos evidenciar desde alterações segmentares (diagnóstico diferencial com as cardiopatias isquêmicas) até alterações difusas (hipocinesia global de um ou ambos os ventrículos).^98 , 99^ Quando o acometimento é agudo e grave, as cavidades ventriculares são pequenas (não dilatadas) e podemos evidenciar a presença de edema miocárdico (aumento da espessura parietal), bem como derrame pericárdico, achados esses comuns na miocardite fulminante. O acometimento do ventrículo direito (VD) geralmente reflete um prognóstico mais reservado.^100^

**Tabela 5:** – Recomendações para realização de ecocardiograma na avaliação inicial da miocardite

Indicações	Classe	Nível de evidência
Ecocardiograma para avaliação de estrutura e função cardíaca	I	C
Ecocardiograma para avaliação e estratificação prognóstica	I	C
Ecocardiograma para guiar a biópsia endomiocárdica	IIa	C

Um papel interessante do ecocardiograma é como adjunto na realização da BEM, visando não só ao sítio ideal para a retirada dos fragmentos, mas também guiando o intervencionista e evitando complicações ( Tabela 5 ).^101^

## 4.6. Ressonância Magnética Cardíaca

Na avaliação dos pacientes com miocardite, assim como na avaliação de outras cardiomiopatias não isquêmicas, a ressonância magnética cardíaca (RMC) apresenta grande utilidade na determinação dos parâmetros morfológicos e funcionais ventriculares. De fato, já foi amplamente validada para quantificar os volumes, a massa e a função tanto do VE quanto do VD, e é considerada, atualmente, a modalidade diagnóstica padrão-ouro para essa avaliação. Dada a sua alta resolução espacial e temporal, e devido a sua natureza tridimencional, que a torna independente de premissas geométricas, a RMC apresenta excelente acurácia e reprodutibilidade características especialmente úteis ao acompanhamento longitudinal dos pacientes.^102^

Entretanto, o maior valor da RMC na avaliação dos pacientes com suspeita ou diagnóstico confirmado de miocardite consiste na sua capacidade de proporcionar detalhada caracterização tecidual. Dessa maneira, permite identificar tanto a lesão miocárdica inflamatória das fases aguda e subaguda quanto as lesões cicatriciais frequentemente presentes na fase crônica da doença. As principais técnicas de RMC classicamente utilizadas na caracterização da lesão miocárdica dos pacientes com miocardite são as sequências ponderadas em T2 (“ *T2 imaging* ”) e a técnica do realce tardio.^103 - 108^

Nas imagens adquiridas pelas sequências ponderadas em T2, quanto maior for o conteúdo líquido de um determinado tecido, maior será sua intensidade de sinal. Portanto, essa técnica permite avaliar o edema miocárdico secundário ao processo inflamatório nos pacientes com miocardite aguda (“ *edema imaging* ”).^102 - 105^ A técnica do realce tardio, por sua vez, permite identificar as regiões de necrose no caso das miocardites agudas ou subagudas, e as regiões de fibrose no caso das miocardites crônicas.^106 , 108 - 110^ Cabe ressaltar que o padrão de realce tardio da miocardite é muito diferente daquele observado nos casos de IAM. A principal diferença é que, no caso do infarto, o realce tardio sempre acomete o subendocárdio. O acometimento pode até ser transmural, mas a camada subendocárdica sempre está envolvida. No caso da miocardite, o realce tardio é mais frequentemente mesoepicárdico, na maior parte das vezes poupando o endocárdio. Além disso, enquanto, no infarto, as regiões de realce tardio tendem a ser únicas, homogêneas e distribuídas de acordo com os territórios coronarianos, no caso da miocardite, as regiões de realce costumam ser multifocais, heterogêneas e esparsas, não respeitado os territórios coronarianos.

O Consenso de Lake Louise (CLL) original,^105^ publicado em 2009, se baseava em três técnicas de RMC. Além da técnica de imagem ponderada em T2 (“ *edema imaging* ”) e da técnica do realce tardio, ambas mencionadas anteriormente, incluía também a chamada técnica do realce miocárdico precoce. Esta última acabou por ser excluída na atualização dos critérios diagnósticos, após ficar demonstrado que não adicionava valor diagnóstico incremental às demais técnicas. Na prática, o realce miocárdico precoce já não vinha sendo utilizado clinicamente na maior parte dos centros de RMC do mundo.

Recentemente, novas técnicas de RMC capazes de medir os tempos de relaxamento longitudinal (T1) e transversal (T2) do miocárdico foram introduzidas como métodos potencialmente sensíveis e específicos para a detecção de processo inflamatório miocárdico.^111^ Em geral, os valores de T1 ou T2 são medidos *pixel* a *pixel* e apresentados na forma de mapas paramétricos, os chamados mapas T1 e T2 do miocárdio. O mapa T1 pode ser adquirido antes do contraste (T1 nativo) e 15 a 20 minutos após contraste (momento de relativo equilíbrio da concentração de gadolínio), permitindo, assim, o cálculo do volume extracelular do miocárdio (VEC ou ECV [do inglês, *extracellular volume* ]). O mapa T2 é usualmente adquirido apenas antes da administração do contraste.

A incorporação dos mapas T1 e T2 constituiu a motivação central para a recente atualização do CLL para o diagnóstico de miocardite pela RMC. De acordo com o novo consenso,^104^ esse diagnóstico se baseia na presença de dois critérios principais que podem estar ou não associados a critérios de suporte ( Tabela 6 ). O primeiro critério diagnóstico principal tem por objetivo identificar a presença de edema miocárdico e se fundamenta na utilização de técnicas baseadas em T2: (1) técnica de imagem ponderada em T2 (“ *edema imaging* ”) e/ou (2) técnica de mapeamento T2. O segundo critério diagnóstico principal também permite detectar a presença de edema miocárdico, mas tem por objetivo primordial identificar a presença de necrose, fibrose e extravasamento capilar. Este segundo critério diagnóstico principal se fundamenta na utilização de técnicas baseadas em T1: (1) técnica do realce tardio e/ou (2) técnicas de mapeamento T1 (T1 nativo ou VEC).

**Tabela 6:** – Critérios para diagnóstico de miocardite, miopericardite ou perimiocardite

Critério Lake Louise atualizado 1 CRITÉRIO T2 POSITIVO + 1 CRITÉRIO T1 POSITIVO	Alvo diagnóstico
**CRITÉRIOS PRINCIPAIS**
**Imagem baseada no T2**	
Intensidade de sinal aumentada regional do VE (análise visual) ou	E
Intensidade de sinal global aumentada – relação ≥ 2 ou	
Tempos de T2 (mapa T2) aumentados global ou regionalmente	EDEMA
**Imagem baseada no T1 **
Aumento regional ou global nos tempos de T1 (mapa T1) ou VEC ou	Aumento de T1 = edema (intra ou extracelular), hiperemia, extravasamento capilar, necrose, fibrose Aumento do VEC = edema (extracelular), hiperemia, extravasamento capilar, necrose, fibrose
Áreas com aumento de intensidade de sinal em padrão de distribuição não isquêmico em imagens de realce tardio	Realce Tardio = necrose, fibrose
**CRITÉRIOS DE SUPORTE**
Derrame pericárdico nas imagens de cine-RM ou aumento de intensidade de sinal do pericárdio em imagens de realce tardio, mapa T1 ou mapa T2	Inflamação pericárdica
Alteração de contratilidade do VE em imagens de cine-RM	Disfunção do VE

*VE: ventrículo esquerdo; RM: ressonância magnética; VEC: volume extracelular.*

Os novos critérios para diagnóstico de miocardite, miopericardite ou perimiocardite e publicados em 2018 estão listados na Tabela 6 .^104^

A acurácia da RMC na avaliação dos pacientes com suspeita de miocardite no primeiro CLL foi estimada em 78% (sensibilidade de 67% e especificidade de 91%).^105^ Essas estimativas foram posteriormente confirmadas em uma metanálise que demonstrou acurácia de 83%, com uma sensibilidade de 80% e especificidade de 87%.^112^ De modo similar, outra metanálise ainda mais recente demonstrou sensibilidade de 78% e especificidade de 88%, com uma área sob a curva (AUC) de 83%.^113^ Ainda não existem dados consistentes avaliando a acurácia da RMC utilizando os critérios diagnósticos propostos na segunda versão do CLL. Entretanto, um pequeno estudo recente que incluiu apenas 40 pacientes com miocardite aguda demonstrou sensibilidade de 88% e especificidade de 96% da RMC utilizando os novos critérios revisados (ver Tabela 6 ).^114^

As recomendações para o uso da RMC na avaliação diagnóstica e prognóstica dos pacientes com suspeita de miocardite aguda estão sumarizadas na Tabela 7 .^57 , 104 , 109 , 114 - 116^

**Tabela 7:** – Recomendações para o uso da ressonância magnética cardíaca na avaliação diagnóstica dos pacientes com suspeita de miocardite aguda

Indicações	Classe	Nível de evidência
Avaliação de pacientes com elevação dos marcadores de necrose miocárdica e coronárias normais na avaliação angiográfica	I	B
Avaliação dos pacientes portadores de cardiomiopatia dilatada e suspeita de miocardite > 6 meses de evolução, com o objetivo de auxiliar na investigação etiológica, excluir possíveis diagnósticos diferenciais e proporcionar informações prognósticas	I	B
Reavaliação em até 4 semanas para pacientes com risco prognóstico intermediário ou alto após o episódio agudo, com o objetivo de diferenciar uma evolução não complicada de um curso complicado	IIa	B

Com base no conjunto das evidências científicas acumuladas desde a primeira versão desta diretriz da SBC, podemos, hoje, indicar uma posição da RMC mais estruturada na tomada de decisão de pacientes com suspeita de miocardite como proposto na estratificação de risco a seguir, na Tabela 8 .^109 , 115 , 117^ Tal estratificação deve ser integrada à estratificação de risco ampla que inclui a apresentação clínica e outros exames complementares.

**Tabela 8:** – Estratificação de risco e probabilidade da indicação de biópsia endomiocárdica baseada nos parâmetros de ressonância magnética cardíaca (RMC)

Risco prognóstico	Parâmetro da RMC	Conduta sugerida	Indicação de biópsia
Baixo	T1 e T2, sem alteração Sem disfunção ventricular	Seguimento clínico	Sem indicação
Intermediário	T1 ou T2, positivos Realce tardio não extenso (<17g e 13% da massa do VE) Função normal ou leve disfunção do VE	Seguimento clínico Repetição da RMC em 1, 3 e 6 meses	Estável: sem indicação Progressão da disfunção: possível indicação
Alto	T1 ou T2, positivos Realce tardio extenso (>17g ou 13% da massa do VE), ou com envolvimento do septo interventricular, e/ou disfunção do VE moderada ou importante	Seguimento clínico Repetição da RMC em 1, 3 e 6 meses	Possível indicação

*VE: ventrículo esquerdo.*

## 4.7. Medicina Nuclear

A medicina nuclear tem tido um papel crescente na avaliação do paciente com miocardite. Novos radiotraçadores e novos equipamentos têm traçado todo um novo espectro de contribuições para o manejo de pacientes com suspeita de doenças inflamatórias do miocárdio.

As alterações fisiopatológicas dos diversos tipos de miocardite vão formar a base para o uso das técnicas de medicina nuclear: o processo inflamatório que leva à lesão do miocárdio é caracterizado por infiltração de linfócitos e macrófagos no miocárdio, pelo aumento da permeabilidade vascular e pelo consumo aumentado de glicose no sítio de inflamação e pela necrose celular com redução da perfusão tecidual em comparação com o miocárdio íntegro. Essas características vão se traduzir pela maior captação de citrato de Gálio-67 no miocárdio (especialmente útil nos casos de sarcoidose), pelo aumento do acúmulo de glicose marcada com flúor radioativo (^18^F-FDG) e pela redução da perfusão miocárdica vista com traçadores com 99mTc-Sestamibi ou 201 Tálio. A Tabela 9 lista os principais radiotraçadores utilizados na miocardite.

**Tabela 9:** – Principais exames de medicina nuclear empregados em pacientes com suspeita ou diagnóstico de miocardite

Exame de medicina nuclear	Principais indicações	Vantagens	Desvantagens
Cintilografia com Gálio-67	Miocardite e sarcoidose	Ampla disponibilidade	Menor sensibilidade
PET com ^18^F-FDG	Sarcoidose, miocardite lúpica, arritmias cardíacas inexplicáveis	Elevada sensibilidade, uso no acompanhamento da resposta ao tratamento	Menor disponibilidade, maior custo
Cintilografia com ^123^I-MIBG	Avaliação do risco de arritmias ventriculares	Identifica pacientes em risco de morte súbita	Menor disponibilidade de acesso

*PET ^18^F-FDG: tomografia por emissão de pósitrons ^18^F-fluorodesoxiglicose.*

### 4.7.1. Radiotraçadores para Cintilografia por Emissão de Fóton Único (SPECT)

O citrato de Gálio-67 é um traçador consagrado para pesquisa de infecção em medicina nuclear que se liga a células inflamatórias em sítios de aumento de permeabilidade vascular graças à sua característica ligação com as proteínas transportadoras do ferro como a lactoferina e nos lisossomos leucocitários. O Gálio-67 tem baixa sensibilidade (36%) para detecção de miocardite em pacientes com miocardiopatia dilatada de início recente e não deve ser empregado de rotina com essa indicação ( Tabela 10 ).^118^ O único tipo de miocardite com alto rendimento positivo para a cintilografia com Gálio-67 é a decorrente da sarcoidose, em que os granulomas com células gigantes são especialmente ávidos para a retenção do radiotraçador. A presença de cintilografia com Gálio-67 positiva é considerada como um critério maior para o diagnóstico de sarcoidose cardíaca pelo consenso de especialistas da Heart Rhythm Society (HRS).^119^ Outro achado significativo observado em pacientes com sarcoidose cardíaca é a alteração de perfusão decorrente da presença de constrição microvascular miocárdica nos vasos circunjacentes aos granulomas. O defeito de perfusão observado na cintilografia em repouso pode desaparecer na imagem de estresse, um padrão denominado redistribuição reversa que pode ser associado à sarcoidose.

**Tabela 10:** – Recomendações para o uso dos exames de medicina nuclear na avaliação diagnóstica dos pacientes com suspeita de miocardite aguda

Indicações	Classe	Nível de evidência
PET com ^18^F-FDG para auxiliar no diagnóstico de miocardite	IIa	B
Cintilografia com Gálio-67 para auxiliar no diagnóstico de miocardite	IIb	B

*PET ^18^F-FDG: tomografia por emissão de pósitrons ^18^F-fluorodesoxiglicose.*

A cintilografia com Gálio-67 pode ser empregada como alternativa a pacientes sem acesso ou que tenham contraindicação à realização de RMcom gadolínio (claustrofobia, alergia ao contraste, insuficiência renal) e pode contribuir em casos suspeitos de miocardite por critérios clínicos (febre, história recente de infecção respiratória ou intestinal, elevação de marcadores de necrose), sendo útil também no diagnóstico diferencial entre IAM com coronárias normais e miocardite, conforme o estudo de Hung et al.,^120^ em que a técnica se mostrou positiva quando realizada precocemente após o surgimento de sintomas.^120^ Alguns casos de miocardite podem apresentar agressão regional no miocárdio e ser a etiologia de arritmias, **onde os** estudos com Gálio-67 podem demonstrar acúmulo focal em áreas dos ventrículos e até mesmo dos átrios isoladamente.^121^

### 4.7.2. Radiotraçadores para Tomografia por Emissão de Pósitrons (SPECT)

O ^18^F-FDG é captado pelas células inflamatórias como transporte ativo de modo independente da ação da insulina. Dessa maneira, quando é realizada uma adequada supressão da captação de glicose pelo miocárdio, o PET com^ 18^F-FDG se transforma em uma sensível ferramenta para diagnóstico de inflamação miocárdica e para acompanhamento da mesma em resposta ao tratamento ( Tabela 10 ).

**Figura 3:**
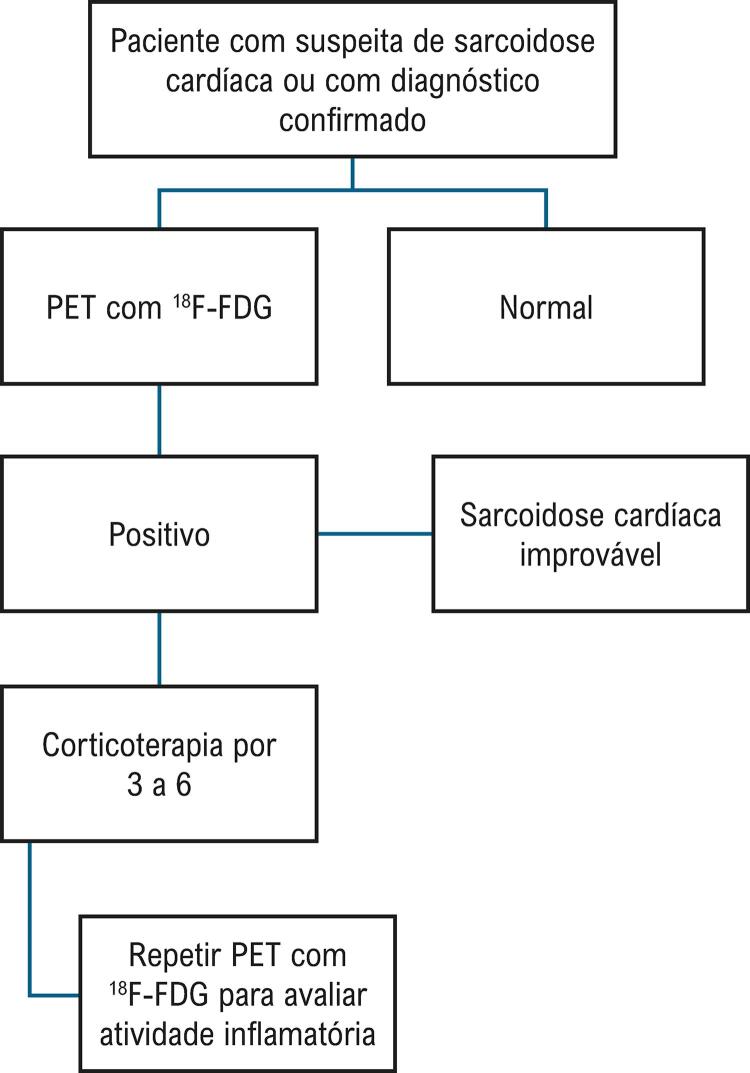
– Algoritmo proposto para diagnóstico e acompanhamento da resposta ao tratamento na sarcoidose cardíaca. PET 18F-FDG: tomografia por emissão de pósitrons 18F-fluorodesoxiglicose.

O maior número de estudos do uso do PET com ^18^F-FDG na miocardite está concentrado na sarcoidose cardíaca, em que recente metanálise demonstrou sensibilidade de 84% e especificidade de 83%.^122^ Para que o PET com ^18^F-FDG seja útil na sarcoidose ou em outras afecções inflamatórias cardíacas como miocardite, endocardite infecciosa ou na rejeição após transplante, é crucial o adequado preparo do paciente para evitar que insulina circulante leve a acúmulo não inflamatório de ^18^F-FDG no miocárdio. Entre os diversos esquemas de preparo indicados, o uso do jejum prolongado de 12 horas a 18 horas antes da injeção do radiotraçador é um dos mais aplicados, bem como a utilização de uma dieta rica em lipídios e proteínas, enquanto o uso da heparina não é consensual.^123 , 124^ O marco diagnóstico de atividade inflamatória é a captação focal do ^18^F-FDG no miocárdio, enquanto há significado prognóstico a presença de captação de ^18^F-FDG no VD e a presença de captação inflamatória em áreas de hipoperfusão, as denominadas áreas de discordância ( *mismatch* ): metabolismo aumentado com perfusão reduzida.^124^ A utilização do PET com ^18^F-FDG também é empregada para acompanhamento da resposta ao tratamento na sarcoidose cardíaca e para avaliação da atividade da doença extracardíaca. Um algoritmo proposto de acompanhamento é o da Figura 3 , adaptado de Young et al.^125^

Adaptado de Young et al.^125^

A miocardite não associada à sarcoidose tem como técnica diagnóstica padrão a RMC. O aumento da intensidade de sinal das imagens pesadas em T2 (edema), o aumento do realce precoce de gadolínio (hiperemia) e a impregnação tardia de gadolínio no miocárdio (realce tardio para necrose) têm, combinados, sensibilidade de 67% e especificidade de 91% para o diagnóstico de miocardite. Entretanto, em muitos casos, há limitações para uso adequado da técnica como baixa qualidade do sinal nas imagens em T2, artefatos e impossibilidade do uso do contraste gadolínio. Nesses casos, o uso do PET ^18^F- FDG pode ser bastante útil na complementação da investigação diagnóstica, seja em equipamentos de PET-CT ou mais modernamente em equipamentos de PET-RM, que associam ao PET um equipamento de RM.^126^ Estudos com PET-RM têm demonstrado que o PET é superior à RM na identificação de áreas de inflamação cardíaca em atividade.^127^

O PET-CT com ^18^F-FDG tem sido utilizado em condições como lúpus eritematoso sistêmico,^128^ miocardite de células gigantes,^129^ esclerodermia^130^ e até mesmo na cardite reumática,^131^como técnica para identificação de inflamação em atividade com sucesso. Outro uso recente do PET com ^18^F-FDG que vem crescendo é na investigação da etiologia de arritmias: sarcoidose cardíaca e miocardite crônica, incluindo doença de Chagas, como causa de arritmias ventriculares,^132^ bem como na investigação de distúrbios de condução, especialmente indivíduos com menos de 50 anos de idade e bloqueio atrioventricular em que o PET tem identificado diversos casos de sarcoidose e mesmo de tuberculose cardíaca como causa do distúrbio de condução.^133^ No estudo de Tung et al., 50% dos pacientes com miocardiopatia e arritmias ventriculares inexplicáveis tiveram o PET com ^18^F-FDG positivo, indicando a presença de miocardite não suspeita por outras técnicas.^134^

### 4.7.3. Perspectivas Adicionais

Novos radiotraçadores têm sido avaliados em pacientes com inflamação miocárdica, como é o caso do 68Gálio-dotatate, que tem afinidade pelos receptores de somatostatina que estão expressos em células inflamatórias. Outro radiotraçador que tem sido analisado é o 123I-MIBG, que avalia o estado da inervação adrenérgica pré- sináptica cardíaca. Apesar de o radiotraçador não identificar de modo direto o estado inflamatório, ele tem relação importante com o risco aumentado de arritmias ventriculares, em especial, em pacientes com miocardite crônica chagásica, demonstrando as áreas de miocárdio viável que são denervadas e, por isso, mais vulneráveis à taquicardia ventricular sustentada.^135^

## 4.8. Angiotomografia de Coronárias e Coronariografia

A miocardite aguda pode mimetizar IAM com dor torácica típica, anormalidades no ECG similares ao IAM com ou sem supradesnivelamento do segmento ST, elevação das enzimas cardíacas e instabilidade hemodinâmica.^136^

Na suspeita de miocardite com apresentação parecida com um infarto, é necessário excluir DAC por coronariografia percutânea ou angiotomografia de coronárias. A cinecoronariografia de rotina também deve ser realizada durante a investigação de uma nova cardiomiopatia dilatada.^137^

A análise de 46 publicações avaliando a fisiopatologia subjacente de IAM com artérias coronárias não obstrutivas (MINOCA) revelou um infarto típico na RMC em apenas 24% dos pacientes, miocardite em 33% e sem anomalia significativa em 26%.^138^ A idade jovem e a PCR estavam associadas à miocardite, enquanto sexo masculino, hiperlipidemia tratada, alta razão de troponina e baixa PCR estavam associados ao IAM verdadeiro.^139^

Como pacientes com miocardite aguda que imitam o IAM com supradesniveamento do segmento ST têm um prognóstico favorável, é importante estabelecer o diagnóstico correto para evitar tratamentos desnecessários e potencialmente perigosos.^139^

A angiotomografia computadorizada de coronárias (angio-TC) é um exame simples e rápido, e fornece uma avaliação abrangente das características das artérias coronárias e do tecido miocárdico. Na prática, a aquisição da angio-TC em primeira passagem permite a avaliação da anatomia coronariana e do realce do ventrículo esquerdo. A aquisição tardia de angio-TC é realizada 3 a 5 minutos mais tarde, sem necessidade de reinjeção do meio de contraste, permitindo a captação de iodo em imagens tardias com contraste realçados de maneira semelhante à RM do coração.^140 , 141^

A angio-TC e a RM do coração têm maneiras próprias e exclusivas de evitar uma angiografia coronariana invasiva, para excluir DAC (significativa) e para detectar outras doenças, como dissecção aguda da aorta, embolia pulmonar, miocardite ou cardiomiopatia de estresse.^142^

A grande disponibilidade da angio-TC, combinada com a possibilidade de descartar síndrome coronariana aguda (SCA) com angiografia coronariana durante o mesmo exame, torna-a promissora no refinamento das imagens de miocardite aguda ( Tabela 11 ).^141^

**Tabela 11:** – Indicação de angiotomografia computadorizada de coronárias na avaliação diagnóstica dos pacientes com suspeita de miocardite aguda

Indicação	Classe	Nível de evidência
Angiotomografia de coronárias para exclusão de coronariopatia obstrutiva grave na investigação de miocardite como alternativa à coronariografia em pacientes com probabilidade pré-teste baixa ou intermediária de DAC	I	C

*DAC: doença arterial coronariana.*

Em crianças com suspeita de miocardite e doença de Kawasaki, a angiotomografia computadorizada pode ser usada na avaliação das anormalidades nas artérias coronarianas.^143^

A última diretriz da European Society of Cardiology (ESC) sugere que, na ausência de doença arterial coronariana angiograficamente significativa (estenose ≥50%) ou condições preexistentes que poderiam explicar o cenário clínico, pacientes que têm pelo menos uma das cinco apresentações clínicas (dor torácica aguda; IC aguda ou com piora com ≤3 meses de dispneia, fadiga e/ou sinais de IC; IC crônica com >3 meses de dispneia, fadiga e/ou sinais de IC; palpitações, sintomas de arritmias inexplicáveis e/ou síncope e/ou morte abortada; choque cardiogênico inexplicável) e/ou certos testes diagnósticos de suporte (ECG, *Holter* , troponina, anormalidades de função ventricular e edema e/ou realce tardio do gadolínio com padrão miocárdico clássico) devem ser considerados como tendo “suspeita clínica de miocardite” e, assim, justificar uma avaliação adicional.^12 , 72^

## 4.9. Biópsia Endomiocárdica: Indicações, Técnica e Complicações

A análise histopatológica do tecido do miocárdio é ferramenta importante para diagnóstico e prognóstico nos pacientes com miocardite. A biópsia endomiocárdica (BEM) utilizando critérios histopatológicos padronizados (critérios de Dallas)^144^ e imuno-histoquímicos é o atual padrão-ouro para diagnóstico de miocardite.^137^

Os critérios de Dallas, isoladamente, apresentam limitações, em virtude do alto grau de variabilidade interobservador na interpretação patológica e detecção de processos inflamatórios não celulares, diagnosticando em torno 10% a 20% dos pacientes.^15^ Assim, de acordo com a definição da OMS, a imuno-histoquímica com o uso de painel de anticorpos monoclonais e policlonais é mandatória para diferenciar os componentes inflamatórios presentes.^145 , 146^

A análise genômica viral no miocárdio doente, quando acoplada com as análises imuno-histoquímicas, melhorou a precisão e a utilidade diagnóstica e prognóstica da BEM.^147^ É recomendada a triagem viral: enterovírus, influenza, adenovírus, citomegalovírus, vírus Epstein-Barr, parvovírus B19, herpes-vírus humano.^137^

No entanto, como alguns genomas virais (p. ex., PVB19) podem ser detectados em corações normais e em doenças cardíacas isquêmicas e valvares,^148^ pode ser necessário o uso complementar de mRNA específicos de DNA virais para definir infecção ativa.^149^

### 4.9.1. Ponderações para Indicação

A BEM realizada precocemente na apresentação clínica grave auxilia no diferencial diagnóstico de tipos específicos de miocardite (células gigantes, alérgica, eosinofílica, sarcoidose) que implicam diferentes tratamentos (p. ex., imunossupressores) e prognóstico ( Tabela 12 ).^150^

**Tabela 12:** – Recomendações para a utilização de biópsia endomiocárdica (BEM)

Indicações	Classe	Nível de evidência
IC de início recente (<2 semanas), sem causa definida, não responsiva ao tratamento usual e com deterioração hemodinâmica	I	B
IC de início recente (2 semanas a 3 meses), sem causa definida e associada a arritmias ventriculares ou bloqueios atrioventriculares de segundo ou terceiro grau	I	B
Na presença de suspeita clínica de miocardite linfocítica grave, miocardite de células gigantes, miocardite eosinofílica necrosante	I	B
IC com início >3 meses e <12 meses, sem causa definida, não responsiva à terapia-padrão otimizada	IIa	C
IC decorrente de cardiomiopatia dilatada de qualquer duração, com suspeita de reação alérgica e/ou eosinofilia	IIa	C
Arritmias ventriculares frequentes na presença ou não de sintomas, sem causa definida	IIb	C
Suspeita clínica apoiada por métodos diagnósticos não invasivos de miocardite	IIb	C

*IC: insuficiência cardíaca.*

Além disso, fornece diagnóstico diferencial de doenças que podem simular miocardite (cardiomiopatia arritmogênica do ventrículo direito, cardiomiopatia de Takotsubo, cardiomiopatia periparto, distúrbios inflamatórios/de armazenamento).^150^

Atualmente, a principal indicação para BEM ocorre em pacientes com IC de início recente (menos de 2 semanas), acompanhada de apresentação clínica grave (instabilidade hemodinâmica, uso de suporte circulatório mecânico ou inotrópico, refratariedade ao tratamento clínico) ou arritmias de alto risco (arritmias ventriculares sustentada ou sintomática ou bloqueios cardíacos de alto grau) ( Tabela 12 ).^151 , 152^

No entanto, sabe-se as recomendações antecedentes foram baseadas notadamente nos critérios de Dallas, nos quais diagnóstico, valor prognóstico e terapêutico é limitada. Com o uso da análise imuno-histoquímica e genômica viral, cresce a tendência de uma aplicação mais liberal da BEM na suspeita de miocardite clinicamente independente do padrão e gravidade da apresentação.^12^

**Figura 4:**
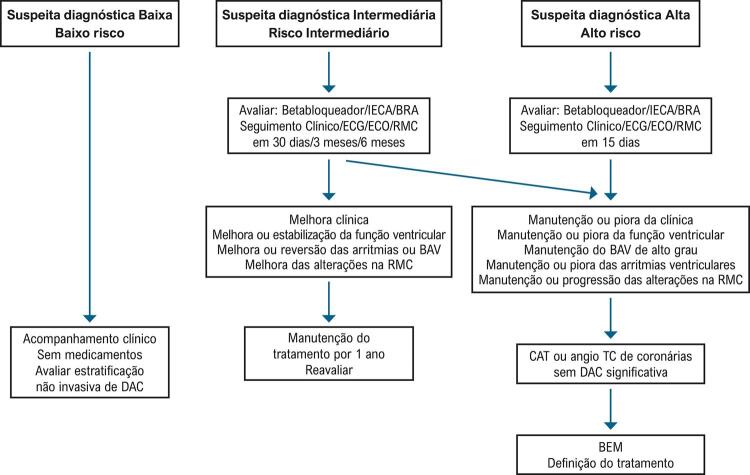
– Fluxograma terapêutico de miocardite com base no grau de suspeição clínica e no prognóstico.

BAV: bloqueio atrioventricular; BEM: biópsia endomiocárdica; BRA: bloqueador do receptor da angiotensina; CAT: coronariografia; DAC: doença arterial coronariana; ECG: eletrocardiograma; ECO: ecocardiograma transtorácico; ESV: extrassístoles ventriculares; IECA: inibidor da enzima de conversão; RMC: ressonância magnética cardíaca; TC: tomografia computadorizada.

Por outro lado, o valor de BEM é questionável em pacientes que apresentam síndromes de baixo risco e respondem a tratamento padrão sem perspectiva de implicação terapêutica ou prognóstica. Finalmente, no cenário de síndromes de risco intermediário, a BEM deve ser considerada no caso de manutenção ou agravamento dos sintomas, disfunção ventricular, arritmias, distúrbios de condução ( Figura 4 ).^153^

### 4.9.2. Prognóstico

Enquanto os critérios de Dallas não são um preditor preciso de resultados clínicos, as evidências imuno-histológicas de inflamação miocárdica estão associadas a um risco aumentado de morte cardiovascular e necessidade de transplante cardíaco.^153^

Na miocardite por células gigantes, a gravidade da necrose e fibrose está associada a um risco aumentado de morte e transplante.^154^

A ausência ou presença de genomas enterovirais residuais em amostras repetidas correlacionou-se com a progressão para a cardiomiopatia em estágio terminal, enquanto depuração viral espontânea foi associada à melhora da função sistólica.^155^

### 4.9.3. Técnica

O procedimento deve ser realizado no laboratório de hemodinâmica, por hemodinamicista com experiência na realização desse procedimento. A anestesia é local com sedação consciente, se necessário, sempre sob a supervisão do anestesiologista.

A BEM pode ser realizada de maneira segura, guiada por fluoroscopia direta, e deve ter auxílio do ecocardiograma na sua realização que servirá de guia para o posicionamento correto do biótomo para que se evite puncionar a parede livre do VD.

A RMC é particularmente útil para facilitar uma abordagem guiada, em virtude de sua utilidade na distinção entre miocárdio normal e doente, e tem sido avaliada para aumentar valores preditivos.^155^

Não existem estudos comparativos para que se recomende a biópsia endocárdica do VD ou do VE; entretanto, a realização da BEM do VE deve ser criteriosamente analisada em casos de doença restrita ou predominante em VE.

As amostras devem ser obtidas no ventrículo direito, especialmente a porção distal do septo interventricular e a área trabeculada apical, evitando-se a parede livre do VD. O número de amostras dependerá da pesquisa a ser realizada. No caso de investigação de miocardite viral, devem ser 10 amostras (6 para pesquisa viral, 2 para hematoxilina-eosina e 2 para imuno-histoquímica). No caso de investigação de doenças infiltrativas ou de depósito, 6 fragmentos (2 para hematoxilina-eosina, 2 para imuno-histoquímica e 2 para microscopia eletrônica). As amostras para HE e imuno-histoquímica devem ser colocadas em frasco de formalina tamponada a 10% e não devem ser refrigeradas. As amostras para pesquisa viral devem ser colocadas em microtubos tipo Eppendorf® (sem soluções de transporte), e estes em recipientes com gelo seco, e rapidamente transferidas para refrigeradores –70 graus para armazenamento. As amostras para microscopia eletrônica devem ser acondicionadas em tubos Eppendorf® com solução oct.

A BEM pode ser repetida, se necessário, para monitorar a resposta à terapia dirigida à etiologia ou se houver suspeita de erro de amostragem em um paciente com progressão inexplicada de IC.^156^

### 4.9.4. Complicações

Embora a BEM tradicional seja considerada um procedimento seguro, diferentes complicações foram relatadas.

Quando realizada em centros experientes, sua principal taxa de complicações é <1%, o que é semelhante ao da angiografia coronariana.^97^ A utilização do ecocardiograma associado à fluoroscopia reduz de forma significativa a possibilidade de punção inadvertida que possa ocasionar perfuração miocárdica ou lesão de coronária.^155^

Podemos distinguir complicações relacionadas ao acesso vascular e inserção da bainha e complicações relacionadas à remoção de amostras. As complicações relacionadas ao acesso vascular são: punção arterial incidental; sangramento prolongado; hematoma e dissecção vascular.

As comumente descritas são: reação vasovagal, BAV de graus variados, perfuração de parede livre de VD, pneumotórax, perfuração do septo interventricular, hematoma de sítio de punção, fístulas intracardíacas, hematoma retroperitoneal (acesso femoral), derrame pericárdico, deslocamento de trombos, tamponamento cardíaco, ruptura de cordoalhas tricúspides, arritmias ventriculares.^157^

Em resumo, o risco da BEM depende da condição clínica dos pacientes, da experiência do operador e de todas as ferramentas tecnológicas disponíveis para prevenir, diagnosticar e gerenciar complicações.

## 4.10. Análise Histológica e Pesquisa Viral – Biologia Molecular e Genoma

### 4.10.1. Análise Histológica

A miocardite é definida como uma doença inflamatória do miocárdio diagnosticada por critérios histológicos e imuno-histológicos. De acordo com os critérios de Dallas, a miocardite ativa é histologicamente definida como uma infiltração inflamatória do miocárdio com necrose de miócitos adjacentes, enquanto a miocardite limítrofe é diagnosticada quando o infiltrado inflamatório está presente, mas não é demonstrada lesão/necrose nas próprias células cardíacas.^158^

No entanto, os critérios de Dallas são considerados inadequados no diagnóstico de paciente com suspeita de miocardite clinicamente devido à sua limitação quanto à variabilidade na interpretação, falta de valor prognóstico e baixa sensibilidade em virtude de erro amostral. Essa limitação pode ser superada pelo envolvimento de manchas imuno-histológicas de células infiltrativas (leucócitos/linfócitos T/macrófagos) e antígenos de superfície (ICAM-1/HLA-DR).

Além do diagnóstico da miocardite, a avaliação histopatológica dos critérios histológicos é essencial para alcançar uma classificação da miocardite nas formas linfocítica, eosinofílica, célula gigante, granulomatosa e/ou polimórfica, que geralmente refletem etiopatogênese diferente do processo inflamatório.^12^

Além disso, o exame histológico das seções de parafina por diferentes protocolos de coloração (HE, EvG, PAS, Azan) é usado para detectar morte celular do miocárdio, cicatrizes, fibrose, disfunções, alterações dos cardiomiócitos e condições vasculares patológicas. Amiloidose, depósitos de ferro, glicogênio e outras doenças de armazenamento podem ser excluídas ou especificadas por coloração adicional.

### 4.10.2. Análise Imuno-histoquímica

A imuno-histoquímica aumentou significativamente a sensibilidade da BEM e fornece informações sobre o prognóstico clínico. A precisão diagnóstica da imuno- histologia para detecção de inflamação é maior que a dos critérios histológicos. A avaliação imuno-histoquímica é baseada na análise de reação específica antígeno-anticorpo. Um valor >14 leucócitos/mm^2^ com presença de pelo menos linfócitos T >7 células/mm^2^ foi considerado um corte realista para se chegar ao diagnóstico de miocardite.^12^

Quantificação de células infiltrativas adicionais, incluindo macrófagos (Mac- 1/CD69), células CD4+, CD8+, células citotóxicas (perforina) e quantificação do antígeno leucocitário humano (HLA-DR) e molécula intracelular de adesão celular- 1 (ICAM- 1) é obrigatória para caracterizar ainda mais as populações de células inflamatórias. Assim, a caracterização e a quantificação exata da inflamação do miocárdio é relevante para o prognóstico e para identificar diferentes marcadores de miocardite crônica/autoimune aguda, infecciosa, negativa por vírus (ver Figura 4 ).

Outras manchas de imunofluorescência devem ser usadas para definir a rejeição humoral na BEM de transplante cardíaco, como C3d e C4d, ou para subtipagem de formas amiloides.

### 4.10.3. Análise do Perfil Genético

Miocardite idiopática de células gigantes e sarcoidose cardíaca são distúrbios raros que causam IC aguda com choque cardiogênico e/ou arritmias ventriculares com risco de vida na ausência de outras etiologias e apresentam prognóstico extremamente ruim, com taxas de sobrevida em 4 anos inferiores a 20%.^159^

O principal problema para o diagnóstico correto é o erro de amostragem pelo exame histológico das BEM. Foram identificados perfis genéticos diferenciais distintos que permitiram uma clara discriminação entre os tecidos que abrigam células gigantes e aqueles com miocardite aguda ou controles livres de inflamação. Além disso, os perfis gênicos específicos da doença mudam durante o tratamento eficaz e podem ser aplicados no monitoramento da terapia.^160^

### 4.10.4. Virologia

Os genomas microbianos são determinados, quantificados e sequenciados usando métodos baseados em PCR, incluindo nested-PCR-RT e PCR quantitativo, determinando a análise da carga viral. A sequenciação do produto genético viral amplificado é obrigatória.

Em especial, é possível analisar todos os vírus que podem ser responsáveis pela doença. Os genomas de vírus cardiotrópicos mais comuns relatados no miocárdio são parvovírus B19 (B19V), enterovírus (EV), adenovírus (ADV), vírus da gripe, vírus do herpes humano-6 (HHV-6), vírus de Epstein-Barr, citomegalovírus, vírus da hepatite C e vírus da imunodeficiência humana (HIV) ( Tabela 13 ).

**Tabela 13:** – Vírus comuns na BEM

Vírus comuns na BEM	
Adenovírus	Parvovírus B19
Arbovírus	Poliomielite
Arenavírus	Raiva
Coronavírus	Vírus sincisial respiratório
Coxsackie vírus (A, B)	Rubéola
Citomegalovírus	Vaccinia
Dengue	Varicela-zóster
Echo vírus	Varíola
Encefalomiocardite	Vírus da Zika
Epstein-Barr	HIV
Hepatite B	Influenza (A, B)
Hepatite C	Metapneumovírus
Herpes simples	Caxumba
Herpes-vírus 6	

*BEM: biópsia endomiocárdica.*

O PVB19 é o vírus cardiotrópico predominante encontrado na miocardite. O impacto clínico no coração ainda está em discussão. O PVB19 cardiotrópico transcripcionalmente ativo com intermediários de replicação positiva nas BEM parece ser clinicamente relevante, porque os pacientes com miocardite caracterizados por PVB19 cardiotrópico transcricionalmente ativo com intermediários de replicação positivos têm uma expressão genética alterada em comparação aos pacientes com controle PVB19 latente. No entanto, a PCR pode resultar negativa, embora o organismo causal tenha origem viral, devido à depuração viral.

Embora se pense que os vírus sejam a causa mais comum de miocardite, os títulos virais não são úteis no diagnóstico e tratamento.

## 5. Tratamento

### 5.1. Fluxogramas Terapêuticos

A maioria das miocardites apresenta um prognóstico favorável com regressão espontânea dos sintomas clínicos e função ventricular preservada sem necessidade de intervenção terapêutica. O fluxograma terapêutico da miocardite na maioria dos pacientes é guiado por meio da suspeita diagnóstica, uma vez que somente uma minoria dos pacientes será submetida à investigação por BEM ( Figura 4 ).^65^

Os pacientes com baixa suspeita diagnóstica de miocardite com apresentação clínica sem sinais de gravidade, função ventricular preservada e sem arritmias ventriculares apresentam evolução prognóstica favorável, sendo seguidos por acompanhamento clínico sem utilização de terapêutica medicamentosa. Nos pacientes com suspeita diagnóstica intermediária, com função ventricular preservada ou com disfunção ventricular com melhora evolutiva, a terapêutica é cardioprotetora com betabloqueadores, inibidores da enzima de conversão ou bloqueadores do receptor da angiotensina com objetivo de preservar ou melhorar a função ventricular.^12 , 55^

**Figura 5:**
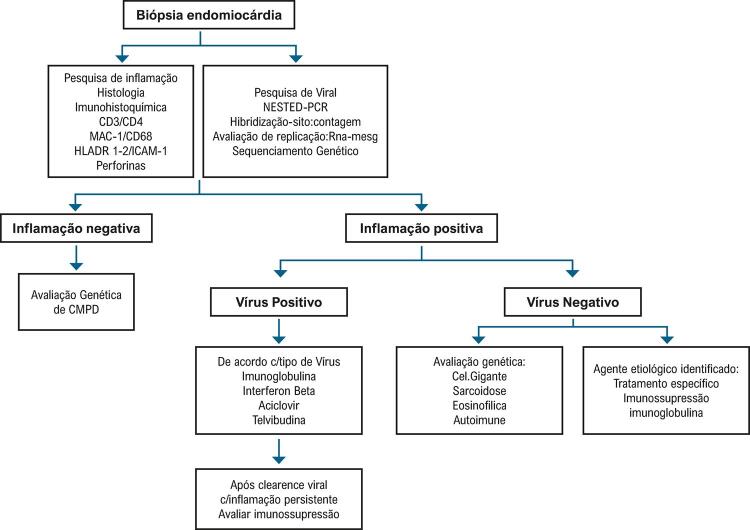
– Fluxograma terapêutico da miocardite com base nos resultados da BEM.

CMPD: cardiomiopatia dilatada; PCR: reação em cadeia da polimerase

Os pacientes com alta suspeita diagnóstica, que evoluem com algum dos indicadores de pior prognóstico, como piora clínica, instabilidade hemodinâmica, manutenção ou piora da disfunção ventricular, arritmias ventriculares frequentes e distúrbios de condução significativos, devem ser submetidos à BEM, com objetivo de pesquisa de inflamação e do agente etiológico, que oferecem a possibilidade de estabelecer terapêutica específica com imunossupressão,^161 , 162^ imunomodulação^163 - 166^ e antivirais,^167 - 170^ que poderão trazer benefícios na melhora clínica, classe funcional, função ventricular e sobrevida^8 , 162 , 171 - 175^ ( Figura 5 ).

### 5.2. Imunossupressão: Indicações e Tipos

A terapêutica imunossupressora na miocardite tem como objetivo suprimir a resposta inflamatória e a atividade autoimune, visando como alvo à melhora clínica, da função ventricular, além de redução da mortalidade.

Na miocardite linfocitária, apesar do racional fisiopatológico para utilização de imunossupressão, com base na presença de inflamação miocárdica por meio da BEM, associada à pesquisa de genoma viral negativa, as evidências corroborando seu uso são limitadas. Fatores como a regressão espontânea da inflamação, a falta de uniformidade dos estudos quanto aos critérios diagnósticos, o reduzido número de pacientes na maioria dos ensaios, a heterogeneidade das características clínicas das populações estudadas e a ausência de estudos com objetivo principal de avaliar a redução da mortalidade de forma isolada dificultam a análise dos benefícios clínicos da terapêutica imunossupressora na miocardite linfocitária ( Tabela 14 ).^55 , 161 , 162 , 172 , 176 - 179^

**Tabela 14:** – Análise dos benefícios clínicos da terapêutica imunossupressora na miocardite linfocitária

Autor	Desenho	Intervenção	Placebo	N	Doença	Duração dos sintomas	Inclusão	BEM vírus positivo	FEVE	Resultados
Parillo 1989	Randomizado controlado	Prednisona	Não	102	<2 anos	Média 8m	Idiop	Sim Não	>35%	Neutro
Latham 1989	Randomizado controlado	Prednisona	Não	52	MCD	< 2 anos Média 1,6 a 1,8 m	Idiop	Sim Não	<40%	Neutro
Wojnicz 2001	Randomizado controlado, aberto	Prednisona + Azatioprina	Sim	84	MCD	>6 meses	HLA	Sim Não	<40%	Melhora FE
Wojnicz 2006	Randomizado controlado, aberto, 2 centros	Atorvastatina	Não	74	MCD	>6 meses	HLA	Sim Não	<40%	Melhora FE/CF NYHA
Frustaci 2009	Randomizado controlado, duplo cego, multicêntrico	Prednisona + Azatioprina	Sim	85	MCD	>6 meses	CD3>7 CD45>14 Vírus neg	Sim Sim	<45%	Melhora FE
Merken 2018	Série de Casos	Prednsiona + azatioprina	Não	180	MCD	Média 8 a 11 m	CD3>7 CD45>14 Vírus neg	Sim Sim	<45%	Melhora sobrevida livre de TX/ FE

*BEM: biópsia endomiocárdica; CF NYHA: classe funcional da New York Heart Association; CMD: cardiomiopatia dilatada; FEVE: fração de ejeção de ventrículo esquerdo; HLA: antígeno de histocompatibilidade; TXC: transplante cardíaco.*

No estudo MTT,^178^ que incluiu pacientes com miocardite diagnosticada pelos critérios de Dallas associada à presença de disfunção ventricular, o uso de imunossupressão por 6 meses não demonstrou superioridade na melhora da função ventricular e de sobrevida em relação ao tratamento convencional, apesar de não ter realizado pesquisa de agentes infecciosos. O estudo italiano duplo-cego, randomizado, placebo-controlado TIMIC^162^ demonstrou melhora da função ventricular com imunossupressão em pacientes com miocardite à biópsia (acima de sete linfócitos por campo), mais de 6 meses de IC e ausência de genoma viral na BEM. Dessa forma, apesar de momento evolutivo diferente em relação à fase mais aguda da miocardite, esse estudo demonstrou o benefício da imunossupressão na ausência de genoma viral no miocárdio. No entanto, a não identificação de vírus específicos define que não estão presentes os vírus pesquisados, não afastando a possibilidade de que outros microrganismos poderiam estar presentes.^162^ Além disso, o achado qualitativo de microrganismos em BEM não estabelece uma relação causal indubitável com o desenvolvimento de miocardite/miocardiopatia, uma vez que podemos encontrar genoma viral em miocardiopatias de outras etiologias específicas e até mesmo em corações normais.^45 , 180 , 181^ Tomando como exemplo o parvovírus B19, cuja presença no tecido miocárdico pela PCR qualitativa é comum, outras técnicas documentando a baixa quantidade de cópias^167^ ou ausência de transcrição para RNA^182^ poderiam inferir a não correlação com desenvolvimento de miocardite/miocardiopatia, permitindo a consideração de imunossupressão, mesmo com o genoma desse vírus presente.

No contexto da miocardite por doenças autoimunes, a utilização de imunossupressão é bem estabelecida, e, para cada entidade, diferentes estratégias podem ser consideradas, sendo a maioria envolvendo o uso de corticosteroide, geralmente combinado com outras drogas imunossupressoras ( Tabela 15 ).^183 - 188^

**Tabela 15:** – Indicação de imunossupressão na miocardite por doença autoimune

Miocardite de células gigantes	Rara, porém fulminante, melhora de prognóstico com imunossupressão combinada	Corticosteroide em combinação (ciclosporina + azatioprina)
**Sarcoidose**	Doença sistêmica, principalmente pulmonar. Miocardite em 10%, bloqueios, taquiarritmias e disfunção ventricular	Corticosteroide em combinação (ciclofosfamida, metotrexato) imunobiológicos em casos refratários
**Lúpus eritematoso sistêmico**	Miocardite em 50%, pode ser subclínica; rara com imunossupressão atual; pode acelerar aterosclerose	Pulsoterapia com corticosteroide (depois VO em desmame), combinação (ciclofosfamida), plasmaférese, IVIg
**Esclerose sistêmica**	Miocardite ou secundária à hipertensão pulmonar. Arritmias, distúrbios de condução e disfunção ventricular	Corticosteroide em combinação (ciclosfamida, azatioprina)
**Behçet**	Miocardite é rara (0,5%), prognóstico ruim	Corticosteroide em combinação (colchicina, anticoagulação)
**GEPA (Churg-Strauss)**	Miocardite em até 50%; história de asma, presença de eosinofilia; dor torácica, palpitações até choque cardiogênico	Corticosteroide em combinação (ciclofosfamida)
**Artrite reumatoide**	Miocardite em 30%, pode ser subclínica; rara com imunossupressão atual; pode acelerar aterosclerose	Corticosteroide em combinação (metotrexate, imunobiológicos)

Devido à gravidade do quadro clínico, apesar da baixa incidência, o diagnóstico de miocardite de células gigantes não pode ser postergado, e seu tratamento envolve imunossupressão intensiva combinada. Trabalho clássico de Cooper et al.^8^demonstrou aumento de sobrevida de 3 para 12 meses, quando comparado ao não uso de imunossupressão ou apenas corticosteroide em relação ao uso combinado de imunossupressão (corticosteroide e/ou azatioprina e/ou ciclosporina e/ou anticorpo antilinfócito).^8^ Casuística mais recente demonstrou sobrevida de 58% em 5 anos com uso combinado de corticosteroide, ciclosporina e azatioprina.^189^ Em casos refratários, existe a descrição do uso de anticorpo antilinfócito,^190^ micofenolato^191^ e sirolimo.^192^

A miocardite eosinofílica pode ser secundária à reação de hipersensibilidade a drogas, doenças autoimunes (granulomatose eosinofílica com poliangeíte ou síndrome de Churg-Strauss), síndrome hipereosinofílica, infecções e câncer, ou idiopática, sendo a imunossupressão também considerada nesse contexto, habitualmente utilizando corticosteroide. Revisão recente dos casos de literatura demonstram presença de eosinofilia periférica em 75% dos casos, uso de imunossupressão em 80%, e combinação em 20% (especialmente Churg-Strauss e hipereosinofílica, com elevada mortalidade em 30 dias (13% hipereosinofílica, 17% idiopática, 23% Churg-Strauss e 40% por hipersensibilidade).^193^

A terapêutica imunossupressora mais comumente utilizada nos pacientes com diagnóstico de miocardite envolve corticosteroide isoladamente ou em associação com azatioprina ( Tabela 16 ), havendo o diagnóstico por BEM de inflamação com ausência de infecção viral como determinantes para a realização da imunossupressão ( Tabela 17 ). Os pacientes submetidos à terapêutica imunossupressora devem ser clinicamente monitorados de modo contínuo quanto ao desenvolvimento de paraefeitos, pois estes podem aumentar de forma significativa tanto a morbidade quanto a mortalidade.^55^

**Tabela 16:** – Terapêutica imunossupressora com corticosteroide

**Miocardite de células gigantes** Pulsoterapia com corticosteroide – metilprednisolona 500 a 1.000 mg por 3 a 5 dias; prednisona – 1 mg/kg e, depois, retirada lenta e gradual Anticorpo antilinfócito – Thymoglobulina – 1,5 mg/kg/dia, conforme evolução de linfócitos T CD3 – Ciclosporina – 3 a 8 mg/kg Azatioprina – 2 mg/kg
**Miocardite linfocitária e eosinofílica** Primeiras 4 semanas – 1 mg/kg 5 a 12 semanas – Redução da posologia em 0,08 mg/kg/semana 13 a 20 semanas – manter a dose em 0,3 mcg/kg/dia 21 a 24 semanas – redução da posologia 0,08 mg/kg/semana Estudo TIMIC: prednisona – 1 mg/kg por 4 semanas e 0,33 mg/kg por 5 meses; azatioprina – 2 g/kg por 6 meses
**Sarcoidose** Prednisona – 30 mg/dia – retirada de 5 mg por mês por 12 a 24 meses Associação quando dificuldade de suspensão de corticosteroide: metotrexato – 10 - 20 mg/semana Azatioprina – 2 mg/kg; hidroxicloroquina – 200 a 400 mg/dia Leflunomida – 10 a 20 mg/dia

**Tabela 17:** – Indicações da terapêutica imunossupressora na miocardite

Indicações	Classe	Nível de evidência
Na presença de miocardite positiva – por células gigantes, doenças autoimunes, sarcoidose e eosinofílica – associada à disfunção ventricular	I	B
Na presença de miocardite positiva com pesquisa viral negativa, comprovada por biópsia endomiocárdica, em pacientes com insuficiência cardíaca crônica, com objetivo de melhora clínica e da função ventricular	IIa	B
Na insuficiência cardíaca aguda não responsiva à terapêutica usual	III	C

### 5.3. Antivirais: Indicações e Tipos

O prognóstico da cardiomiopatia inflamatória/miocardite é afetado negativamente pela persistência do vírus. O curso da cardiomiopatia viral é para certos vírus intimamente associados ao curso espontâneo da infecção viral, pois a eliminação espontânea do vírus é acompanhada de melhora clínica, enquanto isso não se aplica a pacientes que desenvolvem persistência do vírus.^194 - 197^

Pacientes com genomas enterovirais e adenovirais em CEM devem ser tratados com interferon beta (IFN-ß) (4 milhões de unidades por via subcutânea a cada 48 horas na primeira semana, 8 milhões de unidades por via subcutânea a cada 48 horas a partir da segunda semana e por 6 meses). Pode ser demonstrado em um estudo não randomizado que a administração de IFN-ß em pacientes positivos para EV e ADV induziu a eliminação do vírus, reduziu a lesão do miocárdio e melhorou significativamente a sobrevida a longo prazo.^198 , 199^ Em um estudo de fase 2 seguinte – betaferon em um teste de cardiomiopatia viral crônica (BICC) – 143 pacientes com sintomas de IC e confirmação por biópsia dos genomas de EV, ADV e/ou B19V foram aleatoriamente designados para tratamento duplo-cego e receberam placebo ou IFN-ß por 24 semanas, além do tratamento padrão da IC. Em comparação ao placebo, a eliminação e/ou redução da carga viral foram maiores nos grupos IFN-ß. O tratamento com IFN-ß foi associado a efeitos favoráveis na melhora da classe funcional da NYHA, qualidade de vida e avaliação global do paciente. Em análises retrospectivas, foi possível demonstrar que o tratamento com IFN-ß foi significativamente menos eficaz na eliminação da infecção por B19V.^171^

O herpes-vírus humano 6 foi detectado em alta prevalência no tecido miocárdico de pacientes que apresentaram sintomas de IC em um cenário clinicamente suspeito de miocardite. Curiosamente, o HHV-6 é capaz de integrar seu genoma em telômeros de cromossomos humanos, o que permite a transmissão do HHV-6 através da linha germinativa. O HHV-6 integrado no cromossomo (ciHHV-6) parece estar associado a um risco aumentado de miocardite e pode levar a um agravamento da IC. O HHV-6 também não é eliminado pelo IFN-ß, mas os sintomas de reativação e insuficiência cardíaca do HHV-6 diminuem após um período de tratamento de 6 meses com ganciclovir seguido de valganciclovir (ganciclovir 1.000 mg/24h por via intravenosa por 5 dias, depois valganciclovir 900 mg/24h ou 1.800 mg/24h por 6 meses) em pacientes sintomáticos com ciHHV6^200^ reativado (RNA mensageiro positivo).

A infecção por B19V do músculo cardíaco ainda é motivo de discussão. Os primeiros dados forneceram evidências de que inibidores antivirais da transcriptase reversa e análogos de nucleosídios como a telbivudina podem melhorar o resultado clínico de pacientes com DNA positivo de B19V e intermediários replicativos.^201^ No entanto, agora é necessário agendar um grande estudo clínico randomizado, controlado por placebo, para avaliar os resultados.

### 5.4. Imunomodulação (Imunoglobulina – Imunoadsorção): Indicações e Tipos de Imunoglobulinas

O racional para uso das imunoglobulinas intravenosas (IVIG) no tratamento das miocardites está na sua ampla capacidade de interagir com o sistema imune. São capazes de estimular o sistema complemento e células imunológicas a liberarem citocinas anti-inflamatórias e inibirem a liberação de citocinas pró-inflamatórias.^83^

As imunoglobulinas têm sido estudadas em diferentes cenários como na IC crônica;^202 , 203^ na cardiomiopatia dilatada;^166 , 204^ na cardiomiopatia periparto;^205^ na miocardite aguda,^164 , 165 , 206 , 207^ na miocardite fulminante^208^e nas miocardites virais.^167 , 169^

Embora alguns desses estudos apontem para potencial benefício do uso de imunoglobulina, estudo randomizado controlado em pacientes adultos com cardiomiopatia dilatada de início recente (<6 meses) ou miocardite não demonstrou benefício do uso de imunoglobulina em relação à função ventricular quando comparado ao grupo controle. Houve melhora da função ventricular e até mesmo normalização em 36% dos casos ao longo do seguimento, independentemente do grupo de tratamento. Vale destacar que não foi realizada pesquisa viral na biópsia dos pacientes, e apenas 16% tinham quadro de miocardite comprovado por presença de inflamação na biópsia.^166^

Em pacientes com miocardite aguda, os primeiros estudos apontavam para melhora da função ventricular e tendência a melhor prognóstico em 1 ano, com o uso de altas doses de IVIG.^164^ No entanto, revisão sistemática realizada em 2005, envolvendo 62 estudos, encontrou apenas um estudo randomizado controlado sobre o tema, e não demonstrou benefício do uso da terapêutica em pacientes com miocardite aguda, concluindo serem insuficientes as evidências para recomendação rotineira do uso de IVIG nesse cenário.^207^ Mais recentemente, estudo multicêntrico randomizado pequeno (41 pacientes) avaliou o prognóstico em curto prazo de pacientes com miocardite aguda ou cardiomiopatia de início recente, submetidos ao tratamento com IVIG comparado com pacientes que não receberam IVIG e revelou melhor sobrevida em curto prazo entre os pacientes que receberam IVIG sem diferença em relação à melhora da função ventricular que ocorreu nos dois grupos. No entanto, houve redução significativa de citocinas inflamatórias no grupo tratado. Tal estudo levanta a hipótese de potencial benefício das imunoglobulinas e sugere o mecanismo pelo qual tal benefício pode ser observado; no entanto, devido ao pequeno número de pacientes, o estudo não é suficiente para embasar a recomendação da terapêutica de forma irrestrita para pacientes com miocardite aguda.^209^

No entanto, nas miocardites virais, há dados de literatura que demonstram benefício do uso de imunoglobulina. Em estudo piloto em pacientes com miocardite por parvovírus B19, o uso de IVIG gerou redução significativa da carga viral e melhorou a função cardíaca.^167^ Em outra análise incluindo 152 pacientes com miocardite por adenovírus ou parvovírus B19, o uso de imunoglobulina também mostrou melhora da capacidade para o exercício; melhora da fração de ejeção de VE e melhora da classe funcional. Houve redução significativa da inflamação nos dois grupos de pacientes e redução expressiva da carga viral apenas entre os pacientes com miocardite por adenovírus; pacientes com parvovirose apresentaram persistência viral em torno de 40%.^169^ Esses dados sugerem potencial benefício de uso de imunoglobulina em pacientes com miocardite viral com comprovação por BEM.

Os dados atuais, embora não sejam suficientes para recomendação rotineira da terapêutica, apontam para potencial benefício da imunoglobulina em pacientes com miocardite com inflamação comprovada por biópsia, especialmente nas miocardites virais por adenovírus e parvovírus B19.

#### 5.4.1. Imunoadsorção

A patogênese da progressão para disfunção ventricular na cardiomiopatia dilatada envolve processos inflamatórios que podem ser identificados e quantificados por métodos imuno-histoquímicos, o que sugere relação causal entre miocardite e cardiomiopatia.^210^ A presença de linfócitos, células mononucleares e aumento da expressão gênica de antígenos HLA é frequente, assim como anticorpos contra proteínas mitocondriais e de contratilidade; receptores B1 e receptores muscarínicos também têm sido descritos na cardiomiopatia dilatada.^211 - 214^

A extração desses anticorpos cardíacos é possível por imunoadsorção, e alguns estudos têm testado a eficácia dessa metodologia no tratamento de pacientes com cardiomiopatia dilatada/miocardite.^215 , 216^ Em estudo controlado pequeno, 25 pacientes foram randomizados para realizar imunoadsorção seguida de substituição por IgG ou manter tratamento padrão sendo observada redução significativa de inflamação miocárdica (células CD3; linfócitos CD4 e CD8, além de reduzir a expressão antígenos HLA classe II) no grupo tratado.^217^ Em outros estudos pequenos randomizados, observa-se melhora da hemodinâmica e da função ventricular.^216^

Dados atuais sugerem que imunoadsorção pode ser uma abordagem terapêutica nova e promissora para pacientes com cardiomiopatia dilatada e presença de anticorpos cardíacos. Contudo, até o momento, as evidências baseiam-se em estudos pequenos não controlados ou estudos controlados abertos comparados à terapia convencional, que precisam ter seus resultados confirmados por grandes estudos multicêntricos prospectivos randomizados.^218^ No momento, está em andamento um estudo multicêntrico duplo-cego placebo-controlado que tem por objetivo avaliar os efeitos da imunoadsorção seguida de substituição por IgG em pacientes com cardiomiopatia dilatada.^219^ Apenas após os resultados deste grande estudo poderemos estabelecer grau de recomendação para essa terapêutica no contexto da cardiomiopatia dilatada/miocardite.

## 5.5. Terapêutica Cardioprotetora Convencional

### 5.5.1. Sem Disfunção Ventricular

A abordagem terapêutica dos pacientes com miocardite com função ventricular preservada tem como objetivo a prevenção do desenvolvimento de disfunção ventricular ou de arritmias malignas. Nos pacientes com suspeita diagnóstica e risco intermediário, podemos utilizar betabloqueadores e inibidores da enzima conversora de angiotensina (IECA) ou bloqueadores dos receptores de angiotensina (BRA) pelo período mínimo de 12 meses, com objetivos de redução da mortalidade e morbidade. A decisão de manutenção da terapêutica além desse período será de acordo com a avaliação da função ventricular e potencial arritmogênico. Como não foram realizados ensaios clínicos em pacientes com esse perfil de miocardite, o manuseio do tratamento deve seguir as orientações da diretriz de insuficiência cardíaca crônica e aguda pela SBC.

### 5.5.2. Com Disfunção Ventricular Hemodinâmica Estável

O manejo terapêutico da disfunção ventricular na miocardite deve estar alinhado com as diretrizes atuais de IC.^55 , 220 , 221^ As medicações recomendadas para todos os pacientes com disfunção ventricular sintomática e hemodinamicamente estáveis, como terapia cardioprotetora, salvo contraindicações, são conhecidas como terapia tripla – IECA ou BRA, betabloqueadores e antagonistas dos receptores mineralocorticoides. Os IECA/BRA e betabloqueadores podem ser iniciados em todos os indivíduos com ICFER mesmo que assintomáticos, salvo contraindicações, e devem ser mantidos quando ocorre normalização da função ventricular. A espironolactona, representante dos antagonistas de receptores mineralocorticoides no Brasil, deve ser iniciada quando o paciente já está em uso das demais medicações, mantendo sintomas (CF NYHA II-IV), devendo ser evitada em pacientes com creatinina >2,5 mg/dL ou com hipercalemia persistente ( Tabela 18 ).

**Tabela 18:** – Recomendações de medidas farmacológicas gerais na miocardite

Indicações	Classe	Nível de evidência
Tratamento com medicações modificadoras de prognóstico para pacientes com disfunção sistólica de ventrículo esquerdo, sintomáticos ou assintomáticos, de acordo com diretriz de insuficiência cardíaca vigente	I	C
Manutenção de uso de terapia com bloqueio neuro-hormonal após normalização da função ventricular	I	C
Considerar o uso de medicação com ação sobre bloqueio neuro-hormonal em pacientes com evidência de fibrose miocárdica sem disfunção	IIa	C

### 5.5.3. Paciente com Disfunção Ventricular e Hemodinâmica Instável: Abordagem Terapêutica

Pacientes com miocardite aguda e presença de disfunção ventricular sistólica podem apresentar-se em distintos modelos clínicos. Assim como a resposta clínica à terapêutica é bastante variável, podendo ou não haver manifestação clara de baixo débito clínico ou evidência de hipervolemia sistêmica. O uso de inotrópicos se justifica, pelo menos em três situações: no contexto claro de baixo débito, em síndrome cardiorrenal em refratariedade à otimização diurética e na presença de SVO2 abaixo de 60% com critérios hemodinâmicos invasivos de baixo débito. Conforme a dinâmica do cuidado, deve-se discutir o monitoramento invasivo para os pacientes sem resposta clara à essa terapia ( Tabela 19 ).^222 - 225^

**Tabela 19:** – Inotrópicos utilizados na miocardite com disfunção ventricular e hemodinâmica instável222-225

	Dobutamina	Milrinona	Levosimendana
**Prática clínica em miocardite**	Beta-agonista de ação seletiva B1, que promove o inotropismo por estimulação direta de betarreceptores.	Estudos experimentais em modelos murinos sugerem efeitos protetores relacionados à vasodilatação em miocardite da milrinona e levosimendana em detrimento da dobutamina. Age como um inibidor de fosfodiesterase em qualquer dose, aumentando a concentração de cálcio no cardiomiócito. A vasodilatação sistêmica contribui para o incremento no resultado de aumento de débito cardíaco.	Existem estudos experimentais em modelos murinos demonstrando a redução de apoptose celular e citocinas inflamatórias no uso de levosimendana em miocardite aguda. Entretanto, não há evidência robusta que recomende sua utilização como cardioprotetora em pacientes com miocardite ou seu benefício clínico frente a outros inotrópicos. Funciona como um sensibilizador do cálcio até 0,2mcg/kg/min; em doses maiores, funciona como um inibidor de fosfodiesterase, sem uso clínico testado. Não há evidência clínica de seu uso contínuo por mais de 48 horas.
**Inotropismo**	Moderado	Importante	Importante
**Vasodilatação**	Leve	Moderada a importante	Moderada a importante
**Aumento de débito cardíaco**	Pequeno a moderado	Importante e associado à vasodilatação	Importante
**Risco de hipotensão**	Pequeno	Importante e dose-dependente, assim como maior em pacientes com disfunção renal estabelecida	Importante, principalmente em caso de bolus realizado. Aumenta conforme o aumento da dose.
**Risco de arritmias**	Aumenta exponencialmente quando maior que 10mcg/kg/min	Aumenta em caso de dose de *bolus* (não recomendada)	Aumenta em caso de *bolus* inicial, também dose-dependente mais comum na dose de 0,2mcg/kg/min

## 5.6. Cuidados Gerais: Atividade Física e Vacinação

A miocardite é uma importante causa de morte súbita em atletas, podendo ocorrer tanto na sua fase aguda como na fase crônica. Está relacionada não só ao grau de inflamação do miocárdio, mas também à deflagração de arritmias complexas e ao desenvolvimento de disfunção ventricular esquerda.^226 - 228^

Atletas competitivos ou recreacionais portadores de miocardite ativa não devem praticar esportes competitivos ou exercícios físicos de alta intensidade até o término do período de convalescença. Não há consenso sobre esste período. Até recentemente, era estabelecido um período de, no mínimo, 6 meses após o início das manifestações clínicas. Atualmente, alguns especialistas já recomendam períodos menores, como de 3 meses, para a liberação de treinamentos e competições, dependendo da presença de sintomas, arritmias, disfunção ventricular, marcadores inflamatórios e alterações no ECG^12 , 229^ ( Tabela 20 ).

**Tabela 20:** – Recomendações de exercício físico na miocardite para atletas e não atletas12,229

Indicações	Classe	Nível de evidência
Os atletas podem retornar aos treinos e competições, e os não atletas, às suas atividades físicas habituais, após 3 a 6 meses da miocardite, apenas se todos os critérios a seguir forem preenchidos: – Função sistólica de VE na faixa de normalidade – Biomarcadores de lesão miocárdica normais – Ausência de arritmias no *Holter* de 24h e no teste ergométrico	IIa	C
Com miocardite prévia, devem ser reavaliados periodicamente, especialmente nos primeiros 2 anos, pelo risco aumentado de recorrência e progressão silenciosa da doença.	IIa	C
Considera-se o retorno a atividades competitivas em atletas e não atletas assintomáticos, no período de 3 a 6 meses após o quadro miocardite, com realce tardio persistente na RMC se função de VE normal e ausência de arritmias no *Holter* de 24h e teste ergométrico, devendo ser seguido periodicamente pelo risco potencial de taquiarritmias. Na presença de realce tardio positivo na RMC, devem ser avaliados anualmente.	IIa	C

*RMC: ressonância magnética cardíaca; VE: ventrículo esquerdo.*

O Consenso Europeu de Reabilitação Cardíaca e Prevenção recomenda que, nos pacientes portadores de IC, incluindo os indivíduos com miocardite, a prática de exercícios físicos deve ser de moderada intensidade (até 50% do VO2 pico ou 60% da frequência cardíaca máxima prevista), desde que não haja evidência laboratorial de inflamação ou arritmias.^230^

**Figura 6:**
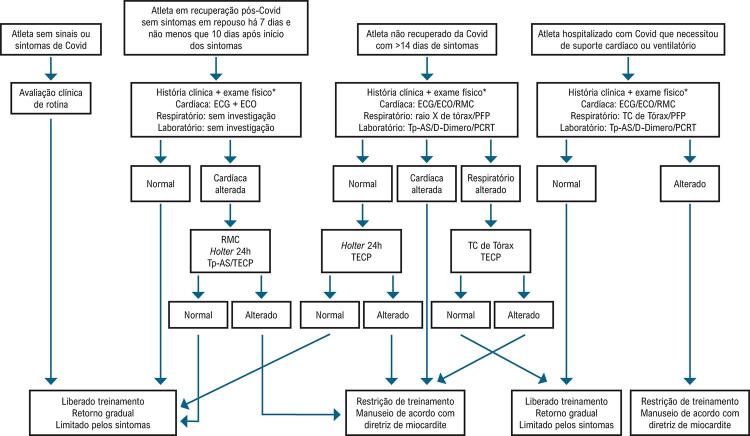
- Fluxograma de retomada de exercício para altas pós-Covid-19.

Em função da pandemia pela Covid-19, os atletas profissionais necessitaram interromper ou postergar suas atividades profissionais pelo risco de contaminação. Com abrandamento das medidas de afastamento, temos o questionamento de como os atletas poderão retornar suas atividades de forma segura. Os atletas que foram acometidos pela Covid-19 podem vir a apresentar sintomas respiratórios, fadiga muscular e risco de eventos trombóticos. Em decorrências de tais riscos, um fluxograma com recomendações de avaliação clínica e de liberação de atividades tem o objetivo de fornecer um guia para retomada das atividades físicas ( Figura 6 ).^231^

*História e exame físico na pesquisa de complicações pós-Covid: neurológico, gastrointestinal e dermatológico. ECG: eletrocardiograma; ECO: ecocardiograma transtoráxico; PCRT: proteína C reativa titulada; PFP: prova de função pulmonar; RMC: ressonância magnética cardíaca; TC de tórax: tomografia computadorizada de tórax; TECP: teste de exercício cardiopulmonar; Tp-AS: troponina de alta sensibilidade.

A vacinação segue as mesmas recomendações da imunização anual contra gripe e pneumococo feitas nos pacientes com IC e as demais vacinas disponíveis (caxumba, sarampo, rubéola, poliomielite). Não há evidências robustas de que estas predispõem a agudização ou o desenvolvimento de miocardite aguda para sobrepor os benefícios da imunização.^231 - 235^ O mesmo racional se aplica na vacinação para Covid-19. Para serem vacinados, os pacientes não podem estar na fase aguda da miocardite, sendo o mais aconselhável cerca de 3 meses após o diagnóstico de miocardite ( Tabela 21 ).

**Tabela 21:** – Recomendações de vacinação na miocardite

	Classe	Nível de evidência
Vacina contra gripe, pneumococo, caxumba, sarampo, rubéola, poliomielite e para Covid-19. Os pacientes não devem estar na fase aguda de doença, sendo recomendado com mais de 3 meses do início da suspeita diagnóstica.	I	C

## 6. Situações Especiais

### 6.1. Miocardite Fulminante

Miocardite fulminante pode ser definida contempora- neamente de forma pragmática, contemplando uma visão predominantemente clínica, independentemente de achados histológicos, em que existe: 1) apresentação clínica de sintomas graves de IC inferior a 30 dias; 2) instabilidade hemodinâmica com choque cardiogênico e arritmias com risco de vida (incluindo parada cardiorrespiratória recuperada ou abortada); e 3) necessidade de suporte hemodinâmico (inotrópicos ou assistência circulatória mecânica).^236^ Além dos exames já citados recomendados em casos de miocardite, o uso da BEM na miocardite fulminante é recomendado, sendo usualmente positivo, demonstrando múltiplos focos inflamatórios, possibilitando caracterização histológica do tipo de miocardite em curso.^237^ O curso clínico da miocardite fulminante é usualmente mais sombrio que outros tipos de miocardite não fulminantes, com menor chance de recuperação da função ventricular, maior mortalidade e maior chance de transplante cardíaco.^236 , 238^

#### 6.1.1. Avaliação Diagnóstica

O diagnóstico de miocardite fulminante envolve os critérios diagnósticos de miocardite *per se* envolvendo quadro clínico de IC aguda, elevação de troponinas e de marcadores inflamatórios, alterações inespecíficas no ECG, como inversões de onda T e/ou alterações de segmento ST, e alteração aguda da função ventricular. No cenário de choque cardiogênico, cateterismo cardíaco direito e angiografia coronária são essenciais para orientar o manejo. A ecocardiografia é ferramenta central no diagnóstico, uma vez que os pacientes com miocardite fulminante frequentemente não apresentam condições para submeterem-se à RM. Os achados ecocardiográficos são altamente dependentes da forma e do tempo de apresentação do paciente. Os pacientes com miocardite fulminante, em geral, apresentam dimensões diastólicas normais, mas aumento na espessura septal na apresentação, enquanto pacientes com miocardite viral aguda (não fulminante) podem apresentar-se com dimensões diastólicas tanto normal quanto aumentadas, mas espessura septal normal, consistente com outras formas de miocardiopatia dilatada.^15 , 64 , 72 , 98 , 239 , 240^

A decisão de realizar uma BEM no momento do cateterismo cardíaco está conforme as da força-tarefa de 2013 da ESC^15^ A BEM pode ser considerada o procedimento diagnóstico inicial quando a RM não é possível (p. ex., choque, presença de dispositivos de metal), se operadores experientes e patologistas cardíacos estão disponíveis. De acordo com as diretrizes, portanto, as indicações para BEM estariam presentes para a maioria dos pacientes com miocardite fulminante ( Figura 4 ). Mais precisão pode ser alcançada quando adicionados análise do genoma viral, imuno-histologia ou biomarcadores transcriptômicos se houver incerteza diagnóstica apesar da histologia.

Além da confirmação diagnóstica, a realização de BEM na miocardite fulminante pode ser decisiva para definição terapêutica. A avaliação imuno-histoquímica tem sido considerada obrigatória em função das conhecidas limitações diagnósticas dos critérios de Dallas, principalmente variabilidade interobservador, que, estima-se, vem trazer confirmação diagnóstica em, no máximo, 20% dos casos.^15 , 64 , 72 , 239 , 240^ De acordo com definição da OMS, para diagnóstico de miocardite ativa, é necessária a detecção imuno-histoquímica de infiltrados mononucleares (linfócitos T ou macrófagos) usando um ponto de corte de mais de 14 células/mm^2^, em adição à expressão aumentada de moléculas HLA classe II.^146^

A detecção de genoma viral nos espécimes da biópsia é factível (ainda pouco disponível no Brasil) e, quando acoplada à análise imuno-histoquímica, aumenta a acurácia diagnóstica, além de prover a etiologia e informação prognóstica.

Para miocardites fulminantes, a indicação classe I, nível de evidência C, já era considerada mesmo quando levava-se em conta apenas a análise histológica (critérios de Dallas). A análise histológica convencional, amplamente disponível, permite diagnósticos etiológicos que podem levar a mudanças de condutas terapêutica e a tratamentos específicos, como em miocardites eosinofílicas necrotizantes, miocardites de células gigantes, sarcoidose, amiloidose e miocardites associadas a doenças autoimunes conhecidas.

#### 6.1.2. Abordagem Terapêutica

Do ponto de vista do tratamento específico da miocardite, o reconhecimento do fator causal por meio da investigação histológica por BEM permite o estabelecimento de estratégias terapêuticas específicas, como a utilização de imunoglobulina nas miocardites virais e imunossupressão nas autoimunes sem presença viral, ou o uso de corticosteroide em pacientes com sarcoidose, miocardite eosinofílica necrotizante ou miocardite por células gigantes. Um ensaio clínico randomizado de imunossupressão em 85 pacientes com miocardite com comprovada ausência de persistência viral (TIMIC Study) demonstrou claro benefício sobre a fração de ejeção desses pacientes. No entanto, tratavam-se de pacientes com mais de 6 meses de diagnóstico e comprovada ausência de vírus.^162^ Ensaios clínicos de imunossupressão em pacientes com miocardite fulminante não existem. Uma opção que tem sido testada é a utilização de altas doses de imunoglobulina, a qual se mostrou benéfica sobre a função ventricular e classe funcional e demonstrou benefício em sobrevida;^208 , 209 , 217^ embora tenha sido demonstrado em um ensaio clínico com 62 pacientes, em que apenas 16% tinham miocardite comprovada por biópsia a ausencia de benefício.^166^

**Figura 7:**
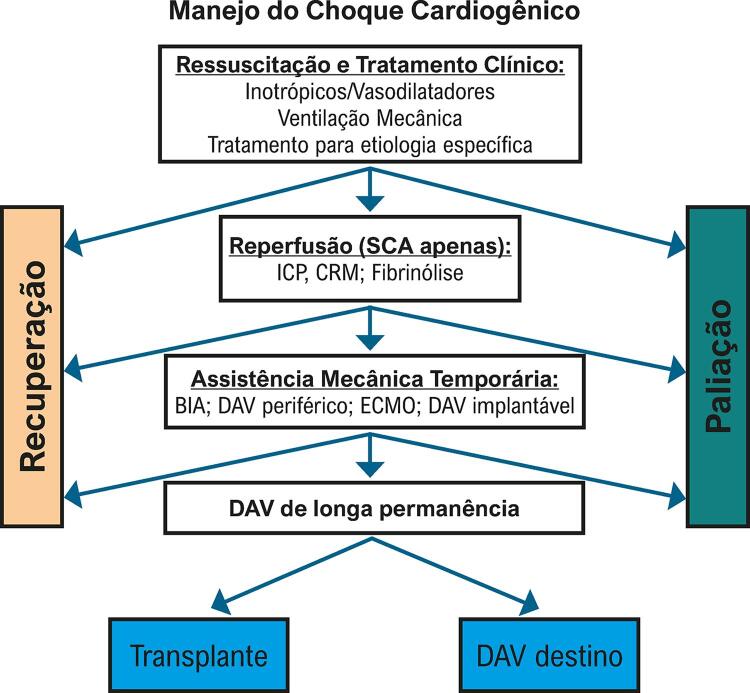
– Abordagem para estabilização inicial de pacientes com choque cardiogênico.

BIA: balão intra-aórtico; CRM: cirurgia de revascularização miocárdica; DAV: dispositivo de assistência ventricular; ECMO: extracorporeal membrane oxigentaion; ICP: intervenção coronariana percutânea; SCA: síndrome coronariana aguda. Adaptada de Kociol et al.^63^

O tratamento de suporte deve ser realizado com fármacos vasoativos e eventualmente vasopressores e em situações nas quais seja possível a introdução de vasodilatadores. O insucesso imediato no tratamento medicamentoso e acerto volêmico deve abrir perspectiva para indicação de suporte hemodinâmico com assistência circulatória. Os dispositivos mais utilizados são balão intra-aórtico, dispositivos percutâneos como *tandem-heart* e *impella* , circulação extracorpórea (ECMO) e ventrículos artificiais paracorpóreos, como ponte para recuperação ou ponte para transplante cardíaco ( Figura 7 ). Os dispositivos de curta duração têm sua indicação para suporte de 7 a 10 dias.^241^ Após esse período e quando não se consegue a estabilização do paciente, a indicação de ECMO ou ventrículos artificiais pode dar suporte por período maior, possibilitando mais chance de recuperação da disfunção ventricular^242^ (ver seção *Choque cardiogênico* ).

## 6.2. Sarcoidose

### 6.2.1. Diagnóstico

A sarcoidose é uma doença inflamatória granulomatosa, de etiologia desconhecida, caracterizada por granulomas não caseosos, podendo acometer vários órgãos, especialmente: pulmões (90%), pele, linfonodos, sistema nervoso central, olhos, fígado, coração e outros órgãos.^243^ Embora a sarcoidose cardíaca clinicamente manifesta só ocorra em 5% a 10% dos pacientes com sarcoidose, estudos em autópsias revelaram que o envolvimento cardíaco está presente em 20% a 30% de estudos com imagem cardíaca avançada; com o uso de CMR ou PET, demonstraram valores de 40% de comprometimento cardíaco.^244 - 246^ Além das diferenças de definições para ela, outro fator que parece impactar no aumento da prevalência dessa doença é o aprimoramento dos métodos de imagem.

**Figura 8:**
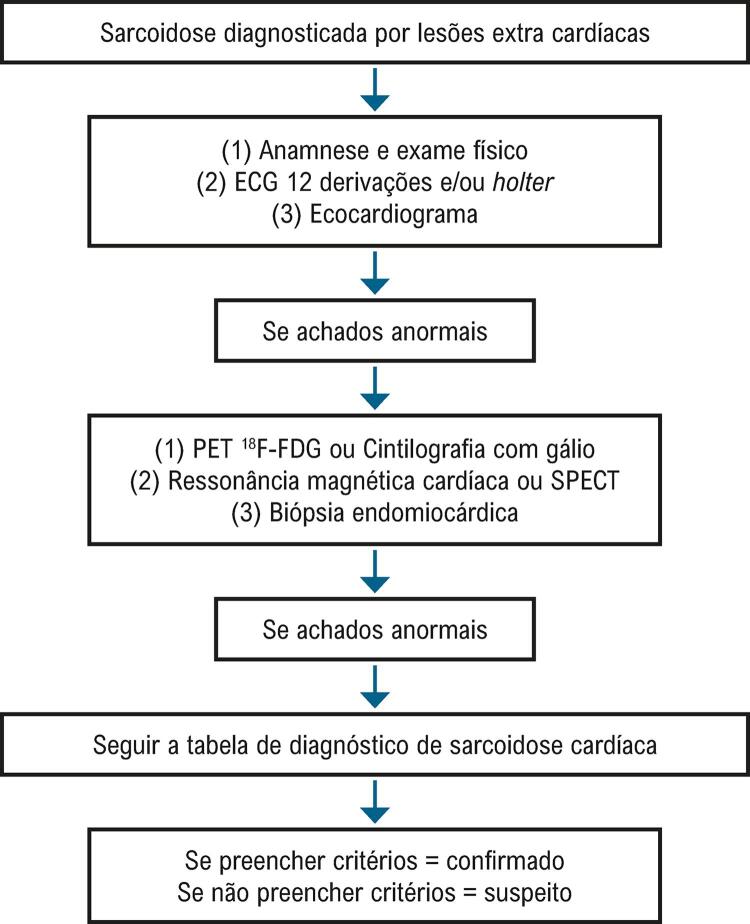
– Fluxograma diagnóstico de sarcoidose cardíaca, após o diagnóstico de lesões extracardíacas de sarcoidose.

ECG: eletrocardiograma; PET ^18^F-FDG: tomografia por emissão de pósitrons ^18^F-fluorodesoxiglicose; SPECT: tomografia computadorizada por emissão de fotón único. Adaptada de Terasaki et al.^247^

Atualmente, preconiza-se o uso das diretrizes da Sociedade Japonesa de Circulação (SJC) lançada em 2019 ( Tabela 22 , Figuras 8 e 9 ). Dentre as mudanças sugeridas neste documento, temos que o acúmulo anormalmente alto de marcadores no coração com tomografia por emissão de pósitrons por^ 18^F-fluorodesoxiglucose (FDG)/tomografia computadorizada (FDG-PET/CT), que foi categorizado nas ‘’Diretrizes para o diagnóstico de envolvimento cardíaco em pacientes com sarcoidose’’, em 2006, foi promovido para os critérios maiores, bem como o realce tardio por gadolínio do miocárdio na RM com gadolínio. Nas atuais diretrizes da SJC, o paciente também é diagnosticado clinicamente com sarcoidose cardíaca quando demonstra achados clínicos fortemente sugestivos de comprometimento cardíaco e de sarcoidose pulmonar ou oftalmológica somados a, ao menos, dois dos cinco achados laboratoriais característicos da sarcoidose. Por fim, a definição da sarcoidose cardíaca isolada foi elaborada pela primeira vez.

**Tabela 22:** – Recomendações da SJC para o diagnóstico da sarcoidose cardíaca247

Critérios para envolvimento cardíaco
Os achados cardíacos devem ser avaliados com base nos critérios maiores e nos menores. Achados clínicos que satisfazem os seguintes 1) ou 2) sugerem fortemente a presença de comprometimento cardíaco.
1. Dois ou mais dos cinco principais (a) a (e) são atendidos.
2. Um dos cinco critérios principais (a) a (e) somados a dois ou mais critérios menores (f) a (h) são atendidos.
**Critérios maiores**
a. Bloqueio atrioventricular de alto grau (incluindo bloqueio atrioventricular completo) ou arritmia ventricular fatal (p. ex., taquicardia ventricular sustentada e fibrilação ventricular)
b. Afinamento basal do septo ventricular ou anatomia anormal da parede ventricular (aneurisma ventricular, afinamento do septo ventricular superior ou médio, espessamento da parede ventricular regional)
c. Disfunção contrátil do ventrículo esquerdo (fração de ejeção do ventrículo esquerdo inferior a 50% ou assinergia da parede ventricular focal)
d. Cintilografia com citrato ^67^Ga ou PET ^18^F-FDG revela acúmulo anormalmente alto de marcadores no coração
e. A RM com gadolínio revela atraso no contraste do miocárdio
**Critérios menores**
f. Achados anormais de ECG: arritmias ventriculares (taquicardia ventricular não sustentada, multifocais ou frequentes contrações ventriculares prematuras), desvio do eixo ou ondas Q anormais
g. Defeitos de perfusão na cintilografia de perfusão miocárdica
h. Biópsia endomiocárdica: infiltração de monócitos e fibrose intersticial miocárdica moderada ou grave. Diretrizes para o diagnóstico de sarcoidose cardíaca
**Diretrizes para o diagnóstico de sarcoidose cardíaca**
1. Grupo de diagnóstico histológico (aqueles com achados positivos na biópsia do miocárdio): a sarcoidose cardíaca é diagnosticada histologicamente quando a biópsia endomiocárdica ou as amostras cirúrgicas demonstram granulomas não caseosos.
2. Grupo de diagnóstico clínico (aquele com achados negativos da biópsia do miocárdio ou aqueles que não foram submetidos à biópsia do miocárdio): o paciente é diagnosticado clinicamente como sarcoidose cardíaca (1) quando granulomas não caseosos são encontrados em outros órgãos que não o coração, e achados clínicos fortemente sugestivos de comprometimento cardíaco anteriormente mencionado estão presentes; ou (2) quando o paciente demonstra no quadro clínico achados fortemente sugestivos de sarcoidose pulmonar ou oftálmica; pelo menos dois dos cinco achados característicos laboratoriais de sarcoidose (linfadenopatia hilar bilateral, atividade sérica alta de ECA ou níveis séricos elevados de lisozima, sIL-2R sério alto, acúmulo significativo de marcadores na cintilografia com citrato ^67^Ga ou PET ^18^F-FDG, alta porcentagem de linfócitos CD4/CD8, razão >3,5 no líquido do LBA). Os achados de imagem sugerem fortemente o envolvimento cardíaco anteriormente mencionado.
**Diretrizes de diagnóstico para sarcoidose cardíaca isolada**
Pré-requisitos
1. Não serem observadas características clínicas da sarcoidose em outros órgãos além do coração (o paciente deve ser examinado detalhadamente para avaliar envolvimentos respiratórios, oftalmológicos e cutâneos de sarcoidose. Quando o paciente é sintomático, outras etiologias que podem afetar os órgãos correspondentes devem ser descartadas.
2. A cintilografia com ^67^Ga ou o PET ^18^F-FDG não revela acúmulo anormal de marcadores em nenhum outro órgão que não o coração.
3. A tomografia computadorizada do tórax não demonstra nos pulmões ou linfadenopatia hilar e mediastinal (eixo menor >10 mm).
**Grupo de diagnóstico histológico**
1. A sarcoidose cardíaca isolada é diagnosticada histologicamente quando a biópsia endomiocárdica ou as amostras cirúrgicas demonstram granuloma não caseoso.
**Grupo de diagnóstico clínico**
1. A sarcoidose cardíaca isolada é diagnosticada clinicamente quando o critério (d) e pelo menos três outros critérios maiores (a) a (e) são satisfeitos. Quando o paciente atende a pelo menos quatro critérios de envolvimento cardíaco que não incluam o critério (d) ou quando o paciente atende os critérios (b) e (d) mais um dos critérios restantes, suspeita-se que o paciente tenha sarcoidose cardíaca isolada.

*ECA: enzima conversora de angiotensina; ECG: eletrocardiograma; LBA: lavado broncoalveolar; PET ^18^F-FDG: tomografia por emissão de pósitrons ^18^F-fluorodesoxiglicose; RM: ressonância magnética. Adaptada de Terasaki et al.^247^*

**Figura 9:**
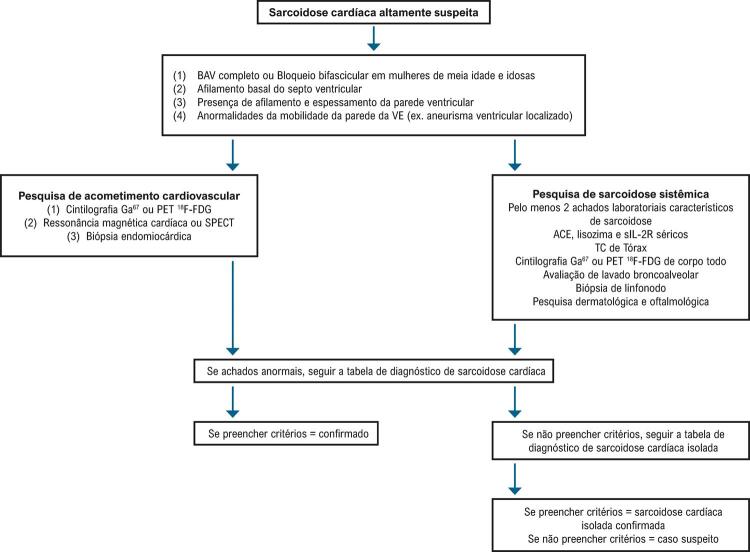
– Fluxograma de diagnóstico para sarcoidose cardíaca em pacientes que apresentam manifestações cardíacas e são fortemente suspeitos de sarcoidose cardíaca. BAV: bloqueio atrioventricular; ECA: enzima de conversão da angiotensina; FDG: fluorodesoxiglicose; PET 18F-FDG: tomografia por emissão de pósitrons 18F-fluorodesoxiglicose; SPECT: tomografia computadorizada por emissão de fóton único; TC: tomografia computadorizada;VE: ventrículo esquerdo. Adaptada de Terasaki et al.247

### 6.2.2. Tratamento e Prognóstico

O tratamento imunossupressor da sarcoidose cardíaca baseia-se na experiência clínica e na opinião de especialistas em que faltam estudos randomizados. O objetivo do tratamento é reduzir a atividade inflamatória e a prevenção de fibrose e deve ser guiado pela magnitude do processo inflamatório e o grau de acometimento miocárdico.^248^

Recomenda-se o tratamento imunossupressor nas seguintes situações: nos casos de disfunção ventricular esquerda, arritmias ventriculares, atividade hipermetabólica no PET-FDG, distúrbios de condução, realce tardio na RMC ou disfunção de ventrículo direito na ausência de hipertensão pulmonar.^248 - 250^

Existem três linhas de tratamento na sarcoidose – primeira linha: corticosteroides; segunda linha: metotrexato e azatioprina nos casos intolerantes ou uso crônico de corticosteroides; e terceira linha: anticorpos anti-TNF (infliximab e andalimumab) nos casos de falha de tratamentos anteriores.^251^

O fármaco de escolha é o corticosteroide. Em uma revisão sistemática do uso de corticosteroide em pacientes com distúrbios de condução ventricular, 27 de 57 pacientes (47,4%) melhoraram após tratamento.^252^ No entanto, em vista da não previsibilidade de resposta, esses pacientes com distúrbios de condução e sarcoidose cardíaca devem receber um marca-passo ou cardiodesfibrilador implantável.^119 , 253^

Estudos mais antigos que avaliaram o efeito do corticosteroide na função ventricular sugerem preservação da função ventricular nos casos de função normal ao diagnóstico, melhora da fração de ejeção ventricular nos casos de pacientes com disfunção leve a moderada e não melhora nos casos de disfunção ventricular importante.^119^ No entanto, por outro lado, um estudo finlandês sugere uma melhora da função ventricular esquerda com o tratamento imunossupressor nos casos de função ventricular severamente comprometida (FEVE<35%), mas sem alterações nos casos de função normal ou moderamente diminuída no início do tratamento. Talvez tais diferenças estejam no diagnóstico e tratamentos precoces.^254^

Nos casos de arritmia ventricular, os estudos são mais limitados; no entanto, a causa da arritmia parece ser secundária a cicatrizes e, talvez, o efeito do corticosteroide nesses pacientes seja pequeno benefício.^255^ A ablação por cateter nos casos de taquicardia ventricular pode ser considerada após o implante de cardiodesfibrilador implantável ou falência das medicações antiarrítmicas.^256^

**Figura 10:**
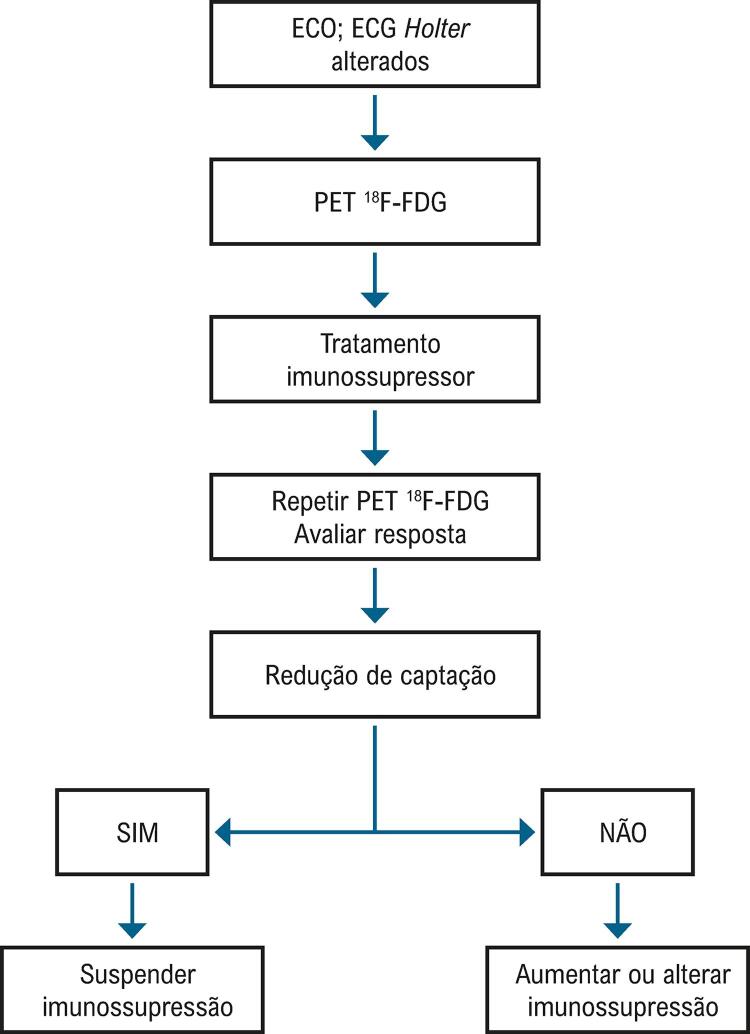
– Algoritmo de tratamento da sarcoidose.

ECG: eletrocardiograma; ECO: ecocardiograma; PET ^18^F-FDG: tomografia por emissão de pósitrons ^18^F-fluorodesoxiglicose.

O algoritmo de tratamento ( Figura 10 ) sugerido seria de doses iniciais de prednisona (30 mg/dia a 40 mg/dia) seguido da repetição do PET entre 4 a 6 meses, com o objetivo de avaliar a atividade da doença e guiar o tratamento farmacológico subsequente.

Yokoyama et al.^257^ compararam o uso de PET^18^F-FDG/CT antes e após utilização de corticosteroide em 18 pacientes com sarcoidose cardíaca, e observaram que a SUV max diminuiu significativamente em comparação com valores basais. Estudo recente utilizou o PET ^18^F-FDG/CT para diagnóstico e tratamento da sarcoidose cardíaca com doses baixas de corticosteroide e controle da doença em 1 ano do diagnóstico.^258^

Medicamentos imunossupressores outros que corticosteroide são necessários devido ao longo tempo do tratamento, e são indicados nos pacientes que necessitam de uma dose de manutenção de prednisona >10 mg/dia e que não toleram efeitos colaterais do corticosteroide.^248 , 250^

São sugeridos: metotrexato,^257^ azatioprina,^258^ ciclofosfamida^259^ e inibidores do fator de necrose tumoral.^260 , 261^ O tipo de fármaco utilizado pode ser determinado pelo tipo de acometimento extracardíaco; COMO evitar metotrexato, NO envolvimento hepático e estudos em pacientes com sarcoidose pulmonar, cutânea, ocular, neurológica e multissistêmica sugerem uma boa eficácia do infliximab ( Tabela 23 [Table t61] ).^262^

**Tabela 23:** – Recomendações de terapia imunossupressora na sarcoidose

Indicação	Classe	Nível de evidência
Prednisona 30 a 40mg/d por 4 a 6 meses	IIa	B
**Outros imunossupressores em caso de uso corticosteroide:**
Azatioprina 50 a 200 mg/d	IIb	C
Metotrexato 10 a 20mg/semana	IIb	C
Infliximab em sarcoidose pulmonar, cutânea, ocular, neurológica e multissistêmica	IIb	C
Leflunomida 10 a 20 mg/d	IIb	C

**Tabela 24 t61:** – Indicação de CDI na sarcoidose

Indicações	Classe	Nível de evidência
Taquicardia ventricular/morte cardíaca abortada	I	C
FEVE <35 % a despeito de tratamento otimizado e período de imunossupressão e inflamação ativa	I	C
Síncope inexplicada de provável causa arrítmica	IIa	C
Fração de ejeção entre 35% e 49% e/ou FEVD <40% a despeito de tratamento otimizado e imunossupressor e evidência pela ressonância ou PET de extensa cicatriz miocárdica	IIa	C
Fração de ejeção entre 35% e 49% e/ou FEVD <40% a despeito de tratamento otimizado e imunossupressor	IIb	C

*CDI: cardiodesfibrilador implantável; FEVD: fração de ejeção de ventrículo direito; FEVE: fração de ejeção de ventrículo esquerdo; PET: tomografia por emissão de pósitron.*

### 6.2.3. Prognóstico

A sarcoidose cardíaca tem um pior prognóstico quando comparada à miocardiopatia dilatada. Uma vez o coração estando acometido, o prognóstico torna desfavorável. O comprometimento cardíaco é responsável por 85% dos óbitos na doença.^183 , 243^

Kandolin et al.^256^ reportaram o efeito a longo prazo do tratamento imunossupressor na coorte finlandesa, e sobrevida livre de transplante em 1 ano, 5 anos e 10 anos foi 97%, 90% e 83%, respectivamente, durante o seguimento de 6,6 anos. Nesse estudo, a presença de IC e a função cardíaca antes do tratamento com corticosteroide foram os fatores mais importantes para estimativa do prognóstico, demonstrando que o tratamento precoce é importante.

A presença de realce tardio miocárdico avaliado pela RM aumentou em 30 vezes o risco de morte, morte súbita abortada ou implante de cardiodesfibrilador em um período de seguimento de 2,6 anos,^262^ posteriormente confirmados em metanálises. Sugere que o limiar de 20% de massa de fibrose esteja associado com risco de eventos.^263^

Em um estudo que utilizou PET, observou-se que 26% dos eventos adversos relatados, tais como taquicardia ventricular e morte, ocorreram nos casos de captação cardíaca ao PET em um seguimento de 1,5 ano. Por outro lado, a captação extracardíaca não se associou com eventos adversos no seguimento.^264^

Outro dado interessante é que pacientes com sarcoidose cardíaca isolada têm pior prognóstico quando comparados com pacientes com sarcoidose sistêmica com comprometimento cardíaco.^265^ Outro estudo finlandês observou elevada frequência de disfunção ventricular e anormalidades septais ao ecocardiograma e alta prevalência de realce tardio miocárdico pela ressonância e maior associação com sexo feminino e maior disfunção ventricular esquerda.^266^Nesse estudo, a presença de IC na apresentação, disfunção ventricular esquerda severa (<35%) e sarcoidose cardíaca isolada também esteve relacionada com o prognóstico.^254^

O ecocardiograma com Strain (GLS <17,3) foi preditor independente de mortalidade, IC, hospitalização, novas arritmias e desenvolvimento de sarcoidose cardíaca.^267^

Já biomarcadores séricos como BNP estiveram relacionados com desenvolvimento de IC, e a troponina, com desenvolvimento de arritmias fatais,^268^ menor fração de ejeção e pior prognóstico.^269^

## 6.3. Células Igantes

### 6.3.1. Tratamento

De acordo com Registro Internacional, a MCG é etiologia de 12% das miocardites fulminantes e 3,6% das miocardites não fulminantes.^242^ Os alvos do tratamento são limitados porque não são conhecidos adequadamente os mecanismos da MCG, embora um mecanismo autoimune envolvendo inflamação miocárdica mediada por linfócitos-T tenha sido proposto.^270 , 271^

A MCG tem um prognóstico pior que as miocardites eosinofílicas e linfocitárias e está mais frequentemente associada à IC, parada cardíaca, fibrilação e taquicardia ventricular, bloqueios ou simulação de IAM.^242 , 272^ Sem tratamento, a evolução geralmente é fatal, com morte até os 5,5 meses de evolução.^271^Mesmo com tratamento, a MCG tem alta mortalidade ou necessidade de indicação precoce de suporte mecânico circulatório e/ou transplante cardíaco.

Recentemente, foi descrita sobrevida livre de transplante aos 5 anos de 42%. Como importantes marcadores de prognóstico de morte precoce ou necessidade de suporte mecânico ou transplante cardíaco, foram descritos os níveis de troponina e moderada/severa necrose ou fibrose na BEM. Também são marcadores de prognóstico níveis elevados de BNP/nt-proBNP e redução importante de FEVE.^191^ O prognóstico reservado pode ser devido à lesão miocárdica ou recorrência da MCG.^273^ Após transplante cardíaco, também tem sido descrita recorrência da MCG.

O diagnóstico precoce é crítico e baseia-se nos resultados da BEM, ou análise histológica de coração explantado durante transplante cardíaco, ou de fragmento de miocárdio obtido durante implante de dispositivo de assistência ventricular.^270 , 274 , 275^ A sensibilidade da biópsia pode ser limitada pelo erro de amostragem. Fragmentos são obtidos preferencialmente da porção apical do septo do ventrículo direito, porque diminui o risco de complicações. Uma biópsia negativa não necessariamente exclui o diagnóstico de MCG. A sensibilidade da BEM aumentou de 68% para 93% depois de repetir o procedimento ( Tabela 25 ).

**Tabela 25:** – Recomendações de biópsia endomiocárdica (BEM) na avaliação diagnóstica na miocardite de células gigantes

Indicações	Classe	Nível de evidência
BEM ou análise de coração explantado durante transplante cardíaco, ou de fragmento miocárdico obtido durante implante de dispositivo de assistência mecânica em pacientes com quadro agudo de insuficiência cardíaca com grave comprometimento hemodinâmico ou fulminante	I	B
Suspeita de diagnóstico de miocardite associada com parada cardíaca, ou fibrilação ou taquicardia ventricular, ou bloqueios, ou simulação de infarto agudo do miocárdio	I	B

O tratamento da MCG pode ser dividido em tratamento da IC com FEVEr provocada pela lesão miocárdica ou recorrência da MCG, das arritmias, bloqueios e o tratamento do provável mecanismo com imunossupressores.

O tratamento da IC, dos distúrbios hemodinâmicos, bloqueios e arritmias segue as mesmas orientações do tratamento da IC segundo as Diretrizes da SBC, quer seja medicamentoso e/ou com inotrópicos, marca-passo/desfibriladores e/ou suporte mecânico circulatório e transplante cardíaco.^242^ O transplante cardíaco pode ter indicação mais precoce devido ao prognóstico reservado da MCG, mesmo com imunossupressores. A indicação de implante de cardiodesfibrilador pode ser feita para prevenção primária de morte súbita ou secundária com base na alta incidência de arritmias complexas e graves.^276^ Foi descrito que 59% dos pacientes com MCG apresentaram taquicardica ventricular sustentada ou choques para arritmia ventricular complexa, apesar de estarem livres de IC grave.

A indicação de imunossupressores está baseada em resultados de série de casos ou de pequenos estudos randomizados, e foram utilizadas medicações imunossupressoras como prednisona, ciclosporina, azatioprina, micofenolato, everolimus, sirolimus ou globulina de coelho, globulina antitimocitária ou soro muromonab-CD3 para citólise de linfócito T. Após o diagnóstico inicial, em geral, utilizam-se corticosteroides em altas doses e/ou globulina de coelho, globulina antitimocitária ou soro muromonab-CD3, podendo já associar medicação para imunossupressão crônica. O uso de hemoadsorção também tem sido relatado ( Tabela 26 ).^277^

**Tabela 26:** – Recomendações da terapêutica na miocardite de células gigantes

Indicações	Classe	Nível de evidência
Corticosteroide em doses altas em combinação com anticorpos antilinfocitários e/ou inibidores de calcineurina (ciclosporina ou tacrolimus) e/ou antiproliferativos (azatioprina ou microfenolato)	I	B
Imunossupressão de manutenção com corticosteroide e inibidor de calcineurina (ciclosporina ou tacrolimus) ou esquema tríplice acrescentando antiproliferativo (azatioprina ou micofenolato)	I	B
Transplante cardíaco	I	B
Indicação de cardiodesfibrilador para prevencão primária ou secundária de arritmias complexas ventriculares	I	B

Em geral a imunosssupressão de manutenção é baseada na ciclosporina em esquema duplo ou triplo.^270 , 278^ Entretanto, existem importantes limitações na avaliação do seu real benefício. Combinações das medicações prednisona, ciclosporina, azatioprina, micofenolato ou uso isolado ou combinado com RATG ou soro muromonab-CD3 têm sido feitos. Foi descrito que imunossupressão tripla pode aumentar a chance de estar vivo livre de transplante cardíaco para 58% aos 5 anos.^191^ Contudo, tem que ser mantida a imunossupressão pela possibilidade de haver recorrência. A imunossupressão combinada (prednisona, ciclosporina e azatioprina) parece ser mais aceita, embora outras combinações tenham sido utilizadas, tais como ciclosporina com RATG, ou RATG com corticosteroides em altas doses. Não existem estudos comparativos para confirmar a melhor imunossupressão.^191 , 274^ A utilização de ciclosporina associada a corticosteroides em altas dose ou muromonab-CD3 por 4 semanas diminui necrose, inflamação celular e células gigantes.^279^

Transplante cardíaco está indicado com melhora da sobrevida a médio prazo, mas pode haver recorrência de 20% a 25%.^8 , 280^ É o tratamento de escolha, apesar de maior risco de rejeição.^281^

### 6.3.2. Manifestação Clínica e Diagnóstico

A miocardite de células gigantes é reconhecida como uma doença rápida e progressiva, na maioria das vezes fatal, se o paciente não for submetido a transplante cardíaco. Em boa parte dos casos, é associada a processo autoimune.

Dados do Giant Cell Myocarditis Study Group mostraram uma incidência predominante em adultos jovens, brancos, sem predomínio de sexo e com manifestação principal de IC aguda (75% dos casos), porém metade dos pacientes desenvolveu arritmia ventricular complexa na evolução da doença. A sobrevida média livre de transplante cardíaco foi de 5,5 meses.^8^

Registro mais recente sobre miocardite de células gigantes mostrou incidência também em adultos jovens, mulheres, e as principais manifestações clínicas foram IC aguda, BAV e arritmias ventriculares.^274^

Exames de imagem não apresentam nenhuma alteração específica na miocardite de células gigantes. O diagnóstico baseia-se nos achados característicos da BEM com infiltrado inflamatório difuso e misto, constituído principalmente por macrófagos, seguido em quantidade por linfócitos e células gigantes multinucleadas derivadas de macrófagos, tipicamente dispersas, e, ainda, com menor representação de eosinófilos e células plasmáticas.^282^

## 6.4. Miocardite chagásica aguda e reagudização

### 6.4.1. Manifestações Clínicas e meios de Infecção, Reagudização nos Pacientes Imunossuprimidos

Nos últimos anos, a doença de Chagas aguda (DCA) vem apresentando aumento no número de casos tanto por transmissão oral ou vetorial quanto por quadros de reativação da doença em países da América Latina. Os principais meios de infecção da DCA, atualmente, são: transmissão oral (68,4%), vetorial (5,9%), vertical (0,5%), transfusional (0,4%), acidental (0,1%) e desconhecida (24,7%), como descrito em série de casos diagnosticados na Amazônia Brasileira.^19^

A transmissão vetorial ocorre pelo hábito de os triatomíneos defecarem durante ou logo após a hematofagia, com a deposição de fezes contaminadas fazendo com que as formas infectantes do *Trypanosoma cruzi* atinjam a pele, mucosas e, posteriormente, a corrente sanguínea. O período de incubação é de 4 a 15 dias. A transmissão oral ocorre quando há a ingestão de alimentos ou bebidas contaminadas com parasitos. Atualmente, é a causa mais comum da doença aguda, ocasionando surtos em regiões endêmicas e não endêmicas. Seu período de incubação varia de 3 a 22 dias.^283^

Os casos de DCA podem cursar com sinais e sintomas inespecíficos de síndrome infecciosa, tais como febre, mialgias, edema de face e artralgias; além de sinais relacionados com a porta de entrada como o chagoma de inoculação e sinal de Romaña na forma vetorial e quadros digestivos, podendo ocorrer hemorragias digestivas na forma oral. ^284^

Os casos agudos podem ou não cursar com miocardite e pericardite. Relatos de necrópsia mostram intensa inflamação aguda do epicárdico e miocárdio, observando-se atividade inflamatória intensa e difusa e dissociação extensa de fibras cardíacas, sendo observadas as formas amastigotas do parasita.^285^ Sinais e sintomas compatíveis com IC variaram de 26% a 58%. Podem ocorrer casos graves com tamponamento cardíaco e choque cardiogênico por disfunção sistólica de VE. A letalidade na forma de transmissão oral variou de 2% a 5% nas maiores séries. A presença de alterações cardíacas em exames complementares variou de 33% a 70% de alterações eletrocardiográficas (bloqueio de ramo direito, BAV de primeiro grau, fibrilação atrial aguda, bloqueio divisional anterossuperior) e de 13% a 52% de alterações ao ecocardiograma, com derrame pericárdico sendo a alteração mais frequente (10% a 82%), e alterações de contração segmentar, comuns na fase crônica, são pouco encontradas na fase aguda. Apesar da ocorrência de casos graves de comprometimento cardíaco, a maioria dos pacientes cursa com função sistólica preservada com poucos casos de redução da fração de ejeção, e a maioria dos óbitos ocorre devido à presença de derrame pericárdico importante e tamponamento cardíaco.^286 , 287^

### 6.4.2. Diagnóstico

Os exames parasitológicos diretos são os mais indicados para o diagnóstico da miocardite aguda.^288^ Métodos indiretos, como a hemocultura e o xenodiagnóstico, têm baixa sensibilidade, não sendo ideais para utilização na fase aguda. Os exames sorológicos não são os melhores métodos para diagnóstico na fase aguda, mas podem ser feitos quando os exames parasitológicos diretos forem persistentemente negativos e a suspeita clínica persistir.

A pesquisa a fresco do parasita no sangue circulante é rápida e simples, além de ser mais sensível que o esfregaço corado. A condição ideal de coleta é com o paciente ainda febril e dentro de 1 mês do início dos sintomas. Métodos de concentração (Strout, micro-hematócrito, creme leucocitário) são recomendados quando a pesquisa a fresco resultou negativa, por serem mais sensíveis. São empregados também quando o quadro clínico agudo começou há mais de 1 mês. Resultados negativos na primeira análise não devem ser considerados definitivos, principalmente se os sintomas persistirem, a não ser que outra etiologia seja comprovada.

A PCR, sendo um método de diagnóstico molecular, vem se tornando mais importante para detectar infecção recente, visto que mostra resultados positivos dias a semanas antes que sejam detectadas tripomastigotas circulantes.^289 - 291^ Pode ser feita em sangue periférico e no tecido obtido por BEM para detectar reativação precoce pós-transplante cardíaco, antes do aparecimento do quadro clínico ou de disfunção do enxerto.^292^

A reativação da doença de Chagas no período pós-transplante cardíaco pode acontecer em 19,6% a 45% dos casos.^293^ O quadro clínico pode ser de miocardite aguda, com vários graus de IC, frequentemente acompanhada de manifestações sistêmicas. Na pele, podem surgir eritema e nódulos subcutâneos, que devem ser biopsiados para pesquisa de ninhos de amastigotas. O monitoramento deve ser rotineiro, mesmo sem suspeita de reagudização. Quando não há sinais clínicos extracardíacos, a biópsia deve ser realizada.

### 6.4.3. Tratamento

O tratamento tripanosomicida está indicado nos pacientes com DCA com ou sem manifestações de miocardite e na reativação da doença crônica devido à imunossupressão (transplantados) ( Tabela 27 ).^294^

**Tabela 27:** – Recomendações para o tratamento etiológico na miocardite chagásica aguda

Indicações	Classe	Nível de evidência
Infecção aguda, independentemente do mecanismo de transmissão	I	C
Reativação de infecção crônica pelo *T. cruzi*	I	C

O benzonidazol é a droga disponível e recomendada para o tratamento da infeção pelo *T. cruzi* .^295^ As informações a respeito desse tema, no entanto, são escassas, baseadas em estudos não randomizados, com número de pacientes e tempo de observação insuficientes. Embora a definição sobre os critérios de cura da doença permaneça controversa, existe um consenso atual de que o tratamento com benzonidazol deve ser realizado nas formas agudas e que existe um provável benefício a longo prazo.^296^

A dose de benzonidazol em crianças é de 5 a 10mg/kg por dia, dividindo em duas tomadas, por 60 dias. Em adultos, a dose é de 5mg/kg. Reações adversas ocorrem em aproximadamente 30% dos pacientes, sendo as mais frequentes uma dermatite alérgica (30%) e uma neuropatia periférica sensitiva (10%).

## 6.5. Miocardite por Doenças Tropicais

As doenças tropicais são entidades infecciosas geralmente transmitidas por vetores e ocorrem nas regiões tropicais. Há pouca atenção dos governos, e os recursos destinados ao controle dessas doenças são escassos, com acometimento das populações vulneráveis em áreas com saneamento básico inadequado e sistemas de saúde deficitários. A Amazônia brasileira é região endêmica dessas doenças, muito embora outras regiões do país também sejam afetadas. Muitas das doenças tropicais causam miocardite e parecem contribuir para o aumento da carga das doenças cardíacas nos países em desenvolvimento.^297^ As doenças tropicais que causam miocardite e são prevalentes no Brasil são malária, dengue, Chikungunya, Zika e febre amarela ( Tabela 28 ). Essas doenças devem ser consideradas na investigação das miocardites que ocorrem em áreas endêmicas.

**Tabela 28:** – Características das principais causas de miocardites tropicais

	Agente	Vetor	Quadro clínico
**Malária**	*Plasmodium spp* (protozoário)	Mosquito *Anopheles*	**Forma leve:** febre, calafrios, cefaleia, mialgias e mal-estar **Forma grave:** choque, convulsões, confusão mental, insuficiência renal, síndrome da dificuldade respiratória aguda, coma e morte – Pode haver casos assintomáticos, especialmente em regiões endêmicas
**Dengue**	Vírus da dengue	Mosquito *Aedes aegypti*	**Forma leve:** febre, cefaleia, mialgia, artralgia, dor retro-orbital e erupção maculopapular, náusea, vômito **Forma grave (com sinais de alerta):** intensa dor abdominal, vômito persistente (≥ 3 vezes/24h), epistaxe, sangramento gengival, fadiga, inquietação ou irritação, hematêmese ou melena, alteração mental – Cerca de 50% dos casos apresentam sintomas
**Chikungunya**	Vírus da Chikungunya	Mosquito *Aedes aegypti*	**Forma leve:** febre, erupção cutânea, artralgia, mialgia, edema e cefaleia **Forma grave:** doença neurológica grave, miocardite e falência de múltiplos órgãos **Forma crônica:** persistência da artralgia e mialgia, associado a edema, principalmente nos pulsos, mãos, tornozelos e pés. Pode durar meses ou até anos e resultar em incapacitação – Cerca de 80% dos casos são sintomáticos
**Zika**	Vírus da Zika	Mosquito *Aedes aegypti*	**Forma leve:** febre (geralmente leve), erupção cutânea, artralgia, artrite, mialgia, dor de cabeça, conjuntivite e edema **Forma grave:** casos graves que requerem hospitalização são incomuns e fatalidades são raras **Forma congênita:** anomalias oculares, cardíacas e neurológicas, como a microcefalia (mais comum) – Apenas cerca de 20% dos casos apresentam sintomas
**Febre amarela**	Vírus da febre amarela	*Mosquitos Haemagogus* (silvestre) e *Aedes aegypti* (urbano)	**Sintomas leves:** febre de início súbito, calafrios, cefaleia, mialgia, fraqueza, fadiga, náuseas, vermelhidão ocular **Quadro grave (fase tóxica):** febre alta, icterícia, epigastralgia, sangramento, diátese hemorrágica (hematêmese), choque e falência de órgãos – Cerca de 50% dos casos são sintomáticos

A malária é causada pelo protozoário do gênero *Plasmodium* (no Brasil, as espécies *P. vivax* e *P. falciparum* ), transmitido pela picada do mosquito *Anopheles* . A malária é endêmica na região Amazônica, onde mais de 155 mil casos foram diagnosticados no ano de 2019. O *P. falciparum* é responsável pelas formas mais graves da doença e tem sido mais associado ao desenvolvimento de miocardite.^298^ Estudos de necrópsia de casos de malária grave mostram grande quantidade de parasitas no miocárdio e inflamação compatível com miocardite. A maioria dos estudos que reportam miocardite por malária consiste em séries de casos de pacientes internados, com avaliações de ECG, marcadores de lesão miocárdica e ecocardiograma.^299^ Essas séries de casos contemplam casos graves e mostram alteração dos marcadores de lesão cardíaca em até 59% e alterações ecocardiográficas como redução da função sistólica em até 19% dos pacientes avaliados. Muitos estudos que associam a malária ao IAM exibem falhas na definição do desfecho avaliado, sendo provavelmente casos de miocardite descritos como infartos. Nos casos de malária aguda que evoluem com a forma grave da doença, a disfunção miocárdica devido à miocardite por malária deve ser considerada. A avaliação com biomarcadores de lesão miocárdica e a função ventricular devem ser avaliadas para otimização do manejo cardiovascular.

As arboviroses são as doenças causadas pelos arbovírus, que incluem o vírus da dengue, Zika, febre Chikungunya e da febre amarela. São transmitidas pela picada do mosquito *Aedes aegypti* . O envolvimento cardiovascular nas arboviroses vem sendo demonstrado especialmente na dengue, que é a arbovirose mais prevalente no Brasil. A dengue é também aquela que tem maior percentual de manifestações cardiovasculares descritas, com estudos prospectivos relatando que 48% dos pacientes com a forma grave desenvolvem miocardite. Um estudo de necrópsia de quatro casos fatais de dengue mostrou achados de miocardite com presença de edema, hemorragia, infiltrado mononuclear e presença de antígeno e replicação viral.^300^

A Chikungunya é, dentre todas as arboviroses aqui mencionadas, a mais sintomática (80% dos casos); no entanto, normalmente se apresenta com sintomas leves e mais relacionados ao sistema osteoarticular. Ainda assim, a infecção pode se apresentar de maneira sistêmica e causar danos generalizados ou em órgãos específicos, como o coração. Um relato de caso em paciente com Chikungunya que desenvolveu dor torácica mostra ressonância com achados típicos de miocardite.^301^ Diversas séries de casos em situações de epidemia pelo vírus relatavam percentual de até 37% de acometimento cardiovascular, geralmente quadros compatíveis com micoardite.^302^

De todos as infecções tropicais aqui abordadas, a Zika é a que foi descoberta mais recentemente e também é a que apresenta o maior percentual de casos assintomáticos; quando tem manifestação clínica, esta ocorre predominantemente de forma congênita e envolvendo o sistema neurológico. Apesar disso, há alguns poucos estudos longitudinais envolvendo complicações não neurológicas dessa infecção em adultos, nos quais são apresentados desfechos cardiovasculares como IC, arritmias e IAM, bem como relatos de miocardite associada à Zika,^303 , 304^ além de estudos prospectivos de Zika congênita em que são relatadas alterações ecocardiográficas sugestivas de dano cardiovascular, sendo que este quadro possivelmente não representa o real impacto na doença no coração, uma vez que não há muitos estudos longitudinais que avaliem isso.

A febre amarela é uma arbovirose tropical negligenciada, a qual por muito tempo esteve concentrada apenas no ciclo silvestre, com baixa incidência (pouco notificada) e pouca expansão geográfica, o que contribuiu para que poucos estudos e casos fossem adequadamente relatados, em especial envolvendo o sistema cardiovascular. Ainda assim, com a crescente urbanização dessa doença e a melhor compreensão de seus mecanismos fisiopatológicos, sua relação com o coração vem sendo demonstrada por alguns estudos, entre eles, o estudo PROVAR+, que relatou, respectivamente, percentuais de 48% e 52% de alterações ecocardiográficas e eletrocardiográficas,^305^ além de análises *post-mortem* que isolaram o vírus no tecido cardíaco ou demonstraram dano miocárdico.

Portanto, muito embora a associação entre doenças tropicais e miocardite seja baseada em séries de casos e poucos estudos com diagnóstico bem-definido de miocardite, justifica-se a investigação diagnóstica das doenças comuns na região nos casos de miocardites em áreas endêmicas. Para tal, deve-se incluir a pesquisa de antígenos ou sorologias para arboviroses e gota espessa para pesquisa de malária. Nos casos de diagnóstico dessas doenças, um infectologista deve ser consultado para orientar o tratamento específico da malária ou o suporte nos casos de arboviroses. Uma outra situação clínica inclui pacientes com diagnóstico de arbovirose ou malária que evoluem com forma grave, especialmente choque; nesses casos, deve haver avaliação de lesão cardíaca com marcadores de necrose miocárdica e de função miocárdica com ecocardiograma para diagnóstico de acometimento miocárdico (miocardite), e o manejo deve incluir otimização da função miocárdica.

## 6.6. Miocardite por Covid-19

Coronavírus humanos têm sido associados à miocardite.^306 - 308^ Entre os seres humanos, durante o surto de SARS de Toronto, o RNA do vírus da SARS-CoV foi detectado em 35% dos corações autopsiados.^309^ Isso aumenta a possibilidade de danos diretos de cardiomiócitos pelo vírus^310 - 312^ ( Tabela 29 ).^313^

**Tabela 29:** – Estudos representativos abordando as manifestações cardiovasculares agudas da infecção por coronavírus e suas implicações clínicas311-313

Vírus	Tamanho da amostra	Manifestações cardiovasculares	Resultados
SARS	N=121	Hipotensão, taquicardia, braquicardia, cardiomegalia e arritmia	Principalmente transitória
N=15	Parada cardíaca	Morte
N=46	Compromisso diastólico subclínico sem envolvimento sistólico na ecocardiografia	Reversível na recuperação clínica
MERS	N=1	Miocardite aguda e insuficiência cardíaca aguda	Recuperada
Covid-19	N=14	Lesão miocardial (manifestando-se com aumento da troponina I cardíaca de alta sensibilidade) em cinco pacientes	Quatro pacientes necessitaram de cuidados intensivos
N=138	Lesão cardíaca aguda (7,2%), choque (8,7%) e arritmia (16,7%)	A maioria dos pacientes necessitou de cuidados intensivos

*Fonte: Tabela adaptada de Xiong et al.^313^*

### 6.6.1. Possível Fisiopatologia da Miocardite Relacionada ao SARS-CoV-2

**Figura 11:**
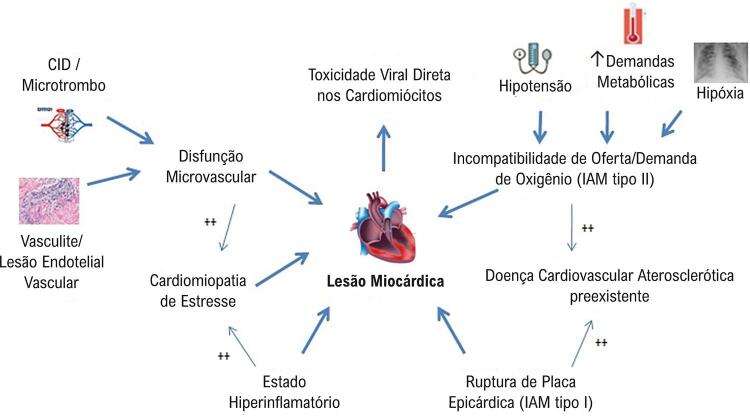
– Mecanismos potenciais de lesão miocárdica na Covid-19.

CID: coagulação intravascular disseminada; IAM: infarto agudo do miocárdio. Fonte: Adaptada de Atri D et al.^315^

Os mecanismos da lesão miocárdica não estão bem estabelecidos, mas provavelmente envolvem: lesão miocárdica secundária ao desequilíbrio entre oferta e demanda de oxigênio; lesão microvascular; resposta inflamatória sistêmica; cardiomiopatia por estresse; síndrome coronariana aguda não obstrutiva; e lesão miocárdica viral direta^314^ ( Figura 11 ).^315^

### 6.6.2. Lesão Miocárdica Viral Direta

**Figura 12:**
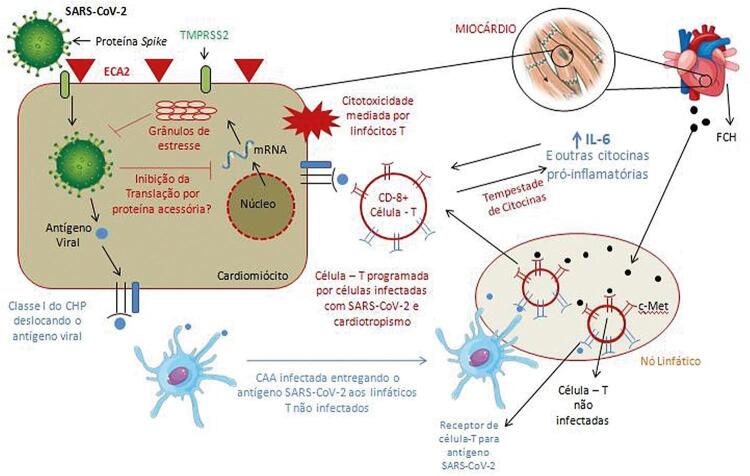
– Fisiopatologia proposta para miocardite por SARS-CoV-2. O SARS-CoV-2 utiliza a proteína spike (iniciada por TMPRSS2) para ligar o ACE2 para permitir a entrada de células. O SARS-CoV-2 intracelular pode prejudicar a formação de grânulos de estresse por meio de sua proteína acessória. Sem os grânulos de estresse, o vírus pode se replicar e danificar a célula. Os linfócitos T ingênuos podem ser preparados para antígenos virais via células apresentadoras de antígenos e cardiotropismo pelo HGF produzido pelo coração. O HGF liga o c-Met, um receptor de HGF nos linfócitos T. Os linfócitos T CD-8 iniciados migram para os cardiomiócitos e causam inflamação do miocárdio por citotoxicidade mediada por células. Na síndrome da tempestade de citocinas, na qual as citocinas pró-inflamatórias são liberadas na circulação, a ativação dos linfócitos T é aumentada e libera mais citocinas. Isso resulta em um ciclo de feedback positivo de ativação imune e dano do miocárdio.

CAA: célula apresentadora de antígeno; CHP: complexo principal de histocompatilbilidade; ECA2: enzima conversora de angiotensina 2; FCH: fator de crescimento de heapatócitos; IL-6: interleucina-6; SARS-CoV-2: síndrome respiratória aguda grave coronavírus 2. Fonte: Adaptada de Siripanthong B et al.^318^

Relatos de casos de miocardite na Covid-19 fornecem evidências de inflamação cardíaca, mas não determinam o mecanismo. A infecção por SARS- CoV-2 é causada pela ligação da proteína *Spike* da superfície viral ao receptor da enzima conversora de angiotensina 2 (ECA-2) humana. No entanto, a proteína *spike* deve primeiro ser clivada nos locais S1/S2 e, subsequentemente, nos locais S2’ para permitir a ligação à ECA-2. A clivagem no local S1/S2 parece ser mediada pela protease serina 2 transmembrana (TMPRSS2)^316 , 317^ ( Figura 12 ).^318^

Até o momento, temos apenas um relato de miocardite viral por SARS-CoV- 2 comprovada por biópsia com inclusões virais ou DNA viral detectado no tecido do miocárdio.^319^ Contudo, não havia a presença de partículas virais no cardiomiócito, apenas no interior dos macrófagos no interstício cardíaco. Outro mecanismo hipotético de lesão viral direta ao miocárdio é por meio de uma vasculite mediada por infecção. O receptor ECA2 é altamente expresso em artérias e veias endoteliais.^320^

Embora a ECA2 seja apenas levemente expressa no cardiomiócito, ela é altamente expressa nos pericitos. A Covid-19 pode atacar pericitos, essenciais para a estabilidade endotelial, causando disfunção endotelial, que leva a distúrbios microcirculatórios. Isso explica por que, embora a ECA2 seja apenas ligeiramente expressa nos cardiomiócitos, Covid-19 pode causar lesão cardíaca.^320^ As autópsias mostram infiltrados inflamatórios compostos por macrófagos e, em menor grau, por células T e CD4+.^321 - 322^ Esses infiltrados mononucleares estão associados a regiões de necrose de cardiomiócitos que, pelo Critério de Dallas, definem miocardite.^323^

### 6.6.3. Diagnóstico de Miocardite Relacionada à Covid-19

A apresentação clínica da miocardite por SARS-CoV-2 pode variar desde aqueles com sintomas leves, como fadiga, dispneia e dor precordial; em casos mais graves, podemos ter choque cardiogênico. Os pacientes podem apresentar sinais de IC direita, com aumento da pressão venosa jugular, edema periférico e dor no quadrante superior direito. A apresentação mais emergente é miocardite fulminante, definida como disfunção ventricular e IC dentro de 2 a 3 semanas após a infecção pelo vírus. Os sinais precoces de miocardite fulminante geralmente se assemelham aos da sepse.^14 , 324 - 329^

### 6.6.4. Laboratório

Elevações de troponina e NT-proBNP foram observadas nos casos de miocardite por Covid-19.^14 , 312 , 324 - 326^

Valores anormais de troponina são comuns nos pacientes com Covid-19, em especial quando utilizamos troponina cardíaca de alta sensibilidade (hs-cTn). Estudos que avaliaram o curso clínico de pacientes com Covid-19 observaram hs- cTnI detectável na maioria dos pacientes, e hs-cTnI foi significativamente elevado em mais da metade dos pacientes que morreram.^327 , 328^

Pacientes com Covid-19 geralmente demonstram elevação significativa do BNP ou NT-proBNP. O significado desse achado é incerto e não deve, necessariamente, desencadear uma avaliação ou tratamento para IC, a menos que haja clara evidência clínica para o diagnóstico. Em pacientes com Covid-19, o nível de BNP (NT-pro) também pode aumentar secundário ao estresse do miocárdio, como possível efeito de doença respiratória grave.

Devido à frequência e à natureza inespecífica dos resultados anormais de troponina ou peptídio natriurético entre pacientes com infecção por Covid-19, suas dosagens devem ser realizadas apenas se o diagnóstico de IAM ou IC estiver sendo considerado por motivos clínicos. Um resultado anormal de troponina ou peptídio natriurético não deve ser considerado evidência de IAM ou IC sem evidências corroboradoras.^329^

### 6.6.5. Eletrocardiograma

Alterações no ECG comumente associadas à pericardite, como elevação de ST e depressão de PR, podem ser observadas na miocardite;^310^ no entanto, esses achados não são sensíveis para a detecção da doença e sua ausência não é excludente.

Por exemplo, uma miocardite relacionada com Covid-19 não mostrou elevação do segmento ST nem depressão PR.^330^ Outras anomalias no ECG, incluindo novo bloqueio de ramo, prolongamento do intervalo QT, padrão de pseudoinfarto, extrassístoles ventriculares e bradiarritmia com BAV avançado, podem ser observadas na miocardite.^331^

Recentemente, foi publicada uma série de casos de pacientes com diagnóstico de Covid-19 que se apresentaram, em algum momento da infecção, com elevação do segmento ST no ECG.^332^

### 6.6.6. Imagem

A European Society of Cardiology (ESC), em recente documento, aponta as condições que devem ser consideradas diante da necessidade do uso de qualquer método de imagem cardiovascular em pacientes com Covid-19: deve ser utilizada para casos em que venha a determinar uma mudança substancial na conduta, ou quando uma decisão para salvar a vida do paciente esteja em jogo; deve-se usar a modalidade de imagem com a melhor capacidade para atender a essa solicitação, considerando-se sempre a segurança da equipe médica em relação à exposição; exames não urgentes, eletivos ou de rotina devem ser adiados ou até mesmo cancelados.^333^

Nesse sentido, a ecocardiografia transtorácica, embora tenha papel central na propedêutica cardiovascular desses pacientes, não deve ser rotineiramente indicada diante da corrente pandemia de Covid-19, sendo criteriosamente utilizada em casos específicos.^334^

As recentes recomendações da Society of Cardiovascular Computed Tomography (SCCT) para uso da angio-TC coronariana no contexto da Covid-19 incluem insuficiência cardíaca aguda de causa desconhecida^335 , 336^( Tabela 30 ).^337^

**Tabela 30:** – Recomendações da Society of Cardiovascular Computed Tomography (SCCT) para uso da angiotomografia coronariana no contexto da Covid-19

Urgência	Condições	Tempo para realização do exame
Eletivos	Coronariopatia assintomática ou estável Cardiomiopatia ou doença estrutural estável (valvar, planejamento de TAVI ou ablação de FA, congênita) Massas benignas	Em >8 semanas
Semiurgentes	Cardioversão de FA crônica Disfunção crônica ou subaguda de prótese valvar	Em 4 a 8 semanas
Urgentes	Dor torácica aguda ou estável de alto risco Intervenções estruturais de urgência (TAVI, oclusão de aurícula esquerda etc.) ou cardioversão de FA aguda Insuficiência cardíaca aguda de causa desconhecida Disfunção aguda valvar (ou prótese) Planejamento de biópsia de massa maligna	Em horas ou <2 a 4 semanas (a depender da gravidade)

*Descartar trombos quando RMC não factível. FA: fibrilação atrial; TAVI: implante transcateter da válvula aórtica. Fonte: adaptada de Araujo-Filho et al.^337^*

O documento da ESC sugere que troponinas positivas, associadas à disfunção miocárdica ou arritmias graves não explicadas por outros métodos, podem ser indicação para RMC, caso o diagnóstico seja crucial para o tratamento e o paciente esteja estável o suficiente para ser transferido com segurança para realização do exame.^334^

Nesse contexto, a atual orientação da *Society for Cardiovascular of Magnetic Resonance (SCMR)* sugere que um exame de RMC deva ser considerado de forma criteriosa e individualizada diante da suspeita de miocardite aguda com implicações imediatas no manejo do paciente.^337^ Caso a RMC seja realizada, os resultados devem ser interpretados de acordo com os critérios de Lake Louise: (1) edema; (2) lesão celular irreversível; e (3) hiperemia ou extravasamento capilar^338^ ( Tabela 31 ).^337^

**Tabela 31:** – Recomendações da Society for Cardiovascular of Magnetic Resonance (SCMR) para uso da ressonância magnética cardíaca (RMC) no contexto da Covid-19

Condições	Tempo sugerido para o exame
Pesquisa de isquemia e viabilidade miocárdica para orientar revascularização urgente	Dentro de 1 semana ou menos, a depender da gravidade
Suspeita de massa intracardíaca ou trombo com contraindicação para anticoagulação ou em pacientes com suspeita de eventos embólicos
Planejamento de ablação urgente em pacientes instáveis com arritmias graves
Constrição pericárdica exigindo potencial cirurgia urgente
Planejamento de implante percutâneo de valva cardíaca protética, com necessidade de cirurgia urgente

*Nota 1: Escolhas baseadas em consenso de especialistas. Nota 2: Condições clínicas individuais e contraindicações ao exame devem ser mandatoriamente consideradas. Fonte: Araujo-Filho et al.^337^*

### 6.6.7. Biópsia Endomiocárdica

Tanto a AHA como a ESC recomendam a BEM para o diagnóstico definitivo de miocardite, mas ambas as sociedades reconhecem suas limitações.^339 , 340^ Na era SARS-CoV-2, a utilidade clínica e o papel da BEM, atualmente o padrão-ouro para confirmar o diagnóstico de miocardite, permanecem incertos; além disso, há grande dificuldade na realização de imagens não invasivas, como ecocardiografia e RMC, com medidas adequadas de precaução e isolamento.^341 , 342^

Outro ponto a ser considerado é que em, em alguns casos, a infecção por SARS-CoV-2 pode não aparecer inicialmente com sinais e sintomas claros sugestivos de pneumonia intersticial, mas pode aparecer como miocardite sem sintomas respiratórios, às vezes complicada por choque cardiogênico com um curso fulminante.^14 , 316^

Adicionalmente, existem poucas evidências sobre o tratamento terapêutico da miocardite associada ao SARSCoV-2. Há um relato de caso em que foi utilizada terapia precoce com glicocorticoides e imunoglobulinas, com benefício para o paciente.^316^ Os corticosteroides têm sido utilizados em várias infecções respiratórias virais (influenza, SARS-CoV e MERS-CoV), demonstrando um benefício limitado e, em alguns casos, retardando a depuração viral e aumentando a mortalidade.^333^

No entanto, o Grupo de Trabalho da ESC sobre doenças miocárdicas e pericárdicas indica o uso de esteroides em miocardites por doenças autoimunes comprovada, miocardite com vírus negativo somente após determinar a infecção ativa no BEM.^340^ É evidente que, na prática real, a BEM nem sempre está disponível ,e seu papel na miocardite relacionada à SARS-CoV-2 ainda é desconhecido. Além disso, na ausência de estudos randomizados multicêntricos, o uso rotineiro de imunoglobulina também não é recomendado.

**Figura 13:**
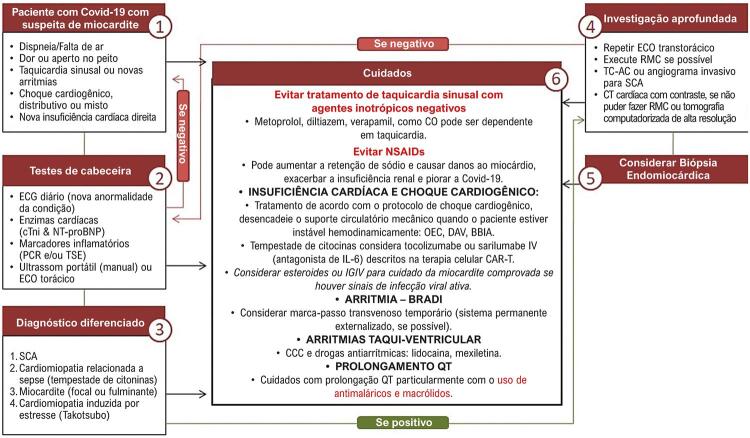
– Protocolo de diagnóstico e gerenciamento sugerido para miocardite relacionada a SARS-CoV-2.

BBIA: bomba de balão intra-aórtica; CCC: cardioversão por corrente contínua; DAV: dispositivo de assistência ventricular; DC: débito cardíaco; ECG: eletrocardiograma; IGIV: imunoglobulina intravenosa; IV: intravenoso; OEC: oxigenação por membrana extracorpórea; PCR: proteína C reativa; QT: intervalo QT; RMC: ressonância magnética cardiovascular; SCA: síndrome coronariana aguda; TC-AC: tomografia computadorizada angiograma coronário; TSE: taxa de sedimentação de eritrócitos. Fonte: Adaptada de Siripanthong et al.^318^

Em conclusão, acreditamos que existem lacunas significativas na avaliação do IAM em pacientes com SARS-CoV-2 que requerem uma análise diagnóstica completa, tratamentos priorizados e, ainda, estratégias mais agressivas,^318 , 319^ se necessário, especialmente naqueles que desenvolvem choque cardiogênico durante a miocardite fulminante^332 - 342^ ( Figura 13 ).^318^

## 6.7. Cardiotoxidade Aguda por Terapêutica Antineoplásica

### 6.7.1. Agentes Antineoplásicos Indutores de Cardiotoxidade Aguda

A evolução do tratamento do câncer nas últimas décadas resultou em melhora da sobrevida e da qualidade de vida dos pacientes.^343^ Entretanto, simultaneamente, com o aumento da longevidade, os fatores de risco cardiovasculares incidem por mais tempo e, associados a esse fato, adiciona-se o potencial risco de lesão ao sistema cardiovascular induzido pela quimioterapia, radioterapia e imunoterapia.^344^ Estudos recentes demonstram que há dois períodos de maior ocorrência de doença cardiovascular no paciente oncológico: o primeiro ano após o diagnóstico e os anos após a cura, nos quais denominamos os pacientes como sobreviventes, grupo este que demonstra aumento significativo de mortalidade cardiovascular.^345 , 346^

Dentre as toxicidades emergentes, destaca-se a miocardite. Mais recentemente, a miocardite relacionada ao tratamento do câncer ganhou importância devido à evolução da imunoterapia, mais especificamente relacionada aos inibidores de *checkpoint* imunológico (ICIs).^347 , 348^ Contudo, ela tem o potencial de estar associada a qualquer terapia que module o sistema imunológico. Identificar a miocardite nos ensaios clínicos em oncologia é desafiador, dada sua relativa baixa incidência e alta taxa de mortalidade.

Devemos ressaltar que as recomendações a seguir são advindas de consensos de especialistas, dada a escassez de dados científicos a respeito do tema.

O modelo clássico de cardiotoxicidade é a disfunção ventricular causada pelas antraciclinas.^349^ As antraciclinas são uma das classes mais utilizadas de quimioterápicos ainda nos dias de hoje. IC ocorre em até 30% dos pacientes, habitualmente após meses de tratamento, relacionada à dose cumulativa acima de 300 mg/m^2^. Na maioria dos casos, manifesta-se de forma subaguda ou crônica, após meses e anos do tratamento, com a irreversibilidade sendo sua característica predominante. A miocardite aguda relacionada às antraciclinas é manifestação rara, não apresentando relação com dose, sendo reversível na maioria dos casos.^350^ O mecanismo de ação da toxicidade está diretamente ligado ao estresse oxidativo consequente a sua metabolização, além da inibição da topoisomerase IIb, que, em última instância, resulta em dano ao DNA do cardiomiócito, por disfunção mitocondrial e apoptose.^351^

A ciclofosfamida é um agente alquilante tipo mostarda nitrogenada, que usualmente é parte de regimes de quimioterápicos que envolvem o uso concomitante de antraciclinas. Pode resultar em toxicidade aguda do tipo miocardite aguda hemorrágica e multifocal, caracterizada por endotelite, capilarite hemorrágica e trombogênese.^352^

Os inibidores de *checkpoint* imunológicos (ICI) são o modelo atualmente mais estudado como indutor de miocardite, sendo os mais comumente utilizados o nivolumabe, o durvalimabe, o ipilimumabe, o pembrolizumabe e o atezolizumabe.^353^ Essa terapia significou uma revolução no tratamento do câncer nos últimos anos, melhorando a sobrevida dos pacientes com câncer de pulmão, câncer de cabeça e pescoço, carcinoma renal, melanoma, entre outros.^354^ O mecanismo de ação se dá pelo bloqueio da apoptose dos linfócitos T (anti-CTLA4, anti-PD1, anti-PDL1), culminando na ativação dos linfócitos por todo o organismo. Se isso, por um lado, reativa o linfócito e a imunidade antitumoral, por outro lado, os linfócitos T ativados podem desencadear miocardite grave, fatal em até 50% dos casos. Clinicamente, manifesta-se em torno de 0,2% dos pacientes, em média 30 a 90 dias após o início do tratamento.^355 , 356^

### 6.7.2. Diagnóstico da Cardiotoxidade Aguda

A miocardite no paciente com câncer deve ser diagnosticada em situações de condições cardíacas sem diagnóstico primário alternativo (p. ex., síndrome coronariana aguda, traumatismo etc.).^357^ A história clínica deve considerar o regime de droga, o tempo do tratamento, assim como a dose e outras comorbidades. O diagnóstico laboratorial inclui a dosagem de biomarcadores como troponina ultrassensível e NT-proBNP. No caso da miocardite por imunoterápico, a dosagem de CPK também é recomendada pela associação com miosite em até 20% dos casos.^358^

O ECG pode ser útil para confirmar a suspeita de miocardite. Alterações comuns são arritmias ventriculares, alterações de ST-T, alterações do segmento PR, bradicardias e bloqueios.^357^

O ecocardiograma é o exame de escolha para a abordagem diagnóstica da miocardite. É realizado no início e na evolução, acessando função de maneira evolutiva. Os achados mais comuns incluem disfunção sistólica difusa, anormalidades segmentares, alterações na esfericidade do ventrículo, espessamento de parede, derrame pericárdico e alterações no *strain* .^357^

A RM é a modalidade de imagem de maior sensibilidade para o diagnóstico de miocardite, também tendo efeito de determinar o prognóstico. A combinação de achados da RMtem sido denominada Critérios de Lake Louise para o diagnóstico de miocardite aguda. Muitos avanços ocorreram no diagnóstico de miocardite por ressonância, e incluem avanços na caracterização tecidual por meio do MAPT1 e MAPT2 e cálcio do volume extracelular.^359^

A BEM pode ser considerada para investigação da miocardite relacionada a quimioterápicos e imunoterápicos. Especialistas recomendam, sempre que possível, a realização da biópsia, pois, em muitos casos, antes de manifestação clínica expressiva, os achados anatomopatológicos já exprimem a gravidade das alterações patogênicas da miocardite do câncer.^360^

A seguir, descrevemos os principais agentes antineoplásicos com potencial de induzir a miocardite com disfunção miocárdica ( Tabela 32 ).

**Tabela 32:** – Características da miocardite induzida pelo tratamento do câncer

	Antraciclinas	Ciclofosfamida	Inibidores de checkpoint imunológico
**Incidência**	10%	10%	0,2%
**Mortalidade**	20%	20%	50%
**Manifestação clínica**	IC aguda	IC aguda	IC aguda
**Diagnóstico**	Clínico, laboratorial, imagem e biópsia	Clínico, laboratorial, imagem e biópsia	Clínico, laboratorial, imagem e biópsia
**Reversibilidade**	Geralmente reversível	Geralmente reversível	Geralmente irreversível
**Reexposição**	Possível	Possível	Não recomendado

*IC: insuficiência cardíaca.*

### 6.7.3. Tratamento da Cardiotoxidade Aguda

Após a suspeita do diagnóstico, o tratamento deve ser iniciado imediatamente, pois o tempo pode ser importante na determinação do curso da doença. Embora não haja grandes estudos prospectivos para orientar o tratamento na MICI, a imunossupressão é a pedra angular do tratamento.

Os esteroides intravenosos são amplamente utilizados nos eventos adversos relacionados à imunoterapia (EAri) e podem ser eficazes na MICI.^347^ Altas doses de corticosteroides (p. ex., 1.000 mg por dia de metilprednisona por 3 dias, seguidas de 1 mg/kg de prednisona) são amplamente utilizadas e podem estar associadas a melhores resultados.^22^ Mahmood et al.^22^ relataram que 31% dos 35 pacientes receberam corticosteroides, e que altas doses foram associadas a níveis mais baixos de pico de troponina e menores taxas de MACE em comparação com doses reduzidas do corticoide. A Sociedade Americana de Oncologia Clínica (ASCO) recomenda 1 mg/kg de corticosteroides como dose inicial.^361^ A duração dos esteroides não é clara, mas a ASCO recomenda uma redução durante 4 a 6 semanas em pacientes com EAri. Os biomarcadores cardíacos séricos (p. ex., troponinas, BNP) podem ser úteis para definir a necessidade de maior duração após o desmame.

Imunossupressão adicional também pode ser usada. Evidências anedóticas sugerem que pode ser eficazes outros imunossupressores, tais como imunoglobulina intravenosa,^362^ infliximab,^363^ micofenolato,^364^ tacrolimus,^362^ globulina antitimocítica,^365 , 366^ plasmaférese,^362^ abatacept^367^ e alemtuzumab^368^. No estudo de Mahmood et al.,^22^ um pequeno número de pacientes recebeu outros imunossupressores não esteroides Dada a falta de dados robustos sobre sua eficácia no MICI, tais agentes geralmente são reservados para MICI refratário ou muito grave.

Sugerimos considerar a adição de imunossupressão não esteroide em pacientes que não demonstrem melhora sintomática, funcional ou de biomarcadores dentro de 24 a 48 horas após o início do corticosteroide. A escolha do segundo agente não é identificada, mas pode ser motivada pela disponibilidade e contraindicações. Vários e sequenciados imunossupressores podem ser necessários para alcançar remissão.^22^

**Figura 14:**
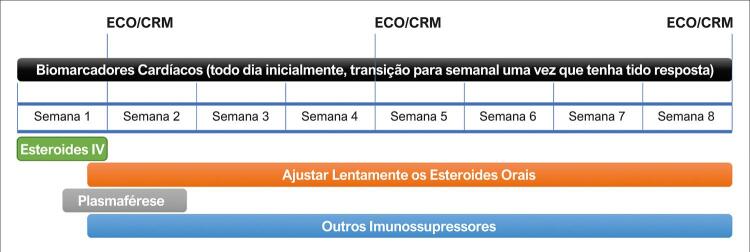
– Proposta do curso terapêutico de imunossupressão, biomarcadores e avaliação da função ventricular.

ECO: ecocardiograma; CRM: cirurgia de revascularização miocárdica; IV: intravenoso.

Recomendamos o início de altas doses de esteroides intravenosos no momento do diagnóstico de MICI (metilprednisona 1 mg/kg /dia). Os biomarcadores cardíacos (troponina e BNP) devem ser verificados em série. Se os biomarcadores cardíacos continuarem a aumentar, apesar da alta dose de esteroides, a plasmaférese deve ser iniciada. Um imunossupressor adicional deve ser adicionado se os biomarcadores cardíacos continuarem a aumentar ou se houver associação ou piora de arritmias ou IC ( Figura 14 ). A escolha do imunossupressor depende da experiência local e das comorbidades coexistentes ( Tabela 33 ).

**Tabela 33:** – Imunossupressores usados no tratatmento da MICI

Imunossupressor	Classe	Dose	Início	Duração
**Metilprednisona**	Corticosteroides	1 mg/kg/dia	No diagnóstico	2 a 3 dias
**Prednisona**	Corticosteroides	40 a 60 mg/dia	Dia 2-3	Desmame lento durante 4 a 8 semanas
**Infliximab**	TNF-alfabloqueador	5 mg/kg	Dia 4-5	Dose única (pode ser repetido em alguns meses)
**Globulina antimonócito**	?	10 a 30 mg/kg	Dia 2-3	7 a 14 dias
**Tacrolimus**	Inibidor da calcineurina	0,10 a 0,15 mg/kg/dia	?	?
**Micofenolato**	Inibidor IMPDH	1g 2×/dia	?	?
**Abatacept**	CTLA-4 agonista	500 mg a cada 2 semanas	Dia 7-14	Total de 5 doses
**Alemtuzumab**	Anti CD52	30 mg	?	Dose única

*IMPDH: inosina 5-monofosfato desidrogenase.*

Recomendamos a administração de uma dose única de infliximabe (5 mg/kg) se não houver contraindicações (por exemplo, tuberculose, hepatite). Alternativamente, podem ser usadas globulina antitimócita (10 a 30 mg/kg), alemtuzumabe (30 mg uma vez) ou abatacept (500 mg). Entre 3 a 5 dias após o início do corticosteroide, a função ventricular deve ser examinada (por ecocardiografia ou RMC). Pacientes que mostrem melhora significativa na função do VE (melhora da FEVE de pelo menos 5%) podem ser transferidos para corticosteroide oral (prednisona 40 a 60 mg por dia) por um longo tempo (4 a 8 semanas). Se os biomarcadores diminuírem e o paciente demonstrar resposta clínica, MMF ou tacrolimus podem ser utilizados para encurtar a cronicidade de esteroides. Dada a alta mortalidade e morbidade com o MICI, o ICI deve ser descontinuado mesmo em pacientes com cardiotoxicidade leve ( Tabela 33 ).

Dada a potencial reversibilidade do MICI, as terapias de suporte podem ser instituídas após cuidadosa consideração multidisciplinar do *status* da malignidade subjacente e do potencial de recuperação. Estratégias de suporte podem incluir suporte inotrópico, marca-passo temporário ou permanente, suporte circulatório mecânico temporário (p. ex., bomba de balão intra-aórtico,^6^ dispositivos de assistência ventricular percutânea^369^ou oxigenação extracorpórea por membrana [ECMO]).^362 , 364^ Uma avaliação cuidadosa do VD deve ser feita antes do início dos dispositivos de assistência do VE, pois MICI tem alta probabilidade de afetar o VD,^362 , 364 , 369^ o que pode exigir suporte biventricular.^363 , 369^ Ademais, devido ao ambiente protrombótico induzido pela neoplasia subjacente e EAri, é essencial excluir trombos do VE com RMC ou ecocardiograma com contraste, antes da inserção de dispositivos percutâneos de assistência do VE.^363^ A terapia medicamentosa para IC deve ser iniciada conforme tolerado. Isso inclui bloqueadores da angiotensina (ACE, ARB, ARNi), betabloqueadores e antagonistas dos mineralocorticoides (p. ex. espironolactona).

A segurança de reiniciar o tratamento com ICI após a resolução da miocardite não é conhecida. Em um estudo com 40 pacientes que desenvolveram EAri (1 MICI) nos quais ICI for reintroduzido (43% com o mesmo agente), 22 (55%) desenvolveram EAri recorrentes em um seguimento de 14 meses. Extrapolando esses dados para o MICI, e dada a alta probabilidade de recorrência de EAri com a reintrodução, a ASCO recomenda a descontinuação permanente do ICI em todos os casos de MICI.^370^ Há relato de reintrodução bem-sucedida em um caso de miocardite leve,^371^e a reintrodução de ICI pode ser tentada em casos selecionados de MICI leve e assintomática (grau I),^361^ especialmente com ICI de baixo risco com pembrolizumab. No entanto, essa recomendação permanece controversa.

### 6.7.4 Prognóstico

O prognóstico da MICI é difícil de definir devido à sua rara ocorrência. Em um registro multicêntrico de 35 pacientes com MICI, quase metade (n = 16) desenvolveu eventos cardiovasculares adversos importantes ao longo de um período de 102 dias (6 mortes cardiovasculares, 3 choques cardiogênicos, 4 paradas cardíacas, 3 bloqueios cardíacos completos).^22 , 347^ Em um registro francês de 30 pacientes com MICI em dois centros, oito pacientes morreram de complicações cardiovasculares. Um estudo recente que acompanhou 101 pacientes com MICI mostrou uma taxa de MACE de 51% durante um seguimento de 162 dias.^347^ Entre 250 pacientes com MICI relatados ao Sistema de Notificação de Eventos Adversos da Administração Federal de Medicamentos dos EUA (FAERS), a taxa de mortalidade foi de 50%.^361^ Não houve diferença na taxa de fatalidade por idade, sexo, ano de notificação ou tipo de ICI (proteína de morte celular antiprogramada-1/ligante de morte celular programada-1 *vs.* proteína de linfócito T anticitotóxica-4).^362^ Mahmood et al.^22^ descobriram que pacientes com MICI e troponina elevada na ocasião de alta hospitalar apresentaram taxas significativamente mais altas de MACE (troponina T de alta de ≥1,5 ng/mL: HR 4,0; IC95%: 1,5-10,9; p=0,003). Escudier et al.^348^ relataram que 80% dos pacientes com MICI e doença de condução tiveram morte cardiovascular. Um estudo recente de pacientes com MICI relatou que o *strain longitudinal global* (GLS) obtido ao diagnóstico de MICI estava fortemente associado a MACE ao longo de um seguimento de 162 dias.^363^ Dado o pequeno número de pacientes nesses estudos, é difícil identificar fatores de risco para mau prognóstico em pacientes que apresentam MICI.^364 - 368^

No geral, as taxas de recuperação com a terapia apropriada foram substanciais. Um total de 67% dos pacientes que receberam esteroides teve recuperação da função do VE no registro francês do MICI.^347^ Recuperação também foi descrita mesmo em pacientes com MICI fulminante que necessitaram de suporte hemodinâmico mecânico.^369 - 374^

### 6.7.5. Prevenção

A maioria dos estudos publicados na prevenção da cardiotoxicidade induzida pela quimioterapia baseia-se nas antraciclinas e nos agentes anti-HER2.

A prevenção da cardiotoxicidade deve iniciar-se antes do tratamento do câncer, com uma avaliação do risco cardiovascular do paciente e uma interação entre o cardiologista e o oncologista, a fim de programar a melhor abordagem durante o tratamento oncológico.

Os pacientes sob maior risco de desenvolver a cardiotoxicidade são aqueles que apresentam os fatores de risco clássicos para doença cardiovascular (hipertensão arterial, diabetes melito, dislipidemia, tabagismo, obesidade, sedentarismo, entre outros) ou aqueles sob maior exposição a fármacos cardiotóxicos (doses cumulativas altas para antraciclinas, associações de fármacos cardiotóxicas e antecedente de quimioterapia ou radioterapia).^375 , 376^

As principais recomendações para prevenção da cardiotoxicidade estão descritas na Tabela 34 .

**Tabela 34:** – Medidas para prevenção de cardiotoxicidade

Droga quimioterápica	Medida cardioprotetora
	Identificar fatores de risco cardiovascular
Tratar comorbidades (hipertensão arterial, diabetes melito, dislipidemia, tabagismo, sedentarismo, obesidade)
Atividade física aeróbica de intensidade moderada
Cuidados com arritmias: evitar drogas que prolongem o intervalo QT, ajustar distúrbios eletrolíticos
Minimizar radiação cardíaca
**Antraciclinas**	Limitar dose cumulativa (mg/m^2^):
	Daunorrubicina < 800
Doxorrubicina < 360
Epirrubicina < 720
Mitoxantrona < 160
Idarrubicina < 150
	Utilizar as formulações lipossomais
	Realizar infusão contínua
	Utilizar análogos menos cardiotóxicos (epirrubicina, idarrubicina)
	Avaliar uso de drogas cardioprotetoras (dexrazoxano, IECA, betabloqueadores, estatinas)
**Trastuzumabe**	Avaliar uso de drogas cardioprotetoras (IECA, betabloqueadores)

*IECA: inibidor da enzima conversora de giotensina; QT: intervalo QT. Fonte: Adaptada de Zamorano et al.^377^*

Entre as medicações cardioprotetoras, o dexrazoxano, um quelante de ferro, é a única medicação aprovada para a prevenção da cardiotoxicidade. Seu efeito contra a cardiotoxicidade por antraciclinas já foi comprovado em diversos estudos tanto na população adulta como na pediátrica.^376 - 383^ As limitações para o uso do dexrazoxano é o custo elevado e alguns potenciais efeitos adversos, tais como interferência na eficácia das antraciclinas, risco de desenvolvimento de tumores secundários (evidência controversa)^384 , 385^ e toxicidade medular. Seu uso está indicado em adultos com câncer de mama com estágio avançado ou metastático que receberam uma dose cumulativa prévia de 300 mg/m^2^ de doxorrubicina, 540 mg/m^2^ de epirrubicina, quando necessário a continuidade do tratamento com antraciclinas.

O uso de drogas cardiovasculares como betabloqueadores, IECA e bloqueadores do receptor da angiotensina (BRA) na prevenção da cardiotoxicidade secundária às antraciclinas é controverso e se baseia em poucos ensaios clínicos.^386 - 391^ Algumas evidências demonstraram benefícios dos betabloqueadores e IECA em pacientes que utilizaram doses cumulativas de antraciclinas elevadas ou em pacientes de alto risco, com troponina positiva durante a quimioterapia.^386 , 390^ Em doses cumulativas de antraciclina mais baixas, esse benefício não foi evidênciado com betabloqueadores,^389 , 392^ mas houve uma discreta prevenção com o uso de BRA.^389^

O ensaio clínico CECCY,^392^ um estudo brasileiro, testou o uso de betabloqueadores para prevenção primária da cardiotoxicidade por antraciclinas, não demonstrou benefício do uso do carvedilol na prevenção relacionada a antraciclinas. Entretanto, o carvedilol esteve associado a valores atenuados de troponina e menor porcentagem de pacientes com aparecimento de disfunção diastólica.

Em relação ao uso do trastuzumabe, alguns estudos também apontam benefício para o uso de drogas cardiovasculares tanto na prevenção da cardiotoxicidade^393 , 394^ quanto após o aparecimento da cardiotoxicidade, auxiliando na recuperação da disfunção ventricular.^395^ A decisão da suspensão do tratamento quimioterápico, bem como seu retorno, deve ser feita em conjunto, pesando o risco e o benefício da manutenção do tratamento oncológico.

## 6.8 Miocardite em Crianças e Adolescentes

### 6.8.1. Fatores Causais

**Figura 15:**
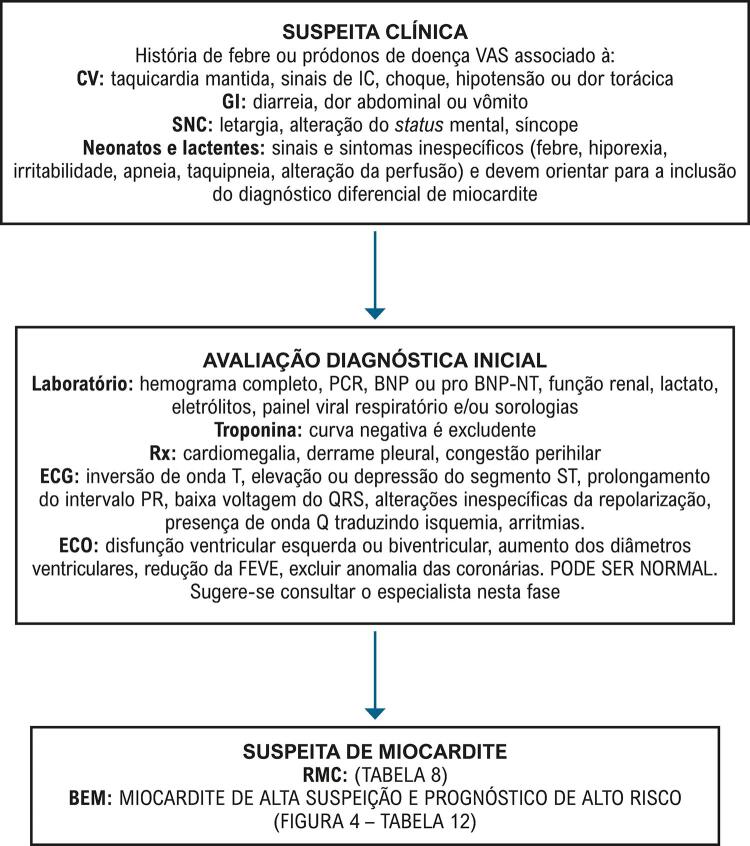
– Fluxograma de suspeita e investigação das miocardites em crianças e adolescentes.

BEM: biópsia endomiocárdica; BNP: peptídeo natriurético tipo B; CV: cardiovascular; ECG: eletrocardiograma; ECO: ecocardiograma; FEVE: fração de ejeção de ventrículo esquerdo; GI: gastrointestinal; IC: insuficiência cardíaca; PCR: proteína C reativa; RMC: ressonância magnética cardíaca; SNC: sistem nervoso central.

A miocardite em crianças e adolescentes apresenta particularidades em sua etiologia, e o seu diagnóstico pode ser subestimado pela similaridade de sua apresentação inicial com inúmeras viroses comuns na infância. Estima- se que mais de 83% dos pacientes compareceram aos serviços de urgência por duas ou mais visitas antes do diagnóstico.^396^ Nas análises retrospectivas, a dor torácica foi referida predominantemente em crianças maiores de 10 anos, e os sinais mais comuns observados nos mais jovens foram taquipneia, febre e desconforto respiratório^397^ ( Tabela 35 ). A aplicação de algoritmos para o diagnóstico em salas de emergência tem se mostrado promissora, com a possibilidade de aumentar o número de pacientes suspeitos ( Figura 15 ).^239 , 398^ Em relação à etiologia, estudos avaliando a coleta de painel viral no quadro agudo e a confirmação por biópsias, observamos o parvovírus B19 como predominante, seguido pelos enterovírus, Coxsackievírus B e herpes-vírus humano.^398^ Casos relacionados aos arbovírus – responsáveis por dengue, Zika e Chikungunya – t =êm sido descritos em regiões endêmicas ao redor do mundo.^399^ Mais recentemente, com a pandemia de SARS-Cov2, têm sido relatadas apresentações com agressão miocárdica associada ou não à síndrome inflamatória multissistêmica com fisiopatologia ainda pouco esclarecida.^400^ Sobreviventes ao tratamento dos cânceres da infância, principalmente os submetidos ao tratamento com antracíclicos e inibidores de *checkpoint* , constituem uma parcela de alto risco à instalação do processo inflamatório levando à IC na idade adulta.^401^

**Tabela 35:** – Achados clínicos mais comuns na apresentação inicial das miocardites em crianças e adolescentes

Sinais e sintomas	Menores de 2 anos	Pré-escolares	Escolares e adolescentes
**Específicos**	Sinais de IC	Sinais de IC	Sinais de IC
	História de doença viral nas últimas 3 a 6 semanas	História de doença viral nas últimas 3 a 6 semanas	Dor torácica
Dor torácica (pouco comum)	Dor torácica (pouco provável)	História de doença viral pode não estar tão clara
**Inespecíficos**	Febre	Dispneia aos esforços	Dispneia aos esforços
	Letargia	Taquicardia em repouso	Taquicardia em repouso
Irritabilidade	Fadiga muscular	Fadiga muscular
Alteração da perfusão	Arritmias	Arritmias
Hiporexia	Choque	Choque
Taquicardia mantida em repouso		
Arritmias
Choque

*IC: insuficiência cardíaca.*

### 6.8.2. Prognóstico

É difícil estimar a incidência e a prevalência de miocardite na faixa etária pediátrica é devido ao amplo espectro de sintomas, que pode variar desde um quadro viral leve sem comprometimento hemodinâmico até um quadro de IC congestiva, com disfunção ventricular, arritmias e morte súbita.^164 , 402 - 405^ Como os sintomas, muitas vezes, são inespecíficos, um significativo número de casos não é diagnosticado, o que dificulta a caracterização da real incidência e prognóstico. Entretanto, é a principal etiologia da miocardiopatia dilatada em crianças.

Com a melhora das unidades de terapia intensiva, incluindo a possibilidade de suporte mecânico à circulação, o prognóstico de crianças de todas as faixas etárias tem melhorado, com possibilidade de recuperação completa mesmo de casos com doença fulminante.^402^

Os principais desfechos em paciente pediátricos incluem recuperação completa, progressão para miocardiopatia dilatada e morte ou transplante cardíaco.^405^

Acredita-se que, em crianças com miocardite viral, o prognóstico tende a ser melhor do que nas miocardiopatias dilatadas. A sobrevida de pacientes pediátricos com miocardite pode ser de até 93%. Entretanto, um estudo multicêntrico englobando todas as faixas etárias demonstrou que existe uma significativa mortalidade em neonatos e lactentes. A sobrevida nessa faixa etária foi de 33% a 45%, e a melhora clínica, de 23% a 32%. Em crianças entre 1 e 18 anos, a sobrevida foi melhor, em torno de 78% e 80%, e a melhora clínica, entre 46% e 67%.^406^Em um estudo recente do Pediatric Cardiomyopathy Registry (PCMR), crianças com miocardite confirmada por biópsia tiveram uma sobrevida de 75% em 3 anos, e 54% do grupo normalizaram as dimensões e função ventricular, e apenas 20% permaneceram com anormalidades ecocardiográficas.^404^

Em outro estudo com 28 pacientes com diagnóstico de miocardite, foi observado que apenas 17 sobreviveram e tiveram alta hospitalar, com vários graus de melhora da função cardíaca. Os demais 11 pacientes evoluíram para IC refratária, sendo necessário transplante cardíaco em sete casos, e ocorreu óbito em quatro casos. Preditores de mau prognóstico foram: fração de ejeção abaixo de 30%, fração de encurtamento abaixo de 15%, dilatação ventricular esquerda, regurgitação mitral moderada a severa.^406^

Várias séries de casos envolvendo crianças que necessitaram de suporte mecânico à circulação por miocardite reportam taxa de sobrevida entre 67% e 83%. Para 21 pacientes com suporte mecânico com Berlin Heart Excor por miocardite ou miocardiopatia dilatada, 90% sobreviveram com alta hospitalar.^407^

O prognóstico em miocardite comprovada por BEM depende da gravidade dos sintomas, da classificação histológica e biomarcadores. Miocardite aguda fulminante é associada com melhor sobrevida. Miocardite por células gigantes, apesar de rara, é associada com mau prognóstico, com uma sobrevida média de 5,5 meses, com uma taxa de mortalidade ou transplante de 89%.^406^

Miocardite contribui para pelo menos 50% das miocar- diopatias dilatadas na infância. O desfecho de pacientes com miocardite viral é melhor que aqueles com miocardiopatia dilatada. Por esse motivo, deve-se sempre suspeitar de miocardite, instituir medidas de suporte precocemente, evitando que um paciente com miocardite seja encaminhado à lista de transplante sem a chance de recuperação. A indicação de transplante na miocardite só deve ser considerada quando a recuperação for desfavorável, apesar do manejo terapêutico adequado ( Tabela 36 ).

**Tabela 36:** – Principais informações sobre miocardites na criança e no adolescente

Miocardites na criança e no adolescente
Os principais desfechos incluem: recuperação completa, progressão para miocardiopatia dilatada e morte ou transplante cardíaco
Imunoglobulina endovenosa tem se tornado uma prática no tratamento das miocardites, porém o seu efeito na função cardíaca ainda não está completamente esclarecido
O espectro de manifestações clínicas da miocardite é muito amplo, desde um quadro viral leve até insuficiência cardíaca congestiva com choque cardiogênico com necessidade de suporte inotrópico ou mecânico à circulação
Apesar de a biópsia miocárdica ser considerada padrão-ouro para o diagnóstico de miocardite, em crianças, o risco de eventos adversos varia de 1% a 5% (taquiarritmias, hipotensão pela anestesia, alterações isquêmicas, perfuração ventricular). Portanto, essa prática não tem sido adotada de rotina^5^
Fração de ejeção <42% e troponina elevada ao diagnóstico têm maior associação com mortalidade
Pacientes que sobrevivem à fase aguda têm um desfecho tardio melhor que aqueles que têm um quadro mais insidioso
Miocardite é a principal etiologia da miocardiopatia dilatada na infância

A utilização de imunoglobulina (IVIG) tem se tornado parte do tratamento imunomodulatório em crianças com miocardite aguda em muitos centros, na dose *standard* de 2g/kg em 24 horas. Essa prática tem sido instituída desde a clássica publicação de Drucker et al., em 1994.^164^ Foi demonstrada uma tendência à recuperação da função ventricular naqueles que receberam imunoglobulina. Em uma coorte de 94 pacientes com miocardiopatia de início recente, IVIG foi administrada em 22% dos pacientes, e o seguimento de 5 anos demonstrou uma maior taxa de recuperação quando comparados com os demais pacientes que não receberam imunoglobulina.^408^

Em um estudo realizado em Taiwan com 94 pacientes, a avaliação da curva ROC identificou que a fração de ejeção <42% (sensibilidade 86,7% e especificidade de 82,8%) e a dosagem de troponina I >45ng/mL (sensibilidade de 62,6% e especificidade de 91%) tiveram a maior associação com mortalidade.^403^

Vários estudos demonstraram que os pacientes que sobrevivem à fase aguda inicial têm um desfecho mais favorável a longo prazo, ao contrário daqueles com doença mais insidiosa.

Evidência histológica de miocardite como causa de miocardiopatia dilatada tem sido considerada um indicador prognóstico positivo para recuperação, com chances de cura entre 50% e 80% em 2 anos.^402^ Da mesma forma, a evolução para IC crônica com necessidade de transplante cardíaco pode ocorrer tardiamente mesmo após a melhora clínica inicial.

## 6.9. Miocardites com Envolvimento Pericárdico

### 6.9.1. Diagnóstico e Tratamento

Miocardites e pericardites são doenças que, com frequência, se apresentam associadas na prática clínica e representam diferentes espectros dentro do grupo das síndromes inflamatórias miopericárdicas ( Tabela 37 ).^409 , 410^Isso se deve ao fato de ambas as doenças apresentarem agentes etiológicos comuns (especialmente virais).^411^ No entanto, raramente o acometimento miocárdico e pericárdico ocorre na mesma intensidade. O mais comum é haver predomínio da miocardite (perimiocardite) ou da pericardite (miopericardite).^412^ A distinção entre as diferentes formas de apresentação é relevante por ter impacto no prognóstico e tratamento. A miopericardite usualmente tem boa evolução, sem IC ou pericardite constritiva.^413 - 416^ No cenário da miocardite aguda, o acometimento pericárdico (perimiocardite) tem importância prognóstica. No estudo de Di Bella et al.,^417^ que avaliou uma coorte de 467 pacientes com miocardite aguda viral/idiopática diagnosticada pela RMC, observou-se que aproximadamente 24% dos pacientes tinham acometimento pericárdico. Além disso, a presença de pericardite aumentou em 2,5 vezes o risco de eventos cardíacos (desfecho combinado de morte, transplante cardíaco, implante de CDI, hospitalização por IC descompensada).^416^

**Tabela 37:** – Recomendação de avaliação nos casos de miocardite com suspeita de acometimento pericárdico

Indicação	Classe	Nível de evidência
No paciente com miocardite aguda com suspeita de acometimento pericárdico, a ressonância magnética cardíaca é indicada para elucidação diagnóstica nos casos duvidosos	I	C

O diagnóstico de miocardite associada à pericardite aguda deve ser suspeitado nos pacientes que apresentam diagnóstico de miocardite e pelo menos dois dos seguintes critérios: dor torácica de caráter pleurítico, que pode ser difícil de identificar pela presença de dor pelo acometimento miocárdico; atrito pericárdico; alterações eletrocardiográficas sugestivas de pericardite com infra do segmento PR e supra de ST difuso com a concavidade para cima; derrame pericárdico novo ou piora do preexistente. O laboratório geralmente revela leucocitose com predomínio de linfócitos (nos quadros virais) e elevação da PCR e velocidade de hemossedimentação (VHS). A RMC é o exame não invasivo com melhor acurácia para avaliação de acometimento pericárdico no paciente com miocardite.^409 , 417^ O exame revela a presença de inflamação, espessamento, derrame e massas no pericárdio, e está indicado em todo os casos com dúvida diagnóstica (grau de recomendação I, nível de evidência C).^409 , 418^

No paciente com miocardite e acometimento pericárdico, o tratamento deve seguir as recomendações para tratamento da miocardite e depende essencialmente da causa de base. Nos casos virais/idiopáticos sem disfunção ventricular, o uso de AINEs para controle da lesão pericárdica deve ser considerado com cautela, em doses reduzidas, uma vez que, em estudos experimentais, os AINES revelaram aumento de mortalidade e piora da inflamação miocárdica.^411 , 419 , 420^

## 6.10. Miocardite Simulando Infarto Agudo do Miocárdio

Estudos anteriores indicaram que 2,6% a 25% dos pacientes com suspeita de IAM revelaram-se como IAM sem doença arterial coronariana obstrutiva (MINOCA; do inglês, *myocardial infarction with non-obstructive coronary artery* ). Existem várias etiologias que podem ser atribuídas aos indivíduos com suspeita de IAM, mas com angiogramas sem lesões culpadas, dentre as quais miocardite aguda tem sido reconhecida como um fator particularmente importante.^421^

É comum que as apresentações clínicas típicas do IAM, como dor no peito, elevação do segmento ST e marcadores séricos incrementais, apareçam em pacientes diagnosticados com miocardite.^422 , 423^

Além disso, no cenário clínico de doença aguda com elevação de troponina, pode ser clinicamente desafiador diferenciar um IAM tipo 2 de causas de lesão miocárdica sem isquemia, principalmente a miocardite. O IAM tipo 2 é aquele secundário à isquemia devido ao aumento da demanda de oxigênio ou diminuição da oferta, causado, por exemplo, por espasmo da artéria coronária, embolia coronária, anemia, arritmias, hipertensão ou hipotensão.^424^

O termo *isquemia miocárdica* é utilizado quando há evidências de valores elevados de troponina com pelo menos um valor acima do limite superior de referência (URL) do percentil 99. O termo *injúria miocárdica* é considerado se houver aumento e/ou queda dos valores de troponina. O diagnóstico de IAM é específico quando há lesão miocárdica aguda associado à evidência clínica de isquemia miocárdica aguda, exigindo tanto detecção de um aumento e/ou queda dos valores de troponina e a presença de pelo menos uma das seguintes condições: sintomas de isquemia miocárdica, alterações isquêmicas novas no ECG, desenvolvimento de ondas Q patológicas, evidência de imagem de nova perda de miocárdio viável ou de novas anormalidades do movimento da parede em um padrão consistente com um quadro isquêmico e/ou identificação de trombo coronário por angiografia ou autópsia.^424^

As principais entidades clínicas que podem simular um IAM com supradesnível do segmento ST são: miocardite/pericardite, cardiomiopatia de Takotsubo, síndromes da onda J (usado para descrever tanto a síndrome de Brugada quanto a síndrome de repolarização precoce), anormalidades secundárias da repolarização (como bloqueio de ramo esquerdo, marca-passo ventricular e hipertrofia ventricular), distúrbios eletrolíticos (hipercalemia e hipercalcemia) e outras causas não isquêmicas (como síndrome de Wolff-Parkinson-White, embolia pulmonar, hemorragia intracraniana, hipotermia e pós-parada cardiorrespiratória), porém as alterações eletrocardiográficas evolutivas podem ajudar na diferenciação, além das diferenças nas histórias clínicas.^425^

A caracterização tecidual *in vivo* com RMC permite a identificação de edema/inflamação nas síndromes coronarianas agudas/miocardite e diagnóstico de doenças crônicas e condições fibróticas (p. ex., em cardiomiopatias hipertróficas e dilatadas, estenose aórtica e amiloidose).^425^ Na doença não isquêmica, o padrão e a distribuição do realce tardio (LGE; do inglês, *late gadolinium enhancement* ) podem oferecer pistas sobre etiologia e significado prognóstico.^425^ A miocardite geralmente causa cicatrizes subepicárdicas/mesocárdicas, geralmente (embora nem sempre) em uma distribuição não coronariana, poupando o subendocárdio^426 , 427^

Na miocardite, a imagem ponderada em T2 também pode identificar regiões de inflamação, caracteristicamente, em uma distribuição não coronariana. Por outro lado, o mapeamento T1 paramétrico está também disponível, fornecendo avaliação quantitativa e objetiva do edema/inflamação (p. ex., no IAM/miocardite). ^426 , 427^Existe uma interação dinâmica entre inflamação e fibrose em vários precursores de IC, como IAM e miocardite. O diagnóstico precoce de IC com biomarcadores e imagem é fundamental; enquanto a RMC é útil para avaliar a extensão da lesão, medições seriadas de biomarcadores indicam se inflamação e fibrose são progressivas.^427^

**Figura 16:**
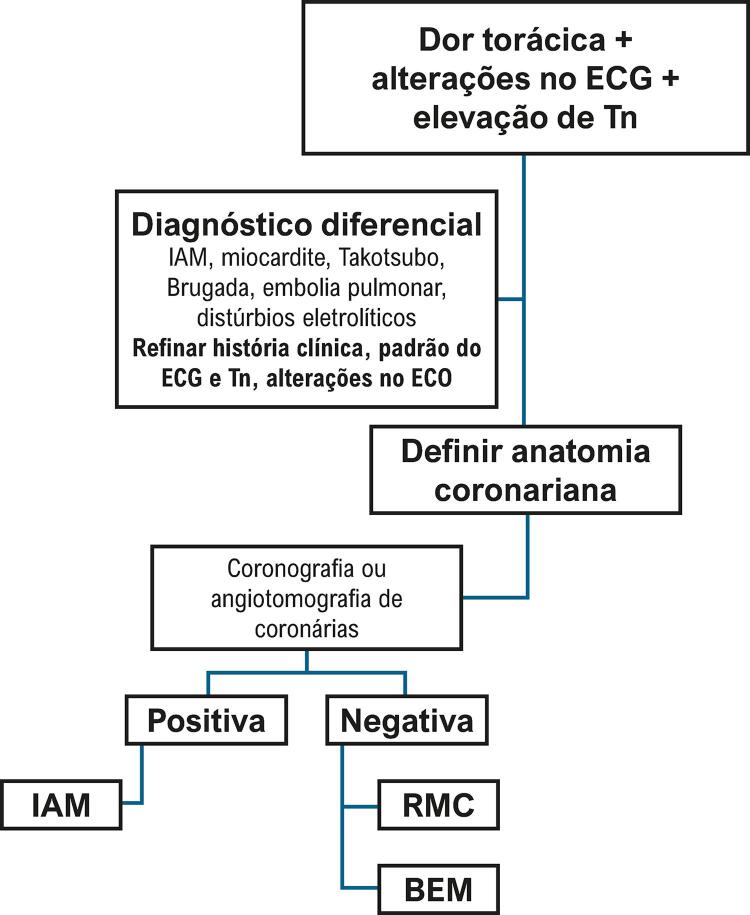
- Diagnóstico diferencial de dor torácica: IAM versus miocardite.

BEM: biópsia endomiocárdica; ECG: eletrocardiograma; ECO: ecocardiograma; IAM: infarto agudo do miocárdio; RMC: ressonância magnética cardíaca; Tn: troponina.

Clinicamente, caso de miocardite simulando IAM é extremamente complexo para os médicos fazerem um diagnóstico preciso. A definição da anatomia coronariana é mandatória, seja com a coronariografia ou com a angiotomografia de coronárias. Além disso, um diagnóstico correto de miocardite, por si só, é um desafio devido a padrões não específicos de sua apresentação clínica e a falta de um método de diagnóstico preciso e confiável. Embora seja recomendada a realização de BEM nas diretrizes como método ideal, o diagnóstico de miocardite na prática rotineira é geralmente baseado em considerações abrangentes do histórico médico dos pacientes, manifestações clínicas e exames complementares, dentre os quais a RMC, que tem vantagem significativa na detecção de anormalidades do miocárdio e na discriminação precisa de pacientes com miocardite daqueles com IAM verdadeiro.^421 - 423 , 425 - 427^ Na Figura 16 , sugerimos um fluxograma de avaliação de paciente com IAM *versus* miocardite.

## 7. Cardite Reumática

Em 2018, a Organização Mundial da Saúde (OMS) reconheceu a endemia de febre reumática em países de baixa renda e orientou ação global focada em prevenção, diagnóstico e profilaxia secundária.^428^ A febre reumática é uma doença bifásica, cujo surto agudo manifesta-se com variáveis combinações de artrite, cardite, coreia, lesões cutâneas e subcutâneas, e a miocardite ocorre em mais de 50% dos pacientes.^429^ Cerca de 5% dos pacientes com miocardite reumática aguda apresentam manifestação clínica significativa que motivam atendimento médico e até 50% dos pacientes com cardite aguda evoluem para cardiopatia reumática crônica (fase tardia), caracteristicamente valvopatia mitral e/ou aórtica.^430 , 431^ A prevalência de cardite reumática em nosso meio não é conhecida, mas várias informações evidenciam que se trata de condição frequente e subdiagnosticada. Em 2013, o Sistema Único de Saúde (SUS) brasileiro informou que ocorreram 5.169 hospitalizações relacionadas à febre reumática aguda.^432^ Estima-se que, atualmente, cerca de 40 milhões de pessoas ao redor do mundo tenham cardiopatia reumática crônica e que essa doença leve a aproximadamente 300.000 mortes por ano.^433^ Um estudo brasileiro realizado no estado de Minas Gerais, em 5.996 estudantes de 21 escolas, encontrou 0,42% de prevalência de cardiopatia reumática crônica, número 2 a 10 vezes maior à média documentada em países desenvolvidos.^434^

A suspeita de cardite reumática deve ser feita concomitante à suspeita de surto agudo de febre reumática, inicialmente por meio da aplicação dos critérios de Jones, que foram revisados em 2015.^435^ Recomenda-se estratificar epidemiologicamente o risco de causa reumática, sendo considerados de alto risco pacientes oriundos de regiões cuja incidência de febre reumática é maior que 2 por 100 mil escolares (5 a 14 anos) por ano, ou prevalência de sequela valvar reumática maior que 1 por 1.000 pessoas por ano. Estima-se que grande parte da população brasileira resida em regiões com essas características. Também houve inclusão de critérios ecocardiográficos e expansão da utilização dos critérios para diagnóstico de recidiva^436^ ( Tabela 38 ). Portanto, a etiologia reumática deve ser considerada em pacientes com cardite em nosso meio, principalmente jovens, em regiões de baixa renda e/ou com antecedente de valvopatia reumática.

**Tabela 38:** – Critérios de Jones modificados em 2015

Primeiro surto de febre reumática	Recidiva de febre reumática
2 critérios maiores; ou 1 critério maior e pelo menos 2 menores	2 critérios maiores; ou 1 critério maior e pelo menos 2 menores; ou 3 critérios menores
População de baixo risco (<2/100.000 casos de febre reumática aguda por anos e <1/1.000 casos de sequela valvar reumática por ano)	População de risco moderado/alto risco (>2/100.000 casos de febre reumática aguda por anos e >1/1.000 casos de sequela valvar reumática por ano)
**Critérios maiores**	**Critérios maiores**
– Cardite (clínica ou subclínica)	– Cardite (clínica ou subclínica)
– Artrite (apenas poliartrite)	– Artrite (apenas poliartrite, poliartralgia e/ou monoartrite)
– Coreia	– Coreia
– Eritema marginado	– Eritema marginado
– Nódulo subcutâneo	– Nódulo subcutâneo
**Critérios menores**	**Critérios menores**
– Poliartralgia	– Monoartralgia
– Febre (maior ou igual 38,5°C)	– Febre (maior ou igual 38°C)
– Elevação de VHS (>60 mm na primeira hora) e/ou PCR maior que o limite superior de referência)	– Elevação de VHS (>60 mm na primeira hora) e/ou PCR maior que o limite superior de referência)
– Intervalo PR prolongado corrigido para idade (quando não houver cardite)	– Intervalo PR prolongado corrigido para idade (quando não houver cardite)
Evidência de infecção prévia pelo *Streptococcus* B-hemolítico do grupo A (cultura positiva de orofaringe; teste rápido positivo; escarlatina; títulos elevados de anticorpos antiestreptocócicos)	Evidência de infecção prévia pelo *Streptococcus* B-hemolítico do grupo A (cultura positiva de orofaringe; teste rápido positivo; escarlatina; títulos elevados de anticorpos antiestreptocócicos)

*PCR: proteína C reativa; VHS: velocidade de hemossedimentação.*

Quando há documentação de surto agudo de febre reumática ou manifestação clínica de IC, é fundamental a busca ativa por cardite reumática. A cardite reumática é uma pancardite, acometendo em grau variável pericárdio, miocárdio e endocárdio, sendo esta a principal manifestação: valvulite aguda – presente em 90% dos casos, caracteristicamente por valvopatia regurgitativa aguda mitral e/ou aórtica.^437^ Quando há sintomas, o principal mecanismo é a valvopatia aguda (preferencialmente mitral) e, menos frequente e com menos intensidade, miocardite e pericardite.^438^ Portanto, o foco inicial da investigação é a detecção da valvopatia, podendo ser reconhecida por exame físico, mas é mandatória a realização ecocardiográfica, inicialmente transtorácica, reservando-se a avaliação transesofágica para situações infrequentes de janela inadequada.^439^ O ECG de 12 derivações, além do alargamento do intervalo PR, pode demonstrar QT longo e alterações compatíveis com pericardite e sobrecarga de câmaras esquerdas.^440^ Habitualmente, não há elevação de troponina e CKMB, indicando que o dano miocárdico é pequeno.^431 , 441^ A radiografia de tórax pode ser útil em documentar cardiomegalia e congestão.^442^Após essa avaliação inicial, a hipótese diagnóstica pode ser:^443 , 444^

- Cardite subclínica: exame clínico sem alterações de alerta, ECG apenas com intervalo PR prolongado e/ou ecodopplercardiograma evidenciando regurgitação leve mitral e/ou aórtica
- Cardite leve: taquicardia desproporcional à febre, sopro regurgitativo identificável, ECG apenas com intervalo PR prolongado, radiografia de tórax sem alterações de alerta e ecodopplercardiograma evidenciando regurgitação leve a moderada mitral e/ou aórtica
- Cardite moderada: critérios da cardite leve associados a sintomas leves de IC e/ou QT longo e/ou cardiomegalia e congestão na radiografia e/ou dilatação leva a moderada de câmaras esquerdas
- Cardite grave: sintomas limitantes de IC com regurgitação valvar importante e/ou cadiomegalia importante e/ou disfunção ventricular sistólica.

Dessa forma, a miocardite reumática, em si, é pouco exuberante, deve ser suspeitada quando há critérios para cardite reumática, IC manifesta, sem valvopatia aguda anatomicamente importante. Nessa situação, também é fundamental a avaliação minuciosa de possível diagnóstico diferencial de miocardite.

Pacientes com quadros leves, moderados e graves devem ter continuação da investigação diagnóstica com exames de imagem. A cintilografia com Gálio-67 apresenta alta sensibilidade e especificidade, é o exame mais estudado e deve ser o primeiro a ser realizado.^445 , 446^ Cintilografia antimiosina é menos sensível, assim como o PET *scan* , sendo ambos opções em indisponibilidade do Gálio, ou quando há evidência de outros diagnósticos diferenciais.^447 , 448^A RM carece de trabalhos específicos para febre reumática, ainda mais que o acometimento é prioritariamente valvar, sendo mais um exame útil em diagnósticos diferenciais.^449^ A BEM apresenta baixa sensibilidade, porém altíssima especificidade, sendo o achado de nódulos de Aschoff patognomônico de miocardite reumática. Sua indicação é em casos graves e refratários^450^ ( Tabela 39 ).

**Tabela 39:** – Exames diagnósticos para cardite reumática

Indicações	Classe	Nível de evidência
Eletrocardiograma 12 derivações	I	B^440^
Radiografia de tórax	I	C^442^
Ecodopplercardiograma transtorácico	I	B^436,439^
Ecodopplercardiograma transesofágico em situações de dificuldade de visualização transtorácica	I	C^436,439^
VHS e PCR (ver critérios de Jones)	I	B^436^
Antiestreptolisina O (ver critérios de Jones)	I	C^436^
Antidexirribonuclease B com alternativa à antiestreptolisina O	IIa	C^435^
Alfa-1 glicoproteína ácida para monitoramento da atividade inflamatória	IIa	C^444^
Eletroforese de proteína (alfa-2 globulina) para monitoramento da atividade inflamatória	IIa	C^444^
Troponina como critério diagnóstico	IIb	B^431,441^
Cintilografia com Gálio-67	IIa	B^445,446^
PET ^18^F-FDG CT	IIb	B^448^
Ressonância cardíaca	IIb	C^449^
Biópsia endomiocárdica	IIb	C^444,450^

*PCR: proteína C reativa; PET ^18^F-FDG: tomografia por emissão de pósitrons ^18^F-fluorodesoxiglicose; VHS: velocidade de hemossedimentação.*

Para todos os pacientes com cardite reumática, apesar de se tratar de resposta imune tardia, recomenda-se erradicação estreptocócica.^451^ O tratamento da forma subclínica e leve implica controle dos sintomas associados ao surto agudo e monitoramento da evolução. A forma moderada e grave implica uso de corticosteroides, inicialmente via oral, e pulsoterapia se refratariedade.^452 - 455^ Medicações como iECA, furosemida, espironolactona e digoxina devem ser usadas se IC manifesta.^450^ A refratariedade implica ponderar tratamento cirúrgico valvar na fase aguda ( Tabela 40 ).^456 - 457^

**Tabela 40:** – Tratamento da cardite reumática

Indicações	Classe	Nível de evidência
Erradicação do *Streptococcus* B-hemolítico do grupo A: – Penicilina G benzatina 1.200.000 UI IM profunda dose única para maiores que 20kg – Penicilina G benzatina 600.000 UI IM profunda dose única para menores que 20kg – Amoxicilina 50mg/kg/dia (máximo 1.500mg) divida em 3 doses, por 10 dias – Para alérgicos à penicilina – eritromicina 40mg/kg/dia (máximo 1.000mg) dividida em 4 doses, por 10 dias	I	C^444, 451^
Repouso, se caso moderado ou grave	IIa	C^444^
Hospitalização para controle de sintomas em cardite moderada a grave	IIa	C^444^
Prednisona 0,5 a 1 mg/kg/dia (máximo 50mg) via oral, pode ser dividida em 2 a 3 doses diárias, mantendo por 15 dias, com redução posterior semanal de 20% da dose em casos subclínicos a leves. Tempo total de 4 a 8 semanas.	IIb	B^444,452,453^
Ácido acetilsalicílico 100mg/kg (máximo 3 a 4g) dividido em 4 doses ou Naproxeno 20mg/kg (máximo 1.000mg) dividido em 2 doses para casos subclínicos com artrite e/ou pericardite associada. Tempo total de 2 semanas.	I	B^444,453^
Prednisona 1 a 2 mg/kg/dia (máximo 60mg) via oral, pode ser dividida em 2 a 3 doses diárias, mantendo por 15 dias, com redução posterior semanal de 20% da dose em casos moderados a graves. Tempo total em torno de 12 semanas.	I	B^444,452,453^
Metilprednisolona 30mg/kg/dia em ciclos semanais em casos graves e refratários ao tratamento inicial.	IIb	B^454,455^
Na presença de sinais/sintomas de disfunção ventricular, tratamento medicamentoso da IC com diurético e drogas com atuação neuro-hormonal	I	C^450^
Cirurgia cardíaca valvar em casos graves e refratários: – Plástica valvar mitral com técnica que permita crescimento anel – Prótese mecânica aórtica preferencial	I	B^456,457^

*IC: insuficiência cardíaca.*

## 8. Miocardites por Doenças Autoimunes

O envolvimento cardíaco nas doenças autoimunes pode incluir pericárdio, miocárdio, endocárdio, valvas e coronárias. Dentre as entidades, merecem destaque em relação à miocardite, a sarcoidose, a miocardite de células gigantes, a doença de Behçet, a granulomatose eosinofílica com poliangeíte, o LES, a esclerodermia e a artrite reumatoide. Existe uma evidente limitação em relação ao diagnóstico de miocardite e sua prevalência nas doenças autoimunes, porém devemos considerar essa possibilidade quando da presença de sinais e sintomas sugestivos de acometimento cardíaco, quer seja com arritmias, síncope, IC, dor torácica e elevação de marcadores de necrose miocárdica, especialmente em pacientes com antecedente de doença autoimune ou quando existe acometimento cardíaco associado a sintomas de inflamação atingindo outros sistemas.

A elevação de marcadores inflamatórios inespecíficos, incluindo PCR/VHS e de lesão miocárdica, como troponina e BNP, habitualmente está presente, porém sem especificidade. ECG e ECO devem ser realizados para todos os pacientes com doenças autoimunes na suspeita de acometimento cardíaco.^12 , 188^ A RM pode ser utilizada como método sensível e específico para avaliação de miocardite, além de ampliar o raciocínio em relação a diagnósticos diferenciais.^458 , 459^ Outro método não invasivo tem sido o PET, especialmente em situações de suspeita de sarcoidose.^243^ A solicitação de marcadores de autoimunidade, como FAN, fator reumatoide e ANCA, deve ser considerada e orientada pela suspeita clínica.^460^ A BEM é o padrão-ouro para o diagnóstico de miocardite, quer seja por doenças autoimunes ou outras etiologias; mediante utilização de técnicas além da histologia, consegue diferenciar o acometimento infeccioso em relação ao não infeccioso; além disso, pode identificar a presença de vasculite ou outras doenças miocárdicas não inflamatórias.^151^ O tratamento da miocardite pelas doenças autoimunes foi discutido em outra sessão deste documento.

A elevação de marcadores inflamatórios inespecíficos, incluindo PCR/VHS e de lesão miocárdica, como troponina e BNP, habitualmente está presente, porém sem especificidade. ECG e ECO devem ser realizados para todos os pacientes com doenças autoimunes na suspeita de acometimento cardíaco. ^12 , 188^ A RM pode ser utilizada como método sensível e específico para avaliação de miocardite, além de ampliar o raciocínio em relação a diagnósticos diferenciais. ^458 , 459^ Outro método não invasivo tem sido o PET, especialmente em situações de suspeita de sarcoidose. ^243^ A solicitação de marcadores de autoimunidade, como FAN, fator reumatoide e ANCA, deve ser considerada e orientada pela suspeita clínica. ^460^ A BEM é o padrão-ouro para o diagnóstico de miocardite, quer seja por doenças autoimunes ou outras etiologias; mediante utilização de técnicas além da histologia, consegue diferenciar o acometimento infeccioso em relação ao não infeccioso; além disso, pode identificar a presença de vasculite ou outras doenças miocárdicas não inflamatórias. ^151^ O tratamento da miocardite pelas doenças autoimunes foi discutido em outra sessão deste documento.

## 9. Manejo das Arritmias Cardíacas na Miocardite

### 9.1. Avaliação Não Invasiva e Invasiva das Arritmias na Fase Aguda e Crônica das Diversas Causa das Miocardites

As arritmias cardíacas são manifestações relativamente frequentes no paciente com miocardite, podendo ocorrer em qualquer fase da doença. Os mecanismos arritmogênicos estão direta ou indiretamente relacionados ao grau de agressão inflamatória miocárdica.^55^

Na fase aguda pela agressão viral e da resposta inflamatória, temos miocitólise associada à fibrose, que promovem a hiperatividade do sistema simpático e disfunção dos canais iônicos, especialmente na regulação do cálcio, criando o substrato eletrofisiológico para geração de arritmias.^461^ Quanto maior o dano celular e o grau de comprometimento inflamatório, maior a probabilidade de ocorrência de arritmias ventriculares, sendo a reentrada o principal mecanismo arritmogênico.

Um amplo espectro de bradiarritmias e taquiarritmias ocorre no contexto da miocardite. BAV, alterações da repolarização ventricular e o prolongamento do intervalo QT são achados comuns na fase aguda da doença. Fibrilação atrial e taquicardias atriais também podem estar presentes no curso da miocardite aguda ou na fase crônica.

As arritmias ventriculares podem se manifestar através de extrassístoles e/ou taquicardias ventriculares. Estas podem ter um caráter monomórfico ou polimórfico e apresentarem-se de forma não sustentada ou sustentada (duração ≥30 segundos).

Os sintomas variam de acordo com a forma de apresentação da arritmia, o estado hemodinâmico e o grau de disfunção ventricular esquerda, podendo se manifestar por meio de palpitações, taquicardias, síncope ou morte súbita.

Os métodos diagnósticos diretos utilizados para avaliação não invasiva das arritmias são o ECG basal de 12 derivação, a eletrocardiografia ambulatorial contínua por 24 ou 48 horas (sistema *Holter* ) e o monitoramento de eventos (sistema Looper).

O ECG é geralmente alterado nos pacientes com miocardite, porém tais achados apresentam baixa sensibilidade e especificidade.^462^ Ukena et al.^94^ relataram que a duração prolongada do complexo QRS é um preditor independente para morte cardíaca ou transplante cardíaco em pacientes com suspeita de miocardite. O prolongamento do intervalo QTc acima de 440 ms, o desvio do eixo QRS e a presença de ectopias ventriculares, presentes no curso da miocardite, não parecem ser preditores independentes de pior prognóstico. O ECG é uma ferramenta de grande utilidade na detecção de bradiarritmias e taquiarritmias que se apresentam de forma sustentada.

Para documentação das arritmias que apresentam um caráter paroxístico, o monitoramento de eletrocardiografia ambulatorial pode ser utilizado. A duração do monitoramento depende da frequência dos sintomas e quanto mais esporádico mais difícil a sua documentação.

Através da eletrocardiografia ambulatorial por 24 horas ( *Holter* ), é possível a documentação de arritmias e anormalidades da condução atrioventricular. O *Holter* também nos auxilia na análise da distribuição nictemeral das arritmias, do sistema nervoso autônomo e do provável mecanismo eletrofisiológico.^463^ Recomendamos a realização do *Holter* 24 horas na fase hospitalar para avaliação de possíveis arritmias assintomáticas e anormalidades intermitentes da condução atrioventricular ( Tabela 41 ). O *Holter* também pode ser recomendado na fase crônica da miocardite como método auxiliar para estratificação de risco de morte súbita.^463^

**Tabela 41:** – Recomendações para a avaliação de arritmias na miocardite aguda

Indicação	Classe	Nível de evidência
*Holter* para pacientes de risco prognóstico intermediário e alto	I	C

O real papel da avaliação invasiva por meio do estudo eletrofisiológico para estratificação do risco de morte súbita é ainda ponto de investigação em pacientes com miocardites. Um dos pontos a se considerar é que a reprodutibilidade de eventos arrítmicos significativos deve variar de acordo com a etiologia e o tipo de acometimento miocárdico.^464^ Na sarcoidose cardíaca, por exemplo, encontramos alto grau de reprodutibilidade de eventos clínicos significativos com a estimulação elétrica programada, sendo esta útil na tomada de decisão. Nos pacientes que apresentaram taquicardia ventricular monomórfica não sustentada ou sustentada em algum momento da doença, importante realce tardio ou zonas de baixa voltagem ao estudo eletrofisiológico com mapeamento eletroanatômico parecem ter pior prognóstico, e estes achados podem auxiliar na estratificação do risco de morte súbita.^465^ Na ausência de dados específicos, recomenda-se a utilização cautelosa desse método de estratificação do risco de morte súbita nesses pacientes, especialmente nos assintomáticos.^259^

### 9.2. Tratamento de Arritmias e Prevenção da Morte Súbita na Fase Aguda e Subaguda

As arritmias podem estar associadas à miocardite principalmente na fase aguda, mas também na fase crônica, dependendo do grau de lesão tecidual, nos quais se destacam a inflamação e a fibrose residual, mas com uma base fisiológica ampla^466 , 467^ ( Tabela 42 ). Podem estar presentes em 33,7% dos casos internados por miocardite, se apresentando tanto por taqui, quanto bradiarritmias e estão associados a morbidades como hipertireioidismo, idade, obesidade, IC, desequilíbrio eletrolítico e doença valvar.^95^ A preexistência de cardiomiopatias como displasia arritmogênica do VD e canalopatias preexistentes está também associada à ocorrência de arritmias durante inflamação miocárdica.^468 , 469^

**Tabela 42:** – Mecanismos potencialmente geradores de arritmia em pacientes com miocardites

Lesão viral direta, gerando lise celular miocárdica e resposta imune inata Persistência viral
Apoptose celular
Fibrose favorecendo mecanismos de reentrada Efeito pró-arrítmico das citocinas
Alteração das *gap junctions* celulares Infarto por lesão microvascular

As bradiarritmias são geralmente associadas aos BAV, que podem ser de vários graus e ocorrem predominantemente na fase aguda; mesmo assim, são raras. Obongayo et al.^92^ observaram uma prevalência de 1,7% de BAV, sendo somente 1,1% de bloqueios avançados na fase intra-hospitalar de 31.760 pacientes internados com o diagnóstico de miocardite a partir do banco de dados do Nationawide Inpatient Survey dos EUA. Nos casos de BAV avançado de 3º grau, houve associação com maior morbidade e mortalidade.

A fibrilação atrial pode ocorrer em até 9% dos pacientes com miocardite aguda na fase hospitalar e se associa a maior mortalidade hospitalar (RC:1,7 com IC 95% 1,1-2,7, p = 0.02); choque cardiogênico (RC: 1,9, com IC 95% 1,3-2,8, p < 0.001) e tamponamento cardíaco (RC: 5,6 com IC 95% 1.2-25.3, p = 0.002).^470^

As arritmias ventriculares, as mais associadas à probabilidade de morte súbita, podem corresponder até aproximadamente um quarto de todas as arritmias registradas em pacientes internados por miocardite, sendo a taquicardia ventricular a mais frequente.^95^

O manejo das arritmias na fase aguda deve seguir o princípio de transitoriedade do processo, e as ectopias frequentes ou taquicardias não sustentadas não devem ser tratadas por antiarrítmicos específicos, exceto o betabloqueador, quando indicado. O uso do marca-passo temporário pode ser utilizado em BAV avançados nesta fase, e a indicação de marca-passo definitivo ou cardiodesfibrilador implantado deve seguir as indicações convencionais ( Tabela 43 ).

**Tabela 43:** – Tratamento das arritmias e prevenção da morte súbita relacionadas à miocardite

Indicações	Classe	Nível de evidência
Tratamento com betabloqueador, espironolactona e sacubitril-valsartana para pacientes com disfunção sistólica de VE	I	C
Marca-passo provisório para bradiarritmias sintomáticas e/ou BAV avançado durante a fase aguda da miocardite	I	C
Terapia antiarrítmica com amiodarona na TVNS sintomática ou TV sustentada durante a fase aguda de miocardite	I	C
Implante de CDI na prevenção primária de MS em pacientes com cardiomiopatia dilatada na fase crônica (>6 meses), da miocardite com tratamento clínico otimizado CF II e III, FEVE ≤35% e expectativa de vida de pelo menos 1 ano	IIa	C
Indicação de CDI nas fases aguda e subaguda de miocardite (<6 meses)	III	C
Indicação de agentes antiarrítmicos para prevenção primária de arritmias cardíacas em pacientes com miocardite	III	C

*BAV: bloqueio atrioventricular; CDI: cardiodesfibrilador implantável; CF: classe funcional; FEVE: fração de ejeção de ventrículo esquerdo; MS: morte súbita; TV: taquicardia ventricular; TVNS: taquicardia ventricular não sustentada; VE: ventrículo esquerdo..*

## 10. Avaliação Prognóstica e Seguimento

### 10.1. Marcadores de Prognóstico e Evolução

A miocardite se apresenta com ampla diversidade fenotípica. Grande parte de indivíduos com miocardite aguda, que se apresentam com cardiomiopatia dilatada aguda, evoluem com melhora do quadro em poucos dias.^14^ Relatos de séries apresentam valores entre 10% e 20% de eventos cardiovasculares sérios a longo prazo e risco de recaída de 10%.^109^

Inúmeros fatores têm sido envolvidos no prognóstico, quer sejam clínicos, quer sejam laboratoriais. A manutenção da função ventricular preservada durante o quadro agudo tem sido repetidamente relacionada com melhora espontânea sem sequelas.^15^ Outras análises registram que níveis reduzidos da pressão arterial e da frequência cardíaca, síncope, disfunção sistólica do ventrículo direito, pressão arterial pulmonar elevada assim como classe funcional avançada da New York

Heart Association devem ter papel importante.^94^ A etiologia também tem se mostrado valorosa no espectro prognóstico. Portadores de miocardite linfocítica aguda, que mantiveram função ventricular preservada, evoluíram com melhora espontânea e sem sequelas. Em contraposição, o Myocarditis Treatment Trial registrou que pacientes com IC e FEVE inferior a 45% apresentaram mortalidade de 56% em 4 anos. A miocardite de células gigantes e a eosinofílica evoluem de forma mais sombria.^14^ Portadores de miocardite fulminante apresentam dramático prognóstico a curto prazo; no entanto, quando sobreviventes, apresentaram melhor prognóstico que várias outras etiologias.^17 , 98^

O ECG mostrou valor prognóstico em avaliação recente.^471^ A RM, exame de destacado valor no cenário do diagnóstico da miocardite, já apresentou utilidade com o uso da técnica do realce tardio;^109^ contudo, em mais recente publicação, não confirmou valor preditivo, seja na melhora da função ventricular ou na remodelação em avaliação a longo prazo.^472^ Apesar do avanço no diagnóstico, o prognóstico continua sendo um desafio, provavelmente por inúmeros fatores conhecidos ou não. É possível considerar as causas que variam enormemente com suas peculiaridades, apresentação clínica, envolvimento imunológico e genético, entre outras.^137^

### 10.2 Seguimento Ambulatorial nas Avaliações dos Métodos Complementares

O seguimento clínico acompanhado do ECG deve acontecer continuamente nos pacientes que já apresentaram o diagnóstico. Diante do valor inegável da função ventricular, exames de imagem devem ser incluídos. O ecocardiograma surge como alternativa útil e de mais fácil acesso, trazendo a informação mais relevante nesse cenário ( Tabela 44 ).

**Tabela 44:** – Recomendações gerais de acompanhamento na miocardite473,474

Indicações	Classe	Nível de evidência
Acompanhamento clínico com eletrocardiograma dos pacientes de baixo risco com 1, 3, 6 e 12 meses, e anualmente a seguir	I	C
Acompanhamento por ecocardiograma dos pacientes de baixo risco com 1, 6 e 12 meses, e anualmente a seguir	I	C
Avaliação clínica laboratorial em pacientes com risco intermediário e com *Holter* e exames de imagem aos 1, 3 e 6 meses (ecocardiograma e/ou ressonância de acordo com disponibilidade), e anualmente a seguir	I	C
Entre pacientes portadores de miocardite de alto risco, realizar acompanhamento clínico e laboratorial com *Holter* e exames de imagem com 15 dias, 1, 3 e 6 meses (ecocardiograma ou ressonância de acordo com a disponibilidade), e anualmente a seguir	I	C
