# Supplementary material for: Diretriz de Miocardites da Sociedade Brasileira de Cardiologia – 2022
Source: Arq Bras Cardiol. 2022 Jul 7;119(1):143–211. [Article in Portuguese] doi: 10.36660/abc.20220412 (PMC9352123; doi:10.36660/abc.20220412)
Supplement: Translation

# Translation: Brazilian Society of Cardiology Guideline on Myocarditis – 2022

**Authors:** Marcelo Westerlund Montera, Fabiana G. Marcondes-Braga, Marcus Vinícius Simões, Lídia Ana Zytynski Moura, Fabio Fernandes, Sandrigo Mangine, Amarino Carvalho de Oliveira, Aurea Lucia Alves de Azevedo Grippa de Souza, Bárbara Maria Ianni, Carlos Eduardo Rochitte, Claudio Tinoco Mesquita, Clerio F. de Azevedo, Dhayn Cassi de Almeida Freitas, Dirceu Thiago Pessoa de Melo, Edimar Alcides Bocchi, Estela Suzana Kleiman Horowitz, Evandro Tinoco Mesquita, Guilherme H. Oliveira, Humberto Villacorta, João Manoel Rossi, João Marcos Bemfica Barbosa, José Albuquerque de Figueiredo, Louise Freire Luiz, Ludhmila Abrahão Hajjar, Luis Beck-da-Silva, Luiz Antonio de Almeida Campos, Luiz Cláudio Danzmann, Marcelo Imbroise Bittencourt, Marcelo Iorio Garcia, Monica Samuel Avila, Nadine Oliveira Clausell, Nilson Araujo de Oliveira, Odilson Marcos Silvestre, Olga Ferreira de Souza, Ricardo Mourilhe-Rocha, Roberto Kalil, Sadeer G. Al-Kindi, Salvador Rassi, Silvia Marinho Martins Alves, Silvia Moreira Ayub Ferreira, Stéphanie Itala Rizk, Tiago Azevedo Costa Mattos, Vitor Barzilai, Wolney de Andrade Martins, Heinz-Peter Schultheiss

**Affiliations:** 1 Brasil Hospital Pró-Cardíaco, Rio de Janeiro, RJ – Brasil; 2 Brasil Instituto do Coração (InCor) do Hospital das Clínicas da Faculdade de Medicina da Universidade de São Paulo (HCFMUSP), São Paulo, SP – Brasil; 3 Brasil Faculdade de Medicina de Ribeirão Preto da Universidade de São Paulo, São Paulo, SP – Brasil; 4 Brasil Pontifícia Universidade Católica de Curitiba, Curitiba, PR – Brasil; 5 Brasil Universidade Federal Fluminense,Rio de Janeiro, RJ – Brasil; 6 Brasil Instituto do Coração (InCor) – Faculdade de Medicina da Universidade de São Paulo, São Paulo, SP – Brasil; 7 Brasil Hospital do Coração (HCOR), São Paulo, SP – Brasil; 8 Brasil Hospital Vitória, Rio de Janeiro, RJ – Brasil; 9 EUA Duke University, Durham – EUA; 10 Brasil Universidade Federal do Acre, Rio Branco, AC – Brasil; 11 Brasil Hospital do Coração de Campinas, Campinas, SP – Brasil; 12 Brasil Instituto de Cardiologia do Rio Grande do Sul/Fundação Universitária de Cardiologia,Porto Alegre, RS – Brasil; 13 Brasil Centro de Ensino e Treinamento Edson de Godoy Bueno / UHG, Rio de Janeiro, RJ – Brasil; 14 EUA University Hospitals Cleveland Medical Center, Cleveland, Ohio – EUA; 15 Brasil Instituto Dante Pazzanese de Cardiologia, São Paulo, SP – Brasil; 16 Brasil Universidade do Estado do Amazonas, Boca do Acre, AM – Brasil; 17 Brasil Universidade Federal do Maranhão, São Luís, MA – Brasil; 18 Brasil Instituto do Câncer do Estado de São Paulo da Faculdade de Medicina da Universidade de São Paulo, São Paulo, SP – Brasil; 19 Brasil Hospital de Clínicas de Porto Alegre, Porto Alegre, RS – Brasil; 20 Brasil Universidade Federal do Rio Grande do Sul (UFRGS), Porto Alegre, RS – Brasil; 21 Brasil Universidade Luterana do Brasil, Canoas, RS – Brasil; 22 Brasil Universidade do Estado do Rio de Janeiro, Rio de Janeiro, RJ – Brasil; 23 Brasil Hospital Universitário Pedro Ernesto, Rio de Janeiro, RJ – Brasil; 24 Brasil Hospital Universitário Clementino Fraga Filho (HUCFF) da Universidade Federal do Rio de Janeiro (UFRJ), Rio de Janeiro, RJ – Brasil; 25 Brasil Rede Dor São Luiz de Hospitais, Rio de Janeiro, RJ – Brasil; 26 Brasil Hospital Sírio Libanês, São Paulo, SP – Brasil; 27 EUA Harrington Heart and Vascular Institute, University Hospitals and Case Western Reserve University,Cleveland, Ohio – EUA; 28 Brasil Universidade Federal de Goiás, Goiânia, GO – Brasil; 29 Brasil Pronto Socorro Cardiológico de Pernambuco (PROCAPE), Recife, PE – Brasil; 30 Brasil Universidade de Pernambuco (UPE), Recife, PE – Brasil; 31 Brasil Instituto de Cardiologia do Distrito Federal, Brasília, DF – Brasil; 32 Brasil DASA Complexo Hospitalar de Niterói, Niterói, RJ – Brasil; 33 Alemanha Institute of Cardiac Diagnostics and Therapy (IKDT), Berlim – Alemanha

**Table:** 

Brazilian Society of Cardiology Guideline on Myocarditis – 2022
The report below lists declarations of interest as reported to the SBC by the experts during the period of the development of these statement, 2021.
Expert	Type of relationship with industry
Amarino Carvalho de Oliveira Júnior	Nothing to be declared
Aurea Lucia Alves de Azevedo Grippa de Souza	Other relationships Any economically relevant equity interest in companies in the healthcare or education industry or in any companies competing with or supplying to SBC: - Cardiology: Course - PROKIDS Assistência Multidisciplinar LTDA
Bárbara Maria Ianni	Nothing to be declared
Carlos Eduardo Rochitte	Nothing to be declared
Claudio Tinoco Mesquita	Financial declaration A - Economically relevant payments of any kind made to (i) you, (ii) your spouse/partner or any other person living with you, (iii) any legal person in which any of these is either a direct or indirect controlling owner, business partner, shareholder or participant; any payments received for lectures, lessons, training instruction, compensation, fees paid for participation in advisory boards, investigative boards or other committees, etc. From the brazilian or international pharmaceutical, orthosis, prosthesis, equipment and implants industry: - Bayer: Xarelto. - Servier: Vastarel. B - Research funding under your direct/personal responsibility (directed to the department or institution) from the brazilian or international pharmaceutical, orthosis, prosthesis, equipment and implants industry: - Alnylan: Onpatro.
Clerio F. Azevedo	Nothing to be declared
Dhayn Cassi de Almeida Freitas	Nothing to be declared
Dirceu Thiago Pessoa de Melo	Financial declaration A - Economically relevant payments of any kind made to (i) you, (ii) your spouse/partner or any other person living with you, (iii) any legal person in which any of these is either a direct or indirect controlling owner, business partner, shareholder or participant; any payments received for lectures, lessons, training instruction, compensation, fees paid for participation in advisory boards, investigative boards or other committees, etc. From the brazilian or international pharmaceutical, orthosis, prosthesis, equipment and implants industry: - Daiichi Sankyo. Other relationships Funding of continuing medical education activities, including travel, accommodation and registration in conferences and courses, from the brazilian or international pharmaceutical, orthosis, prosthesis, equipment and implants industry: - Pfizer.
Edimar Alcides Bocchi	Financial declaration A - Economically relevant payments of any kind made to (i) you, (ii) your spouse/partner or any other person living with you, (iii) any legal person in which any of these is either a direct or indirect controlling owner, business partner, shareholder or participant; any payments received for lectures, lessons, training instruction, compensation, fees paid for participation in advisory boards, investigative boards or other committees, etc. From the brazilian or international pharmaceutical, orthosis, prosthesis, equipment and implants industry: - AstraZeneca: ISGLT2; Bayer: ISGLT2, Vericiguat; Boehringer: ISGLT2.
Estela Suzana Kleiman Horowitz	Financial declaration B - Research funding under your direct/personal responsibility (directed to the department or institution) from the brazilian or international pharmaceutical, orthosis, prosthesis, equipment and implants industry: - Jansen: Rivaroxabana. C - Personal research funding paid by the brazilian or international pharmaceutical, orthosis, prosthesis, equipment and implants industry: - Jansen: Rivaroxabana.
Evandro Tinoco Mesquita	Other relationships Employment relationship with the brazilian or international pharmaceutical, orthosis, prosthesis, equipment and implants industry, as well as any employment relationship with health insurance companies or medical audit companies (including part-time jobs) in the year to which your declaration refers: - UnitedHealth Group.
Fabiana G. Marcondes-Braga	Financial declaration A - Economically relevant payments of any kind made to (i) you, (ii) your spouse/partner or any other person living with you, (iii) any legal person in which any of these is either a direct or indirect controlling owner, business partner, shareholder or participant; any payments received for lectures, lessons, training instruction, compensation, fees paid for participation in advisory boards, investigative boards or other committees, etc. From the brazilian or international pharmaceutical, orthosis, prosthesis, equipment and implants industry: - Novartis: Lectures; AstraZeneca: Lectures and Advisory Council; Boehringer: Advisory Council.
Fabio Fernandes	Financial declaration A - Economically relevant payments of any kind made to (i) you, (ii) your spouse/partner or any other person living with you, (iii) any legal person in which any of these is either a direct or indirect controlling owner, business partner, shareholder or participant; any payments received for lectures, lessons, training instruction, compensation, fees paid for participation in advisory boards, investigative boards or other committees, etc. From the brazilian or international pharmaceutical, orthosis, prosthesis, equipment and implants industry: - Pfizer: Tafamidis; Alnylan: Patisiran.
Guilherme H. Oliveira	Nothing to be declared
Heinz-Peter Schultheiss	Nothing to be declared
Humberto Villacorta	Financial declaration A - Economically relevant payments of any kind made to (i) you, (ii) your spouse/partner or any other person living with you, (iii) any legal person in which any of these is either a direct or indirect controlling owner, business partner, shareholder or participant; any payments received for lectures, lessons, training instruction, compensation, fees paid for participation in advisory boards, investigative boards or other committees, etc. From the brazilian or international pharmaceutical, orthosis, prosthesis, equipment and implants industry: - Novartis: Heart failure; Roche: Biomarkers; Servier: Heart failure. C - Personal research funding paid by the brazilian or international pharmaceutical, orthosis, prosthesis, equipment and implants industry: - Roche: GDF-15.
João Manoel Rossi Neto	Financial declaration A - Economically relevant payments of any kind made to (i) you, (ii) your spouse/partner or any other person living with you, (iii) any legal person in which any of these is either a direct or indirect controlling owner, business partner, shareholder or participant; any payments received for lectures, lessons, training instruction, compensation, fees paid for participation in advisory boards, investigative boards or other committees, etc. From the brazilian or international pharmaceutical, orthosis, prosthesis, equipment and implants industry: - Novartis: Lectures; AstraZeneca: Lectures.
João Marcos Bemfica Barbosa	Financial declaration A - Economically relevant payments of any kind made to (i) you, (ii) your spouse/partner or any other person living with you, (iii) any legal person in which any of these is either a direct or indirect controlling owner, business partner, shareholder or participant; any payments received for lectures, lessons, training instruction, compensation, fees paid for participation in advisory boards, investigative boards or other committees, etc. From the brazilian or international pharmaceutical, orthosis, prosthesis, equipment and implants industry: - Novartis: Entresto.
José Albuquerque de Figueiredo Neto	Financial declaration A - Economically relevant payments of any kind made to (i) you, (ii) your spouse/partner or any other person living with you, (iii) any legal person in which any of these is either a direct or indirect controlling owner, business partner, shareholder or participant; any payments received for lectures, lessons, training instruction, compensation, fees paid for participation in advisory boards, investigative boards or other committees, etc. From the brazilian or international pharmaceutical, orthosis, prosthesis, equipment and implants industry: - Novartis: Heart failure.
Lídia Ana Zytynski Moura	Financial declaration A - Economically relevant payments of any kind made to (i) you, (ii) your spouse/partner or any other person living with you, (iii) any legal person in which any of these is either a direct or indirect controlling owner, business partner, shareholder or participant; any payments received for lectures, lessons, training instruction, compensation, fees paid for participation in advisory boards, investigative boards or other committees, etc. From the brazilian or international pharmaceutical, orthosis, prosthesis, equipment and implants industry: - Novartis: Entresto; AstraZeneca: Forxiga. B - Research funding under your direct/personal responsibility (directed to the department or institution) from the brazilian or international pharmaceutical, orthosis, prosthesis, equipment and implants industry: - AstraZeneca: Forxiga.
Louise Freire Luiz	Nothing to be declared
Ludhmila Abrahão Hajjar	Nothing to be declared
Luis Beck-da-Silva	Financial declaration A - Economically relevant payments of any kind made to (i) you, (ii) your spouse/partner or any other person living with you, (iii) any legal person in which any of these is either a direct or indirect controlling owner, business partner, shareholder or participant; any payments received for lectures, lessons, training instruction, compensation, fees paid for participation in advisory boards, investigative boards or other committees, etc. From the brazilian or international pharmaceutical, orthosis, prosthesis, equipment and implants industry: - Novartis: Heart failure; AstraZeneca: Heart failure. B - Research funding under your direct/personal responsibility (directed to the department or institution) from the brazilian or international pharmaceutical, orthosis, prosthesis, equipment and implants industry: - Amgen: Heart failure.
Luiz Antonio de Almeida Campos	Nothing to be declared
Luiz Cláudio Danzmann	Financial declaration A - Economically relevant payments of any kind made to (i) you, (ii) your spouse/partner or any other person living with you, (iii) any legal person in which any of these is either a direct or indirect controlling owner, business partner, shareholder or participant; any payments received for lectures, lessons, training instruction, compensation, fees paid for participation in advisory boards, investigative boards or other committees, etc. From the brazilian or international pharmaceutical, orthosis, prosthesis, equipment and implants industry: - Novartis: Entresto; AstraZeneca: Forxiga; Servier: Procoralan.
Marcelo Imbroise Bittencourt	Financial declaration A - Economically relevant payments of any kind made to (i) you, (ii) your spouse/partner or any other person living with you, (iii) any legal person in which any of these is either a direct or indirect controlling owner, business partner, shareholder or participant; any payments received for lectures, lessons, training instruction, compensation, fees paid for participation in advisory boards, investigative boards or other committees, etc. From the brazilian or international pharmaceutical, orthosis, prosthesis, equipment and implants industry: - GENEONE - DASA: Genetic tests; Sanofi: Enzyme replacement therapy; AstraZeneca: Forxiga.
Marcelo Iorio Garcia	Nothing to be declared
Marcelo Westerlund Montera	Nothing to be declared
Marcus Vinícius Simões	Financial declaration A - Economically relevant payments of any kind made to (i) you, (ii) your spouse/partner or any other person living with you, (iii) any legal person in which any of these is either a direct or indirect controlling owner, business partner, shareholder or participant; any payments received for lectures, lessons, training instruction, compensation, fees paid for participation in advisory boards, investigative boards or other committees, etc. From the brazilian or international pharmaceutical, orthosis, prosthesis, equipment and implants industry: - Novartis: Entresto; AstraZeneca: Dapagliflozina. B - Research funding under your direct/personal responsibility (directed to the department or institution) from the brazilian or international pharmaceutical, orthosis, prosthesis, equipment and implants industry: - Amgen: Omecamtiv/Mecarbil; Beringher Ingelheim: Empagliflozina.
Monica Samuel Avila	Nothing to be declared
Nadine Oliveira Clausell	Nothing to be declared
Nilson Araujo de Oliveira Jr.	Financial declaration A - Economically relevant payments of any kind made to (i) you, (ii) your spouse/partner or any other person living with you, (iii) any legal person in which any of these is either a direct or indirect controlling owner, business partner, shareholder or participant; any payments received for lectures, lessons, training instruction, compensation, fees paid for participation in advisory boards, investigative boards or other committees, etc. From the brazilian or international pharmaceutical, orthosis, prosthesis, equipment and implants industry: - Johnson and Johnson: Catheters for invasive electrophysiology. B - Research funding under your direct/personal responsibility (directed to the department or institution) from the brazilian or international pharmaceutical, orthosis, prosthesis, equipment and implants industry: - Biotronik: Dispositivos de estimulação cardíaca. Other relationships Funding of continuing medical education activities, including travel, a commodation and registration in conferences and courses, from the brazilian or international pharmaceutical, orthosis, prosthesis, equipment and implants industry: - Johnson and Johnson: Catheters for electrophysiology.
Odilson Marcos Silvestre	Nothing to be declared
Olga Ferreira de Souza	Nothing to be declared
Ricardo Mourilhe-Rocha	Financial declaration A - Economically relevant payments of any kind made to (i) you, (ii) your spouse/partner or any other person living with you, (iii) any legal person in which any of these is either a direct or indirect controlling owner, business partner, shareholder or participant; any payments received for lectures, lessons, training instruction, compensation, fees paid for participation in advisory boards, investigative boards or other committees, etc. From the brazilian or international pharmaceutical, orthosis, prosthesis, equipment and implants industry: - AstraZeneca: Dapagliflozina; Boehringer: Empagliflozina; Novartis: Sacubitril/Valsartana. B - Research funding under your direct/personal responsibility (directed to the department or institution) from the brazilian or international pharmaceutical, orthosis, prosthesis, equipment and implants industry: - PROADI/SUS: Telemedicina; Boehringer: Empagliflozina.
Roberto Kalil Filho	Nothing to be declared
Sadeer G. Al-Kindi	Nothing to be declared
Salvador Rassi	Financial declaration A - Economically relevant payments of any kind made to (i) you, (ii) your spouse/partner or any other person living with you, (iii) any legal person in which any of these is either a direct or indirect controlling owner, business partner, shareholder or participant; any payments received for lectures, lessons, training instruction, compensation, fees paid for participation in advisory boards, investigative boards or other committees, etc. From the brazilian or international pharmaceutical, orthosis, prosthesis, equipment and implants industry: - Novartis: Entresto; Servier: Procoralan B - Research funding under your direct/personal responsibility (directed to the department or institution) from the brazilian or international pharmaceutical, orthosis, prosthesis, equipment and implants industry: - Novartis: Entresto; Servier: Procoralan; Boehringer Ingelheim: Jardiance. Other relationships Funding of continuing medical education activities, including travel, accommodation and registration in conferences and courses, from the brazilian or international pharmaceutical, orthosis, prosthesis, equipment and implants industry: - Novartis: Entresto; Servier: Procoralan.
Sandrigo Mangine	Financial declaration A - Economically relevant payments of any kind made to (i) you, (ii) your spouse/partner or any other person living with you, (iii) any legal person in which any of these is either a direct or indirect controlling owner, business partner, shareholder or participant; any payments received for lectures, lessons, training instruction, compensation, fees paid for participation in advisory boards, investigative boards or other committees, etc. From the brazilian or international pharmaceutical, orthosis, prosthesis, equipment and implants industry: - Novartis: Sacubitril/Valsartan; Pfizer: Rare deseases. Other relationships Funding of continuing medical education activities, including travel, accommodation and registration in conferences and courses, from the brazilian or international pharmaceutical, orthosis, prosthesis, equipment and implants industry: - Pfizer: Rare deseases.
Silvia Marinho Martins Alves	Nothing to be declared
Silvia Moreira Ayub Ferreira	Financial declaration A - Economically relevant payments of any kind made to (i) you, (ii) your spouse/partner or any other person living with you, (iii) any legal person in which any of these is either a direct or indirect controlling owner, business partner, shareholder or participant; any payments received for lectures, lessons, training instruction, compensation, fees paid for participation in advisory boards, investigative boards or other committees, etc. From the brazilian or international pharmaceutical, orthosis, prosthesis, equipment and implants industry: - Abbott: Mitraclip; Novartis: Entresto. Other relationships Funding of continuing medical education activities, including travel, accommodation and registration in conferences and courses, from the brazilian or international pharmaceutical, orthosis, prosthesis, equipment and implants industry: - Abbott: Heartmate II e HeartMate 3.
Stéphanie Itala Rizk	Nothing to be declared
Tiago Azevedo Costa Mattos	Nothing to be declared
Vitor Barzilai	Nothing to be declared
Wolney de Andrade Martins	Financial declaration A - Economically relevant payments of any kind made to (i) you, (ii) your spouse/partner or any other person living with you, (iii) any legal person in which any of these is either a direct or indirect controlling owner, business partner, shareholder or participant; any payments received for lectures, lessons, training instruction, compensation, fees paid for participation in advisory boards, investigative boards or other committees, etc. From the brazilian or international pharmaceutical, orthosis, prosthesis, equipment and implants industry: - Bayer: Cardio-oncology.

## Content

1. Epidemiology 149

2. Definition and etiology 150

2.1. Genetic factors in the etiopathogenesis of myocarditis 151

3. Pathophysiology 151

4. Diagnostic evaluation 152

4.1. Diagnostic criteria for suspected myocarditis 152

**4.1.1. Diagnostic evaluation flowchart** 152

4.2. Clinical evaluation: suspected clinical situations 152

4.3. Biomarkers 154

**4.3.1. Laboratory markers of inflammatory injury** 154

**4.3.2. Laboratory markers for etiopathogenic investigation** 155

4.4. Electrocardiogram 155

**4.4.1. Diagnostic criteria for electrocardiogram/Holter/stress testing** 156

**4.4.2. Prognosis** 157

4.5. Echocardiogram 157

4.6. Cardiac magnetic resonance imaging 157

4.7. Nuclear medicine 159

**4.7.1. Single-photon emission computed tomography (SPECT) radiotracers** 159

**4.7.2. Positron emission tomography (PET) radiotracers** 160

**4.7.3. Additional perspectives** 160

4.8. Coronary computed tomography angiography and coronary angiography 161

4.9. Endomyocardial biopsy: indications, technique, and complications 162

**4.9.1. Considerations for indication** 162

**4.9.2. Prognosis** 162

**4.9.3. Technique** 162

**4.9.4. Complications** 163

4.10. Histological analysis and viral screening – Molecular biology and genome 164

**4.10.1. Histological analysis** 164

**4.10.2. Immunohistochemical analysis** 164

**4.10.3. Gene expression profile analysis** 164

**4.10.4. Viral analysis** 164

5. Treatment 164

5.1. Therapeutic flowcharts 164

5.2. Immunosuppression: indications and types 166

5.3. Antiviral therapy: indications and types 167

5.4. Immunomodulation (immunoglobulin and immunoadsorption): indications and types of immunoglobulins 168

**5.4.1. Immunoadsorption** 169

5.5. Conventional cardioprotective therapy 169

**5.5.1. No ventricular dysfunction** 169

**5.5.2. Ventricular dysfunction and hemodynamic stability** 169

**5.5.3. Hemodynamically unstable patients with ventricular dysfunction: therapeutic approach** 169

5.6. General recommendations: physical activity and vaccination 169

6. Special situations 171

6.1. Fulminant myocarditis 171

**6.1.1. Diagnostic evaluation** 172

**6.1.2. Therapeutic approach** 172

6.2. Sarcoidosis 172

**6.2.1. Diagnosis** 172

**6.2.2. Treatment and prognosis** 173

**6.2.3. Prognosis** 175

6.3. Giant cell myocarditis 176

**6.3.1. Treatment** 176

**6.3.2. Clinical manifestation and diagnosis** 177

6.4. Acute Chagasic myocarditis and reactivation 176

**6.4.1. Clinical manifestations and modes of transmission, reactivation in immunosuppressed patients** 176

**6.4.2. Diagnosis** 176

**6.4.3. Treatment** 179

6.5. Myocarditis due to tropical diseases 179

6.6. Covid-19-related myocarditis 180

**6.6.1. Possible pathophysiology of SARS-CoV-2-related myocarditis** 180

**6.6.2. Direct viral myocardial injury** 180

**6.6.3. Diagnosis of Covid-19-related myocarditis** 182

**6.6.4. Laboratory** 182

**6.6.5. Electrocardiogram** 182

**6.6.6. Imaging** 183

**6.6.7. Endomyocardial biopsy** 183

6.7. Acute cardiotoxicity of antineoplastic therapy 184

**6.7.1. Antineoplastic agents inducing acute cardiotoxicity** 184

**6.7.2. Diagnosis of acute cardiotoxicity** 185

**6.7.3. Treatment of acute cardiotoxicity** 186

**6.7.4. Prognosis** 187

**6.7.5. Prevention** 187

6.8. Myocarditis in children and adolescents 188

**6.8.1. Causal factors** 188

**6.8.2. Prognosis** 188

6.9. Myocarditis with pericardial involvement 191

**6.9.1. Diagnosis and treatment** 191

6.10. Acute myocarditis mimicking MI 191

7. Rheumatic carditis 192

8. Myocarditis due to autoimmune diseases 195

9. Management of cardiac arrhythmias in myocarditis 195

9.1. Noninvasive and invasive assessments of arrhythmias in the acute and chronic phases of the several causes of myocarditis 195

9.2. Arrhythmia treatment and sudden death prevention in the acute and subacute phases 196

10. Prognostic evaluation and follow-up 197

10.1. Prognosis and evolution markers 197

10.2. Outpatient follow-up with additional evaluations 197

References 197

## 1. Epidemiology

The actual incidence of myocarditis is difficult to determine because the clinical presentations are highly heterogeneous and a large number of cases develop subclinically. Another contributing factor is the very low frequency of use of endomyocardial biopsy (EMB), the gold standard for diagnosis.^1^

A review of several postmortem studies addressing young victims of unexplained sudden death has showed that the incidence of myocarditis varies widely, accounting for up to 42% of cases.^2^The Global Burden of Disease Study 2013 has used the International Classification of Diseases coding in regional and global statistical analyses regarding 187 countries and estimated the annual incidence of myocarditis to be approximately 22 cases per 100,000 patients treated.^3^In cohorts of patients with dilated cardiomyopathy of undefined etiology, EMB-proven myocarditis has been detected in up to 16% of adult patients^4^and up to 46% of pediatric patients.^5^

Many studies have reported a higher prevalence of acute myocarditis in men compared to women.^6 , 7^Some studies have suggested that the most common clinical manifestation in adults is lymphocytic myocarditis; their median age is 42 years, while patients with giant cell myocarditis have a median age of 43 years.^8^However, newborns and children more typically exhibit fulminant myocarditis and are more susceptible to virus-induced pathogenicity compared to adults.^9 , 10^

Myocarditis has a wide prognostic spectrum depending on the severity of initial clinical symptoms and etiology. Patients with mild symptoms and no ventricular dysfunction often show spontaneous resolution and excellent prognosis.^11^However, approximately 30% of severe cases of EMB-proven myocarditis with associated ventricular dysfunction are expected to progress to dilated cardiomyopathy and heart failure (HF) with a poor prognosis. In pediatric patients, prognosis appears to be worse: 10-year heart transplant-free survival can be as low as 60%.^5^

## 2. Definition and etiology

Myocarditis is defined as an inflammatory disease of the myocardium that should be diagnosed by histological, immunological, and immunohistochemical criteria. Histological criteria include evidence of inflammatory infiltrates within the myocardium together with cardiomyocyte degeneration and necrosis of nonischemic origin. Quantitative immunohistochemical criteria to identify an abnormal inflammatory infiltrate, indicative of active myocarditis, are leukocyte count ≥14 cells/mm^2^, including up to 4 monocytes/mm^2^, with presence of CD3-positive T lymphocytes ≥7 cells/mm^2^.^12^

Additionally, depending on cell type, the type of inflammatory infiltrate observed on histological diagnosis is used to classify myocarditis as lymphocytic, eosinophilic, polymorphic, giant cell myocarditis, or cardiac sarcoidosis.^13^

Myocarditis is caused by a wide variety of infectious agents, including viruses, protozoans, bacteria, chlamydiae, rickettsiae, fungi, and spirochetes ( Table 1 ). It may also be triggered by noninfectious mechanisms in toxic myocarditis (drugs, heavy metals, radiation) and by autoimmune and hypersensitivity mechanisms (eosinophilic myocarditis, collagenosis, virus-induced disease, heart transplant rejection).^14 , 15^

**Table 1:** – Etiology of acute myocarditis*

1 – Infectious myocarditis
Viral
RNA viruses	Coxsackieviruses A and B, echovirus, poliovirus, influenza A and B viruses, respiratory syncytial virus, mumps virus, measles virus, rubella virus, hepatitis C virus, dengue virus, yellow fever virus, Chikungunya virus, Junin virus, Lassa fever virus, rabies virus, human immunodeficiency virus-1
DNA viruses	Adenoviruses, parvovirus B19, cytomegalovirus, human herpesvirus 6, Epstein-Barr virus, varicella-zoster virus, herpes simplex virus, variola virus, vaccinia virus
Bacterial	*Staphylococcus, Streptococcus, Pneumococcus, Meningococcus, Gonococcus, Salmonella, Corynebacterium diphtheriae, Haemophilus influenzae, Mycobacterium (tuberculosis), Mycoplasma pneumoniae, Brucella*
Spirochetal	*Borrelia* (Lyme disease), *Leptospira* (Weil disease)
Fungal	*Aspergillus, Actinomyces, Blastomyces, Candida, Coccidioides, Cryptococcus, Histoplasma, Mucormycoses, Nocardia, Sporothrix*
Protozoal	*Trypanosoma cruzi, Toxoplasma gondii, Entamoeba, Leishmania*
Parasitic	*Trichinella spiralis, Echinococcus granulosus, Taenia solium*
Rickettsial	*Coxiella burnetii* (Q Fever), *R. Rickettsii* (Rocky Mountain spotted fever), *R. tsutsugamushi*
**2 – Immune-mediated myocarditis**
Allergens	Tetanus toxoid, vaccines, serum sickness Drugs: penicillin, cefaclor, colchicine, furosemide, isoniazid, lidocaine, tetracycline, sulfonamides, phenytoin, phenylbutazone, methyldopa, thiazide diuretics, amitriptyline
Alloantigens	Heart transplant rejection
Autoantigens	Infection-negative lymphocytic myocarditis, infection-negative giant cell miocarditis associated with autoimmune disorders: systemic lupus erythematosus, rheumatoid arthritis, Churg-Strauss syndrome, Kawasaki disease, inflammatory bowel disease, scleroderma, polymyositis, myasthenia gravis, insulin-dependent diabetes mellitus, sarcoidosis, Wegener granulomatosis, rheumatic fever, immuno-oncology (immune checkpoint inhibitors)
**3 – Toxic myocarditis**
Drugs	Amphetamines, anthracyclines, cocaine, cyclophosphamide, ethanol, fluorouracil, lithium, catecholamines, hemetine, trastuzumab, clozapine, interleukin-2, immune checkpoint inhibitors
Heavy metals	Copper, iron, lead
Miscellaneous	Scorpion sting, snake and spider bites, bee and wasp stings, carbon monoxide, inhalants, phosphorus, arsenic, sodium azide
Hormones	Pheochromocytoma
Physical agents	Radiation, electric shock

Source: *Adapted from Caforio et al.^5^

Viral infection is the most prevalent trigger of myocarditis, particularly in children. The most common cardiotropic viruses are enterovirus, parvovirus B19 (B19V), adenovirus, influenza A virus, human herpesvirus (HHV), Epstein-Barr virus, cytomegalovirus, hepatitis C virus, and human immunodeficiency virus (HIV). Some evidence suggests that there may be regional differences in the prevalence of viral agents, with a predominance of adenoviruses, parvoviruses, and herpesviruses in the European population^16^and enteroviruses in the American population.^17^However, these epidemiological differences may be partially explained by outbreaks of specific viral infections occurring over the years across different regions of the world as well as variations in viral detection techniques. Thus, the actual influence of geographic factors on cardiotropic viral infections remains controversial.^18^

In South America, especially some regions of Brazil, Chagasic myocarditis caused by *Trypanosoma cruzi* is one of the most prevalent causes of acute myocarditis, with particular importance after a recent report of outbreak of cases associated with oral transmission in the Brazilian Amazon.^19^Systemic autoimmune diseases such as Churg-Strauss syndrome and hypereosinophilic syndrome are associated with eosinophilic myocarditis. Giant cell myocarditis and sarcoidosis are rare but clinically significant because, if diagnosis is made early, there is specific treatment that may ensure an improved prognosis.^20 , 21^

Autoimmune myocarditis may develop with exclusive cardiac involvement or with systemic manifestations in the setting of autoimmune diseases. The most frequent diseases are sarcoidosis, hypereosinophilic syndrome, scleroderma, and systemic lupus erythematosus.

New immunotherapies for cancer treatment may be associated with risk of myocarditis. Cases linked to immune checkpoint inhibitors, such as nivolumab and ipilimumab, have been recently reported.^22 - 24^

### 2.1. Genetic factors in the etiopathogenesis of myocarditis

In classic descriptions of the etiopathogenesis of myocarditis, evidence of mechanisms involving viral action and autoimmune reaction is well documented. Little is said about genetic predisposition. Many authors believe that genetic phenomena are likely to contribute to the development of viral and/or autoimmune myocarditis.^12 , 25^

Laboratory data consistent with this hypothesis have been documented in a study of 342 family members of patients with dilated cardiomyopathy. The presence of cardiac antibodies was found to be higher in that group compared to the control group.^26^

The likelihood of a complex interaction between genetic (linked to individual predisposition) and nongenetic (linked to the offending agent) causes in the ultimate progression to dilated cardiomyopathy is also widely recognized. The problem is that the scientific evidence supporting such hypothesis is limited.^27^

There is evidence that, in susceptible mouse strains, infection and inflammation trigger autoimmune reactions in the heart, generally as a result of myocyte necrosis and subsequent release of autoantigens previously hidden in the immune system. The same strains of genetically predisposed animals develop lymphocytic or autoimmune giant cell myocarditis and then dilated cardiomyopathy after immunization with cardiac autoantigens (eg, cardiac myosin).^28^

Evidence also suggests that myocarditis may be present in specific cardiomyopathies (eg, arrhythmogenic cardiomyopathy) leading to changes in the phenotype and abrupt progression of the disease. In this context, some mutations may increase the susceptibility to myocarditis.^29^

Nonetheless, in general, myocarditis is still classified as a nonfamilial acquired disorder, with evidence from experimental studies indicating that genetic changes may provide greater susceptibility to this disease.

## 3. Pathophysiology

In simple terms, the pathophysiology of myocarditis can be divided into infectious and noninfectious. Infectious myocarditis is the most common form and includes a wide range of viruses, bacteria, protozoans, fungi, and other rare pathogens (see Table 1 ). Viruses are the most commonly involved and experimentally studied agents. In noninfectious myocarditis, autoimmunity is present through specific diseases, drugs, and autoantibodies; genetic predisposition plays an important role in both (see Table 1 ).

Murine models suggest that the development of viral myocarditis has three phases: acute (a few days), subacute (a few weeks to months), and chronic (development of dilated cardiomyopathy);^30^also, two pathogenic mechanisms are described: direct cytopathic effect of the virus and virus-induced anticardiac immune response.

Phase 1 corresponds to initial infection, which may heal without sequelae, or lead to HF or death, or progress to phases 2/3.^31^In most patients with viral myocarditis, the pathogen is eliminated and the immune system reduces activity with no further complications. However, in a minority of patients, the virus is not eliminated and causes persistent myocardial injury and inflammation secondary to antibody production.^17^Thus, viral myocarditis could be considered a precursor of dilated cardiomyopathy, with progression having been observed in 21% of patients within 3 years.^32^

Enteroviruses, especially coxsackievirus B3 (CVB3), initiate myocarditis by attaching to the coxsackievirus and adenovirus receptor (CAR) and decay accelerating factor (DAF), culminating in cell death by apoptosis^33^or necroptosis.^34^Infected cardiomyocytes are then lysed, which results in cytosolic release of proteins and viral products. After the acute phase, the course of the disease depends on genetic basis and includes two possibilities: progression to dilated cardiomyopathy or resolution.^35 - 39^Coxsackievirus infection activates innate and adaptive immune responses, initially including the production of interferon and activation of toll-like receptors.^40^In the adaptive response, T- and B-cell deficiency leads to viral persistence and clinical deterioration.^41 , 42^

Another important aspect is the production of specific autoantibodies to cardiomyocytes, which is based on the release of cardiac peptides with molecular mimicry between cardiac proteins and viral agents. In the presence of costimulatory cytokines such as tumor necrosis factor (TNF) and interleukin (IL)-1, these antibodies promote an effector T-cell response.^43^

Other viruses such as B19V and HHV-6 have been increasingly described in cardiac biopsies, while enteroviruses and adenoviruses have shown a downward trend.^44^However, these viruses have also been detected in hearts without myocarditis or cardiomyopathies of other etiologies, making the interpretation of the association between presence of infectious agents in cardiac tissue and development of myocarditis more complex. Another finding has been the persistent influence of these agents on clinical outcomes.^45^

Regarding noninfectious myocarditis, animal models of autoimmune myocarditis with genetically susceptible strains have demonstrated the presence of CD4+ T cells reactive to autoantigens, such as myosin heavy chain, in the absence of infectious agents.^46^In addition to lymphocyte autoimmune responses, macrophage responses have been observed in cases of granulomatous myocarditis and eosinophilic myocarditis in situations of hypersensitivity.

Giant cell myocarditis is an autoimmune form of myocardial damage characterized histologically by an infiltrate of multinucleated giant cells as well as an infiltrate of T cells, eosinophils, and histiocytes. The marked presence of (cytotoxic) CD8 cells together with the release of inflammatory cytokines and oxidative stress mediators leads to intense myocyte damage and replacement by fibrosis, culminating in rapid loss of ventricular function and unfavorable clinical outcomes. Twenty percent of patients exhibit an association with autoimmune diseases such as Hashimoto thyroiditis, rheumatoid arthritis, myasthenia gravis, Takayasu arteritis, and others.^47^Sarcoidosis affects multiple systems, including the lungs in 90% of cases, and is associated with the accumulation of T lymphocytes, mononuclear phagocytes, and noncaseating granulomas in involved tissues.^48 , 49^

In drug-induced myocarditis, the time to hypersensitivity response varies from hours to months. Hypersensitivity is partly explained by a response to chemically reactive components that bind to proteins promoting structural changes. These particles are phagocytosed by defense cells, sometimes macrophages, which present them on the surface of these cells to T cells. Cytokines such as IL-5, which stimulates eosinophils, are then released as a delayed hypersensitivity response. This accumulation of IL-5 promotes major eosinophilic infiltration with increased hypersensitivity response and severe myocardial injury. Genetic predisposition appears to favor this response pattern.^50^

Hypereosinophilic syndrome may be associated with several systemic diseases, such as Churg-Strauss syndrome, cancer, and parasitic and helminthic infections, or with vaccinations. These can produce an intense inflammatory response in the myocardium, leading to cell damage with dysfunction and HF.^51 , 52^Pathophysiologically, similar to what happens in other organs, there is intense eosinophilic infiltration in the myocardium promoting the release of potent mediators of myocyte damage, leading to necrosis and loss of myocardial structure. These mediators include eosinophil-derived neurotoxin, eosinophil cationic protein, and eosinophilic protease. Also, the production of inflammatory cytokines such as IL-1, TNF-alpha, IL-6, IL-8, IL-3, IL-5, and macrophage inflammatory proteins promotes myocyte injury and loss with progression to myocardial dysfunction.^53^

More recently, nivolumab, an antitumor drug that acts as a checkpoint inhibitor, has been considered a cause of lymphocytic myocarditis. A possible pathophysiological mechanism suggests that myocardial cells could share antigens with tumor cells, thus being targets for activated T cells, resulting in inflammatory infiltration and development of HF and conduction disorders.^54^

## 4. Diagnostic evaluation

### 4.1. Diagnostic criteria for suspected myocarditis

Clinical suspicion of myocarditis as proposed by the European Society of Cardiology (ESC) Working Group on Myocardial and Pericardial Diseases is based on the association of clinical presentation with abnormal test results suggestive of myocardial inflammatory injury.^12 , 55^

By analyzing the most frequent clinical presentations of myocarditis and the diagnostic accuracy of additional evaluations for prognosticating myocardial inflammation, we propose that clinically suspected myocarditis be stratified into three levels: low, intermediate, and high diagnostic suspicion ( Figure 1 ).^32 , 56 - 63^These suspicion criteria have been established by expert consensus and require further validation by clinical registries or multicenter studies.

**Figure 1:** – Algorithm for diagnostic stratification of clinically suspected myocarditis.

AVB: atrioventricular block; BNP: brain natriuretic peptide; CMR: cardiac magnetic resonance; CRP: C-reactive protein; ECG: electrocardiogram; ECHO: echocardiogram; LGE: late gadolinium enhancement; NSVT: nonsustained ventricular tachycardia; PR: PR segment; ST: ST segment; VT: ventricular tachycardia.

#### 4.1.1. Diagnostic evaluation flowchart

Our flowchart for diagnostic evaluation of myocarditis is based on the degree of clinical and prognostic suspicion (see Figure 1 ). Patients with low clinical suspicion have a favorable prognosis and, during clinical follow-up, are evaluated regarding the need for noninvasive coronary artery disease (CAD) stratification. Patients with intermediate clinical suspicion and favorable course undergo the same line of clinical follow-up and diagnostic investigation as low-risk patients. Patients with maintained or deteriorated clinical status, ventricular dysfunction , arrhythmias, or atrioventricular (AV) block should undergo coronary angiography and EMB. Patients with high diagnostic suspicion generally have a poor prognosis and should undergo coronary angiography and EMB for establishing etiology and then defining a specific treatment to improve the prognosis.^32 , 56 , 64 , 65^

## 4.2. Clinical evaluation: suspected clinical situations

Myocarditis manifests through different forms, ranging from mild and oligosymptomatic to severe cases associated with ventricular arrhythmias, hemodynamic instability, and cardiogenic shock. Sudden death is rare (8.6% to 12%) and affects mostly children and young adults.^66 , 67^

The most common situation consists of young patients with chest pain suggestive of acute myocardial infarction (MI) with normal coronary arteries after respiratory or intestinal viral infection, although viral symptoms do not always precede myocarditis (10% to 80%).^68 - 70^Despite being predominant in young patients, the syndrome may appear at any age. Subclinical myocarditis, transient troponin elevation, or electrocardiographic changes may also occur after an acute viral infection consisting of nonspecific manifestations such as fever, myalgia, and respiratory or gastrointestinal symptoms.^68 , 71^

Myocarditis has different presentations, which are described below:^12 , 71 , 72^

1. Clinical condition similar to acute coronary syndrome (chest pain, electrocardiographic changes suggestive of ischemia; elevated myocardial necrosis markers with normal coronary arteries).
2. Acute new symptoms of HF (3 days to 3 months) in the absence of coronary heart disease or known cause of symptoms.
3. New-onset HF symptoms within the past months (>3 months) in the absence of coronary heart disease or known cause of symptoms.
4. Life-threatening conditions: unexplained ventricular arrhythmias, and/or syncope, and/or aborted sudden death; cardiogenic shock without associated coronary heart disease.

## A) Manifesting as chest pain

Patients with chest pain may present with different electrocardiographic changes, such as ST-segment elevation or depression, T-wave inversion, or pathological Q waves. Segmental changes on Doppler echocardiography and elevated myocardial necrosis markers, especially troponin, in patients with normal coronary arteries are suggestive of myocarditis.^68 , 73^In most studies, these patients have a good short-term prognosis, and the degree of ventricular impairment is predictive of risk of death.^71 , 74^A minority develops persistent and recurrent myopericarditis with normal left ventricular function that may respond to colchicine.^75^

## B) Manifesting as acute heart failure

Presentation may be acute, associated with the onset of HF symptoms within days, but also subacute/chronic, associated with new-onset cardiomyopathy in a patient with no apparent cause for abnormal myocardial function.

Myocarditis presenting as HF symptoms (dyspnea, fatigue, exercise intolerance) may be associated with mild impairment of ventricular function (left ventricular ejection fraction [LVEF]: 40% to 50%) that improves within weeks to months. However, a small number of patients may have significant ventricular dysfunction (LVEF <35%) and, of those, 50% develop chronic LV dysfunction; approximately 25% will need a heart transplant or ventricular assist device, while the remaining 25% will have improved ventricular function over the course of follow-up; a minority of cases may progress to cardiogenic shock and require mechanical circulatory support.^68 , 76 - 79^The risk of death or need for transplantation is strongly associated with the degree of hemodynamic compromise and left and right ventricular dysfunction, which may respond to standard drug treatment for HF.^80^

Fulminant presentation of the disease is characterized by sudden onset (days) of symptoms of advanced HF. These patients generally have severe ventricular dysfunction with minor changes in ventricular diameters. This is a dramatic presentation that requires early intervention.^68 , 81^When fulminant condition is associated with persistent ventricular tachycardia or no response to standard therapy, the prognosis is poor, and more severe forms of myocarditis, such as giant cell myocarditis, should be considered and investigated.^8^

## C) Manifesting as chronic or progressive heart failure

Myocarditis confirmed by immunohistopathological criteria is found in up to 40% of patients with chronic cardiomyopathy who remain symptomatic despite drug treatment. The presence of inflammation shown by histology is associated with a poor prognosis.^71^

## D) Manifesting as a life-threatening condition

- Arrhythmias or conduction disorders

Patients with myocarditis may also present with conduction disorders, such as second- or third-degree or complete AV block, especially those with echocardiographic signs of hypertrophy due to interstitial edema.^82^The presence of heart block or symptomatic or sustained ventricular arrhythmias in patients with cardiomyopathy should raise suspicion for myocarditis with a definite cause (Lyme disease; sarcoidosis; arrhythmogenic right ventricular dysplasia, or Chagas disease in endemic areas).^71^

- Cardiogenic shock

A small subgroup of patients presenting with sudden onset of HF within 2 weeks of viral infection may need inotropic and/or mechanical circulatory support. Ventricular function recovery generally occurs when they survive the initial condition, but adequate therapy should be initiated as early as possible.^71 , 81^

Table 2 summarizes the main clinical syndromes of suspected myocarditis and suggests possible agents responsible for each presentation of the disease.^83^

**Table 2:** – Description of clinical presentations and possible causes of the different clinical syndromes of myocarditis

Clinical syndrome	Clinical manifestations	Possible causes
Acute chest pain	Angina symptoms; CAD ruled out; ST/T-segment change; arrhythmias; intermittent troponin I/T and NT-proBNP elevations	Parvovirus B19 or other cardiotropic viruses associated or not with pericarditis
Acute HF	Dyspnea; edema; LV systolic and/or diastolic dysfunction; ECG change; intermittent troponin I/T and NT-proBNP elevations	Viral or nonviral myocarditis or inflammatory cardiomyopathy
Chronic HF	All HF symptoms for some time; CAD ruled out; ECG changes such as LBBB, RBBB, AVB; intermittent troponin I/T and NT-proBNP elevations	Viral or nonviral focal myocarditis or inflammatory cardiomyopathy
Life-threatening HF/arrhythmia	Cardiogenic shock; NYHA class III/IV HF; elevated troponin and NT-proBNP; severe arrhythmia; CAD ruled out	Giant cell myocarditis, eosinophilic myocarditis, toxic myocarditis

*AVB: atrioventricular block; CAD: coronary artery disease; ECG: electrocardiogram HF: heart failure. LBBB: left bundle branch block; LV: left ventricular; RBBB: right bundle branch block; ST/T: ST segment and T wave.*

## 4.3. Biomarkers

### 4.3.1. Laboratory markers of inflammatory injury

No single biomarker is sufficient to diagnose myocarditis; however, some may be useful as prognostic markers. The most commonly used biomarkers are described below.

- **Inflammatory markers.** Leukocyte count, erythrocyte sedimentation rate (ESR), and C-reactive protein (CRP) may be high in patients with myocarditis. However, they are nonspecific and thus have no diagnostic value.
- **Troponins.** Troponins are more specific than creatine phosphokinase (CPK) and creatine kinase MB (CKMB) for myocardial damage and are often high in patients with myocarditis. ^84^ However, normal troponins do not exclude the diagnosis. Although they are not sufficient to establish the diagnosis, troponins may be suggestive of myocarditis, as long as obvious causes such as acute MI and acute HF have been excluded. In a small study investigating several biomarkers, troponins were predictive of the diagnosis of biopsy-proven myocarditis with an area under the curve of 0.87, a sensitivity of 83%, and a specificity of 80%. ^85^ Troponin is useful for diagnosing myocarditis in patients with acute-onset cardiomyopathy.^12 , 72^
- **Natriuretic peptides.** Brain natriuretic peptide (BNP) and NT-proBNP might be high in myocarditis. ^86^ However, they are not useful for diagnostic confirmation, as different causes of HF may be responsible for their elevation. Nonetheless, they may be prognostic markers. In a study of biopsy-proven myocarditis, only NT-proBNP above the fourth quartile (>4,245 pg/mL) among several biomarkers was predictive of death or heart transplantation. ^85^

### 4.3.2. Laboratory markers for etiopathogenic investigation

**Viral serologies.** Viral serologies are of limited value in diagnosing myocarditis, as IgG antibodies to cardiotropic viruses are highly prevalent in the general population in the absence of viral heart disease. A study has found no correlation between viral serology and biopsy findings.^87^In specific situations, serological screening for hepatitis C, HIV in high-risk individuals, and Lyme disease in endemic areas might be useful. Screening with serological markers should be dictated by high clinical suspicion for that disease ( Table 3 ).

**Table 3:** - Recommendations for initial laboratory evaluation of myocarditis

Indications	Class	Level of evidence
Use of inflammatory markers for the diagnosis of myocarditis	I	C
Myocardial injury biomarkers to aid in the diagnosis of myocarditis	I	B
BNP or NT-proBNP to aid in the diagnosis and prognostic stratification of myocarditis	I	B
Serological screening and/or antigen detection and/or PCR for the diagnosis of Covid-19 in suspected cases	I	B
Serological screening and/or antigen detection and/or RT-PCR for the initial evaluation of special cases of suspected myocarditis due to specific etiologies	IIa	C
Viral serologies in the routine evaluation of all cases of myocarditis	III	C

*BNP: brain natriuretic peptide; RT-PCR: polymerase chain reaction.*

**Immunohistochemical markers and viral genome analysis.** These markers are superior to the Dallas criteria and, therefore, can be useful for an etiological diagnosis. The complication rate of EMB is low ( Table 3 ).^88 - 90^

## 4.4. Electrocardiogram

An electrocardiogram (ECG) is commonly ordered to screen for myocarditis despite of its limited specificity, although patients frequently present with some ECG change.^12^Sinus tachycardia is possibly the most common form of presentation on ECG.^14^Some ECG changes are more suggestive of myocarditis than others. For example, ST-T segment elevation is typically concave in myocarditis (rather than convex in myocardial ischemia), diffuse without reciprocal changes, transient, and reversible during the course of the disease ( Figure 2 ).^91^

**Figure 2:** – Course of early repolarization pattern in acute myocarditis.

Adapted from Oka et al.^91^

An early repolarization electrocardiographic (ER-ECG) pattern in some patients with acute myocarditis may be the evidence of inflammation/edema in the LV epicardium. Oka et al.^91^showed that the ER-ECG pattern in acute myocarditis was transient and reversible, and was not associated with a poor prognosis.

AV block in the presence of mild LV dilatation may be due to a number of causes (including laminopathy) but may also be suggestive of Lyme disease, cardiac sarcoidosis, or giant cell myocarditis. Ogunbayo et al. reported that, among 31,760 patients with primary diagnosis of myocarditis, heart block was found in 540 (1.7%) − 21.6% were first-degree, 11.2% were second-degree, and 67.2% were high-degree. High-degree AV block was independently associated with increased morbidity and mortality.^92^

A recent meta-analysis showed that prolonged QRS duration was an early characteristic of fulminant myocarditis.^93^In a study of patients acutely admitted with myocarditis without previous HF who underwent EMB, prolonged QRS duration was an independent predictor of cardiac death or heart transplantation.^94^

A significant proportion of patients with acute myocarditis experience sudden cardiac death presumably due to cardiac arrhythmia. A recent study conducted by Adegbala et al. reported a total of 32,107 admissions for acute myocarditis between 2007 and 2014 in the United States. Of those, 10,844 (33.71%) patients had arrhythmias, with ventricular tachycardia (22.3%) and atrial fibrillation (26 .9%) being the most common types, and their presence had an impact on mortality.^95^

In sum, an ECG is a convenient tool for risk stratification and initial screening, but its diagnostic value is poor.^14^

### 4.4.1. Diagnostic criteria for electrocardiogram/Holter/stress testing ^12^

The Twelve-lead ECG is a common practice in diagnostic investigation and prognostic evaluation of myocarditis ( Table 4 ). The changes most often associated with myocarditis on Twelve-lead ECG and/or Holter and/or stress testing with any of the following: first- to third-degree AV block or bundle branch block, ST/T-wave change (ST elevation or non-ST elevation, T-wave inversion), sinus arrest, ventricular tachycardia or fibrillation and asystole, atrial fibrillation, reduced R-wave height, intraventricular conduction delay (widened QRS complex), abnormal Q waves, low voltage, frequent premature beats, and supraventricular tachycardia.

**Table 4:** - Recommendations for performing an electrocardiogram in the evaluation of myocarditis

Indications	Class	Level of evidence
Electrocardiogram in suspected myocarditis	I	C
Electrocardiogram for prognostic evaluation in myocarditis	I	C

### 4.4.2. Prognosis

Prolonged QRS duration, high-degree AV block, ventricular tachycardia, and atrial fibrillation increase mortality.

## 4.5. Echocardiogram

An echocardiogram has a limited role in the diagnosis of myocarditis. It is a highly important tool for excluding other diseases and should always be performed in clinically suspected cases ( Table 5 ).^96 , 97^There is no specific echocardiographic finding, and the changes will only reflect myocardial inflammation. Therefore, findings may range from segmental changes (differential diagnosis with ischemic heart diseases) to diffuse changes (global hypokinesia of one or both ventricles).^98 , 99^When involvement is acute and severe, the ventricular chambers are small (not dilated), and myocardial edema (increased wall thickness) and pericardial effusion, which are common findings in fulminant myocarditis, are present. Right ventricular (RV) involvement usually reflects a poor prognosis.^100^

**Table 5:** – Recommendations for performing an echocardiogram in the initial evaluation of myocarditis

Indications	Class	Level of evidence
Echocardiogram for evaluation of cardiac structure and function	I	C
Echocardiogram for prognostic evaluation and stratification	I	C
Echocardiogram to guide endomyocardial biopsy	IIa	C

Interestingly, echocardiography can be a useful adjunct to EMB not only for locating the ideal site for removing the specimens but also for guiding the interventionist and avoiding complications ( Table 5 ).^101^

## 4.6. Cardiac magnetic resonance imaging

In the evaluation of patients with myocarditis, similar to the evaluation of other nonischemic cardiomyopathies, cardiac magnetic resonance (CMR) imaging is very useful for determining ventricular morphological and functional parameters. In fact, it has been extensively validated to quantify both LV and RV volume, mass, and function, and is currently considered the diagnostic gold standard for this evaluation. Because of its high spatial and temporal resolution as well as its three-dimensional nature making it independent of geometric assumptions, CMR has excellent accuracy and reproducibility, characteristics that are especially useful for longitudinal patient follow-up.^102^

However, the greatest value of CMR in the evaluation of patients with suspected or confirmed myocarditis lies in the ability to provide detailed tissue characterization. It thus allows the identification of inflammatory myocardial injury in the acute and subacute phases as well as scarring that is frequently present in the chronic phase of the disease. The CMR techniques classically used in the characterization of myocardial injury in patients with myocarditis are T2-weighted imaging and late gadolinium enhancement.^103 - 108^

On T2-weighted images, the greater the water content in a given tissue, the higher the signal intensity. Therefore, this technique assesses myocardial edema secondary to the inflammatory process in patients with acute myocarditis (known as edema imaging).^102 - 105^Late gadolinium enhancement, in turn, identifies regional necrosis in acute or subacute myocarditis as well as regional fibrosis in chronic myocarditis.^106 , 108 - 110^It is worth noting that the late enhancement pattern of myocarditis is very different from that of acute MI. The main difference is that late gadolinium enhancement in infarction always enhances the subendocardium. Transmural enhancement may also be present, but the subendocardial layer is always enhanced. In myocarditis, mesoepicardial enhancement is most often present, while the endocardium is usually spared. Furthermore, while enhanced regions in infarction tend to be unique, homogeneous, and distributed across the coronary territories, enhanced regions in myocarditis tend to be multifocal, heterogeneous, and sparse, not restricted to the coronary territories.

The original 2009 Lake Louise criteria (LLC)^105^were based on three CMR techniques. In addition to T2-weighted imaging (edema imaging) and late gadolinium enhancement, both mentioned above, it also included a technique called early gadolinium enhancement. The latter was eventually excluded from the updated diagnostic criteria after a study demonstrated that it did not add incremental diagnostic value to the other techniques. In fact, early gadolinium enhancement had not been clinically used in most CMR centers in the world.

Recently, new CMR techniques capable of measuring myocardial longitudinal (T1) and transverse (T2) relaxation times have been introduced as potentially sensitive and specific methods for the detection of myocardial inflammation.^111^T1 and T2 values are generally measured on a pixel-by-pixel basis and presented as parametric maps, the so-called myocardial T1 and T2 mapping. A T1 map can be acquired before contrast injection (native T1) and 15 to 20 minutes after contrast injection (when gadolinium concentration is relatively steady), thus allowing the calculation of myocardial extracellular volume (ECV). A T2 map is usually acquired only before contrast administration.

The introduction of T1 and T2 mapping was the key reason for a recent LLC update regarding the use of CMR in the diagnosis of myocarditis. According to the new consensus recommendations,^104^this diagnosis is based on the presence of two main criteria that may or may not be associated with supportive criteria ( Table 6 ). The first main diagnostic criterion aims to identify the presence of myocardial edema using T2-based techniques: (1) T2-weighted imaging (edema imaging) and/or (2) T2 mapping. The second main diagnostic criterion also allows the detection of myocardial edema, but its primary objective is to identify the presence of necrosis, fibrosis, and capillary leakage. This second main diagnostic criterion uses T1-based techniques: (1) late gadolinium enhancement and/or (2) T1 mapping (native T1 or ECV).

**Table 6:** – Diagnostic criteria for myocarditis, myopericarditis, or perimyocarditis

Updated Lake Louise criterion 1 POSITIVE T2 CRITERION + 1 POSITIVE T1 CRITERION	Diagnostic target
**MAIN CRITERIA**
**Imagem baseada no T2**	
Increased regional signal intensity in the LV (visual analysis) or	AND
Increased global signal intensity – ratio ≥2 or
Regional or global increase in T2 times (T2 mapping)	EDEMA
**T1-based imaging**
Regional or global increase in T1 times (T1 mapping) or ECV or	Increased T1 = edema (intra- or extracellular), hyperemia, capillary leakage, necrosis, fibrosis Increased ECV = edema (extracellular), hyperemia, capillary leakage, necrosis, fibrosis
Areas with increased signal intensity in a nonischemic distribution pattern on late enhancement imaging	Late gadolinium enhancement = necrosis, fibrosis
**SUPPORTIVE CRITERIA**
Pericardial effusion on cine MRI or increased signal intensity of the pericardium on late enhancement imaging, T1 mapping, or T2 mapping	Pericardial inflammation
LV wall motion abnormality on cine MRI	LV dysfunction

*LV: left ventricular; MRI: magnetic resonance imaging; ECV: extracellular volume.*

The new 2018 diagnostic criteria for myocarditis, myopericarditis, or perimyocarditis are listed in Table 6 .^104^

The accuracy of CMR in patients with suspected myocarditis in the original LLC has been estimated at 78% (sensitivity, 67% and specificity, 91%).^105^These estimates have been subsequently confirmed in a meta-analysis that has demonstrated an accuracy of 83%, a sensitivity of 80%, and a specificity of 87%.^112^Similarly, a more recent meta-analysis has shown a sensitivity of 78% and a specificity of 88%, with an area under the curve (AUC) of 83%.^113^There are no consistent data on the accuracy of CMR using the new diagnostic LLC yet. However, a recent study including only 40 patients with acute myocarditis has demonstrated a sensitivity of 88% and a specificity of 96% for CMR using the new revised criteria (see Table 6 ).^114^

Recommendations for the use of CMR in the diagnostic and prognostic evaluation of patients with suspected acute myocarditis are summarized in Table 7 .^57 , 104 , 109 , 114 - 116^

**Table 7:** – Recommendations for the use of cardiac magnetic resonance imaging in the diagnostic evaluation of patients with suspected acute myocarditis

Indications	Class	Level of evidence
Evaluation of patients with elevated markers of myocardial necrosis and normal coronary arteries on angiography	I	B
Evaluation of patients with dilated cardiomyopathy and suspected myocarditis with a course >6 months, aiming to aid in the etiological investigation, exclude possible differential diagnoses, and provide prognostic information	I	B
Reassessment in 4 weeks for patients with intermediate or high prognostic risk after the acute episode, aiming to distinguish uncomplicated from complicated courses	IIa	B

Based on the body of scientific evidence accumulated since the first version of this SBC guideline, we can now introduce CMR more accurately in the decision-making framework for patients with suspected myocarditis according to the risk stratification approach proposed in Table 8 .^109 , 115 , 117^This approach should be integrated into broad risk stratification criteria that include clinical presentation and additional testing.

**Table 8:** – Risk stratification and likelihood of indication for endomyocardial biopsy based on cardiac magnetic resonance (CMR) parameters.

Prognostic risk	CMR parameter	Suggested approach	Indication for biopsy
Low	No changes in T1 and T2 No ventricular dysfunction	Clinical follow-up	No indication
Intermediate	Positive T1 or T2 Nonextensive late enhancement (<17 g and 13% of LV mass) Normal function or mild LV dysfunction	Clinical follow-up, repeat CMR at 1, 3, and 6 months	Stable: no indication Progressive dysfunction: possible indication
High	Positive T1 or T2 Extensive late gadolinium enhancement (>17 g or 13% of LV mass), or interventricular septal involvement, and/or moderate or severe LV dysfunction	Clinical follow-up, repeat CMR at 1, 3, and 6 months	Possible indication

*LV: left ventricular.*

## 4.7. Nuclear medicine

Nuclear medicine has played an increasing role in the evaluation of patients with myocarditis. New radiotracers and other technologies have allowed a whole new spectrum of contributions to the management of patients with suspected inflammatory myocardial diseases.

The pathophysiological changes of the different types of myocarditis will form the basis for the use of nuclear medicine techniques: the inflammatory process leading to myocardial injury is characterized by infiltration of lymphocytes and macrophages in the myocardium, by increased vascular permeability and increased glucose consumption at the inflammation site, and by cell necrosis with reduced tissue perfusion compared to intact myocardium. These characteristics will translate into increased uptake of gallium-67 citrate in the myocardium (especially useful in cases of sarcoidosis), increased accumulation of^18^F-fluorodeoxyglucose (^18^F-FDG), and reduced myocardial perfusion seen with the tracers technetium (^99m^Tc) sestamibi or thallium-201. Table 9 lists the most commonly used radiotracers for evaluating myocarditis.

**Table 9:** – Nuclear medicine tests frequently used in patients with suspected or confirmed myocarditis

Nuclear medicine test	Main indications	Advantages	Disadvantages
Gallium-67 scintigraphy	Myocarditis and sarcoidosis	Widely available	Less sensitive
^18^F-FDG PET	Sarcoidosis, lupus myocarditis, unexplained cardiac arrhythmias	Highly sensitive, used for monitoring response to treatment	Less available, higher costs
^123^I-mIBG scintigraphy	Assesses risk of ventricular arrhythmias	Identifies patients at risk of sudden death	Less available

*^18^F-FDG PET:^18^F-fluorodeoxyglucose positron emission tomography.*

### 4.7.1. Single-photon emission computed tomography (SPECT) radiotracers

Gallium-67 citrate is an established tracer for nuclear medicine imaging of infections that binds to inflammatory cells at sites of increased vascular permeability thanks to its characteristic binding to iron transport proteins such as lactoferin and leukocyte lysosomes. Gallium-67 has low sensitivity (36%) for detecting myocarditis in patients with new-onset dilated cardiomyopathy and thus should not be routinely indicated ( Table 10 ).^118^The only type of myocarditis with a high positive yield for gallium-67 scintigraphy is sarcoidosis, in which giant cell granulomas are particularly avid for radiotracer retention. A positive gallium-67 scan is considered a major criterion for the diagnosis of cardiac sarcoidosis by the Heart Rhythm Society (HRS) expert consensus statement.^119^Another significant finding in patients with cardiac sarcoidosis is a perfusion defect caused by myocardial microvascular constriction in the vessels surrounding the granulomas. A perfusion defect seen at rest may disappear on stress imaging, a pattern called reverse distribution that may be associated with sarcoidosis.

**Table 10:** - Recommendations for the use of nuclear medicine imaging in the diagnostic evaluation of patients with suspected acute myocarditis

Indications	Class	Level of evidence
^18^F-FDG PET to aid in the diagnosis of myocarditis	IIa	B
Gallium-67 scintigraphy to aid in the diagnosis of myocarditis	IIb	B

*^18^F-FDG PET:^18^F-fluorodeoxyglucose positron emission tomography.*

Gallium-67 scintigraphy can be used as an alternative for patients without access to or with a contraindication to gadolinium-enhanced magnetic resonance imaging (MRI) (claustrophobia, contrast allergy, renal failure) and may contribute to cases of suspected myocarditis based on clinical criteria (fever, recent history of respiratory or intestinal infection, elevated necrosis markers). It is also useful for the differential diagnosis between acute MI with normal coronary arteries and myocarditis, according to a study conducted by Hung et al.,^120^in which scans were positive when performed early after symptom onset.^120^Some patients with myocarditis may present with regional damage in the myocardium, which can be the etiology of arrhythmias. Consequently, gallium-67 scans may demonstrate focal accumulation in ventricular areas and even in the atria alone.^121^

### 4.7.2. Positron emission tomography (PET) radiotracers

^18^F-FDG is taken up by inflammatory cells and actively transported independently of insulin action. Thus, when adequate suppression of myocardial glucose uptake is achieved,^18^F-FDG PET becomes a sensitive tool for the diagnosis of myocardial inflammation and monitoring of treatment response ( Table 10 ).

Most studies on the use of^18^F-FDG PET in myocarditis have focused on cardiac sarcoidosis, and a recent meta-analysis has demonstrated a sensitivity of 84% and a specificity of 83%.^122^For^18^F-FDG PET to be helpful in sarcoidosis or other inflammatory cardiac conditions such as myocarditis, infective endocarditis, or transplant rejection, adequate patient preparation is crucial to prevent circulating insulin from leading to noninflammatory^18^F-FDG accumulation in the myocardium. The most commonly indicated preparation protocols include prolonged fasting (12 to 18 hours) before radiotracer injection and a high-fat, protein-permitted diet, while the utility of heparin remains unclear.^123 , 124^Diagnostic evidence of inflammatory activity is focal^18^F-FDG uptake in the myocardium, while the presence of^18^F-FDG uptake in the RV and the presence of inflammatory uptake in hypoperfused areas, the so-called mismatches, have prognostic significance, as they reveal increased metabolism with reduced perfusion.^124 18^F-FDG PET is also used to monitor treatment response in cardiac sarcoidosis and to assess the activity of extracardiac disease. A monitoring algorithm is proposed in Figure 3 , adapted from Young et al.^125^

**Figure 3:** – Proposed algorithm for diagnosis and monitoring of treatment response in cardiac sarcoidosis. 18F-FDG PET: 18F-fluorodeoxyglucose positron emission tomography.

Adapted from Young et al.^125^

CMR is the standard diagnostic technique for myocarditis not associated with sarcoidosis. Increased signal intensity of T2-weighted images (edema), increased early gadolinium enhancement (hyperemia), and late myocardial gadolinium uptake (late enhancement for necrosis) have a combined sensitivity of 67% and specificity of 91% for the diagnosis of myocarditis. However, there are frequent limitations to proper use of the technique, such as poor signal quality on T2-weighted images, artifacts, and inability to use gadolinium contrast media. In such cases,^18^F-FDG PET is quite useful as a complement to the diagnostic investigation and is available in PET-CT systems or, more recently, in PET-MRI systems.^126^PET-MRI studies have shown that PET is superior to MRI in identifying areas of active cardiac inflammation.^127^

^18^F-FDG PET-CT has been successfully used to identify active inflammation in conditions such as systemic lupus erythematosus,^128^giant cell myocarditis,^129^scleroderma,^130^and even rheumatic carditis.^131^Recently,^18^F-FDG PET has been increasingly used in the investigation of cardiac sarcoidosis and chronic myocarditis, including Chagas disease, as a cause of ventricular arrhythmias.^132^It is also useful for investigating conduction disorders, especially in individuals below 50 years of age with AV block; in these patients, PET has identified several cases of sarcoidosis and even cardiac tuberculosis as a cause of conduction disorder.^133^In a study conducted by Tung et al., 50% of patients with unexplained myocardiopathy and ventricular arrhythmias had a positive^18^F-FDG PET scan indicating the presence of myocarditis not suspected by other techniques.^134^

### 4.7.3. Additional perspectives

New radiotracers have been evaluated in patients with myocardial inflammation, such as gallium-68 DOTATATE, which has an affinity for somatostatin receptors that are expressed by inflammatory cells. Another radiotracer under analysis is^123^I-mIBG, which assesses cardiac presynaptic adrenergic innervation. Although this radiotracer does not directly identify an inflammatory state, it bears an important relationship to increased risk of ventricular arrhythmias, particularly in patients with chronic Chagasic myocarditis, demonstrating viable myocardial areas that are denervated and thus more vulnerable to sustained ventricular tachycardia.^135^

## 4.8. Coronary computed tomography angiography and coronary angiography

Acute myocarditis may mimic acute MI with typical chest pain, ECG abnormalities similar to ST- or non-ST-segment elevation MI, high cardiac enzymes, and hemodynamic instability.^136^

In suspected myocarditis with infarct-like presentation, CAD must be excluded by percutaneous or tomographic coronary angiography. Routine coronary angiography should also be performed during the investigation of a new dilated cardiomyopathy.^137^

An analysis of 46 publications evaluating the underlying pathophysiology of myocardial infarction with nonobstructive coronary arteries (MINOCA) has revealed a typical infarct on CMR in only 24% of patients, myocarditis in 33%, and no significant abnormality in 26%.^138^Young age and high C-reactive protein (CRP) were associated with myocarditis, while male sex, treated hyperlipidemia, high troponin ratio, and low PCR were associated with true MI.^139^

Because patients with acute myocarditis mimicking ST-segment elevation MI have a favorable prognosis, correct diagnosis must be established to prevent unnecessary and potentially hazardous treatments.^139^

Cardiac computed tomography (CT) is a simple and rapid examination that provides a comprehensive assessment of the characteristics of coronary arteries and myocardial tissue. In practice, first-pass CT acquisition allows the evaluation of coronary anatomy and LV enhancement. Delayed CT acquisition is performed 3 to 5 minutes later without any reinjection of contrast medium, allowing iodine uptake to be seen on late contrast-enhanced images in a similar fashion as CMR.^140 , 141^

Computed tomography angiography and CMR have their own and unique ways to avoid invasive coronary angiography, exclude (significant) CAD, and detect other diseases such as acute aortic dissection, pulmonary embolism, myocarditis, and stress cardiomyopathy.^142^

The wide availability of CT combined with the possibility of ruling out acute coronary syndrome (ACS) with coronary CT angiography during the same examination makes it promising for refining acute myocarditis imaging ( Table 11 ).^141^

**Table 11:** – Indication for coronary computed tomography angiography in the diagnostic evaluation of patients with suspected acute myocarditis

Indication	Class	Level of evidence
Coronary computed tomography angiography for exclusion of severe obstructive coronary heart disease in myocarditis investigation as an alternative to coronary angiography in patients with low or intermediate pretest probability of CAD	I	C

*CAD: coronary artery disease.*

In children with suspected myocarditis and Kawasaki disease, CT angiography can be used in the assessment of coronary artery abnormalities.^143^

The latest ESC guideline suggests that, in the absence of angiographically significant CAD (stenosis ≥50%) or preexisting conditions that could explain the clinical setting, patients who have at least one of the five clinical presentations (acute chest pain; acute or worsening HF with ≤3 months of dyspnea, fatigue, and/or signs of HF; chronic HF with >3 months of dyspnea, fatigue, and/or signs of HF; palpitations, symptoms of unexplained arrhythmias and/or syncope and/or aborted death; unexplained cardiogenic shock) and/or certain supportive diagnostic tests (ECG, Holter, troponin, ventricular function abnormalities and edema and/or late gadolinium enhancement with a classic myocardial pattern) should be considered as having “clinically suspected myocarditis” and should undergo an additional evaluation.^12 , 72^

## 4.9. Endomyocardial biopsy: indications, technique, and complications

Histopathological analysis of myocardial tissue is an important diagnostic and prognostic tool in patients with myocarditis. EMB with standard histopathological (Dallas criteria)^144^and immunohistochemical criteria is the current gold standard for diagnosis of myocarditis.^137^

The Dallas criteria alone have limitations by virtue of a high degree of interobserver variability in pathological interpretation and an inability to detect noncellular inflammatory processes, and yields a diagnosis in approximately 10% to 20% of patients.^15^Therefore, according to the WHO definition, immunohistochemistry with a panel of monoclonal and polyclonal antibodies is mandatory to identify the different inflammatory components.^145 , 146^

Viral genome analysis of diseased myocardium, when coupled with immunohistochemical analysis, has improved the diagnostic accuracy and prognostic utility of EMB.^147^Viral screening for enteroviruses, influenza viruses, adenoviruses, cytomegalovirus, Epstein-Barr virus, B19V, and HHV is recommended.

However, as some viral genomes (eg, B19V) can be detected in normal hearts and ischemic and valvular heart diseases,^148^complementary use of virus-specific mRNAs may be required to define active infection.^149^

### 4.9.1. Considerations for indication

Early EMB in severe clinical presentations provides important information to the differential diagnosis of specific types of myocarditis (giant cell, allergic, eosinophilic, sarcoidosis) leading to different treatments (eg, immunosuppressants) and prognoses ( Table 12 ).^150^

**Table 12:** - Recommendations for the use of endomyocardial biopsy

Indications	Class	Level of evidence
New-onset HF (<2 weeks), undefined cause, unresponsive to standard treatment, with hemodynamic deterioration	I	B
New-onset HF (2 weeks to 3 months), undefined cause, associated with ventricular arrhythmia or second- or third-degree atrioventricular block	I	B
In the presence of clinically suspected severe lymphocytic myocarditis, giant cell myocarditis, necrotizing eosinophilic myocarditis	I	B
HF with onset >3 months and <12 months, undefined cause, unresponsive to optimized standard therapy	IIa	C
HF due to dilated cardiomyopathy of any duration with suspected allergic reaction and/or eosinophilia	IIa	C
Frequent ventricular arrhythmias with or without symptoms, undefined cause	IIb	C
Clinical suspicion supported by noninvasive diagnostic methods of myocarditis	IIb	C

*HF: heart failure.*

It is also used in the differential diagnosis of diseases that may mimic myocarditis (arrhythmogenic right ventricular cardiomyopathy, Takotsubo cardiomyopathy, peripartum cardiomyopathy, inflammatory/storage disorders).^150^

Currently, the main indication for EMB consists of patients with new-onset HF (less than 2 weeks) accompanied by a severe clinical presentation (hemodynamic instability, use of inotropic or mechanical circulatory support, being refractory to medical treatment) or high-risk arrhythmias (sustained or symptomatic ventricular arrhythmias or high-degree heart blocks) ( Table 12 ).^151 , 152^

However, it is known that the previous recommendations were notably based on the Dallas criteria, whose diagnostic, prognostic, and therapeutic value is limited. With the use of immunohistochemical and viral genome analyses, there is a growing trend towards a more liberal application of EMB in clinically suspected myocarditis regardless of pattern and severity of presentation.^12^

However, the value of EMB is questionable in patients who have low-risk syndromes and respond to standard treatment with no prospect of therapeutic or prognostic implications. Finally, in intermediate-risk syndromes, EMB should be considered in case of maintenance or worsening of symptoms, ventricular dysfunction, arrhythmias, conduction disorders ( Figure 4 ).^153^

**Figure 4:** – Therapeutic flowchart for myocarditis based on clinical suspicion and prognosis.

ACEI: angiotensin-converting enzyme inhibitor; ARB: angiotensin receptor blocker; AVB: atrioventricular block; CAD: coronary artery disease; CMR: cardiac magnetic resonance; CT: computed tomography; ECG: electrocardiogram; ECHO: echocardiogram; EMB: endomyocardial biopsy.

### 4.9.2. Prognosis

While the Dallas criteria are not an accurate predictor of clinical outcomes, immunohistological evidence of myocardial inflammation is associated with an increased risk of cardiovascular death and need for heart transplantation.^153^

In giant cell myocarditis, the severity of necrosis and fibrosis is associated with an increased risk of death and transplantation.^154^

The absence or presence of residual enteroviral genomes in repeated samples has correlated with progression to end-stage cardiomyopathy, while spontaneous viral clearance has been associated with improved systolic function.^155^

### 4.9.3. Technique

The procedure should be performed in a catheterization laboratory by an experienced interventional cardiologist. Local anesthesia is used with conscious sedation if required, always under the supervision of an anesthesiologist.

EMB is safely performed under direct fluoroscopy guidance and should be supported by echocardiography, which will serve as a guide for correct positioning of the bioptome to avoid puncturing the RV free wall.

CMR is particularly useful for facilitating a guided approach, given its ability to distinguish normal from diseased myocardium, and has been assessed to increase predictive values.^155^

There are no comparative studies to recommend RV or LV biopsy; however, LV EMB should be carefully analyzed in cases of restricted or predominant LV disease.

Samples should be obtained from the RV, especially the distal portion of the interventricular septum and the apical trabecular component, avoiding the RV free wall. The number of samples will depend on the studies to be performed. In the investigation of viral myocarditis, 10 samples should be used (6 for viral screening, 2 for hematoxylin-eosin staining, and 2 for immunohistochemistry). In the investigation of infiltrative or deposition diseases, 6 specimens are required (2 for hematoxylin-eosin staining, 2 for immunohistochemistry, and 2 for electron microscopy). Hematoxylin-eosin staining and immunohistochemistry samples should be placed in a 10% buffered formalin vial and should not be refrigerated. Viral screening samples should be placed in Eppendorf® microtubes (without embedding medium), and these should be placed in containers with dry ice to be quickly transferred to -70-degree refrigerators for storage. Electron microscopy samples should be placed in Eppendorf® tubes with optimal cutting temperature compound.

EMB may be repeated if required to monitor response to etiology-directed therapy or if sampling error is suspected in patients with unexplained HF progression.^156^

### 4.9.4. Complications

Although conventional EMB is considered a safe procedure, different complications have been reported.

In experienced centers, the major complication rate for EMB is <1%, which is similar to that of coronary angiography.^97^The use of echocardiography combined with fluoroscopy significantly reduces the risk of inadvertent puncture that could cause myocardial perforation or coronary artery injury.^155^

Complications of vascular access and sheath insertion can be distinguished from complications of the biopsy procedure. Complications of vascular access are accidental arterial puncture, prolonged bleeding, hematoma, and vascular dissection.

Commonly described complications of the biopsy procedure include vasovagal reaction, AV block of varying degrees, RV free wall perforation, pneumothorax, interventricular septal perforation, access site hematoma, intracardiac fistulas, retroperitoneal hematoma (femoral access), pericardial effusion, thrombus displacement, cardiac tamponade, tricuspid valve chordal rupture, and ventricular arrhythmias.^157^

In sum, the risk of EMB depends on the patient’s clinical status, the operator’s experience, and all the technologies available to prevent, diagnose, and manage complications.

## 4.10. Histological analysis and viral screening – Molecular biology and genome

### 4.10.1. Histological analysis

Myocarditis is defined as an inflammatory disease of the myocardium that should be diagnosed by histological and immunohistological criteria. According to the Dallas criteria, active myocarditis is histologically defined as an inflammatory infiltrate of the myocardium with necrosis of adjacent myocytes, whereas borderline myocarditis is diagnosed when an inflammatory infiltrate is present but no injury/necrosis is demonstrated in the cardiac cells.^158^

However, the Dallas criteria are considered diagnostically inadequate in patients with clinically suspected myocarditis because of variability in interpretation, lack of prognostic value, and low sensitivity due to sampling error. These limitations can be overcome by immunohistological staining of infiltrating cells (leukocytes/T lymphocytes/macrophages) and surface antigens (intercellular cell adhesion molecule-1 [ICAM-1]/human leukocyte antigen [HLA-DR]).

In addition to the diagnosis of myocarditis, histopathological evaluation using histological criteria is key to classifying myocarditis as lymphocytic, eosinophilic, giant cell, granulomatous, and/or polymorphic, which generally reflect different etiopathogeneses of the inflammatory process.^12^

Furthermore, histological examination of paraffin sections by different staining protocols (hematoxylin-eosin, elastica-van Gieson [EvG], periodic acid-Schiff [PAS], Azan) is used to detect myocardial cell death, scarring, fibrosis, dysfunction, cardiomyocyte abnormalities, and pathological vascular conditions. Amyloidosis, iron and glycogen deposition, and other storage diseases can be excluded or specified by additional staining.

### 4.10.2. Immunohistochemical analysis

Immunohistochemistry has significantly increased the sensitivity of EMB and provides prognostic information. The diagnostic accuracy of immunohistology for detecting inflammation is greater than that of histological criteria. Immunohistochemical evaluation is based on specific antigen-antibody reaction analysis. A count ≥14 leukocytes/mm^2^with the presence of T lymphocytes ≥7 cells/mm^2^was considered a realistic cutoff point to achieve the diagnosis of myocarditis.^12^

Quantification of additional infiltrating cells, including macrophages (Mac-1/CD69), CD4+, CD8+ cells, and cytotoxic cells (perforin), and quantification of HLA-DR and ICAM-1 are mandatory to further characterize inflammatory cell populations. Thus, accurate characterization and quantification of myocardial inflammation is relevant for establishing a prognosis and identifying different markers of virus-negative, infectious, chronic/acute autoimmune myocarditis (see Figure 4 ).

Additional immunofluorescence staining methods should be used to define humoral rejection on heart transplant EMB, such as C3d and C4d staining, or to obtain amyloid subtyping.

### 4.10.3. Gene expression profile analysis

Idiopathic giant cell myocarditis and cardiac sarcoidosis are rare disorders causing acute HF with cardiogenic shock and/or life-threatening ventricular arrhythmias in the absence of other etiologies. Prognosis is extremely poor, with 4-year survival rates of less than 20%.^159^

A major barrier to correct diagnosis is sampling error by histological examination of EMB. Distinct differential gene expression profiles have been identified and allowed a clear discrimination between tissues harboring giant cells and those with active myocarditis or inflammation-free controls. Also, disease-specific gene expression profiles change during effective treatment and are suitable for therapy monitoring.^160^

### 4.10.4. Viral analysis

Microbial genomes are determined, quantified, and sequenced by polymerase chain reaction (PCR)-based methods, including nested RT-PCR and quantitative PCR assays, providing viral load analysis. Sequencing of the amplified viral gene product is mandatory.

Importantly, all viruses that may cause the disease should be analyzed. The most commonly reported cardiotropic viral genomes in the myocardium are B19V, enterovirus, adenovirus, influenza virus, HHV-6, Epstein-Barr virus, cytomegalovirus, hepatitis C virus, and HIV ( Table 13 ).

**Table 13:** - Common viruses on endomyocardial biopsy

Vírus type	
Adenovirus	Parvovirus B19
Arbovirus	Poliomyelitis virus
Arenavirus	Rabies virus
Coronavirus	Respiratory syncytial virus
Coxsackievirus (A, B)	Rubella virus
Cytomegalovirus	Vaccinia virus
Dengue virus	Varicella-zoster virus
Echovirus	Variola virus
Encephalomyocarditis virus	Zika virus
Epstein-Barr virus	Human immunodeficiency virus
Hepatitis B virus	Influenza virus (A, B)
Hepatitis C virus	Metapneumovirus
Herpes simplex virus	Mumps virus
Human herpesvirus 6	

B19V is the predominant cardiotropic virus in myocarditis. The clinical impact on the heart is still a matter of debate. Transcriptionally active cardiotropic B19V with positive replicative intermediates on EMB appears to be clinically relevant because patients with myocarditis characterized by such virus have abnormal gene expression compared to control patients with latent B19V. However, despite the causative agent being of viral origin, PCR results may be negative because of viral clearance.

Although viruses are thought to be the most common cause of myocarditis, viral titers are not useful for diagnosis and treatment.

## 5. Treatment

### 5.1. Therapeutic flowcharts

Most cases of myocarditis have a favorable prognosis with spontaneous regression of clinical symptoms and preserved ventricular function not requiring therapeutic intervention. The therapeutic flowchart for myocarditis in most patients is guided by diagnostic suspicion, since only a minority of patients will undergo EMB ( Figure 4 ).^65^

Patients with low diagnostic suspicion of myocarditis presenting with no signs of severity, preserved ventricular function, and no ventricular arrhythmias have a favorable prognosis and should be clinically monitored with no need for drug therapy. In patients with intermediate diagnostic suspicion of myocarditis presenting with preserved ventricular function or ventricular dysfunction with progressive improvement, cardioprotective therapy with beta-blockers, angiotensin-converting enzyme inhibitors (ACEIs), or angiotensin receptor blockers (ARBs) is used to preserve or improve ventricular function.^12 , 55^

Patients with high diagnostic suspicion presenting with any of the indicators of poor prognosis, such as clinical deterioration, hemodynamic instability, maintained or progressive ventricular dysfunction, frequent ventricular arrhythmias, and significant conduction disorders, should undergo EMB for investigation of inflammation and etiologic agent. This will allow establishing a specific therapy with immunosuppression,^161 , 162^immunomodulation,^163 - 166^and antiviral drugs,^167 - 170^which may be beneficial in terms of clinical improvement, functional class, ventricular function, and survival ( Figure 5 ).^8 , 162 , 171 - 175^

**Figure 5:** – Therapeutic flowchart for myocarditis based on endomyocardial biopsy results.

PCR: polymerase chain reaction; DCM: dilated cardiomyopathy

### 5.2. Immunosuppression: indications and types

Immunosuppressive therapy in myocarditis is used to suppress the inflammatory response and autoimmune activity with the purpose of improving clinical status and ventricular function and thus reducing mortality.

In lymphocytic myocarditis, despite the pathophysiological rationale for using immunosuppression based on the presence of myocardial inflammation on EMB combined with absence of viral genome, the evidence supporting this hypothesis is limited. Factors such as spontaneous regression of inflammation, lack of uniform diagnostic criteria in the studies, reduced number of patients in most trials, heterogeneity of the clinical characteristics of study populations, and paucity of studies evaluating mortality reduction alone hamper an analysis of the clinical benefits of immunosuppressive therapy in lymphocytic myocarditis ( Table 14 ).^55 , 161 , 162 , 172 , 176 - 179^

**Table 14:** - Analysis of the clinical benefits of immunosuppressive therapy in lymphocytic myocarditis

Author	Design	Intervention	Placebo	N	Disease	Duration of symptoms	Inclusion	Virus-positive EMB	LVEF	Outcomes
Parrillo et al., 1989	Randomized, controlled	Prednisone	No	102	DCM	Mean: 8 months	Idiopathic	Yes No	>35%	Neutral
Latham et al., 1989	Randomized, controlled	Prednisone	No	52	DCM	<2 years, mean: 1.6 to 1.8 months	Idiopathic	Yes No	<40%	Neutral
Wojnicz et al., 2001	Randomized, controlled, open-label	Prednisone + azathioprine	Yes	84	DCM	>6 months	HLA	Yes No	<40%	Improved EF
Wojnicz et al., 2006	Randomized, controlled, open-label, 2-center	Atorvastatin	No	74	DCM	>6 months	HLA	Yes No	<40%	Improved EF/NYHA functional class
Frustaci et al., 2009	Randomized, controlled, double-blind, multicenter	Prednisone + azathioprine	Yes	85	DCM	>6 months	CD3 >7, CD45 >14 virus-negative	Yes Yes	<45%	Improved EF
Merken et al., 2018	Case series	Prednisone + azathioprine	No	180	DCM	Mean: 8 to 11 months	CD3 >7, CD45 >14 Virus-negative	Yes Yes	<45%	Improved transplant-free survival/EF

*DCM: dilated cardiomyopathy; EMB: endomyocardial biopsy; HLA: histocompatibility antigen; LVEF: left ventricular ejection fraction; NYHA: New York Heart Association.*

In the Myocarditis Treatment Trial (MTT),^178^which included patients with myocarditis meeting the Dallas criteria combined with the presence of ventricular dysfunction, immunosuppression for 6 months was not superior to conventional treatment in improving ventricular function and survival, although infectious agents were not investigated. The Tailored Immunosuppression in Inflammatory Cardiomyopathy (TIMIC) study, an Italian double-blind, randomized, placebo-controlled trial,^162^demonstrated improved ventricular function with immunosuppression in patients with myocarditis on biopsy (more than 7 lymphocytes per mm^2^), HF for more than 6 months, and absence of viral genome on EMB. Thus, although the evaluated phase was different from the acute phase of myocarditis, the study demonstrated the benefit of immunosuppression in the absence of viral genome in the myocardium. However, the nonidentification of specific viruses only means that the investigated viruses are not present and does not rule out the possible presence of other microbes.^162^Also, qualitative findings of microbes on EMB do not establish an undoubted causal relationship with the development of myocarditis/myocardiopathy, since viral genomes can be found in cardiomyopathies of other specific etiologies and even in normal hearts.^45 , 180 , 181^Taking B19V as an example, as its presence in myocardial tissue on qualitative PCR assay is common, other techniques documenting a low amount of copies^167^or absence of RNA transcription^182^could infer a noncorrelation with the development of myocarditis/myocardiopathy, allowing immunosuppression to be considered, even when the genome of this virus is present.

Immunosuppression is well established in myocarditis due to autoimmune disorders, and different strategies should be considered for each entity. Most strategies consist of corticosteroids usually combined with additional immunosuppressive drugs ( Table 15 ).^183 - 188^

**Table 15:** - Indications for immunosuppression in autoimmune myocarditis

Giant cell myocarditis	Rare but fulminant, improved prognosis with combined immunosuppression	Combined corticosteroid (cyclosporine + azathioprine)
**Sarcoidosis**	Systemic disease primarily affecting the lungs. Myocarditis in 10%, blocks, tachyarrhythmias, and ventricular dysfunction	Combined corticosteroids (cyclophosphamide, methotrexate), biologics in refractory cases
**Systemic lupus erythematosus**	Myocarditis in 50%, might be subclinical; rare with current immunosuppression; might accelerate atherosclerosis	Corticosteroid pulse therapy (followed by oral therapy for weaning), combined therapy (cyclophosphamide), plasmapheresis, IVIg
**Systemic sclerosis**	Myocarditis or secondary to pulmonary hypertension. Arrhythmias, conduction disorders, and ventricular dysfunction	Combined corticosteroids (cyclophosphamide, azathioprine)
**Behçet disease**	Myocarditis is rare (0.5%), poor prognosis	Combined corticosteroids (colchicine, anticoagulation)
**EGPA (Churg-Strauss syndrome)**	Myocarditis in up to 50%; history of asthma, presence of eosinophilia; chest pain, palpitations, and cardiogenic shock	Combined corticosteroids (cyclophosphamide)
**Rheumatoid arthritis**	Myocarditis in 30%, might be subclinical; rare with current immunosuppression; might accelerate atherosclerosis	Combined corticosteroids (methotrexate, biologics)

Despite the low incidence, the diagnosis of giant cell myocarditis cannot be delayed because of the severity of clinical manifestations, and the treatment consists of intensive combined immunosuppression. A classic study conducted by Cooper et al.^8^showed that survival increased from 3 to 12 months with combined immunosuppression (corticosteroid and/or azathioprine and/or cyclosporine and/or antilymphocyte antibody) compared to no immunosuppression or only corticosteroids.^8^A more recent study demonstrated a 5-year survival of 58% with the combined use of corticosteroids, cyclosporine, and azathioprine.^189^In refractory cases, antilymphocyte antibodies,^190^mycophenolate mofetil,^191^and sirolimus^192^have been described.

Eosinophilic myocarditis may be idiopathic or secondary to drug hypersensitivity reactions, autoimmune diseases (eosinophilic granulomatosis with polyangiitis or Churg-Strauss syndrome), hypereosinophilic syndrome, infections, and cancer. Immunosuppression is also considered in this setting, usually with corticosteroids. A recent literature review demonstrated peripheral eosinophilia in 75% of patients, immunosuppression in 80%, and combined therapy in 20% (especially Churg-Strauss and hypereosinophilic syndromes), with high 30-day mortality (13% hypereosinophilic syndrome, 17% idiopathic, 23% Churg-Strauss syndrome, and 40% hypersensitivity).^193^

The most commonly used immunosuppressive therapy in patients with confirmed myocarditis consists of corticosteroids alone or in combination with azathioprine ( Table 16 ), with the diagnosis by EMB of inflammation with no viral infection as determinants for immunosuppression ( Table 17 ). Patients undergoing immunosuppressive therapy should be continuously monitored for the development of side effects, as these may significantly increase both morbidity and mortality.^55^

**Table 16:** - Immunosuppressive therapy with corticosteroids

**Giant cell myocarditis** Corticosteroid pulse therapy – methylprednisolone 500 to 1,000 mg for 3 to 5 days; prednisone – 1 mg/kg and then slow and gradual withdrawal Antilymphocyte antibody – Thymoglobulin – 1.5 mg/kg/day, according to the evolution of CD3 T lymphocytes – cyclosporine – 3 to 8 mg/kg Azathioprine – 2 mg/kg
**Lymphocytic and eosinophilic myocarditis** Up to week 4 – 1 mg/kg Weeks 5 to 12 – reduce dosage by 0.08 mg/kg/week Weeks 13 to 20 – maintain dosage at 0.3 mcg/kg/day Weeks 21 to 24 – reduce dosage by 0.08 mg/kg/week TIMIC study: prednisone – 1 mg/kg for 4 weeks and 0.33 mg/kg for 5 months; azathioprine – 2 g/kg for 6 months
**Sarcoidosis** Prednisone – 30 mg/day – remove 5 mg per month for 12 to 24 months Combination when corticosteroid withdrawal is difficult: methotrexate – 10 to 20 mg/week Azathioprine – 2 mg/kg; hydroxychloroquine – 200 to 400 mg/day Leflunomide – 10 to 20 mg/day

**Table 17:** - Indications for immunosuppressive therapy in myocarditis

Indications	Class	Level of evidence
In the presence of positive myocarditis – giant cell, autoimmune disorders, sarcoidosis, and eosinophilia – associated with ventricular dysfunction	I	B
In the presence of endomyocardial biopsy-proven positive myocarditis and negative viral screening in patients with chronic heart failure, with the purpose of improving clinical status and ventricular function	IIa	B
In acute heart failure unresponsive to standard therapy	III	C

### 5.3. Antiviral therapy: indications and types

The prognosis of inflammatory cardiomyopathy/myocarditis is negatively affected by viral persistence. In viral cardiomyopathy, certain viruses are closely associated with a spontaneous course of viral infection, since spontaneous viral elimination is accompanied by clinical improvement, but this does not apply to patients who develop viral persistence.^194 - 197^

Patients with enteroviral and adenoviral genomes on EMB should be treated with interferon-beta (IFN-ß) (4 million units subcutaneously every 48 hours in the first week, 8 million units subcutaneously every 48 hours from the second week on, for 6 months). A nonrandomized study demonstrated that administration of IFN-ß to patients positive for enteroviral and adenoviral infection induced viral elimination, reduced myocardial injury, and significantly improved long-term survival.^198 , 199^In a subsequent phase 2 study – Betaferon in Chronic Viral Cardiomyopathy (BICC) trial –, 143 patients with symptoms of HF and biopsy-proven enterovirus, adenovirus, and/or B19V genomes were randomly assigned to double-blind treatment and received either placebo or IFN-ß for 24 weeks, in addition to standard HF treatment. Compared to placebo, viral elimination and/or viral load reduction were higher in the IFN-ß groups. IFN-ß treatment was associated with favorable effects on NYHA functional class, quality of life, and patient global assessment. In retrospective analyses, IFN-ß treatment was found to be significantly less effective in eliminating B19V infection.^171^

A high prevalence of HHV-6 has been detected in the myocardial tissue of patients presenting with symptoms of HF in a clinically suspected setting of myocarditis. Interestingly, HHV-6 is able to integrate its genome into telomeres of human chromosomes, which allows transmission of HHV-6 via the germline. Chromosomally integrated HHV-6 (ciHHV-6) appears to be associated with an increased risk of myocarditis and may lead to severe HF. HHV-6 is also not eliminated by IFN-ß, but symptoms of HHV-6 reactivation and HF have decreased after a 6-month treatment period with ganciclovir followed by valganciclovir (ganciclovir 1,000 mg/24h intravenously for 5 days, then valganciclovir 900 mg/24h or 1,800 mg/24h for 6 months) in symptomatic patients with reactivated ciHHV6 (positive messenger RNA).^200^

B19V infection of the heart muscle is still a matter of debate. Initial data have provided evidence that antiviral nucleoside analogue reverse transcriptase inhibitors such as telbivudine can improve the clinical outcomes of patients with positive B19V DNA and replicative intermediates.^201^However, a large randomized, placebo-controlled clinical trial is now required to evaluate the results.

### 5.4. Immunomodulation (immunoglobulin and immunoadsorption): indications and types of immunoglobulins

The rationale for the use of intravenous immunoglobulin (IVIg) in the treatment of myocarditis is based on their ability to interact widely with the immune system. They are able to stimulate the complement system and immune cells to release anti-inflammatory cytokines and inhibit the release of proinflammatory cytokines.^83^

Immunoglobulins have been assessed in different settings such as chronic HF,^202 , 203^dilated cardiomyopathy,^166 , 204^peripartum cardiomyopathy,^205^acute myocarditis,^164 , 165 , 206 , 207^fulminant myocarditis,^208^and viral myocarditis.^167 , 169^

Although some of these studies suggest a potential benefit of immunoglobulin, a randomized controlled trial evaluating adult patients with new-onset dilated cardiomyopathy (<6 months) or myocarditis did not demonstrate evidence of beneficial effect of immunoglobulin on ventricular function in the treatment group versus the control group. There was improvement in ventricular function and even normalization in 36% of cases during follow-up, regardless of the study group. It is worth noting that the biopsy did not include any viral screening, and only 16% of patients had myocarditis confirmed by the presence of inflammation on biopsy.^166^

In patients with acute myocarditis, early studies have suggested an improvement in ventricular function and a tendency to higher 1-year survival with high-dose IVIg.^164^However, a 2005 systematic review found 17 studies including only one randomized controlled trial (62 patients), and did not demonstrate any benefit of IVIg in patients with acute myocarditis. The authors concluded that there is insufficient evidence to recommend routine use of IVIg in this setting.^207^More recently, a small randomized, multicenter study (41 patients) evaluated the short-term prognosis of patients with acute myocarditis or new-onset cardiomyopathy undergoing IVIg therapy compared to patients who did not receive IVIg. The study revealed improved short-term survival among patients who received IVIg and no significant difference in ventricular function between groups. However, there was a significant reduction in inflammatory cytokines in the treated group. The study hypothesizes a potential benefit of immunoglobulins and suggests their mechanism of action; however, because of the small number of patients, the evidence is insufficient to recommend unrestricted use of IVIg in patients with acute myocarditis.^209^

In viral myocarditis, nonetheless, there are data demonstrating the benefits of immunoglobulin. In a pilot study evaluating patients with B19V myocarditis, IVIg significantly reduced viral load and improved cardiac function.^167^In an analysis of 152 patients with adenovirus- or B19V-positive myocarditis, immunoglobulin improved exercise capacity, LV ejection fraction, and NYHA functional class. There was a significant reduction in inflammation in both groups of patients and a significant reduction in viral load only among patients with adenovirus-positive myocarditis; approximately 40% of patients with B19V infection had viral persistence.^169^These findings suggest a potential benefit of immunoglobulin in patients with EMB-proven viral myocarditis.

Current data, although insufficient for routine recommendation of IVIg, are indicative of a potential benefit of immunoglobulin in patients with biopsy-proven myocardial inflammation, especially viral myocarditis caused by adenoviruses and B19V.

#### 5.4.1. Immunoadsorption

The pathogenesis of progression to ventricular dysfunction in dilated cardiomyopathy involves inflammatory processes that can be identified and quantified by immunohistochemical methods, which suggests a causal relationship between myocarditis and cardiomyopathy.^210^The presence of lymphocytes, mononuclear cells, and increased gene expression of HLA antigens is frequent, as well as that of antibodies against mitochondrial and contractile proteins; B1 receptors and muscarinic receptors have also been described in dilated cardiomyopathy.^211 - 214^

These cardiac antibodies are extractable by immunoadsorption, and some studies have tested the efficacy of this method in the treatment of patients with dilated cardiomyopathy/myocarditis.^215 , 216^In a small controlled study, 25 patients were randomized to either undergo immunoadsorption followed by IgG substitution or continue conventional therapy, and a significant reduction in myocardial inflammation (decreases in CD3 cells, CD4 and CD8 lymphocytes, and HLA class II antigen expression) was found in the treated group.^217^In other small randomized studies, improvement in hemodynamics and ventricular function was observed.^216^

Current data suggest that immunoadsorption may be a new and promising therapeutic approach for patients with dilated cardiomyopathy and the presence of cardiac antibodies. However, to date, evidence is based on small uncontrolled studies or open-label controlled studies compared to conventional therapy, and their results require confirmation by large randomized, prospective, multicenter studies.^218^An ongoing double-blind, placebo-controlled, multicenter study will evaluate the effects of immunoadsorption followed by IgG substitution in patients with dilated cardiomyopathy.^219^Only with the results of this large study will we be able to establish a grade of recommendation for this therapy in the setting of dilated cardiomyopathy/myocarditis.

## 5.5. Conventional cardioprotective therapy

### 5.5.1. No ventricular dysfunction

The therapeutic approach for patients with myocarditis with preserved ventricular function aims to prevent the development of ventricular dysfunction or malignant arrhythmias. In patients with suspected diagnosis and intermediate risk, beta-blockers and ACEIs or ARBs can be used for at least 12 months to reduce mortality and morbidity. The decision to extend therapy will be based on the assessment of ventricular function and arrhythmogenic potential. As no clinical trials have addressed patients with this myocarditis profile, management should follow the SBC chronic and acute heart failure guidelines.

### 5.5.2. Ventricular dysfunction and hemodynamic stability

Therapeutic management of ventricular dysfunction in myocarditis should be in line with current HF guidelines.^55 , 220 , 221^Medications recommended for all hemodynamically stable patients with symptomatic ventricular dysfunction, such as cardioprotective therapy, unless contraindicated, are known as triple therapy – ACEIs or ARBs, beta-blockers, and mineralocorticoid receptor antagonists. ACEIs/ARBs and beta-blockers can be initiated in all individuals with HF with reduced ejection fraction, even if they are asymptomatic, unless contraindicated, and should be maintained when ventricular function normalizes. Spironolactone, a mineralocorticoid receptor antagonist marketed in Brazil, should be initiated when the patient is already taking other medications and maintaining symptoms (NYHA II-IV functional class), and should be avoided in patients with creatinine >2.5 mg/dL or persistent hyperkalemia ( Table 18 ).

**Table 18:** - Recommendations for general pharmacological measures in myocarditis

Indications	Class	Level of evidence
Treatment with prognostic-modifying drugs for symptomatic or asymptomatic patients with left ventricular systolic dysfunction, according to current heart failure guidelines	I	C
Maintenance of neurohormonal blockade therapy after normalization of ventricular function	I	C
Consider neurohormonal blockade drugs in patients with evidence of myocardial fibrosis without dysfunction	IIa	C

### 5.5.3. Hemodynamically unstable patients with ventricular dysfunction: therapeutic approach

Patients with acute myocarditis and systolic ventricular dysfunction may fit into different clinical models. Likewise, clinical response to therapy is quite variable, and there may or may not be a clear manifestation of low cardiac output or evidence of systemic hypervolemia. The use of inotropes is warranted in at least three situations: a clear low-output state, cardiorenal syndrome refractory to optimization of diuretic therapy, and mixed venous oxygen saturation (SvO_2_) below 60% with invasive hemodynamic criteria for low output. According to the line of care, invasive monitoring for patients with no clear response to this therapy should be considered ( Table 19 ).^222 - 225^

**Table 19:** - Inotropes used in hemodynamically unstable patients with myocarditis and ventricular dysfunction222-225

	Dobutamine	Milrinone	Levosimendan
**Clinical practice in myocarditis**	B1 selective agonist, which promotes inotropism by direct stimulation of beta-receptors.	Experimental murine models suggest protective effects of milrinone and levosimendan over dobutamine on vasodilation in myocarditis. It acts as a phosphodiesterase inhibitor at any dose, thus increasing calcium concentration in cardiomyocytes. Systemic vasodilation contributes to increased cardiac output.	There are experimental murine models demonstrating a reduction in cell apoptosis and inflammatory cytokines with levosimendan in acute myocarditis. However, there is no robust evidence to recommend it as a cardioprotective agent in patients with myocarditis or to prove its clinical benefit over other inotropes. It acts as a calcium sensitizer up to 0.2 mcg/kg/min; at higher doses, it works as a phosphodiesterase inhibitor, with no clinical use tested. There is no clinical evidence of continuous use for more than 48 hours.
**Inotropism**	Moderate	Important	Important
**Vasodilation**	Mild	Moderate to important	Moderate to important
**Increased cardiac output**	Low to moderate	Important, associated with vasodilation	Important
**Risk of hypotension**	Low	Important and dose-dependent, higher in patients with established renal dysfunction	Important, especially if bolus is used. Increases with increasing dose.
**Risk of arrhythmias**	Increases exponentially when higher than 10 mcg/kg/min	Increases in case of bolus dose (not recommended)	Increases in case of initial bolus, also dose-dependent, more commonly at 0.2 mcg/kg/min

## 5.6. General recommendations: physical activity and vaccination

Myocarditis is an important cause of sudden death in athletes, which may occur in both the acute and chronic phases. This is related not only to the degree of myocardial inflammation but also to the triggering of complex arrhythmias and the development of left ventricular dysfunction.^226 - 228^

Competitive or recreational athletes with active myocarditis should not participate in competitive sports or high-intensity exercise until the end of convalescence. There is no consensus on how long this period is. Until recently, the recommendation was to wait at least 6 months after the onset of clinical manifestations. Currently, some experts recommend shorter periods, such as 3 months, for resuming exercise training and competitive sports depending on the presence of symptoms, arrhythmias, ventricular dysfunction, inflammatory markers, and ECG changes^12 , 229^( Table 20 ).

**Table 20:** – Exercise recommendations for athletes and nonathletes with myocarditis12,229

Indications	Class	Level of evidence
Athletes may return to training and competitive sports, and nonathletes, to their usual physical activities, 3 to 6 months after myocarditis only if all of the following criteria are met: – LV systolic function within the normal range – Normal myocardial injury biomarkers – Absence of arrhythmias on 24-hour Holter and stress testing	IIa	C
With previous myocarditis, individuals should be reassessed periodically, especially during the first 2 years, owing to an increased risk of recurrence and silent progression of the disease	IIa	C
Return to competitive sports and physical activities in asymptomatic athletes and nonathletes with persistent late gadolinium enhancement on CMR is considered in the period of 3 to 6 months after myocarditis if normal LV function and absence of arrhythmias on 24-hour Holter and stress testing, and they should be followed-up periodically for the potential risk of tachyarrhythmias. In the presence of positive late gadolinium enhancement on CMR, they should be assessed annually	IIa	C

*CMR: cardiac magnetic resonance; LV: left ventricular.*

The European Consensus Document for Cardiovascular Prevention and Rehabilitation recommends that, in patients with HF, including those with myocarditis, exercise training should be of moderate intensity (up to 50% of VO_2_peak or 60% of predicted maximal heart rate), provided there is no laboratory evidence of inflammation or arrhythmias.^230^

During the Covid-19 pandemic, professional athletes have needed to interrupt or postpone their activities because of the risk of contamination. With the relaxation of social distancing measures, the question now is how athletes can safely return to their activities. Athletes who have had Covid-19 may present with respiratory symptoms, muscle fatigue, and risk of thrombotic events. Because of such risks, a flowchart with recommendations for clinical assessment and return-to-play decisions has been developed to provide guidance for resuming physical activities ( Figure 6 ).^231^

**Figure 6:** – Flowchart for returning to exercise following Covid-19.

*History and physical examination in the investigation of post-Covid-19 complications (neurological, gastrointestinal, and dermatological).

CMR: cardiac magnetic resonance; CPET: cardiopulmonary exercise test; CRP: C-reactive protein; CT: computed tomography; ECG: electrocardiogram; ECHO: echocardiogram; hs-cTn: high-sensitivity cardiac troponin; PFP: pulmonary function test.

Vaccination follows the same recommendations as those for annual influenza and pneumococcal immunization of patients with HF in addition to other available vaccines (mumps, measles, rubella, poliomyelitis). There is no robust evidence that these predispose patients to exacerbation or development of acute myocarditis to outweigh the benefits of immunization.^231 - 235^The same rationale applies to Covid-19 vaccination. To be vaccinated, patients should not be in the acute phase of myocarditis, and the recommendation is to wait 3 months of the diagnosis ( Table 21 ).

**Table 21:** – Vaccination recommendations in myocarditis

	Class	Level of evidence
Vaccines for influenza, pneumococcus, mumps, measles, rubella, poliomyelitis, and Covid-19. Patients should not be in the acute phase of the disease, and the recommendation is to wait 3 months of first diagnostic suspicion.	I	C

## 6. Special situations

### 6.1. Fulminant myocarditis

Fulminant myocarditis is currently defined by a pragmatic approach with predominantly clinical features, irrespective of histological findings, as follows: 1) clinical presentation of severe HF symptoms for less than 30 days; 2) hemodynamic instability with cardiogenic shock and life-threatening arrhythmias (including recovered or aborted cardiac arrest); and 3) need for hemodynamic support (inotropes or mechanical circulatory assist device).^236^In addition to the previously mentioned tests for evaluating patients with myocarditis, EMB is recommended in fulminant myocarditis. Results are usually positive and show multiple inflammatory foci, allowing histological characterization of the type of myocarditis.^237^The clinical course of fulminant myocarditis tends to be more dismal than those of nonfulminant types of myocarditis, with a lower likelihood of ventricular function recovery, higher mortality, and a higher chance of heart transplantation.^236 , 238^

#### 6.1.1. Diagnostic evaluation

The diagnosis of fulminant myocarditis involves the diagnostic criteria of myocarditis per se, including clinical features of acute HF, elevated troponins and inflammatory markers, nonspecific ECG changes such as T-wave inversions and/or ST-segment abnormalities, and acute ventricular function changes. In the setting of cardiogenic shock, right heart catheterization and coronary angiography are essential for guiding management. Echocardiography is a key diagnostic tool, since patients with fulminant myocarditis are frequently unable to undergo MRI. Echocardiographic findings are highly dependent on the form and timing of presentation. Patients with fulminant myocarditis typically have normal diastolic dimensions but increased septal thickness at presentation, whereas patients with acute (nonfulminant) viral myocarditis may present with either normal or increased diastolic dimensions but normal septal thickness consistent with other forms of dilated cardiomyopathy.^15 , 64 , 72 , 98 , 239 , 240^

The decision to perform EMB at the time of cardiac catheterization is in line with those of the ESC 2013^15^task force. EMB can be considered the initial diagnostic procedure when MRI is not possible (eg, shock, presence of metal devices), if experienced operators and cardiac pathologists are available. According to the guidelines, therefore, the indications for EMB would be present for most patients with fulminant myocarditis ( Figure 4 ) . Higher accuracy can be achieved by adding viral genome analysis, immunohistology, or transcriptomic biomarkers if there is diagnostic uncertainty despite histology.

In addition to confirming diagnosis, EMB in fulminant myocarditis can be decisive for defining therapy. Immunohistochemical analysis has been considered mandatory because of known diagnostic limitations of the Dallas criteria, especially interobserver variability, which possibly limits diagnostic confirmation to no more than 20% of cases.^15 , 64 , 72 , 239 , 240^According to the WHO definition for diagnosis of active myocarditis, immunohistochemical detection of mononuclear infiltrates (T lymphocytes or macrophages) using a cutoff point of 14 cells/mm^2^or over is required in addition to increased expression of HLA class II molecules.^146^

Viral genome detection in biopsy specimens is feasible (limited availability in Brazil) and, when coupled with immunohistochemical analysis, increases diagnostic accuracy and provides etiologic and prognostic information.

For fulminant myocarditis, a class I, level C indication has been considered even when only histological analysis is present (Dallas criteria). Conventional histological analysis is widely available and enables etiologic diagnosis that may lead to changes in therapeutic approach and specific treatment for presentations such as necrotizing eosinophilic myocarditis, giant cell myocarditis, sarcoidosis, amyloidosis, and myocarditis associated with known autoimmune diseases.

#### 6.1.2. Therapeutic approach

The recognition of the causal factor through histological investigation by EMB allows the establishment of specific therapeutic strategies, such as immunoglobulin in viral myocarditis, immunosuppression in nonviral, autoimmune myocarditis, or corticosteroids in sarcoidosis, necrotizing eosinophilic myocarditis, or giant cell myocarditis. A randomized clinical trial of immunosuppression in 85 patients with myocarditis with proven absence of viral persistence (TIMIC study) demonstrated a clear beneficial effect on ejection fraction. However, these patients had more than 6 months of diagnosis and proven absence of virus.^162^Clinical trials of immunosuppression in patients with fulminant myocarditis do not exist. One option that has been tested is the use of high-dose of immunoglobulin, which has been shown to be beneficial over ventricular function and functional class, and has shown survival benefit;^208 , 209 , 217^although it has been demonstrated in a clinical trial with 62 patients, in which only 16% had biopsy-proven myocarditis the absence of benefit.^166^

Supportive treatment should include vasoactive drugs and possibly vasopressors, and should be used in situations allowing the introduction of vasodilators. Immediate failure of drug treatment and volume replacement should lead clinicians to consider indication for hemodynamic support with mechanical circulatory assist devices. The most common devices are intra-aortic balloon pump, percutaneous devices such as TandemHeart and Impella, extracorporeal membrane oxygenation (ECMO), and paracorporeal ventricular assist devices, which are all used as a bridge to recovery or to heart transplantation ( Figure 7 ). Short-term support devices are indicated for 7 to 10 days.^241^After that and while stabilization is not achieved, ECMO or artificial ventricles are indicated to provide support for a longer period, allowing patients a greater chance of recovery from ventricular dysfunction^242^(see section on *Cardiogenic shock* ).

**Figure 7:** - Approach for initial stabilization of patients with cardiogenic shock.

ACS: acute coronary syndrome; CABG: coronary artery bypass grafting; ECMO: extracorporeal membrane oxygenation; IABP: intra-aortic balloon pump; PCI: percutaneous coronary intervention; VAD: ventricular assist device. Adapted from Kociol et al.^63^

## 6.2. Sarcoidosis

### 6.2.1. Diagnosis

Sarcoidosis is a granulomatous inflammatory disease of unknown etiology characterized by noncaseating granulomas. It may involve multiple organs, especially lungs (90%), skin, lymph nodes, central nervous system, eyes, liver, heart, and others.^243^Although clinically manifest cardiac sarcoidosis only affects 5% to 10% of patients with sarcoidosis, autopsy studies have revealed that cardiac involvement is present in 20% to 30% of patients on advanced cardiac imaging. With CMR or PET imaging, cardiac involvement has increased to 40%.^244 - 246^In addition to different definitions for the same entity, another factor that seems to have an impact on the increased prevalence of sarcoidosis is the refinement of imaging methods.

Currently, it is recommended the use of the guidelines of the Japanese Circulation Society (SJC) launched in 2019 ( Table 22 , Figures 8 and 9 ). Among the changes suggested in this document, we have that the abnormally high tracer accumulation in the heart with positron emission tomography by^18^F-FDG-PET/computed tomography, which was categorized in the “Guidelines for the diagnosis of cardiac involvement in patients with sarcoidosis”, in 2006, was promoted to the higher criteria, as well as the late enhancement by gadolinium of the myocardium on CMR with gadolinium. In the current JCS guidelines, a clinical diagnosis of cardiac sarcoidosis is also made when the patient shows clinical findings strongly suggestive of cardiac involvement and pulmonary or ophthalmic sarcoidosis as well as at least two of the five characteristic laboratory findings of sarcoidosis. Finally, the definition of isolated cardiac sarcoidosis was proposed for the first time.

**Table 22:** - Japanese Circulation Society recommendations for the diagnosis of cardiac sarcoidosis247

Critérios para envolvimento cardíaco
Cardiac findings should be assessed on the basis of major and minor criteria. Clinical findings satisfying the following 1) or 2) strongly suggest the presence of cardiac involvement.
1. Two or more of the five major criteria (a) to (e) are met.
2. One of the five major criteria (a) to (e) plus two or more of the three minor criteria (f) to (h) are met.
**Major criteria**
a. High-grade atrioventricular block (including complete atrioventricular block) or fatal ventricular arrhythmia (eg, sustained ventricular tachycardia and ventricular fibrillation)
b. Basal thinning of the ventricular septum or abnormal ventricular wall anatomy (ventricular aneurysm, thinning of the middle or upper ventricular septum, regional ventricular wall thickening)
c. Left ventricular contractile dysfunction (left ventricular ejection fraction less than 50%) or focal ventricular wall asynergy
d.^67^Ga citrate scintigraphy or^18^F-FDG PET reveals abnormally high tracer accumulation in the heart
e. Gadolinium-enhanced MRI reveals delayed contrast enhancement of the myocardium
**Minor criteria**
f. Abnormal ECG findings: ventricular arrhythmias (nonsustained ventricular tachycardia, multifocal or frequent premature ventricular contractions), axis deviation, or abnormal Q waves
g. Perfusion defects on myocardial perfusion scintigraphy
h. Endomyocardial biopsy: monocyte infiltration and moderate or severe myocardial interstitial fibrosis Diagnostic guidelines for cardiac sarcoidosis
**Diagnostic guidelines for cardiac sarcoidosis**
1. Histological diagnosis group (those with positive myocardial biopsy findings): cardiac sarcoidosis is diagnosed histologically when endomyocardial biopsy or surgical specimens demonstrate noncaseating epithelioid granulomas.
2. Clinical diagnosis group (those with negative myocardial biopsy findings or those not undergoing myocardial biopsy): the patient is clinically diagnosed as having cardiac sarcoidosis (1) when epithelioid granulomas are found in organs other than the heart, and clinical findings strongly suggestive of the previously mentioned cardiac involvement are present; or (2) when the patient shows clinical findings strongly suggestive of pulmonary or ophthalmic sarcoidosis; at least two of the five characteristic laboratory findings of sarcoidosis (bilateral hilar lymphadenopathy, high serum angiotensin-converting enzyme activity or elevated serum lysozyme levels, high serum soluble interleukin-2 receptor levels, significant tracer accumulation on 67Ga citrate scintigraphy or^18^F-FDG PET, a high percentage of lymphocytes with a CD4/CD8 ratio of >3.5 in bronchoalveolar lavage fluid). Clinical findings strongly suggest the previously mentioned cardiac involvement.
**Diagnostic guidelines for isolated cardiac sarcoidosis**
Prerequisites
1. No clinical characteristics of sarcoidosis are observed in any organs other than the heart (the patient should be examined in detail for respiratory, ophthalmic, and skin involvements of sarcoidosis. When the patient is symptomatic, other etiologies that can affect the corresponding organs must be ruled out.
2.^67^Ga scintigraphy or^18^F-FDG PET reveals no abnormal tracer accumulation in any organs other than the heart.
3. Chest CT reveals no shadow along the lymphatic tracts in the lungs or no hilar and mediastinal lymphadenopathy (minor axis >10 mm).
**Histological diagnosis group**
1. Isolated cardiac sarcoidosis is diagnosed histologically when endomyocardial biopsy or surgical specimens demonstrate noncaseating epithelioid granulomas.
**Grupo de diagnóstico clínico**
1. Isolated cardiac sarcoidosis is diagnosed clinically when the criterion (d) and at least three other criteria of the major criteria (a) to (e) are satisfied. When the patient meets at least four criteria for cardiac involvement other than the criterion (d), or when the patient meets the criteria (b) and (d) plus one of the remaining criteria, the patient should be suspected to have isolated cardiac sarcoidosis.

*^18^F-FDG PET:^18^F-fluorodeoxyglucose positron emission tomography; CT: computed tomography; ECG: electrocardiogram. Adapted from Terasaki et al.^47^*

**Figure 8:** – Diagnostic flowchart for cardiac sarcoidosis after the diagnosis of extracardiac lesions.

ECG: electrocardiogram; PET: positron emission tomography; SPECT: single-photon emission computed tomography. Adapted from Terasaki et al.^247^

**Figure 9:** – Diagnostic flowchart for cardiac sarcoidosis in patients who present with cardiac manifestations and are strongly suspected to have cardiac sarcoidosis.

AVB: atrioventricular block; CT: computed tomography; LV: left ventricular; PET: positron emission tomography; SPECT: single-photon emission computed tomography;^18^F-FDG PET:^18^F-fluorodeoxyglucose positron emission tomography. Adapted from Terasaki et al.^247^

### 6.2.2. Treatment and prognosis

Immunosuppressive treatment of cardiac sarcoidosis is based on clinical experience and expert opinion given the lack of randomized trials. The goal of treatment is to reduce inflammatory activity and prevent fibrosis, and the approach should be guided by the magnitude of the inflammatory process and the degree of myocardial involvement.^248^

Immunosuppressive treatment is recommended in the following situations: left ventricular dysfunction, ventricular arrhythmias, hypermetabolic activity on^18^F-FDG PET, conduction disorders, late gadolinium enhancement on CMR, or right ventricular dysfunction in the absence of pulmonary hypertension.^248 - 250^

There are three lines of treatment for sarcoidosis – first line: corticosteroids; second line: methotrexate and azathioprine in cases of intolerance or chronic use of corticosteroids; and third line: anti-TNF antibodies (infliximab and andalimumab) in cases of failure of previous treatments.^251^

Corticosteroid is the drug of choice. In a systematic review of corticosteroid therapy for ventricular conduction disorders, 27 of 57 patients (47.4%) improved with treatment.^252^However, because responses are unpredictable, patients with conduction disorders and cardiac sarcoidosis should receive a pacemaker or implantable cardioverter-defibrillator.^119 , 253^

Older studies evaluating the effect of corticosteroids on ventricular function have suggested preservation of ventricular function in patients with normal function at diagnosis, improvement in ventricular ejection fraction in patients with mild-to-moderate dysfunction, and no improvement in patients with significant ventricular dysfunction.^119^However, a Finnish study suggested an improvement in left ventricular function with immunosuppressive treatment in patients with severely compromised ventricular function (LVEF <35%), but no changes were observed in those with normal or moderately decreased function at the start of treatment. Perhaps such differences are associated with early diagnosis and treatment.^254^

Studies on ventricular arrhythmia are more limited; however, the cause of arrhythmia appears to be secondary to scarring, and perhaps the corticosteroid effect on these patients is of little benefit.^255^Catheter ablation in ventricular tachycardia may be considered after implantable cardioverter-defibrillator insertion or antiarrhythmic drug failure.^256^

The suggested treatment algorithm ( Figure 10 ) consists of an initial prednisone regimen (30 mg/day to 40 mg/day) followed by a repeat PET scan within 4 to 6 months to assess disease activity and guide subsequent pharmacological treatment.

**Figura 10:** – Sarcoidosis treatment algorithm

ECG: electrocardiogram; ECHO: echocardiogram;^18^F-FDG PET:^18^F-fluorodeoxyglucose positron emission tomography. PET: positron emission tomography.

Yokoyama et al.^257^compared^18^F-FDG PET/CT scans before and after corticosteroid therapy in 18 patients with cardiac sarcoidosis and observed that maximum standardized uptake value decreased significantly from baseline. A recent study used^18^F-FDG PET/CT for the diagnosis and treatment of cardiac sarcoidosis with low corticosteroid doses and good disease control within 1 year of diagnosis.^258^

Immunosuppressive drugs other than corticosteroids are needed because of the long duration of treatment. They are indicated for patients who require a maintenance prednisone dose >10 mg/day and who cannot tolerate corticosteroid side effects.^248 , 250^

The following drugs are suggested: methotrexate,^257^azathioprine,^258^cyclophosphamide,^259^and tumor necrosis factor inhibitors.^260 , 261^The choice of drug will be determined by the type of extracardiac involvement; methotrexate, for example, should be avoided in patients with liver involvement, and studies of pulmonary, cutaneous, ocular, neurological, and multisystem sarcoidosis have suggested good efficacy for infliximab ( Table 23 [Table t59001] ).^262^

**Table 23:** - Recommendations for immunosuppressive therapy in sarcoidosis

Indication	Class	Level of evidence
Prednisone 30 to 40 mg/day for 4 to 6 months	IIa	B
**Additional immunosuppressants in case of corticosteroid therapy:**
Azathioprine 50 to 200 mg/day	IIb	C
Methotrexate 10 to 20 mg/week	IIb	C
Infliximab in pulmonary, cutaneous, ocular, neurological, and multisystem sarcoidosis	IIb	C
Leflunomide 10 to 20 mg/day	IIb	C

**Table 24 t59001:** - Indication for implantable cardioverter-defibrillator in sarcoidosis

Indications	Class	Level of evidence
Ventricular tachycardia/aborted cardiac death	I	C
LVEF <35% despite optimal treatment and period of immunosuppression and active inflammation	I	C
Unexplained syncope probably due to arrhythmia	IIa	C
Ejection fraction 35% to 49% and/or RVEF <40% despite optimal immunosuppressive treatment and MRI or PET evidence of extensive myocardial scarring	IIa	C
Ejection fraction 35% to 49% and/or RVEF <40% despite optimal immunosuppressive treatment	IIb	C

*LVEF: left ventricular ejection fraction; MRI: magnetic resonance imaging; PET: positron emission tomography; RVEF: right ventricular ejection fraction.*

### 6.2.3. Prognosis

Cardiac sarcoidosis has a worse prognosis compared to dilated cardiomyopathy. Once the heart is affected, the prognosis is unfavorable. Cardiac involvement accounts for 85% of deaths from the disease.^183 , 243^

Kandolin et al.^256^reported the long-term effects of immunosuppressive treatment in a Finnish cohort, and 1-year, 5-year, and 10-year transplant-free survival rates were 97%, 90%, and 83%, respectively, during the 6.6-year follow-up period. In that study, presence of HF and cardiac function before corticosteroid therapy were the most important factors for estimated prognosis, which shows that early treatment is key.

The presence of late gadolinium enhancement on CMR increased the risk of death, aborted sudden death, or cardioverter-defibrillator implantation by 30 times during a 2.6-year follow-up period,^262^and this finding was subsequently confirmed by meta-analyses. It has been suggested that the 20% fibrotic mass threshold is associated with risk of events.^263^

A study using PET imaging observed that 26% of reported adverse events, such as ventricular tachycardia and death, were cases of cardiac uptake on PET during a 1.5-year follow-up period. Conversely, extracardiac uptake was not associated with adverse events at follow-up.^264^

Another interesting finding is that patients with isolated cardiac sarcoidosis have a worse prognosis compared to patients with systemic sarcoidosis with cardiac involvement.^265^A Finnish study observed a high frequency of ventricular dysfunction and septal abnormalities on echocardiography and a high prevalence of late gadolinium enhancement on CMR, in addition to more significant associations with female sex and more severe left ventricular dysfunction.^266^In that study, HF at presentation, severe left ventricular dysfunction (<35%), and isolated cardiac sarcoidosis were also related to prognosis.^254^

Echocardiography with strain imaging (global longitudinal strain <17.3) was an independent predictor of mortality, HF, hospitalization, new arrhythmias, and development of cardiac sarcoidosis.^267^

Serum biomarkers such as BNP were related to the development of HF, whereas troponin was associated with the development of fatal arrhythmias,^268^lower ejection fraction, and poor prognosis.^269^

## 6.3. Giant cell myocarditis

### 6.3.1. Treatment

According to an international registry, giant cell myocarditis is the etiology of 12% cases of fulminant myocarditis and 3.6% of cases of nonfulminant myocarditis.^242^Treatment goals are limited because the mechanisms of giant cell myocarditis are not properly known, although an autoimmune mechanism involving myocardial inflammation mediated by T lymphocytes has been proposed.^270 , 271^

Giant cell myocarditis has a worse prognosis than eosinophilic and lymphocytic myocarditis, and is more frequently associated with HF, cardiac arrest, fibrillation and ventricular tachycardia, heart blocks, or simulated acute MI.^242 , 272^Without treatment, the course is usually fatal, with death within 5.5 months.^271^Even with treatment, giant cell myocarditis has a high mortality or requires early indication for mechanical circulatory support and/or heart transplantation.

Recently, a 5-year transplant-free survival of 42% has been reported. As important prognostic markers of early death or need for mechanical support or heart transplantation, troponin levels and moderate-to-severe necrosis or fibrosis on EMB have been described. Elevated BNP/NT-proBNP levels and significant LVEF reduction are additional prognostic markers.^191^Poor prognosis may be associated with myocardial injury or recurrent giant cell myocarditis.^273^After heart transplantation, recurrence has also been described.

Early diagnosis is critical and dependent on EMB results, or histological analysis of a heart explanted during heart transplantation, or myocardial specimens collected during ventricular assist device implantation.^270 , 274 , 275^Biopsy sensitivity might be limited by sampling error. Specimens are preferably collected from the apical portion of the RV septum because the risk of complications is lower. A negative biopsy does not necessarily exclude the diagnosis of giant cell myocarditis. EMB sensitivity has increased from 68% to 93% after repeating the procedure ( Table 25 ).

**Table 25:** - Endomyocardial biopsy (EMB) recommendations in the diagnostic evaluation of giant cell myocarditis

Indications	Class	Level of evidence
EMB or analysis of a heart explanted during heart transplantation, or myocardial specimens collected during mechanical assist device implantation in patients with acute heart failure with severe or fulminant hemodynamic compromise	I	B
Suspected diagnosis of myocarditis associated with cardiac arrest, or ventricular fibrillation or tachycardia, or blocks, or simulated acute myocardial infarction	I	B

The treatment of giant cell myocarditis is divided into the treatment of HF with reduced LVEF caused by myocardial injury or recurrent giant cell myocarditis, arrhythmias, and blocks, and the treatment of the probable mechanism with immunosuppressants.

The treatment of HF, hemodynamic disorders, blocks, and arrhythmias is consistent with the treatment of HF according to the SBC guidelines, including drugs and/or inotropes, pacemakers/defibrillators and/or mechanical circulatory support, and heart transplantation.^242^Heart transplantation might be indicated earlier because of the poor prognosis of giant cell myocarditis, even with immunosuppressants. Implantable cardioverter-defibrillator might be indicated for primary prevention of sudden or secondary death based on the high incidence of complex and severe arrhythmias.^276^It has been described that 59% of patients with giant cell myocarditis had sustained ventricular tachycardia or shocks for complex ventricular arrhythmias despite of being free from severe HF.

The indication for immunosuppressants is based on the results of case series or small randomized studies. Immunosuppressive drugs such as prednisone, cyclosporine, azathioprine, mycophenolate mofetil, everolimus, sirolimus or rabbit antithymocyte globulin, antithymocyte globulin or muromonab-CD3 have been used for cytolysis of T lymphocytes. After initial diagnosis, high-dose corticosteroids and/or rabbit antithymocyte globulin, antithymocyte globulin or muromonab-CD3 are generally used, and combination with long-term immunosuppressants is possible. Hemadsorption has also been reported ( Table 26 ).^277^

**Table 26:** - Therapeutic recommendations in giant cell myocarditis

Indications	Class	Level of evidence
High-dose corticosteroid in combination with antilymphocyte antibodies and/or calcineurin inhibitors (cyclosporine or tacrolimus) and/or antiproliferative drugs (azathioprine or mycophenolate mofetil)	I	B
Maintenance immunosuppression with a corticosteroid and a calcineurin inhibitor (cyclosporine or tacrolimus) or a triple-drug regimen with addition of an antiproliferative drug (azathioprine or mycophenolate mofetil)	I	B
Heart transplantation	I	B
Indication for implantable cardioverter-defibrillator for primary or secondary prevention of complex ventricular arrhythmias	I	B

Maintenance immunosuppression consists of a cyclosporine-based double or triple regimen.^270 , 278^However, there are important limitations to assessing the actual benefit. Combinations of prednisone, cyclosporine, azathioprine, and mycophenolate mofetil have been used, as well as use either alone or in combination with rabbit antithymocyte globulin or muromonab-CD3. Triple-drug immunosuppression has been shown to increase the chance of surviving and being transplant-free to 58% at 5 years.^191^However, immunosuppression must be maintained because of the possibility of recurrence. Combined immunosuppression (prednisone, cyclosporine, and azathioprine) appears to be more accepted, although other combinations have also been used, such as cyclosporine with rabbit antithymocyte globulin or rabbit antithymocyte globulin with high-dose corticosteroids. There are no comparative studies to confirm the best immunosuppression.^191 , 274^Cyclosporine combined with high-dose corticosteroids or muromonab-CD3 for 4 weeks decreases the degree of necrosis, cellular inflammation, and giant cells.^279^

Heart transplantation is indicated and has resulted in improved medium-term survival, but recurrence is 20% to 25%.^8 , 280^This is the treatment of choice despite a higher risk of rejection.^281^

### 6.3.2. Clinical manifestation and diagnosis

Giant cell myocarditis is recognized as a rapid and progressive disease, most often fatal unless heart transplantation is performed. Most cases are associated with an autoimmune process.

Giant Cell Myocarditis Study Group data showed that young, white adults were predominantly affected, with no sex difference. The most common manifestation was acute HF (75% of cases), but half of the patients developed complex ventricular arrhythmia in the course of the disease. Median heart transplant-free survival was 5.5 months.^8^

A more recent report on giant cell myocarditis showed a higher incidence in young adult women, and the main clinical manifestations were acute HF, AV block, and ventricular arrhythmias.^274^

Imaging studies do not show any specific changes in giant cell myocarditis. The diagnosis is based on characteristic findings on EMB, ie, a diffuse and mixed inflammatory infiltrate consisting predominantly of macrophages, followed in number by lymphocytes and typically dispersed multinucleated giant cells derived from macrophages, and, finally, by a lower proportion of eosinophils and plasma cells.^282^

## 6.4. Acute Chagasic myocarditis and reactivation

### 6.4.1. Clinical manifestations and modes of transmission, reactivation in immunosuppressed patients

In recent years, there has been an increasing number of cases of acute Chagas disease associated with both oral or vector-borne transmission and disease reactivation in Latin American countries. The main routes of acute Chagas disease transmission currently are oral (68.4%), vector-borne (5.9%), vertical (0.5%), transfusion (0.4%), accidental (0.1%), and unknown (24.7%), as described in a study of confirmed cases in the Brazilian Amazon.^19^

Vector-borne transmission occurs during or shortly after hematophagy, when triatomine bugs defecate and deposit their contaminated feces causing the infective forms of *Trypanosoma cruzi* to reach the skin, mucous membranes, and then the bloodstream. The incubation period ranges from 4 to 15 days. Oral transmission is associated with consumption of food or beverages contaminated with parasites. Currently, it is the most common cause of acute disease, causing outbreaks in endemic and nonendemic regions. The incubation period ranges from 3 to 22 days.^283^

Patients with acute Chagas disease may present with nonspecific signs and symptoms of infectious syndrome, such as fever, myalgias, facial edema, and arthralgias, in addition to signs related to the portal of entry such as a chagoma and Romaña sign in the vector-borne form and digestive bleeding in the oral form.^284^

Acute cases may or may not present with myocarditis and pericarditis. Autopsy reports have shown acute inflammation of the epicardium and myocardium with intense and diffuse inflammatory activity and extensive dissociation of cardiac fibers, with the amastigote forms of the parasite being observed.^285^Signs and symptoms compatible with HF have ranged from 26% to 58%. Severe cases with cardiac tamponade and cardiogenic shock from LV systolic dysfunction may occur. The lethality of oral transmission has ranged from 2% to 5% in the largest series. Abnormal findings on additional testing have ranged from 33% to 70% for ECG changes (right bundle branch block, first-degree AV block, acute atrial fibrillation, anterosuperior divisional block) and from 13% to 52% for echocardiographic changes. Pericardial effusion is the most frequent abnormality (10% to 82%), and segmental contraction changes, common in the chronic phase, are rarely found in the acute phase. Despite the occurrence of severe cardiac involvement, most patients show preserved systolic function with few cases of reduced EF. Most deaths are caused by significant pericardial effusion and cardiac tamponade.^286 , 287^

### 6.4.2. Diagnosis

Direct parasitology testing is the most indicated method for diagnosing acute myocarditis.^288^Indirect methods, such as blood culture and xenodiagnosis, have low sensitivity and thus are not suitable for the acute phase. Serology testing is not the best diagnostic method in the acute phase but may be performed when direct parasitology testing is persistently negative and clinical suspicion persists.

Wet mount examination to detect the parasite in the circulating blood is quick and simple, in addition to being more sensitive than stained smear examination. Ideal conditions for collection are the patient still being febrile and symptom onset having occurred within 1 month. Concentration methods (Strout, microhematocrit, buffy coat) are recommended when wet mount examination is negative, as they are more sensitive. They are also used when the acute course initiated over 1 month ago. Negative results in the first analysis should not be considered definitive, especially if symptoms persist, unless another etiology is proven.

PCR testing, being a molecular diagnostic technology, has become an important method to show recent infection, as it yields positive results days to weeks before circulating trypomastigotes are detected.^289 - 291^Peripheral blood and tissue collected on EMB can be used to detect early reactivation after heart transplantation, before the onset of clinical symptoms or graft dysfunction.^292^

Chagas disease reactivation in the post-heart transplant period ranges from 19.6% to 45%.^293^The condition may present as acute myocarditis with various degrees of HF, often accompanied by systemic manifestations. Erythema and subcutaneous nodules may appear on the skin and should be biopsied to identify amastigote nests. Monitoring should be routine, even if reactivation is not suspected. When there are no extracardiac clinical signs, biopsy should be performed.

### 6.4.3. Treatment

Trypanosomicidal drug treatment is indicated for patients with acute Chagas disease with or without manifestations of myocarditis and for those with chronic disease reactivation due to immunosuppression (transplanted patients) ( Table 27 ).^294^

**Table 27:** - Recommendations for the etiological treatment of acute Chagasic myocarditis

Indications	Class	Level of evidence
Acute infection, irrespective of mechanism of transmission	I	C
Reactivation of chronic *T. cruzi* infection	I	C

Benznidazole is the currently available drug for the treatment of *T. cruzi* infection.^295^Information, however, is based on nonrandomized studies with insufficient number of patients and follow-up duration. Although the definition of cure criteria remains controversial, there is a current consensus that benznidazole treatment should be performed in the acute phase and provides a likely long-term benefit.^296^

Benznidazole dose range in children is 5 to 10 mg/kg per day in two divided doses for 60 days. The adult dose is 5 mg/kg. Adverse reactions manifest in approximately 30% of patients, most frequently allergic dermatitis (30%) and peripheral sensory neuropathy (10%).

## 6.5. Myocarditis due to tropical diseases

Tropical diseases are infectious entities generally transmitted by vectors in tropical regions. Governments tend to ignore this issue and provide limited resources to control these diseases, which affects vulnerable populations in areas with inadequate sanitation and deficient health systems. The Brazilian Amazon is an endemic region for tropical diseases, although other regions of the country are also affected. Many tropical diseases may cause myocarditis and appear to contribute to the increased burden of heart disease in developing countries.^297^The tropical diseases that cause myocarditis and are prevalent in Brazil are malaria, dengue, chikungunya, Zika, and yellow fever ( Table 28 ). These diseases should be considered in the investigation of myocarditis occurring in endemic areas.

**Table 28:** – Characteristics of the main causes of tropical myocarditis

	Agent	Vector	Clinical manifestations
**Malaria**	*Plasmodium spp* (protozoan)	*Anopheles* mosquito	**Mild form:** fever, chills, headache, myalgias, and malaise **Severe form:** shock, seizures, mental confusion, renal failure, acute respiratory distress syndrome, coma, and death – There may be asymptomatic patients, especially in endemic regions
**Dengue**	Dengue virus	*Aedes aegypti* mosquito	**Mild form:** fever, headache, myalgia, arthralgia, retro-orbital pain and maculopapular rash, nausea, vomiting **Severe form (with warning signs):** severe abdominal pain, persistent vomiting (≥3 times/24 h), epistaxis, gingival bleeding, fatigue, restlessness or irritation, hematemesis or melena, altered mental status – Approximately 50% of patients have symptoms
**Chikungunya**	Chikungunya virus	*Aedes aegypti* mosquito	**Mild form:** fever, rash, arthralgia, myalgia, edema, and headache **Severe form:** severe neurological disease, myocarditis,and multiple organ failure **Chronic form:** persistent arthralgia and myalgia with associated edema, mostly in the wrists, hands, ankles, and feet. It might last for months or even years and result in disability – Approximately 80% of patients are symptomatic
**Zika**	Zika virus	*Aedes aegypti* mosquito	**Mild form:** fever (usually mild), rash, arthralgia, arthritis, myalgia, headache, conjunctivitis, and edema **Severe form:** need for hospitalization is uncommon and deaths are rare **Congenital form:** anomalies of the eye, heart, and brain such as microcephaly (most common) – Only about 20% of patients have symptoms
**Yellow fever**	Yellow fever virus	*Haemagogus* (wild) and *Aedes aegypti* (urban) mosquitoes	**Mild form:** sudden-onset fever, chills, headache, myalgia, weakness, fatigue, nausea, and eye redness **Severe form (toxic phase):** high fever, jaundice, epigastralgia, bleeding, hemorrhagic diathesis (hematemesis), shock, and organ failure – Approximately 50% of patients are symptomatic

Malaria is caused by the protozoans of the genus *Plasmodium* (in Brazil, the species *P. vivax* and *P. falciparum* ), transmitted through the bites of *Anopheles* mosquitoes. Malaria is endemic in the Amazon region, where over 155,000 cases were diagnosed in 2019. *P. falciparum* causes the most severe forms of the disease and has been more significantly associated with the development of myocarditis.^298^Autopsy studies of severe cases of malaria show a large number of parasites in the myocardium and inflammation compatible with myocarditis. Most studies reporting on malarial myocarditis consist of inpatient case series assessed with ECG, myocardial injury markers, and echocardiogram.^299^These case series include severe cases and show changes in cardiac injury markers in up to 59% and echocardiographic changes such as reduced systolic function in up to 19% of patients. Many studies associating malaria with acute MI fail to properly define the evaluated outcome, with probable cases of myocarditis being described as infarctions. In acute malaria progressing to the severe form of the disease, myocardial dysfunction due to malarial myocarditis should be considered. Assessment with biomarkers of myocardial injury and ventricular function should be considered to optimize cardiovascular management.

Arboviruses cause infectious diseases such as dengue, Zika, chikungunya, and yellow fever. They are transmitted through the bites of *Aedes aegypti* mosquitoes. Cardiovascular involvement has been demonstrated especially in dengue, which is the most prevalent arboviral infection in Brazil. Dengue is also the disease with the highest rate of reported cardiovascular manifestations -- prospective studies have shown that 48% of patients with the severe form develop myocarditis. An autopsy study of four fatal cases of dengue revealed findings of myocarditis with edema, hemorrhage, mononuclear infiltrates, and presence of antigens and viral replication.^300^

Of all the previously mentioned arboviral diseases, chikungunya is the most symptomatic (80% of cases); however, it normally presents with mild symptoms and mostly affects joints and muscles. Still, the infection may manifest systemically and cause widespread damage or affect specific organs such as the heart. A case report of a patient with chikungunya who developed chest pain showed typical findings of myocarditis on MRI.^301^Several case series in epidemic settings have reported up to 37% of cardiovascular involvement, generally compatible with myocarditis.^302^

Of all the tropical infections discussed herein, Zika is the most recently discovered and has the highest percentage of asymptomatic cases; when there are clinical manifestations, these are predominantly congenital and involve the neurological system. Nonetheless, a few longitudinal studies have addressed nonneurological complications of this infection in adults and reported cardiovascular outcomes such as HF, arrhythmias, and acute MI, as well as Zika-associated myocarditis.^303 , 304^Also, prospective studies of congenital Zika have reported echocardiographic changes suggestive of cardiovascular damage. These findings, however, possibly do not represent the actual impact of Zika on heart disease, as there is a lack of longitudinal studies.

Yellow fever is a neglected tropical arboviral disease that was restricted to the sylvatic cycle for a long time, with low incidence (underreporting) and limited geographic expansion, which contributed to few cases being studied and adequately reported, especially those involving the cardiovascular system. Still, with the increasing urbanization of this disease and the better understanding of its pathophysiological mechanisms, some studies have demonstrated a relationship with the heart. The PROVAR+ study, for example, reported, respectively, 48% and 52% of echocardiographic and electrocardiographic changes.^305^Also, postmortem studies have isolated the virus in cardiac tissue or demonstrated myocardial damage.

Therefore, although the association between tropical diseases and myocarditis is based on case series and few studies with a well-defined diagnosis of myocarditis, the diagnostic investigation of common regional diseases is warranted in cases of myocarditis in endemic areas. To this end, antigen screening or serology testing for arboviral infections and thick blood smear examination for malaria should be included. Once these diseases are diagnosed, an infectious disease specialist should be consulted to guide the specific treatment of malaria or supportive treatment of arboviral diseases. Another clinical situation consists of patients with confirmed arboviral infection or malaria which progresses to a severe form, especially shock; in these cases, cardiac injury should be evaluated with markers of myocardial necrosis and myocardial function, and echocardiography should be used for the diagnosis of myocardial involvement (myocarditis). Management should include optimization of myocardial function.

## 6.6. Covid-19-related myocarditis

Human coronaviruses have been linked to myocarditis.^306 - 308^During the Toronto severe acute respiratory syndrome (SARS) outbreak, SARS-CoV RNA was detected in 35% of autopsied hearts.^309^This increases the possibility of direct viral damage to cardiomyocytes^310 - 312^( Table 29 ).^313^

**Table 29:** – Representative studies addressing the acute cardiovascular manifestations of coronavirus infection and their clinical implications311-313

Virus	Sample size	Cardiovascular manifestations	Outcomes
SARS	N=121	Hypotension, tachycardia, bradycardia, cardiomegaly, and arrhythmia	Mostly transient
N=15	Cardiac arrest	Death
N=46	Subclinical diastolic impairment without systolic involvement on echocardiography	Reversible on clinical recovery
MERS	N=1	Acute myocarditis and acute-onset heart failure	Recovered
Covid-19	N=14	Myocardial injury (manifesting with increased high-sensitivity cardiac troponin I) in five patients	Four patients required intensive care
N=138	Acute cardiac injury (7.2%), shock (8.7%), and arrhythmia (16.7%)	Most patients required intensive care

*Source: Adapted from Xiong et al.^313^*

### 6.6.1. Possible pathophysiology of SARS-CoV-2-related myocarditis

The mechanisms of myocardial injury are not well established but probably involve the following: myocardial injury secondary to oxygen supply-demand mismatch; microvascular injury; systemic inflammatory response; stress cardiomyopathy; acute nonobstructive coronary syndrome; and direct viral myocardial injury^314^( Figure 11 ).^315^

**Figure 11:** – Potential mechanisms of myocardial injury in Covid-19.

MI: myocardial infarction; DIC: disseminated intravascular coagulation. Source: Adapted from Atri D et al.^315^

### 6.6.2. Direct viral myocardial injury

Case reports of myocarditis in patients with Covid-19 provide evidence of cardiac inflammation but do not determine the mechanism. SARS-CoV-2 infection is caused by the binding of viral surface spike protein to the human angiotensin-converting enzyme 2 (ACE2) receptor. However, the spike protein must first be cleaved at the S1/S2 sites and subsequently at the S2’ sites to enable binding to ACE2. Cleavage at the S1/S2 site appears to be mediated by transmembrane serine protease 2 (TMPRSS2)^316 , 317^( Figure 12 ).^318^

**Figure 12:** – Proposed pathophysiology of SARS-CoV-2 myocarditis. SARS-CoV-2 uses the spike protein (primed by TMPRSS2) to bind ACE2 to allow cell entry. Intracellular SARS-CoV-2 might impair stress granule formation via its accessory protein. Without the stress granules, the virus is allowed to replicate and damage the cell. Naïve T lymphocytes can be primed for viral antigens via antigen-presenting cells and cardiotropism by the heart-produced HGF. The HGF binds c-Met, an HGF receptor on T lymphocytes. The primed CD8+ T lymphocytes migrate to the cardiomyocytes and cause myocardial inflammation through cell-mediated cytotoxicity. In the cytokine storm syndrome, in which proinflammatory cytokines are released into the circulation, T-lymphocyte activation is increased and releases more cytokines. This results in a positive feedback loop of immune activation and myocardial damage.

ACE2: angiotensin-converting enzyme 2; APC: antigen-presenting cell; HGF: hepatocyte growth factor; IL-6: interleukin 6; MHC: major histocompatibility complex; SARS-CoV-2: severe acute respiratory syndrome coronavirus 2.

Source: Adapted from Siripanthong B et al.^318^

To date, there is only one report of biopsy-proven SARS-CoV-2 myocarditis with viral inclusions or viral DNA detected in myocardial tissue.^319^However, viral particles were not present in cardiomyocytes, only within macrophages in the cardiac interstitium. Another hypothetical mechanism of direct viral myocardial injury is through infection-mediated vasculitis. The ACE2 receptor is highly expressed in endothelial arteries and veins.^320^

ACE2 expression is limited in cardiomyocytes but high in pericytes. Covid-19 might attack pericytes, essential for endothelial stability, causing endothelial dysfunction, which leads to microcirculatory disturbances. This explains why, although ACE2 expression is limited in cardiomyocytes, Covid-19 might cause cardiac injury.^320^Autopsies have shown inflammatory infiltrates consisting of macrophages and, to a lesser extent, CD4+ T cells.^321 - 322^These mononuclear infiltrates are associated with regional necrosis of cardiomyocytes which, according to the Dallas criteria, defines myocarditis.^323^

### 6.6.3. Diagnosis of Covid-19-related myocarditis

The clinical presentation of SARS-CoV-2 myocarditis ranges from mild symptoms such as fatigue, dyspnea, and chest pain to severe cases of cardiogenic shock. Patients may present with signs of right HF, with increased jugular venous pressure, peripheral edema, and right upper quadrant pain. The most emerging presentation is fulminant myocarditis, defined as ventricular dysfunction and HF within 2 to 3 weeks of viral infection. Early signs of fulminant myocarditis frequently resemble those of sepsis.^14 , 324 - 329^

### 6.6.4. Laboratory

Troponin and NT-proBNP elevations have been observed in cases of Covid-19 myocarditis.^14 , 312 , 324 - 326^

Abnormal troponin levels are common in patients with Covid-19, especially when high-sensitivity cardiac troponin (hs-cTn) is used. Studies evaluating the clinical course of Covid-19 have reported detectable hs-cTnI in most patients, and hs-cTnI was significantly high in more than half of patients who died.^327 , 328^

Patients with Covid-19 generally demonstrate significantly elevated BNP or NT-proBNP. The significance of this finding is uncertain and should not necessarily trigger an evaluation or treatment for HF unless there is clear clinical evidence for diagnosis. In patients with Covid-19, increased BNP or NT-proBNP levels may also be secondary to myocardial stress as a possible effect of severe respiratory disease.

Because of the low frequency and nonspecific nature of abnormal troponin or natriuretic peptide levels in patients with Covid-19, measurements should only be performed if the clinical diagnosis of acute MI or HF is under consideration. Abnormal troponin or natriuretic peptide results should not be considered evidence of acute MI or HF without additional diagnostic evidence.^329^

### 6.6.5. Electrocardiogram

ECG changes commonly associated with pericarditis, such as ST-segment elevation and PR-segment depression, are seen in myocarditis;^310^however, these findings are not sensitive enough to detect the disease, and their absence does not exclude the diagnosis.

For example, a patient with Covid-19-related myocarditis has shown neither ST-segment elevation nor PR-segment depression.^330^Other ECG abnormalities, including new bundle branch block, prolonged QT interval, pseudoinfarction pattern, ventricular extrasystoles, and bradyarrhythmia with advanced AV block, might be seen in myocarditis.^331^

Recently, a case series of patients with confirmed Covid-19 who presented with, at some point in the infection, ST-segment elevation on ECG was published.^332^

### 6.6.6. Imaging

A recent ESC document lists the conditions that must be considered in a situation requiring the use of any cardiovascular imaging method in patients with Covid-19: imaging studies should only be performed if the management is likely to be changed by the results or if saving a patient’s life is at stake; the best imaging modality to meet the demand should be used considering the safety of the medical team regarding exposure; nonurgent, elective, or routine examinations should be postponed or even canceled.^333^

Thus, transthoracic echocardiography, despite playing a key role in the cardiovascular work-up of these patients, should not be routinely indicated in view of the current Covid-19 pandemic, and specific cases require careful consideration.^334^

Recent Society of Cardiovascular Computed Tomography (SCCT) recommendations for the use of coronary CT angiography in patients with Covid-19 include acute HF of unknown cause^335 , 336^( Table 30 ).^337^

**Table 30:** - Society of Cardiovascular Computed Tomography (SCCT) recommendations for the use of coronary computed tomography angiography in the context of Covid-19

Urgency category	Clinical conditions	Timing for examination
Elective	Asymptomatic or stable coronary artery disease Cardiomyopathy or stable structural heart disease (valvular, TAVI or AF ablation planning, congenital) Benign masses	> 8 weeks
Semiurgent	Chronic AF cardioversion Chronic or subacute prosthetic valve dysfunction	Within 4 to 8 weeks
Urgent	High-risk acute or stable chest pain Urgent structural interventions (TAVI, left atrial appendage occlusion, etc.) or acute AF cardioversion Acute heart failure of unknown cause Acute valve (or prosthetic valve) dysfunction Malignant mass biopsy planning	Within hours to <2 to 4 weeks (depending on severity)

*Rule out thrombi when CMR is not feasible. AF: atrial fibrillation; TAVI: transcatheter aortic valve replacement. Source: Adapted from Araujo-Filho et al.^337^*

The ESC document suggests that positive troponins and myocardial dysfunction or severe arrhythmia not explained by other methods may be an indication for CMR if the diagnosis is crucial for the treatment and the patient is stable enough to be safely transferred and undergo the procedure.^334^

In this context, current Society for Cardiovascular of Magnetic Resonance (SCMR) guidance suggests that CMR examinations should be considered judiciously and individually in cases of suspected acute myocarditis with immediate implications for patient management.^337^If CMR is performed, the results should be interpreted according to the LLC: (1) edema; (2) irreversible cell injury; and (3) hyperemia or capillary leakage^338^( Table 31 ).^337^

**Table 31:** - Society for Cardiovascular of Magnetic Resonance (SCMR) recommendations for the use of cardiac magnetic resonance (CMR) imaging in the context of Covid-19

Clinical conditions	Suggested timing for examination
Investigation of ischemia and myocardial viability to guide urgent revascularization	Within 1 week depending on severity
Suspected intracardiac mass or thrombus with contraindication to anticoagulation or in patients with suspected embolic events
Urgent ablation planning in unstable patients with severe arrhythmias
Pericardial constriction requiring potentially urgent surgery
Planning for percutaneous implantation of a prosthetic heart valve requiring urgent surgery

*Note 1: Choices based on expert consensus. Note 2: Individual clinical status and contraindications to examination must be considered. Source: Araujo-Filho et al.^337^*

### 6.6.7. Endomyocardial biopsy

The AHA and the ESC recommend EMB for the definitive diagnosis of myocarditis, but both societies recognize its limitations.^339 , 340^In the SARS-CoV-2 era, the clinical utility and the role of EMB, currently the gold standard for the diagnosis of myocarditis, remain uncertain; also, performing noninvasive imaging, such as echocardiography and CMR, with adequate safety and isolation measures has been challenging.^341 , 342^

Another point to consider is that, in some cases, SARS-CoV-2 infection may not initially manifest through clear signs and symptoms of interstitial pneumonia but instead as myocarditis without respiratory symptoms, sometimes complicated by cardiogenic shock with a fulminating course.^14 , 316^

Additionally, there is limited evidence regarding the therapeutic treatment of SARSCoV-2-associated myocarditis. A case report showed that early therapy with glucocorticoids and immunoglobulins was beneficial to the patient.^316^Corticosteroids have been used in several viral respiratory infections (influenza, SARS-CoV, and MERS-CoV) with reports of limited benefit and, in some cases, delayed viral clearance and increased mortality.^333^

However, the ESC Working Group on Myocardial and Pericardial Diseases recommends the use of steroids in myocarditis due to proven autoimmune diseases and virus-negative myocarditis only after active infection is assessed on EMB.^340^Clearly, EMB is not always available in real-world practice, and its role in SARS-CoV-2-related myocarditis remains unknown. Furthermore, in the absence of randomized multicenter trials, routine use of immunoglobulin is also not recommended.

In conclusion, there are significant gaps in the assessment of MI in patients with SARS-CoV-2 infection that require a thorough diagnostic evaluation, prioritized treatments, and more aggressive strategies,^318 , 319^if necessary, especially in those who develop cardiogenic shock during fulminating myocarditis^332 - 342^( Figure 13 ).^318^

**Figure 13:** – Suggested diagnostic and management protocol for SARS-CoV-2-related myocarditis.

ACS: acute coronary syndrome; CMR: cardiovascular magnetic resonance; CRP: C-reactive protein; CT-CA: computed tomography-coronary angiogram; DCCV: direct current cardioversion; ECG: electrocardiogram; ECMO: extracorporeal membrane oxygenation; ESR: erythrocyte sedimentation rate; IABP: intra-aortic balloon pump; IVIg: intravenous immunoglobulin; QT: QT interval; VAD: ventricular assist device. Source: Adapted from Siripanthong et al.^318^

## 6.7. Acute cardiotoxicity of antineoplastic therapy

### 6.7.1. Antineoplastic agents inducing acute cardiotoxicity

The evolution of cancer treatment in recent decades has resulted in improved survival and quality of life for patients.^343^Simultaneously, however, increased longevity has led to longer exposure to cardiovascular risk factors, in addition to the potential risk of cardiovascular injury induced by chemotherapy, radiotherapy, and immunotherapy.^344^Recent studies have demonstrated that there are two periods of increased occurrence of cardiovascular disease in patients with cancer: the first year after diagnosis and the years after cure, when patients are called survivors, a group that has shown significantly increased cardiovascular mortality.^345 , 346^

Myocarditis is an emerging toxicity of relevance. Recently, cancer treatment-related myocarditis has been noted as a result of the evolution of immunotherapy, more specifically immune checkpoint inhibitors (ICIs).^347 , 348^However, it is potentially associated with any therapy that modulates the immune system. Identifying myocarditis in oncology clinical trials is challenging given its relatively low incidence and high mortality rate.

Importantly, the following recommendations are based on expert consensus given the paucity of scientific data on this topic.

The classic model of cardiotoxicity consists of ventricular dysfunction caused by anthracyclines.^349^Anthracyclines are a class of chemotherapeutic drugs still widely used today. HF affects up to 30% of patients and usually appears months after treatment, being related to a cumulative dose above 300 mg/m^2^. Most cases are subacute or chronic and manifest months and even years after treatment, with irreversibility being the predominant characteristic. Anthracycline-induced acute myocarditis is a rare manifestation that has no relationship with dose and is reversible in most cases.^350^The mechanism of toxic action is directly linked to the oxidative stress resulting from anthracycline metabolism, in addition to the inhibition of topoisomerase IIb, which ultimately leads to cardiomyocyte DNA damage through mitochondrial dysfunction and apoptosis.^351^

Cyclophosphamide is a nitrogen mustard that acts as an alkylating agent and is usually included in chemotherapy regimens involving the concomitant use of anthracyclines. It may result in acute toxic myocarditis with multifocal hemorrhage, characterized by endothelitis, hemorrhagic capillaritis, and thrombogenesis.^352^

ICIs currently are the most commonly studied model for inducing myocarditis, especially nivolumab, durvalimab, ipilimumab, pembrolizumab, and atezolizumab.^353^This therapy has revolutionized cancer treatment in recent years by improving the survival of patients with lung cancer, head and neck cancer, renal carcinoma, and melanoma, among others.^354^The mechanism of action consists of blocking the apoptosis of T lymphocytes (anti-CTLA4, anti-PD1, anti-PDL1), culminating in the activation of lymphocytes throughout the body. If this, on the one hand, reactivates the lymphocytes and antitumor immunity, on the other hand, activated T lymphocytes might trigger severe myocarditis, which is fatal in up to 50% of cases. Clinically, it affects 0.2% of patients and manifests, on average, 30 to 90 days after starting treatment.^355 , 356^

### 6.7.2. Diagnosis of acute cardiotoxicity

Myocarditis in patients with cancer should be diagnosed in situations of cardiac conditions without an alternative primary diagnosis (eg, acute coronary syndrome, trauma, etc.).^357^Clinical history should consider drug regimen, treatment duration, and other comorbidities. Laboratory diagnosis includes the measurement of biomarkers such as hs-cTn and NT-proBNP. In immunotherapy-related myocarditis, CPK measurement is also recommended because of an association with myositis in up to 20% of cases.^358^

An ECG may be useful for confirming suspected myocarditis. Possible findings are ventricular arrhythmias, ST-T wave abnormalities, PR-segment changes, bradycardias, and blocks.^357^

Echocardiography is the imaging test of choice for a diagnostic approach to myocarditis. It is performed at baseline and during follow-up to assess function over time. The most common findings include diffuse systolic dysfunction, segmental abnormalities, changes in ventricular sphericity, increased wall thickness, pericardial effusion, and strain changes.^357^

MRI is the most sensitive imaging modality for the diagnosis of myocarditis and can determine the prognosis. A combination of MRI findings has been termed the Lake Louise criteria (LLC) for the diagnosis of acute myocarditis. Many advances have occurred in the diagnosis of myocarditis via MRI, including improved tissue characterization using T1 and T2 mapping and extracellular calcium.^359^

EMB may be considered for investigation of chemotherapy- and immunotherapy-related myocarditis. Experts recommend performing a biopsy whenever possible because in many cases, before significant clinical manifestations, pathologic findings already show the severity of the pathogenic changes of cancer myocarditis.^360^

We describe below the main antineoplastic agents that potentially induce myocarditis and their manifestations ( Table 32 ).

**Table 32:** - Characteristics of cancer treatment-induced myocarditis

	Anthracyclines	Cyclophosphamide	Immune checkpoint inhibitors
**Incidence**	10%	10%	0.2%
**Mortality**	20%	20%	50%
**Clinical manifestation**	Acute HF	Acute HF	Acute HF
**Diagnosis**	Clinical, laboratory, imaging, and biopsy	Clinical, laboratory, imaging, and biopsy	Clinical, laboratory, imaging, and biopsy
**Reversibility**	Usually reversible	Usually reversible	Usually irreversible
**Reexposure**	Possible	Possible	Not recommended

*HF: heart failure.*

### 6.7.3. Treatment of acute cardiotoxicity

When a diagnosis is suspected, treatment should be started immediately, as timing is an important factor to determine the course of the disease. Although there are no large prospective studies to guide treatment in ICI-associated myocarditis, immunosuppression is the cornerstone of treatment.

Intravenous steroids are widely used in immune-related adverse events (irAEs) and may be effective in ICI-associated myocarditis.^347^High-dose corticosteroids (eg, methylprednisone 1000 mg daily for 3 days followed by prednisone 1 mg/kg) are commonly used and may be associated with better outcomes.^22^Mahmood et al.^22^reported that 31% of 35 patients received corticosteroids, and that high-dose steroids were associated with lower peak troponin levels and lower rates of major adverse cardiac events (MACEs) compared to lower doses of corticosteroid. The American Society of Clinical Oncology (ASCO) recommends an initial corticosteroid dose of 1 mg/kg.^361^Therapy duration is unclear, but ASCO recommends tapering over 4 to 6 weeks in patients with irAEs. Serum cardiac biomarkers (eg, troponins, BNP) may be useful for defining the need for longer therapy duration after weaning.

Additional immunosuppression may also be used. Anecdotal evidence suggests that other immunosuppressants such as IVIg,^362^infliximab,^363^mycophenolate mofetil,^364^tacrolimus,^362^antithymocyte globulin,^365 , 366^plasmapheresis,^362^abatacept,^367^and alemtuzumab^368^may be effective. In a study conducted by Mahmood et al.,^22^a small number of patients received other nonsteroidal immunosuppressants; given the lack of robust data on their efficacy in ICI-associated myocarditis, such agents are generally reserved for refractory or very severe cases.

We suggest considering the addition of nonsteroidal immunosuppression in patients who do not show symptomatic, functional, or biomarker improvement within 24 to 48 hours of corticosteroid initiation. The choice of the second agent is not defined but may be motivated by availability and contraindications. Several sequential immunosuppressants may be required to achieve remission.^22^

We recommend initiating high-dose intravenous steroids at the time of diagnosis of ICI-associated myocarditis (methylprednisone 1 mg/kg/day). Cardiac biomarkers (troponin and BNP) should be measured sequentially. If cardiac biomarkers continue to increase despite the use of high-dose steroids, plasmapheresis should be initiated. An additional immunosuppressant may be required if cardiac biomarkers continue to increase or if the patient presents with new or more severe arrhythmias or HF ( Figure 14 ). The choice of immunosuppressant depends on local experience and coexisting comorbidities ( Table 33 ).

**Figure 14:** – Proposed therapeutic course with immunosuppression, biomarkers, and ventricular function assessment.

CMR: cardiac magnetic resonance; ECHO: echocardiogram; IV: intravenous.

**Table 33:** - Immunosuppressants used in the treatment of immune checkpoint inhibitor-associated myocarditis

Immunosuppressant	Class	Dose	Start	Duration
**Methylprednisolone**	Corticosteroids	1 mg/kg/day	At diagnosis	2 to 3 days
**Prednisone**	Corticosteroids	40 to 60 mg/day	Day 2-3	Slowly wean over 4 to 8 weeks
**Infliximab**	TNF-alpha inhibitor	5 mg/kg	Day 4-5	Single dose (may be repeated within a few months)
**Antimonocyte globulin**	?	10 to 30 mg/kg	Day 2-3	7 to 14 days
**Tacrolimus**	Calcineurin inhibitor	0.10 to 0.15 mg/kg/day	?	?
**Mycophenolate mofetil**	IMPDH inhibitor	1g 2×/day	?	?
**Abatacept**	CTLA-4 agonist	500 mg every 2 weeks	Day 7-14	Total of 5 doses
**Alemtuzumab**	Anti-CD52	30 mg	?	Single dose

*IMPDH: inosine monophosphate dehydrogenase.*

We recommend administering a single dose of infliximab (5 mg/kg) in the absence of contraindications (eg, tuberculosis, hepatitis). Alternatively, antithymocyte globulin (10 to 30 mg/kg), alemtuzumab (30 mg once), or abatacept (500 mg) may be used. Within 3 to 5 days of corticosteroid initiation, ventricular function should be examined by echocardiography or CMR. Patients showing significantly improved LV function (improvement in LVEF of at least 5%) can be converted to oral corticosteroids (prednisone 40 to 60 mg daily) for a long period (4 to 8 weeks). If biomarkers decline and the patient shows a clinical response, mycophenolate mofetil or tacrolimus can be used to shorten steroid chronicity. Given the high mortality and morbidity of immune-related myocarditis, ICIs should be discontinued even in patients with mild cardiotoxicity ( Table 33 ).

Because of the potential reversibility of ICI-associated myocarditis, supportive therapies may be instituted after careful multidisciplinary consideration of the underlying malignancy status and recovery potential. Supportive strategies may include inotropic support, temporary or permanent pacemaker, and temporary mechanical circulatory support (eg, intra-aortic balloon pump,^6^percutaneous ventricular assist devices,^369^or ECMO).^362 , 364^A careful RV assessment should be performed prior to insertion of LV assist devices, as ICI-associated myocarditis is highly likely to affect the RV,^362 , 364 , 369^which may require biventricular support.^363 , 369^Furthermore, owing to the prothrombotic environment induced by the underlying neoplasm and irAEs, excluding LV thrombi with CMR or contrast-enhanced echocardiography before insertion of percutaneous LV assist devices is essential.^363^Drug therapy for HF should be initiated as tolerated. This includes angiotensin blockers (ACEI, ARB, angiotensin receptor-neprilysin inhibitor [ARNi]), beta-blockers, and mineralocorticoid antagonists (eg, spironolactone).

The safety of restarting ICI therapy after myocarditis has resolved is unknown. In a study of 40 patients who developed irAEs (1 ICI-associated myocarditis) and had ICIs reintroduced (43% used the same agent), 22 (55%) developed recurrent irAEs during a 14-month follow-up period. Extrapolating these data to ICI-associated myocarditis and considering the high probability of recurrent irAEs with reintroduction, ASCO recommends permanent discontinuation of ICIs in all cases of immune-related myocarditis.^370^There is a report of successful reintroduction in one case of mild myocarditis.^371^Also, reintroduction of ICI might be attempted in select cases of mild, asymptomatic (grade I) ICI-associated myocarditis,^361^especially with low-risk ICIs such as pembrolizumab. However, this recommendation remains controversial.

### 6.7.4 Prognosis

The prognosis of ICI-associated myocarditis is challenging because of the rare nature of this condition. In a multicenter registry of 35 patients with ICI-associated myocarditis, nearly half (n = 16) developed MACEs over 102 days (6 cardiovascular deaths, 3 cardiogenic shocks, 4 cardiac arrests, and 3 complete heart blocks).^22 , 347^In a French registry of 30 patients with ICI-associated myocarditis at two centers, eight patients died from cardiovascular complications. A recent study that followed-up 101 patients with ICI-associated myocarditis showed a MACE rate of 51% during a 162-day follow-up period.^347^The mortality rate among 250 patients with ICI-associated myocarditis reported to the US Food and Drug Administration Adverse Event Reporting System (FAERS) was 50%.^361^There was no difference in mortality rate by age, sex, year of reporting, or ICI type (antiprogrammed cell death protein-1/ligand-1 vs. anticytotoxic T-lymphocyte protein-4).^362^Mahmood et al.^22^found that patients with ICI-associated myocarditis and elevated troponin level at the time of discharge had significantly higher rates of MACEs (discharge troponin T ≥1.5 ng/mL: HR: 4.0; 95% CI: 1.5-10.9; p = 0.003). Escudier et al.^348^reported that 80% of patients with ICI-associated myocarditis and conduction disorder had cardiovascular death. A recent study of patients with ICI-associated myocarditis reported that global longitudinal strain obtained at diagnosis was strongly associated with MACEs over a 162-day follow-up period.^363^Given the small number of patients in those studies, it is difficult to identify risk factors contributing to a poor prognosis in patients with ICI-associated myocarditis.^364 - 368^

Overall, recovery rates with appropriate therapy have been substantial. A total of 67% of patients receiving steroids showed recovered LV function in a French registry on ICI-associated myocarditis.^347^Recovery has also been described in patients with fulminant ICI-associated myocarditis requiring mechanical circulatory support.^369 - 374^

### 6.7.5. Prevention

Most published studies on the prevention of chemotherapy-induced cardiotoxicity have focused on anthracyclines and anti-HER2 agents.

Prevention of cardiotoxicity should start before cancer treatment with an evaluation of cardiovascular risk and a conversation between the cardiologist and the oncologist in order to plan the best approach during cancer treatment.

Patients at higher risk of developing cardiotoxicity are those with classic risk factors for cardiovascular disease (hypertension, diabetes mellitus, dyslipidemia, smoking, obesity, and sedentary behavior, among others) or those with higher exposure to cardiotoxic drugs (high cumulative doses of anthracyclines, cardiotoxic drug combinations, and a history of chemotherapy or radiotherapy).^375 , 376^

The main recommendations for preventing cardiotoxicity are described in Table 34 .

**Table 34:** - Measures to prevent cardiotoxicity

Chemotherapeutic drug	Cardioprotective measure
	Identify cardiovascular risk factors
Treat comorbidities (hypertension, diabetes mellitus, dyslipidemia, smoking, sedentary behavior, obesity)
Moderate-intensity aerobic exercise
Arrhythmias: avoid QT prolonging drugs, manage electrolyte abnormalities
Minimize cardiac radiation
**Anthracyclines**	Limit cumulative dose (mg/m2):
	Daunorubicin <800
Doxorubicin <360
Epirubicin <720
Mitoxantrone <160
Idarubicin <150
	Use liposomal formulations
	Perform continuous infusions
	Use less cardiotoxic analogues (epirubicin, idarubicin)
	Assess use of cardioprotective drugs (dexrazoxane, ACEIs, beta-blockers, statins)
**Trastuzumab**	Assess use of cardioprotective drugs (ACEIs, beta-blockers)

*ACEIs: angiotensin-converting enzyme inhibitors; QT: QT interval. Source: Adapted from Zamorano et al.^377^*

Dexrazoxane, an iron chelator, is the only cardioprotective drug that has been approved for prevention of cardiotoxicity. Its protective effect against anthracycline cardiotoxicity has been proven in several studies addressing both the adult and pediatric populations.^376 - 383^The limitations of dexrazoxane are the high cost and some potential adverse effects, such as interference in the efficacy of anthracyclines, risk of secondary tumor development (controversial evidence),^384 , 385^and bone marrow toxicity. Dexrazoxane is indicated for adults with advanced or metastatic breast cancer who have previously received a cumulative dose of 300 mg/m^2^of doxorubicin, 540 mg/m^2^of epirubicin, when continuing treatment with anthracyclines is required.

The use of cardiovascular drugs such as beta-blockers, ACEIs, and ARBs in the prevention of cardiotoxicity secondary to anthracyclines is controversial and has been based on few clinical trials.^386 - 391^Some evidence has shown benefits of beta-blockers and ACEIs in patients who have used high cumulative doses of anthracyclines or in high-risk patients with positive troponin during chemotherapy.^386 , 390^At lower cumulative doses of anthracycline, this benefit has not been shown with beta-blockers,^389 , 392^but there has been a slight preventive effect with ARBs.^389^

The CECCY trial,^392^a Brazilian study which tested the use of beta-blockers for primary prevention of anthracycline-induced cardiotoxicity, did not demonstrate a preventive benefit of carvedilol. However, carvedilol was associated with reduced troponin levels and a lower percentage of patients with onset of diastolic dysfunction.

Regarding the use of trastuzumab, some studies suggest a benefit in the prevention of cardiotoxicity^393 , 394^as well as after the onset of cardiotoxicity by helping in the recovery of ventricular dysfunction.^395^The decision to discontinue or restart chemotherapy must be made jointly by weighing the risks and benefits of maintaining cancer treatment.

## 6,8. Myocarditis in children and adolescents

### 6.8.1. Causal factors

Myocarditis in children and adolescents has a particular etiology, and underdiagnosis may occur because its initial presentation is similar to those of a number of common viruses in childhood. Over 83% of patients are estimated to attend the emergency department at least twice before diagnosis.^396^In retrospective analyses, chest pain has been predominantly reported in children over 10 years of age, and the most commonly observed signs in younger patients have been tachypnea, fever, and respiratory distress^397^( Table 35 ). The application of diagnostic algorithms in emergency rooms has shown promising results, including the possibility of increasing the number of suspected patients ( Figure 15 ).^239 , 398^Regarding etiology, studies evaluating the collection of a viral panel in the acute phase and biopsy confirmation have found a predominance of B19V, followed by enteroviruses, coxsackievirus B, and HHV.^398^Cases of arboviral diseases – dengue, Zika, and chikungunya – have been described in endemic regions across the world.^399^More recently, new presentations have emerged with the SARS-CoV-2 pandemic, including myocardial injury associated or not with multisystem inflammatory syndrome whose pathophysiology remains unclear.^400^Survivors of childhood cancer treatment, especially those undergoing treatment with anthracyclines and ICIs, are at high risk for the onset of an inflammatory process leading to HF in adulthood.^401^

**Table 35:** - Most common clinical findings at initial presentation of myocarditis in children and adolescents

Signs and symptoms	Below 2 years of age	Preschoolers	School-age children and adolescents
**Specific**	Signs of HF	Signs of HF	Signs of HF
	History of viral disease in the past 3 to 6 weeks	History of viral disease in the past 3 to 6 weeks	Chest pain
Chest pain (uncommon)	Chest pain (unlikely)	History of viral disease may not be so clear
**Nonspecific**	Fever	Dyspnea on exertion	Dyspnea on exertion
	Lethargy	Tachycardia at rest	Tachycardia at rest
Irritability	Muscle fatigue	Muscle fatigue
Perfusion change	Arrhythmia	Arrhythmia
Decreased intake	Shock	Shock
Tachycardia at rest		
Arrhythmia
Shock

*HF: heart failure.*

**Figure 15:** – Flowchart for investigation of suspected myocarditis in children and adolescents.

BNP: brain natriuretic peptide; CMR: cardiac magnetic resonance; CNS: central nervous system; CRP: C-reactive protein; CV: cardiovascular; EMB: endomyocardial biopsy; GI: gastrointestinal; HF: heart failure; LVEF: left ventricular ejection fraction.

### 6.8.2. Prognosis

Estimating the incidence and prevalence of pediatric myocarditis is challenging because of the wide spectrum of symptoms, which may range from a mild viral infection without hemodynamic compromise to congestive HF with ventricular dysfunction, arrhythmias, and sudden death.^164 , 402 - 405^As the symptoms are often nonspecific, a significant number of cases are not diagnosed, which makes the actual incidence and prognosis difficult to characterize. However, this is the most common etiology of dilated cardiomyopathy in children.

With improvements in intensive care services, including mechanical circulatory support, the prognosis of children of all age groups has improved, and complete recovery has been possible even in cases of fulminant disease.^402^

Key outcomes in pediatric patients include complete recovery, progression to dilated cardiomyopathy, and death or heart transplantation.^405^

Children with viral myocarditis are believed to have a better prognosis than those with dilated cardiomyopathy. Survival in pediatric patients with myocarditis is up to 93%. However, a multicenter study addressing all age groups demonstrated a significant mortality in neonates and infants. Survival in this group ranged from 33% to 45%, and clinical improvement ranged from 23% to 32%. In children aged 1 to 18 years, survival was higher, approximately 78% to 80%, and clinical improvement ranged from 46% to 67%.^406^In a recent Pediatric Cardiomyopathy Registry (PCMR) study, children with biopsy-proven myocarditis had a 3-year survival of 75%; also, 54% had normalized ventricular dimensions and function, and only 20% showed persistent echocardiographic abnormalities.^404^

A study of 28 patients with confirmed myocarditis reported that only 17 survived and were discharged showing varying degrees of improved cardiac function. The remaining 11 patients progressed to refractory HF; seven required heart transplantation, and four died. Predictors of poor prognosis were ejection fraction below 30%, fractional shortening below 15%, left ventricular dilatation, and moderate-to-severe mitral regurgitation.^406^

Several case series of children requiring mechanical circulatory support for myocarditis have reported a survival rate between 67% and 83%. Of 21 patients mechanically supported with the Berlin Heart EXCOR device for myocarditis or dilated cardiomyopathy, 90% survived and were discharged.^407^

Prognosis in EMB-proven myocarditis depends on the severity of symptoms, histological classification, and biomarkers. Acute fulminant myocarditis is associated with higher survival. Giant cell myocarditis, although rare, is associated with a poor prognosis; median survival is 5.5 months, and mortality or transplantation rate is 89%.^406^

Myocarditis accounts for at least 50% of all dilated cardiomyopathies in childhood. The outcome of patients with viral myocarditis is better than that of patients with dilated cardiomyopathy. For this reason, myocarditis should always be suspected, and supportive measures should be initiated as early as possible to prevent a patient with myocarditis from being placed on a transplant waiting list without having a chance of recovery. The indication for transplantation in myocarditis should only be considered when recovery is unfavorable despite adequate therapeutic management ( Table 36 ).

**Table 36:** - Key information about myocarditis in children and adolescents

Myocarditis in children and adolescents
Key outcomes include complete recovery, progression to dilated cardiomyopathy, and death or heart transplantation
Intravenous immunoglobulin has become a standard practice in the treatment of myocarditis, but its effect on cardiac function is still not fully understood
The spectrum of clinical manifestations of myocarditis is very wide, ranging from a mild viral infection to congestive heart failure with cardiogenic shock requiring inotropic or mechanical circulatory support
Although endomyocardial biopsy is considered the gold standard for diagnosis of myocarditis, the risk of adverse events in children ranges from 1% to 5% (tachyarrhythmias, hypotension after anesthesia, ischemic changes, ventricular perforation). Therefore, this technique has not been routinely adopted^5^
Ejection fraction <42% and elevated troponin at diagnosis are most significantly associated with mortality
Patients who survive the acute phase have better long-term outcomes than those who have a more insidious condition
Myocarditis is the most common etiology of dilated cardiomyopathy in childhood

IVIg therapy has been included in immunomodulatory treatment of children with acute myocarditis at many centers, being used at a standard dose of 2 g/kg over 24 hours. This practice has been established since the classic 1994 study conducted by Drucker et al.^164^A tendency towards ventricular function recovery has been demonstrated in those who received immunoglobulin. In a cohort of 94 patients with new-onset cardiomyopathy, IVIg was administered to 22% of patients, and 5-year follow-up data have demonstrated a higher rate of recovery compared to patients who did not receive immunoglobulin.^408^

A Taiwanese study of 94 patients evaluated receiver-operating characteristic (ROC) curves and found that ejection fraction <42% (sensitivity, 86.7% and specificity, 82.8%) and troponin I >45 ng/mL (sensitivity, 62.6% and specificity, 91%) were most significantly associated with mortality.^403^

Several studies have shown that patients who survive the initial acute phase have a more favorable long-term outcome, unlike those with more insidious disease.

Histological evidence of myocarditis as a cause of dilated cardiomyopathy has been considered a positive prognostic indicator for recovery, with chances of cure ranging from 50% to 80% within 2 years.^402^Likewise, a delayed progression to chronic HF requiring heart transplantation may occur even after an initial clinical improvement.

## 6.9. Myocarditis with pericardial involvement

### 6.9.1. Diagnosis and treatment

Myocarditis and pericarditis are often associated in clinical practice, representing different spectra within the group of inflammatory myopericardial syndromes ( Table 37 ).^409 , 410^This is explained by both conditions sharing common etiological agents, mainly viruses.^411^However, myocardial and pericardial involvement are rarely of equivalent intensity (there is either predominance of myocarditis [perimyocarditis] or pericarditis [myopericarditis]^412^), and differentiating between the two conditions is important for prognosis and treatment. Myopericarditis usually has a good prognosis, without progression to HF or constrictive pericarditis.^413 - 416^In the setting of acute myocarditis, pericardial involvement (perimyocarditis) has prognostic significance. Di Bella et al.^417^evaluated a cohort of 467 patients with idiopathic/viral acute myocarditis diagnosed by CMR and identified that approximately 24% of patients showed pericardial involvement. In addition, pericarditis caused a 2.5-fold increase in the risk of cardiac events (composite endpoint of death, cardiac transplantation, implantable cardioverter-defibrillator, and hospitalization due to decompensated HF).^416^

**Table 37:** - Recommendations for the evaluation of myocarditis with suspected pericardial involvement

Indication	Class	Level of evidence
In patients with acute myocarditis and suspected pericardial involvement, cardiac magnetic resonance is recommended to support the diagnosis in doubtful cases.	I	C

Myocarditis associated with acute pericarditis should be suspected in patients with a diagnosis of myocarditis and at least two of the following criteria: pleuritic chest pain (may be difficult to identify because of pain due to myocardium involvement), pericardial friction rub; ECG changes suggestive of pericarditis (widespread ST-segment elevation, PR-segment depression); and new or worsening pericardial effusion. Laboratory tests usually reveal leukocytosis with a predominance of lymphocytes (in viral cases) and elevated CRP and ESR. CMR is the most accurate noninvasive test for evaluating pericardial involvement in patients with myocarditis.^409 , 417^It detects inflammation, thickening, effusion, and masses in the pericardium and is indicated for cases whose diagnosis is unclear (grade of recommendation: I, level of evidence: C).^409 , 418^

In patients with myocarditis and pericardial involvement, treatment essentially depends on the underlying cause and should follow the recommendations for the treatment of myocarditis. In viral/idiopathic cases without ventricular dysfunction, the use of nonsteroidal anti-inflammatory drugs (NSAIDs) to control pericardial injury should be considered with caution using reduced doses, given that experimental studies have shown increased mortality and enhanced myocardial inflammation with NSAIDs.^411 , 419 , 420^

## 6.10. Acute myocarditis mimicking MI

Previous studies have indicated that 2.6% to 25% of patients with suspected MI actually have MINOCA. Several etiologies may be attributed to patients with suspected acute MI with culprit-free angiograms, among which acute myocarditis has been recognized as a particularly important factor.^421^

Typical clinical presentations of acute MI, such as chest pain, ST-segment elevation, and incremental serum markers, are commonly observed in patients diagnosed with myocarditis.^422 , 423^In addition, in the clinical setting of acute disease with elevated troponins, it may be clinically challenging to differentiate type 2 MI from nonischemic causes of myocardial injury, especially myocarditis. Type 2 MI is secondary to ischemia due to increased oxygen demand or decreased supply; it may be caused by coronary artery spasm, coronary embolism, anemia, arrhythmias, hypertension, or hypotension.^424^

“Myocardial ischemia” is used when there is evidence of elevated troponin values with at least one value above the 99th percentile upper reference limit. “Myocardial lesion”, in turn, is used when there is an increase or decrease in troponin values. The diagnosis of acute MI is specified when there is acute myocardial injury with clinical evidence of acute myocardial ischemia, requiring both the detection of an increase and/or decrease in troponin values and the presence of at least one of the following conditions: symptoms of myocardial ischemia, new ischemic ECG changes, development of pathological Q waves, imaging evidence of new loss of viable myocardium, new wall motion abnormalities in a pattern consistent with an ischemic event, and/or identification of a coronary thrombus on angiography or autopsy.^424^

The clinical entities that may mimic ST-segment elevation MI include myocarditis/pericarditis, Takotsubo cardiomyopathy, J-wave syndromes (used to describe both Brugada syndrome and early repolarization syndrome), secondary repolarization abnormalities (eg, left bundle branch block, ventricular pacing, and ventricular hypertrophy), electrolyte disorders (hyperkalemia and hypercalcemia), and other nonischemic causes (eg, Wolff Parkinson-White syndrome, pulmonary embolism, intracranial bleeding, hypothermia, and postcardioversion). However, the course of ECG changes and differences in clinical history may help distinguish these conditions from acute MI.^425^

In vivo tissue characterization with CMR enables the identification of edema/inflammation in ACS/myocarditis and the diagnosis of chronic diseases and fibrotic conditions (eg, in hypertrophic and dilated cardiomyopathies, aortic stenosis, and amyloidosis).^425^In nonischemic diseases, the pattern and distribution of late gadolinium enhancement may offer clues regarding etiology and prognostic significance.^425^Myocarditis usually causes subepicardial/midmyocardial scarring, typically (though not always) showing a noncoronary distribution with subendocardial sparing.^426 , 427^

In myocarditis, T2-weighted imaging may also identify regional inflammation, characteristically showing a noncoronary distribution. Conversely, parametric T1-mapping is also available and provides a quantitative and objective assessment of edema/inflammation (eg, in acute MI/myocarditis).^426 , 427^There is a dynamic interaction between inflammation and fibrosis in different precursors of HF, such as acute MI and myocarditis. Early diagnosis of HF with biomarkers and imaging tests is imperative; whereas CMR is useful for evaluating the extent of the injury, serial biomarker measurements indicate if inflammation and fibrosis are progressive.^427^

Clinically, myocarditis mimicking acute MI is an extremely complex case for physicians to accurately diagnose. The coronary anatomy must be investigated either with coronary angiography or coronary CT angiography. Furthermore, a correct diagnosis of myocarditis per se is a challenge due to nonspecific patterns of clinical presentation and the lack of a reliable and accurate diagnostic method. Although EMB is recommended in guidelines as the ideal diagnostic method, the diagnosis of myocarditis in routine practice is usually based on comprehensive consideration of medical history, clinical manifestations, and additional tests, among which CMR has significant advantage for detecting myocardial abnormalities and accurately discriminating patients with myocarditis from those with true MI.^421 - 423 , 425 - 427^ Figure 16 shows a flowchart for evaluating patients with acute MI versus myocarditis.

**Figure 16:** – Differential diagnosis of chest pain: acute MI versus myocarditis.

CMR: cardiac magnetic resonance; ECG: electrocardiogram; ECHO: echocardiogram; EMB: endomyocardial biopsy; MI: myocardial infarction; Tn: troponin.

## 7. Rheumatic carditis

In 2018, the World Health Organization (WHO) acknowledged that rheumatic fever is endemic in low-income countries and developed a global action plan focused on prevention, diagnosis, and secondary prophylaxis.^428^Rheumatic fever is a biphasic disease whose acute outbreak manifests as variable combinations of arthritis, chorea, subcutaneous and cutaneous injuries, and myocarditis, which affects more than 50% of patients.^429^Approximately 5% of patients with acute rheumatic myocarditis have significant clinical manifestations that require medical attention, and up to 50% of patients with acute carditis progress to chronic rheumatic heart disease (late stage), specifically mitral and/or aortic valve disease.^430 , 431^The prevalence of rheumatic carditis is unknown, but data suggest that this is a common and underdiagnosed condition. In 2013, the Brazilian Unified Health System (SUS) reported 5,169 hospitalizations due to acute rheumatic fever.^432^Approximately 40 million people worldwide are estimated to currently live with chronic rheumatic heart disease, and this condition is believed to account for approximately 300,000 deaths annually.^433^A Brazilian study included 5,996 students from 21 schools in the state of Minas Gerais and identified a prevalence of chronic rheumatic heart disease of 0,42%, which is 2- to 10-fold higher than the mean rate documented in developed countries.^434^

Rheumatic carditis should be suspected upon an acute outbreak of rheumatic fever, initially by applying the Jones criteria, which were updated in 2015.^435^Patients should be stratified according to epidemiological considerations regarding the risk of rheumatic disease. Patients from regions where the incidence of rheumatic fever is higher than 2/100,000 school-aged children (5 to 14 years) per year or the prevalence of rheumatic valve disease is higher than 1/1,000 person-years are considered at high risk. A large portion of the Brazilian population is believed to live in regions with such characteristics. The 2015 update of the Jones criteria also included echocardiographic criteria and the possibility of using the criteria to diagnose recurrent rheumatic fever^436^( Table 38 ). Therefore, a rheumatic etiology should be considered for patients with carditis, especially young people in low-income regions and/or with a history of rheumatic valve disease.

**Table 38:** – 2015 update of the Jones criteria

First outbreak of rheumatic fever	Rheumatic fever recurrence
2 major criteria; or 1 major criterion and at least 2 minor criteria	2 major criteria; or 1 major criterion and at least 2 minor criteria; or 3 minor criteria
Low-risk population (<2/100,000 cases of acute rheumatic fever per year and <1/1,000 cases of rheumatic valve disease per year)	Moderate- or high-risk population (<2/100,000 cases of acute rheumatic fever per year and <1/1,000 cases of rheumatic valve disease per year)
**Major criteria**	**Major criteria**
– Carditis (clinical or subclinical)	– Carditis (clinical or subclinical)
– Arthritis (polyarthritis only)	– Arthritis (polyarthritis only, polyarthralgia, and/or monoarthritis)
– Chorea	– Chorea
– *Erythema marginatum*	– *Erythema marginatum*
– Subcutaneous nodule	– Subcutaneous nodule
**Minor criteria**	**Minor criteria**
– Polyarthralgia	– Monoarthralgia
– Fever (≥38.5°C)	– Fever (≥38°C)
– Elevated ESR (>60 mm in the first hour) and/or CRP > upper reference limit)	– Elevated ESR (>60 mm in the first hour) and/or CRP > upper reference limit)
– Prolonged PR interval corrected for age (in the absence of carditis)	– Prolonged PR interval corrected for age (in the absence of carditis)
Evidence of a preceding group A β-hemolytic streptococcal infection (positive throat culture; positive rapid test; scarlet fever; increased titers of anti-streptococcal antibodies)	Evidence of preceding group A β-hemolytic streptococcal infection (positive throat culture; positive rapid test; scarlet fever; increased titers of anti-streptococcal antibodies)

*CRP: C-reactive protein; ESR: Erythrocyte sedimentation rate.*

In case of a documented acute outbreak of rheumatic fever or clinical manifestations of HF, an active investigation for rheumatic carditis is warranted. Rheumatic carditis is a pancarditis that affects the pericardium, myocardium, and endocardium in varying degrees. It mainly manifests through acute valvulitis, which affects 90% of cases as mitral and/or aortic acute regurgitant valvular disease.^437^In the presence of symptoms, the main mechanism is acute valvular disease (preferably mitral) and, less frequently and less intensely, myocarditis and pericarditis.^438^Therefore, the initial focus of investigation is the detection of valvular heart disease, which can be achieved by physical examination; however, transthoracic echocardiography is required, and transesophageal evaluation is recommended for uncommon situations with an inadequate window.^439^A 12-lead ECG, besides detecting a prolonged PR interval, may also detect a long QT and changes consistent with pericarditis and left chamber overload.^440^Troponin and CKMB levels are usually low, which indicates minimal myocardial damage.^431 , 441^Chest radiography may be helpful in identifying cardiomegaly and congestion.^442^After the initial evaluation, there are four possible diagnostic hypotheses:^443 , 444^

- Subclinical carditis: clinical examination without alarming changes, prolonged PR interval on ECG, and/or mild mitral and/or aortic regurgitation on Doppler echocardiography.
- Mild carditis: tachycardia disproportionate to fever, detectable regurgitant murmur, prolonged PR interval on ECG, chest radiograph without alarming changes, and mild to moderate mitral and/or aortic regurgitation on Doppler echocardiography.
- Moderate carditis: mild carditis criteria associated with mild symptoms of HF and/or prolonged QT and/ or cardiomegaly and congestion on radiograph and/or mild-to-moderate left-chamber dilatation.
- Severe carditis: limiting HF symptoms with significant valve regurgitation and/or significant cardiomegaly and/or systolic ventricular dysfunction.

Thus, rheumatic myocarditis per se is not very exuberant and should be suspected in the presence of criteria for rheumatic carditis, manifest HF, and no anatomically significant acute valvular disease. In this situation, a thorough evaluation of possible differential diagnoses of myocarditis is also essential.

Mild, moderate, and severe cases should be investigated with additional imaging testing. Gallium-67 scintigraphy is the most widely studied test, is highly specific and sensitive, and should be the first to be conducted.^445 , 446^Antimyosin scintigraphy and PET scanning are less sensitive but may be conducted if gallium-67 is unavailable or if there is evidence of other differential diagnoses.^447 , 448^Studies focusing on the use of MRI for rheumatic fever are lacking, especially because valvular involvement is predominant; thus, MRI is more useful for differential diagnoses.^449^EMB has low sensitivity but extremely high specificity, and the presence of Aschoff nodules is pathognomonic of rheumatic myocarditis. It is indicated for refractory or severe cases^450^( Table 39 ).

**Table 39:** – Diagnostic tests for rheumatic carditis

Indications	Class	Level of evidence
12-lead ECG	I	B^440^
Chest radiograph	I	C^442^
Transthoracic Doppler echocardiography	I	B^436,439^
Transesophageal Doppler echocardiography if difficult transthoracic visualization	I	C^436,439^
ESR and CRP (see the Jones criteria)	I	B^436^
Antistreptolysin O (see the Jones criteria)	I	C^436^
Antidesoxyribonuclease B as an alternative to anti-streptolysin O	IIa	C^435^
Alpha-1 acid glycoprotein for inflammatory activity monitoring	IIa	C^444^
Protein electrophoresis (alpha-2 globulin) for inflammatory activity monitoring	IIa	C^444^
Troponin as a diagnostic criterion	IIb	B^431,441^
Gallium-67 scintigraphy	IIa	B^445,446^
^18^F-FDG PET/CT	IIb	B^448^
Cardiac magnetic resonance	IIb	C^449^
Endomyocardial biopsy	IIb	C^444,450^

*CRP: C-reactive protein; ECG: electrocardiogram; ESR: Erythrocyte sedimentation rate.*

For all patients with rheumatic carditis, despite being a late immune response, streptococcal eradication is recommended.^451^The treatment of subclinical and mild presentations includes controlling the symptoms associated with acute outbreaks and monitoring disease progression. Moderate and severe cases should be treated with corticosteroids (initially via oral route) and pulse therapy if refractory disease.^452 - 455^Medications such as ACEIs, furosemide, spironolactone, and digoxin should be used if manifest HF.^450^Refractory disease means that valve surgery should be considered in the acute phase ( Table 40 ).^456 - 457^

**Table 40:** – Treatments for rheumatic carditis

Indications	Class	Level of evidence
Eradication of group A β-hemolytic *Streptococcus* : – Penicillin G benzathine 1,200,000 IU, deep IM, single dose for those >20kg – Penicillin G benzathine 600,000 IU, deep IM, single dose for those <20kg – Amoxicillin 50 mg/kg/day (maximum 1,500 mg) in 3 divided doses for 10 days – For those with penicillin allergy – erythromycin 40 mg/kg/days (maximum 1,000 mg) in 4 divided doses for 10 days	I	C^444, 451^
Rest if moderate or severe case	IIa	C^444^
Hospitalization for symptom control in moderate-to-severe carditis	IIa	C^444^
Prednisone 0.5 to 1 mg/kg/day (maximum 50 mg) orally; may be divided into 2 to 3 daily doses for 15 days, with subsequent 20% weekly dose reductions in subclinical or mild cases. Total duration: 4 to 8 weeks.	IIb	B^444,452,453^
Acetylsalicylic acid 100 mg/kg (maximum 3 to 4 g) in 4 divided doses or naproxen 20 mg/kg (maximum 1,000 mg) in 2 divided doses for subclinical cases with associated arthritis and/or pericarditis. Total duration: 2 weeks.	I	B^444,453^
Prednisone 1 to 2 mg/kg/day (maximum 60 mg) orally; may be divided into 2 to 3 daily doses for 15 days, with subsequent 20% weekly dose reductions in moderate-to-severe cases. Total duration: 12 weeks.	I	B^444,452,453^
Methylprednisolone 30 mg/kg/day in weekly cycles in severe cases or refractory to initial treatment.	IIb	B^454,455^
In the presence of signs/symptoms of ventricular dysfunction, treat HF with diuretics and neurohormonal blockers.	I	C^450^
Cardiac valve surgery in mild and refractory cases: – Mitral repair with a technique that allows annular growth – Preferred mechanical prosthesis for aortic replacement	I	B^456,457^

*HF: heart failure.*

## 8. Myocarditis due to autoimmune diseases

Cardiac involvement in autoimmune diseases may include the pericardium, myocardium, endocardium, valves, and coronary arteries. Regarding myocarditis, a few entities warrant special attention: sarcoidosis, giant cell myocarditis, Behcet disease, eosinophilic granulomatosis with polyangiitis, systemic lupus erythematosus, scleroderma, and rheumatoid arthritis. There are obvious limitations regarding the diagnosis of myocarditis and its prevalence in autoimmune diseases, but it should be considered when there are signs and symptoms suggestive of cardiac involvement, including arrhythmias, syncope, HF, chest pain, or elevated markers of myocardial necrosis, especially in patients with a history of autoimmune disease or when there is cardiac involvement associated with symptoms of inflammation affecting other systems.

Nonspecific inflammatory markers, including CRP/ESR and myocardial injury markers such as troponin and BNP, are usually elevated. ECG and echocardiography should be performed in all patients with autoimmune diseases when cardiac involvement is suspected.^12 , 188^MRI is a sensitive and specific method for the evaluation of myocarditis, in addition to providing further information for differential diagnosis.^458 , 459^PET is another noninvasive method of choice, especially for suspected sarcoidosis.^243^Autoimmunity markers such as antinuclear antibodies, rheumatoid factors, and antineutrophil cytoplasmic antibodies should be considered, and testing should be guided by clinical suspicion.^460^EMB is the gold standard for the diagnosis of myocarditis due to autoimmune diseases or other causes. EMB uses other techniques in addition to histology to differentiate infectious from noninfectious myocarditis as well as to identify vasculitis and other noninflammatory myocardial diseases.^151^The treatment of myocarditis due to autoimmune diseases was discussed elsewhere in this Guideline.

## 9. Management of cardiac arrhythmias in myocarditis

### 9.1. Noninvasive and invasive assessments of arrhythmias in the acute and chronic phases of the several causes of myocarditis

Cardiac arrhythmias are relatively common manifestations in patients with myocarditis and may appear at any phase of the disease. Arrhythmogenic mechanisms are directly and indirectly associated with the degree of myocardial inflammation.^55^

In the acute phase by viral aggression and inflammatory response, we have myocytolysis associated with fibrosis, which promote hyperactivity of the sympathetic system and ion channel dysfunction, especially in calcium regulation, creating the electrophysiological substrate for genesis of arrhythmias.^461^The higher the cell damage and the degree of inflammatory involvement, the higher the likelihood of ventricular arrhythmia, with reentry being the main arrhythmogenic mechanism.

A broad spectrum of bradyarrhythmias and tachyarrhythmias occur in the setting of myocarditis. AV block, changes in ventricular repolarization, and prolonged QT interval are common findings in the acute phase of the disease. Atrial fibrillation and atrial tachycardias may also occur in acute or chronic myocarditis.

Ventricular arrhythmias may manifest as extrasystoles and/or ventricular tachycardias. These conditions may be monomorphic or polymorphic and manifest as nonsustained or sustained (duration ≥30 seconds).

Symptoms vary according to the presentation of arrhythmia, hemodynamic status, and degree of left ventricular dysfunction, and may include palpitations, tachycardia, syncope, or sudden death.

Direct diagnostic methods for noninvasive assessment of arrhythmias include 12-lead baseline ECG, continuous 24- or 48-hour ambulatory ECG (Holter system), and event monitoring (Looper system).

ECG findings are usually abnormal in patients with myocarditis, although they lack specificity and sensitivity.^462^Ukena et al.^94^reported that prolonged QRS duration is an independent predictor of cardiac death or heart transplantation in patients with suspected myocarditis. QTc prolongation ≥440 ms, deviation of the QRS axis, and premature ventricular ectopic beats, which are part of the course of myocarditis, do not seem to be independent predictors of poor prognosis. An ECG is a very useful tool in the detection of sustained bradyarrhythmias and tachyarrhythmias.

Ambulatory ECG monitoring may be useful for recording paroxysmal arrhythmias. Symptom frequency dictates the duration of the recording: the more infrequent the symptoms, the more difficult monitoring is.

Twenty-four-hour ambulatory ECG (Holter) allows documentation of arrhythmias and AV conduction abnormalities. It also contributes to the assessment of the nychthemeral distribution of arrhythmias, the autonomic nervous system, and the probable electrophysiological mechanism.^463^We recommend performing 24-hour Holter during hospitalization to evaluate possible asymptomatic arrhythmias and intermittent AV conduction abnormalities ( Table 41 ). Holter may also be indicated as a supportive method for risk stratification of sudden death in the acute phase of myocarditis.

**Table 41:** - Recommendations for arrhythmia evaluation in acute myocarditis

Indication	Class	Level of evidence
Holter in patients with intermediate-to-high prognostic risk	I	C

In patients with myocarditis, the actual role of invasive electrophysiology in risk stratification of sudden death is still under investigation. Importantly, the reproducibility of significant arrhythmic events should vary according to the cause and type of myocardial involvement.^464^Cardiac sarcoidosis, for example, has a high degree of reproducibility of significant clinical events with programmed electrical stimulation, which is useful in decision-making. In patients who had nonsustained or sustained monomorphic ventricular tachycardia at some point during the course of the disease, the presence of significant late gadolinium enhancement or low-voltage areas on electrophysiology studies with electroanatomic mapping seems to indicate poor prognosis, and these findings may help to stratify the risk of sudden death.^465^In the absence of specific data, this method of risk stratification for sudden death should be used with caution, especially in asymptomatic patients.^259^

### 9.2. Arrhythmia treatment and sudden death prevention in the acute and subacute phases

Arrhythmias are mostly associated with myocarditis during the acute phase but may also appear during the chronic phase, depending on the degree of tissue damage (especially inflammation and residual fibrosis), showing a wide physiological basis ( Table 42 ).^466 , 467^Arrhythmias may occur in 33.7% of hospitalizations due to myocarditis, manifesting as both tachycardia and bradyarrhythmia, and are associated with morbidities such as hyperthyroidism, age, obesity, HF, electrolyte imbalance, and valvular disease.^95^Preexisting cardiomyopathies such as arrhythmogenic RV dysplasia and preexisting channelopathies are also associated with the occurrence of arrhythmias in myocardial inflammation.^468 , 469^

**Table 42:** - Potentially triggering mechanisms of arrhythmia in patients with myocarditis

Direct viral injury generating myocardial cell lysis and innate immune response Viral persistence
Cell apoptosis
Fibrosis favoring reentry mechanisms Proarrhythmic effect of cytokines
Changes in cell gap junctions Infarction due to microvascular injury

Although rare, bradyarrhythmias are usually associated with AV blocks of varying degrees and mostly occur in the acute phase. Obongayo et al.^92^identified a 1.7% prevalence of AV block among hospitalized patients with myocarditis taken from the Nationwide Inpatient Survey, of which only 1.1% were high-degree AV blocks. Third-degree advanced AV block was associated with increased morbidity and mortality.

Atrial fibrillation may occur in up to 9% of hospitalized patients with acute myocarditis and is associated with increased hospital mortality (OR: 1.7; 95% CI: 1.1 to 2.7; p = 0.02), cardiogenic shock (OR: 1.9; 95% CI 1.3 to 2.8; p = 0.001), and cardiac tamponade (OR: 5.6; 95% CI: 1.2 to 25.3; p = 0.002).^470^

Ventricular arrhythmias have the highest probability of sudden death and may account for approximately one fourth of all arrhythmias reported in hospitalized patients with myocarditis. Ventricular tachycardia is the most frequent type.^95^

Arrhythmia management in the acute phase should be consistent with the transient nature of the process, and recurrent ectopia or nonsustained tachycardia should not be treated with specific antiarrhythmics, except beta-blockers when indicated. During this phase, a temporary pacemaker may be used for advanced AV block, and the indication for a definitive pacemaker or implantable cardioverter-defibrillator should follow conventional indications ( Table 43 ).

**Table 43:** - Treatment and prevention of myocarditis-associated arrhythmia and sudden death

Indications	Class	Level of evidence
Treatment with beta-blockers, spironolactone, and sacubitril/valsartan for patients with LV systolic dysfunction	I	C
Temporary pacemaker for symptomatic bradyarrhythmias and/or advanced AV block in the acute phase of myocarditis	I	C
Antiarrhythmic therapy with amiodarone in symptomatic NSVT or sustained VT in the acute phase of myocarditis	I	C
ICD implantation for primary prevention of sudden death in patients with dilated cardiomyopathy in the chronic phase (>6 months) of myocarditis with optimized clinical treatment for classes II and III, LVEF ≤35%, and life expectancy of at least 1 year	IIa	C
Indication for ICD in the acute and subacute phases of myocarditis (<6 months)	III	C
Indication for antiarrhythmic agents for primary prevention of cardiac arrhythmias in patients with myocarditis	III	C

*AV: atrioventricular; ICD: implantable cardioverter-defibrillator; LV: left ventricular; LVEF: left ventricular ejection fraction; NSVT: nonsustained ventricular tachycardia; VT: ventricular tachycardia.*

## 10. Prognostic evaluation and follow-up

### 10.1. Prognosis and evolution markers

Myocarditis has a wide phenotypic diversity. Most individuals with acute myocarditis who also develop acute dilated cardiomyopathy improve within a few days.^14^Case series report rates between 10% and 20% of serious cardiovascular events in the long term and a risk of recurrence of 10%.^109^

Several clinical and laboratory factors are involved in prognosis. Maintenance of preserved ventricular function during the acute phase has been repeatedly associated with spontaneous improvement and no sequelae.^15^Other studies have reported that reduced levels of blood pressure and heart rate, syncope, RV systolic dysfunction, elevated pulmonary arterial pressure, and advanced NYHA functional class play an important role.^94^Etiology has also been shown to be valuable in the prognostic spectrum. Patients with acute lymphocytic myocarditis and preserved ventricular function showed spontaneous improvement and no sequelae. In contrast, the MTT study reported that patients with HF and LVEF <45% had a 4-year mortality rate of 56%. The course of giant cell and eosinophilic myocarditis is more dismal.^14^Patients with fulminant myocarditis have a dramatic short-term prognosis; however, when they survive, the prognosis is better compared to other causes.^17 , 98^

ECG was shown to have prognostic value in a recent study.^471^MRI, whose value for the diagnosis of myocarditis is outstanding, has been shown to be useful with late gadolinium enhancement;^109^however, a more recent study could not confirm the predictive value of MRI for long-term improvement or remodeling of ventricular function.^472^Despite advances in diagnosis, prognosis remains a challenge, probably due to numerous known and unknown factors as well as the different causes of myocarditis, which vary widely in terms of characteristics, clinical presentation, and genetic and immunological involvement, among others.^137^

### 10.2 Outpatient follow-up with additional evaluations

Clinical follow-up with ECG should be continuous in patients who have already been diagnosed. Given the undeniable value of ventricular function, imaging tests should also be performed. Echocardiography emerges as a useful and easily accessible alternative, providing the most relevant information in this setting ( Table 44 ).

**Table 44:** - General follow-up recommendations in myocarditis473,474

Indications	Class	Level of evidence
Clinical follow-up of low-risk patients with ECG at 1, 3, 6, and 12 months, then annually	I	C
Clinical follow-up of low-risk patients with echocardiogram at 1, 6, and 12 months, then annually	I	C
For intermediate-risk patients, clinical and laboratory evaluation with Holter monitoring and imaging tests should be performed at 1, 3, and 6 months (echocardiogram and/or MRI according to availability), then annually	I	C
For high-risk patients with myocarditis, clinical and laboratory follow-up with Holter monitoring and imaging tests should be performed at 15 days, 1, 3, and 6 months (echocardiogram or MRI according to availability), then annually	I	C

*CG: electrocardiogram; MRI: magnetic resonance imaging.*
